# Resolving Sphingolipid Isomers Using Cryogenic Infrared Spectroscopy

**DOI:** 10.1002/anie.202002459

**Published:** 2020-05-18

**Authors:** Carla Kirschbaum, Essa M. Saied, Kim Greis, Eike Mucha, Sandy Gewinner, Wieland Schöllkopf, Gerard Meijer, Gert von Helden, Berwyck L. J. Poad, Stephen J. Blanksby, Christoph Arenz, Kevin Pagel

**Affiliations:** ^1^ Institut für Chemie und Biochemie Freie Universität Berlin Arnimallee 22 14195 Berlin Germany; ^2^ Abteilung Molekülphysik Fritz-Haber-Institut der Max-Planck-Gesellschaft Faradayweg 4–6 14195 Berlin Germany; ^3^ Institut für Chemie Humboldt-Universität zu Berlin Brook-Taylor-Straße 2 12489 Berlin Germany; ^4^ Chemistry Department Faculty of Science Suez Canal University Ismailia Egypt; ^5^ Central Analytical Research Facility Institute for Future Environments Queensland University of Technology Brisbane QLD 4000 Australia

**Keywords:** deoxysphingolipids, double-bond isomers, IR spectroscopy, isomers, mass spectrometry

## Abstract

1‐Deoxysphingolipids are a recently described class of sphingolipids that have been shown to be associated with several disease states including diabetic and hereditary neuropathy. The identification and characterization of 1‐deoxysphingolipids and their metabolites is therefore highly important. However, exact structure determination requires a combination of sophisticated analytical techniques due to the presence of various isomers, such as ketone/alkenol isomers, carbon–carbon double‐bond (C=C) isomers and hydroxylation regioisomers. Here we demonstrate that cryogenic gas‐phase infrared (IR) spectroscopy of ionized 1‐deoxysphingolipids enables the identification and differentiation of isomers by their unique spectroscopic fingerprints. In particular, C=C bond positions and stereochemical configurations can be distinguished by specific interactions between the charged amine and the double bond. The results demonstrate the power of gas‐phase IR spectroscopy to overcome the challenge of isomer resolution in conventional mass spectrometry and pave the way for deeper analysis of the lipidome.

## Graphical abstract

**Much attention** has been paid recently to deoxysphingolipids, while their important roles in several diseases have been unveiled. Their structural characterization is, however, hampered by the presence of ketone/alkenol and double‐bond regio‐/stereoisomers. Cryogenic gas‐phase infrared spectroscopy was employed to resolve all types of isomerism by exploiting subtle intramolecular interactions.

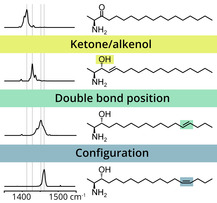

Sphingolipids are ubiquitous in all living organisms ranging from bacteria to humans.^1^ They are major components of cell membranes and involved in essential biological processes such as intra‐ and intercellular signaling.^2^ Complex sphingolipids including nonpolar ceramides and polar phospho‐ or glycosphingolipids are usually built up from one of the three most abundant sphingoid bases—sphingosine (SO), sphinganine (SA), or phytosphinganine (PS).^3^ The de novo biosynthesis of any sphingoid base is initiated by the condensation of palmitoyl‐CoA and l‐serine, which is catalyzed by serine palmitoyl transferase (SPT).^4^ SPT can also use l‐alanine as a substrate to form 3‐keto‐1‐deoxySA instead of canonical 3‐ketoSA.^5^ 3‐Keto‐1‐deoxySA and its downstream products (see Figure 1) lack the primary hydroxyl group and are thus termed *1‐deoxysphingolipids*. As a consequence of the missing OH group, 1‐deoxysphingolipids cannot be transformed into phospho‐ or glycosphingolipids and are not degradable via the canonical pathway, which requires phosphorylation of the 1‐hydroxyl group.^6^ Since their first discovery in marine clams^7^ and detection in mammals only one decade ago,^5^, ^8^ 1‐deoxysphingolipids have moved into the focus of interest as their accumulation is related to several diseases. Elevated 1‐deoxysphingolipid levels were found after anoxia‐associated injuries,^9^ in hereditary sensory and autonomic neuropathy type 1 (HSAN1),^5^ and clinically similar diabetic sensory neuropathy.^10^ Furthermore, they are potential plasma markers for predicting the outbreak of pathologies such as type 2 diabetes.^11^

**Figure 1:**
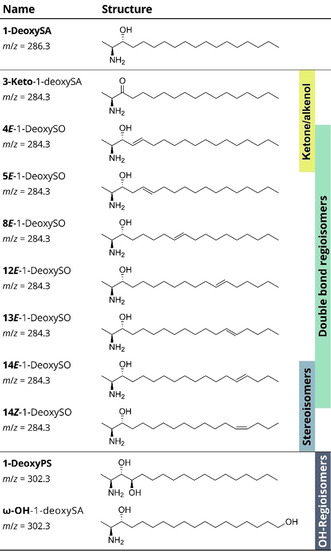
List of investigated 1‐deoxysphingolipids including chemical structures of the molecules and *m*/*z* of the protonated species. Different kinds of isomerism such as ketone/alkenol isomers, C=C bond regio‐ and stereoisomers and OH regioisomers are highlighted.

Despite their seemingly simple structure, the analysis of 1‐deoxysphingolipids is challenging due to the occurrence of different kinds of isomerism. Those involve ketone/alkenol isomers, carbon–carbon double bond (C=C) positional‐ and stereoisomers and hydroxylation (OH) regioisomers, most of which cannot be resolved using established techniques. In particular, C=C bond isomers are not distinguishable without sophisticated techniques such as ozonolysis,^12^ Paternò‐Büchi reactions^13^ or charge‐switching methods^14^ coupled to mass spectrometry (MS). Here we show that the four types of isomerism can be resolved simultaneously by cryogenic IR spectroscopy of 1‐deoxysphingolipid ions in the gas phase.

The basis of our investigation is a consistent set of synthetic 1‐deoxysphingolipids (Figure 1). 3‐Keto‐1‐deoxySA is the primary condensation product of l‐alanine and palmitoyl‐CoA, which is subsequently reduced to 1‐deoxySA. Desaturation of 1‐deoxySA yields 1‐deoxySO, which is an alkenol isomer of 3‐keto‐1‐deoxySA. It was recently shown that the predominant C=C bond position and configuration in 1‐deoxySO is 14*Z*, in contrast to the 4*E* double bond in canonical SO.^15^ Apart from 4*E* and 14*Z*, the C=C bond isomer standards 5*E*, 8*E*, 12*E*, 13*E*, and 14*E* are included in this work. OH Regioisomers are represented by ω‐OH‐1‐deoxySA and 1‐deoxyPS, the deoxy analogue of the most abundant sphingoid base in plants.^3^

Gas‐phase IR spectra of protonated 1‐deoxysphingolipids were obtained by encapsulating the ions in cryogenic helium nanodroplets and irradiating the doped droplets with intense IR light.^16^ Upon vibrational excitation of the ion by the absorption of multiple resonant IR photons, helium atoms evaporate until the bare ion is released and detected by MS. IR spectra are generated by plotting the ion yield as a function of the tunable wavenumber.

The assignment of the characteristic IR bands is exemplified in Figure 2 a, which shows the spectra obtained from the **3‐Keto** and **4*E*** isomers. The ketone and alkenol are readily distinguishable by IR spectroscopy because of the characteristic carbonyl stretching vibration but also by other diagnostic bands. Below 1150 cm^−1^, weak to medium intensity C−O and C−C stretching vibrations of the lipid chain are located. A weak band of the O−H bending vibration in **4*E*** is found below 1400 cm^−1^, whereas the spectra are clearly dominated by the symmetrical NH_3_^+^ umbrella bending modes between 1400 and 1500 cm^−1^. The antisymmetric NH_3_^+^ bending vibrations are located at higher wavenumbers (1550–1650 cm^−1^) and are less intense. The C=C stretching vibration of the double bond in the spectrum of **4*E*** around 1700 cm^−1^ is intrinsically weak, whereas the C=O stretching vibration of the ketone beyond 1700 cm^−1^ is clearly distinguishable. It is important to note that the relative intensities of absorption bands are not reliable due to the nonlinear multiple‐photon absorption process: the ion release from the helium droplet scales nonlinearly with the photon flux. In addition, the regions from 900 to 1150 cm^−1^ and 1550–1800 cm^−1^ were measured with a tighter laser focus (increased fluence) to enhance the visibility of low‐intensity bands.

**Figure 2:**
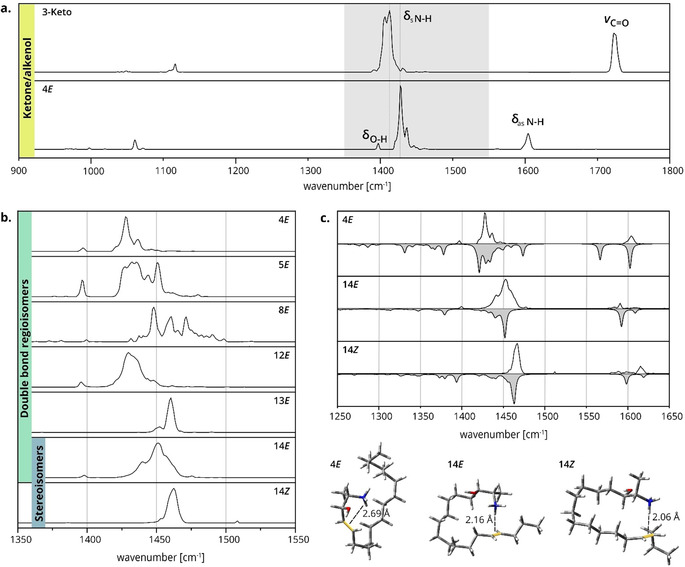
Gas‐phase IR spectra and low‐energy structures of 1‐deoxysphingolipids. a) IR spectra of isomeric 3‐Keto and 4*E*. The ketone and alkenol are distinguishable by diagnostic stretching (ν) and bending (δ) vibrations. The most intense bands in the gray region are assigned to NH_3_^+^ umbrella vibrations. b) Stacked IR spectra of 1‐deoxySO C=C bond regio‐ and stereoisomers in the region of NH_3_^+^ umbrella vibrations (1350–1550 cm^−1^). The absorption patterns and vibrational frequencies depend on the C=C bond position and configuration. c) Spectral matches of 4*E*, 14*E*, and 14*Z* with calculated IR spectra (gray) of DFT‐optimized structures in the region of NH_3_^+^ bending vibrations. The corresponding theoretical structures depicted below highlight the different geometries of charge–olefin interactions.

In contrast to ketone/alkenol isomers that can also be differentiated by conventional chromatography and MS, C=C bond isomers of lipids are usually very difficult to distinguish. The 1‐deoxysphingolipid C=C bond isomers were previously separated by differential‐mobility spectrometry but were found to be indistinguishable by classical drift tube ion mobility spectrometry (DT‐IMS) in nitrogen.^17^ This finding was confirmed by our DT‐IMS measurements in helium yielding identical collision cross sections for all C=C bond regioisomers and a slight difference, within the error limits, for stereoisomers (Table S2). Intuitively, the difficulty in distinguishing C=C bond isomers is also expected to apply to IR spectroscopy as C=C stretching vibrations are generally weak. However, as shown in Figure 2 b, the absorption frequencies of the NH_3_^+^ umbrella modes are significantly shifted depending on the position and configuration of the C=C bond. These frequency shifts are rather surprising but can be explained by a charge–olefin interaction between the protonated amine and the C=C bond, which was predicted in a previous study by Poad et al.^17^ and confirmed by our DFT calculations. The conformational space of the lipid chains was sampled using a genetic algorithm followed by geometry optimization and frequency analysis of selected structures. Spectral matches for the NH_3_^+^ bending vibrations are shown in Figure 2 c for the representative C=C bond regio‐ and stereoisomers **4*E***, **14*E***, and **14*Z***. A charge–olefin interaction was shown to be energetically favored for all three samples. However, substantial differences in the interaction geometry are observed, which is mainly dictated by the distance between the amine and the C=C bond. For example, in the **4*E*** structure, the ammonium proton cannot be centered directly above the C=C bond and the distance between the proton and the C=C bond is longer than that in the **14*E*** and **14*Z*** structures. This difference was already reported by Poad et al., who furthermore observed a deviating behavior of **8*E***, which showed no preference for the charge–olefin interaction. DFT calculations reveal that several low‐energy conformers of **8*E*** favor an interaction between the C=C bond and the hydroxyl proton instead of the ammonium proton or no specific interaction at all (Table S7). Furthermore, the spectrum of **8*E*** contains more than one conformer leading to an unsatisfactory match between experiment and theory (Figure S1). Another interesting observation is the similarity between the IR spectra of **13*E*** and **14*Z*** despite the different position and configuration of the C=C bond. Good qualitative matches of theoretical structures were obtained for both spectra (Figure S1). However, the correlation between experiment and theory is not always perfect. This affects particularly the relative intensities of vibrational bands because of the nonlinear absorption process underlying the experiment. For example, the predicted intensities of antisymmetric NH_3_^+^ bending vibrations are higher than observed throughout all experimental spectra. In addition, it is noteworthy that considerable differences in the N−H stretching vibrations are expected based on the calculated IR spectra (Figure S7). Most of these bands are located around 3000 cm^−1^, a wavelength range that is currently not accessible with the utilized experimental setup.

The study of charge–olefin interactions was extended from mono‐unsaturated sphingolipids to doubly unsaturated 5*E*,14*Z*‐1‐deoxysphingadiene (structure in the Supporting Information). The calculated low‐energy conformers exhibit a sandwich motif, in which the NH_3_^+^ group is wedged in between the two C=C bonds and also interacts with the hydroxyl oxygen (Figure S2). However, the interaction of the NH_3_^+^ group and the C=C bond is disrupted in the presence of a keto group. This was shown for the isomeric 6*E*‐3‐keto‐1‐deoxySO, in which the C=C bond and carbonyl oxygen compete for the interaction with NH_3_^+^ (Figure S2). In the fully saturated reference sample **1‐deoxySA** the ammonium proton coordinates preferentially to the adjacent hydroxyl oxygen, yielding a characteristic NH_3_^+^ umbrella frequency (Figure S1). Finally, the spectra of 1‐deoxysphingolipids were compared with those of 1‐deoxymethylsphingolipids, which are natural condensation products of palmitoyl‐CoA and glycine (Figure S5). 1‐DeoxymethylSA and **1‐deoxySA** yield similar spectra, whereas the absorption frequency of 13*Z*‐1‐deoxymethylSO is slightly shifted compared to the 1‐deoxy analogue **14*Z***. In the absence of the primary methyl group, the NH_3_^+^ group has a larger motional freedom, which allows optimizing the charge–olefin interaction geometry (Figure S3). Overall, the study demonstrates the importance of the intramolecular coordination of NH_3_^+^ to electron‐rich functional groups in the gas phase (Table S19). Those subtle interactions allow for indirect distinction of C=C bond isomers by probing vibrations of the interacting amine. Even though these interactions are restricted to sphingolipids bearing a primary amine, the applicability might be extended to other lipids by modification with coordinating cations prior to spectroscopic interrogation of the lipid. Indeed, ammonium cation adduction is a common strategy for electrospray ionization of neutral lipids while wet‐chemical derivatization of fatty acyl lipids with amine‐functionalized conjugates is also widely deployed to enhance ionization.^18^

Another source of isomerism arises from hydroxylated derivatives of 1‐deoxysphingolipids, which are particularly relevant in the context of 1‐deoxysphingolipid catabolism. The noncanonical sphingolipids are gradually degraded by steps of hydroxylation and desaturation.^6^ Structures of several intermediates have been proposed; however, the positions of hydroxylation remained elusive. **1‐DeoxyPS** and **ω‐OH** were thus investigated as a representative pair of isomers with one fixed and one varying OH position. The corresponding IR spectra differ significantly from each other (Figure 3 a). In addition, DT‐IMS measurements yield CCS differences between the OH regioisomers, which could be an indication of different conformations in the gas phase (Table S2). Quantum chemical calculations suggest that in both cases the NH_3_^+^ group coordinates to both hydroxyl oxygen atoms (Figure 3 b). The terminal OH group of **ω‐OH** has a large motional freedom and can interact with the protonated amine over a short distance in different geometries, whereas the interaction geometry is restricted in the rigid structure of **1‐deoxyPS**. Accordingly, the theoretical spectrum of the calculated **1‐deoxyPS** conformer matches reasonably well with the experimental spectrum, whereas the spectrum of **ω‐OH** cannot be explained with one single conformer (Figure S4). A second pair of OH regioisomers with one varying hydroxyl group are **4*E*** and 4*E*‐3‐deoxySO (Figure S6). Interestingly, the region of NH_3_^+^ umbrella vibrations is almost identical, whereas scaffold vibrations and antisymmetric N−H bending frequencies differ between the isomers. Both examples demonstrate that OH positions can be distinguished by IR spectroscopy, which could enable the characterization of catabolic intermediates.

**Figure 3:**
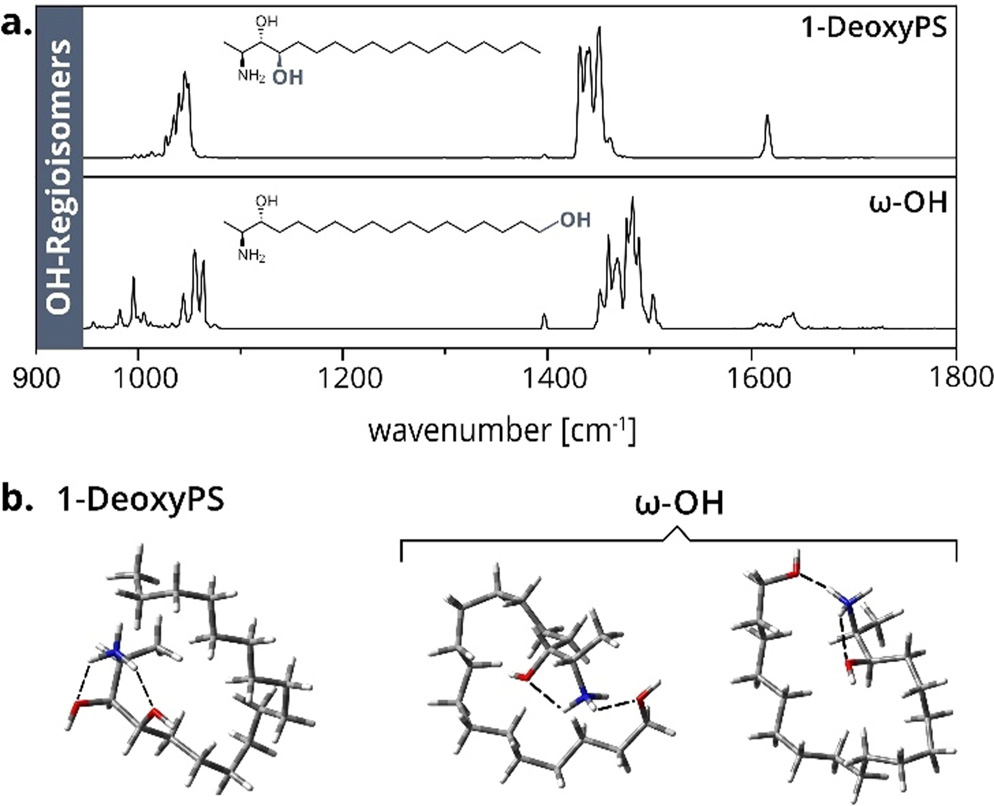
IR spectra and theoretical structures of OH regioisomers. a) IR spectra of the OH regioisomers 1‐deoxyPS and ω‐OH. Both the regions of scaffold vibrations and NH_3_^+^ umbrella vibrations differ significantly. b) Sampled structures of 1‐deoxyPS and ω‐OH. Simultaneous coordination of the NH_3_^+^ group to both hydroxyl groups is favored for both isomers. Several conformers coexist in the case of ω‐OH.

In summary, we show here that all types of 1‐deoxysphingolipid isomers—particularly C=C bond isomers—can be unambiguously resolved as gas‐phase ions using cryogenic gas‐phase IR spectroscopy. Due to its sensitivity towards subtle intramolecular interactions, the method is highly versatile, while requiring only picomolar quantities of sample. Currently, this elegant approach is constrained by the requirement of specialized tunable light sources. However, in the future, a broader application of cryogenic vibrational spectroscopy for structural analysis of lipids may become accessible using tagging IR spectroscopy and exploiting commercially available benchtop optical parametric oscillator or quantum cascade lasers. This would make the experiment technically less elaborate and more accessible, and would allow access to higher wavenumber ranges (≥3000 cm^−1^) to study also N−H and O−H stretching vibrations. Cryogenic gas‐phase IR spectroscopy could thus become a valuable tool for the reliable analysis of lipid isomers that are indistinguishable using established techniques.

## Conflict of interest

The authors declare no conflict of interest.

## Supporting information

As a service to our authors and readers, this journal provides supporting information supplied by the authors. Such materials are peer reviewed and may be re‐organized for online delivery, but are not copy‐edited or typeset. Technical support issues arising from supporting information (other than missing files) should be addressed to the authors.

Supplementary
