# Supplementary material for: Resolving Sphingolipid Isomers Using Cryogenic Infrared Spectroscopy
Source: Angew Chem Int Ed Engl. 2020 May 18;59(32):13638–42. doi: 10.1002/anie.202002459 (PMC7496694; doi:10.1002/anie.202002459)
Supplement: Supplementary file 1 — Supplementary [file ANIE-59-13638-s001.pdf]

Angewandte Chemie
Eine Zeitschrift der Gesellschaft Deutscher Chemiker

Supporting Information

Resolving Sphingolipid Isomers Using Cryogenic Infrared Spectroscopy

Carla Kirschbaum, Essa M. Saied, Kim Greis, Eike Mucha, Sandy Gewinner, Wieland Schöllkopf, Gerard Meijer, Gert von Helden, Berwyck L. J. Poad, Stephen J. Blanksby, Christoph Arenz, and Kevin Pagel**

anie_202002459_sm_miscellaneous_information.pdf

Table of Contents

Supplementary Structures **1**
Table S1: Supplementary structures and *m/z* of protonated deoxysphingolipids. 1
IMS Data: CCS **2**
Table S2: CCS from DT-IMS-MS measurements 2
IR Spectra and Optimized Structures **3**
Figure S1: Saturated and monounsaturated 1-deoxysphingolipids (1200–1800 cm^{-1}). 5
Figure S2: 5*E*,14*Z*-1-Deoxysphingadiene and 6*E*-3-keto-1-deoxySO (1350–1550 cm^{-1}). 6
Figure S3: 1-Deoxymethylsphingolipids (1350–1550 cm^{-1}). 6
Figure S4: 1-DeoxyPS, ω-OH and 4*E*-3-deoxySO (900–1800 cm^{-1}). 7
Comparison between 1-Deoxysphingolipids and 1-Deoxymethylsphingolipids **8**
Figure S5: IR spectra of 1-deoxysphingolipids and their 1-deoxymethylsphingolipid analogues. 8
Comparison between 4*E*-1-deoxySO and 4*E*-3-deoxySO **8**
Figure S6: IR spectra of the OH-regioisomers 4*E*-1-deoxySO and 4*E*-3-deoxySO 8
Calculated Spectra in the 3 Micron Range **9**
Figure S7: Calculated IR spectra in the region between 2500–3800 cm^{-1} 10
Energetics of all Conformers **11**
Table S3: List of distinct structures of 1-deoxySA 11
Table S4: List of distinct structures of 3-keto-1-deoxySA 11
Table S5: List of distinct structures of 4*E*-1-deoxySO 11
Table S6: List of distinct structures of 5*E*-1-deoxySO 12
Table S7: List of distinct structures of 8*E*-1-deoxySO 12
Table S8: List of distinct structures of 12*E*-1-deoxySO 13
Table S9: List of distinct structures of 13*E*-1-deoxySO 13
Table S10: List of distinct structures of 14*E*-1-deoxySO 13
Table S11: List of distinct structures of 14*Z*-1-deoxySO 14
Table S12: List of distinct structures of 1-deoxyPS 14
Table S13: List of distinct structures of ω-OH-1-deoxySA 14
Table S14: List of distinct structures of 4*E*-3-deoxySO 15
Table S15: List of distinct structures of 5*E*-14*Z*-1-deoxySD. 15
Table S16: List of distinct structures of 6*E*-3-keto-1-deoxySO 15
Table S17: List of distinct structures of 1-deoxymethylSA 16
Table S18: List of distinct structures of 13*Z*-1-deoxymethylSO 16
Table S19: Overview of energetics, interactions and interaction distances in selected conformers 17
Figure S8: Comparison of the experimental IR spectrum of 14*E*-1-deoxySO with the computed spectra of all optimized conformers 18
Coordinates of all Conformers **19**
Synthesis Data **245**
References **272**

Supplementary Structures

Table S1: Supplementary structures and *m/z* of protonated deoxysphingolipids.

Name	Structure
**4*E*-3-DeoxySO**<br>*m/z* = 284.3	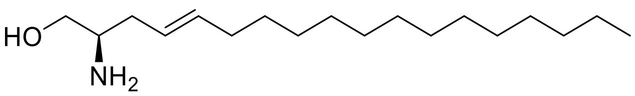
**5*E*,14*Z*-1-Deoxysphingadiene**<br>*m/z* = 282.3	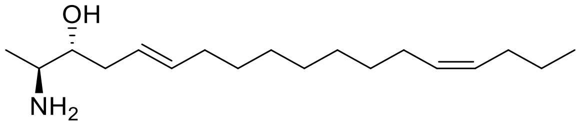
**6*E*-3-keto-1-deoxySO**<br>*m/z* = 282.3	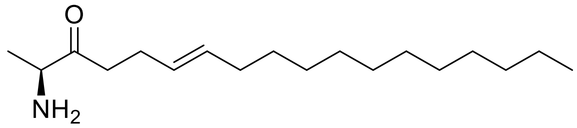
**1-DeoxymethylSA**<br>*m/z* = 272.3	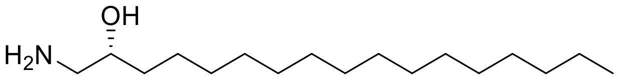
**13*Z*-1-DeoxymethylSO**<br>*m/z* = 270.3	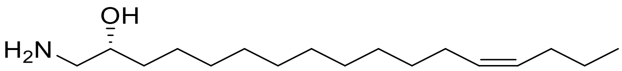

IMS Data: CCS

DT-IMS-MS measurements were performed on a modified Synapt G2-S HDMS instrument (Waters Corporation, Manchester, UK) containing a drift tube instead of the commercial travelling wave cell.[1] Ions were generated by nano electrospray ionization from a 10 µM solution of the lipid in methanol. Drift times were converted into collision cross sections (CCS) using the Mason-Schamp equation.[2]

Table S2: CCS of protonated deoxysphingolipids obtained from DT-IMS-MS measurements (Helium, 2.2 Torr). The values are given in Å^2 ± 1 Å^2. The CCS generally decreases with an increasing number of double bonds.

**Sample**	**$^{DT}CCS_{He}$ [M+H]$^+$**
1-DeoxySA	120
3-Keto-1-deoxySA	120
4*E*-1-DeoxySO	118
5*E*-1-DeoxySO	118
8*E*-1-DeoxySO	117
12*E*-1-DeoxySO	117
13*E*-1-DeoxySO	116
14*E*-1-DeoxySO	117
14*Z*-1-DeoxySO	115
1-DeoxyPS	125
ω-OH-1-DeoxySA	119
4*E*-3-DeoxySO	120
5*E*,14*Z*-1-Deoxysphingadiene	113
6*E*-3-keto-1-deoxySO	119
1-DeoxymethylSA	116
13*Z*-1-DeoxymethylSO	112

IR Spectra and Optimized Structures

IR spectra were measured using an experimental setup described previously.[3] Ions were generated by nano electrospray ionization from a 100 μM solution of the lipid in methanol (Sigma-Aldrich). After *m/z*-selection in a quadrupole mass filter, the ions are deflected into a hexapole ion trap filled with helium buffer gas. The walls of the ion trap were cooled with liquid nitrogen (~80 K) to allow for buffer gas cooling. After pumping out the buffer gas, the trapped ions are picked up from the trap by a pulsed beam of superfluid helium droplets, which are generated by expanding pressurized helium into the vacuum via a cold nozzle. The doped droplets leave the trap and coincide with the pulsed IR beam generated by the Fritz Haber Institute free-electron laser (FHI FEL).[4] In the case of resonant absorption of multiple photons, the ion is released from the droplet and detected on a time-of-flight detector. Spectra were recorded by scanning the spectral range of interest in steps of 2 cm^{-1}. The laser was more strongly focused in the regions from 900 to 1150 cm^{-1} and 1550–1800 cm^{-1} to increase the fluence in these wavenumber ranges.

The conformational space of the sphingolipids was sampled using the genetic algorithm FAFOOM.[5] Each generated structure was optimized at PBE+vdW^{TS}/light level of theory in FHI-aims.[6] The conformational search included rotation of all rotatable bonds. For each sample, a subset of the energetically most stable structures were re-optimized at PBE0-D3/6-311+G(d,p) level of theory followed by a frequency calculation in Gaussian 16.[7]

The following figures visualize the match between experimental and computed IR spectra. The displayed theoretical spectra were obtained from specific low-energy conformers (conf) numbered according to the labels in Tables S3 to S18. They were selected based on the match of the absorption frequencies in the region between 1400–1650 cm^{-1} with the experimental spectrum (example shown in Figure S8). ΔF is the harmonic free energy relative to the lowest-energy conformer (see Tables S3–S18). It is a useful value in this experiment, which is conducted at constant temperature and volume. The selected conformer is not always the lowest-energy conformer according to ΔF with the employed level of theory.

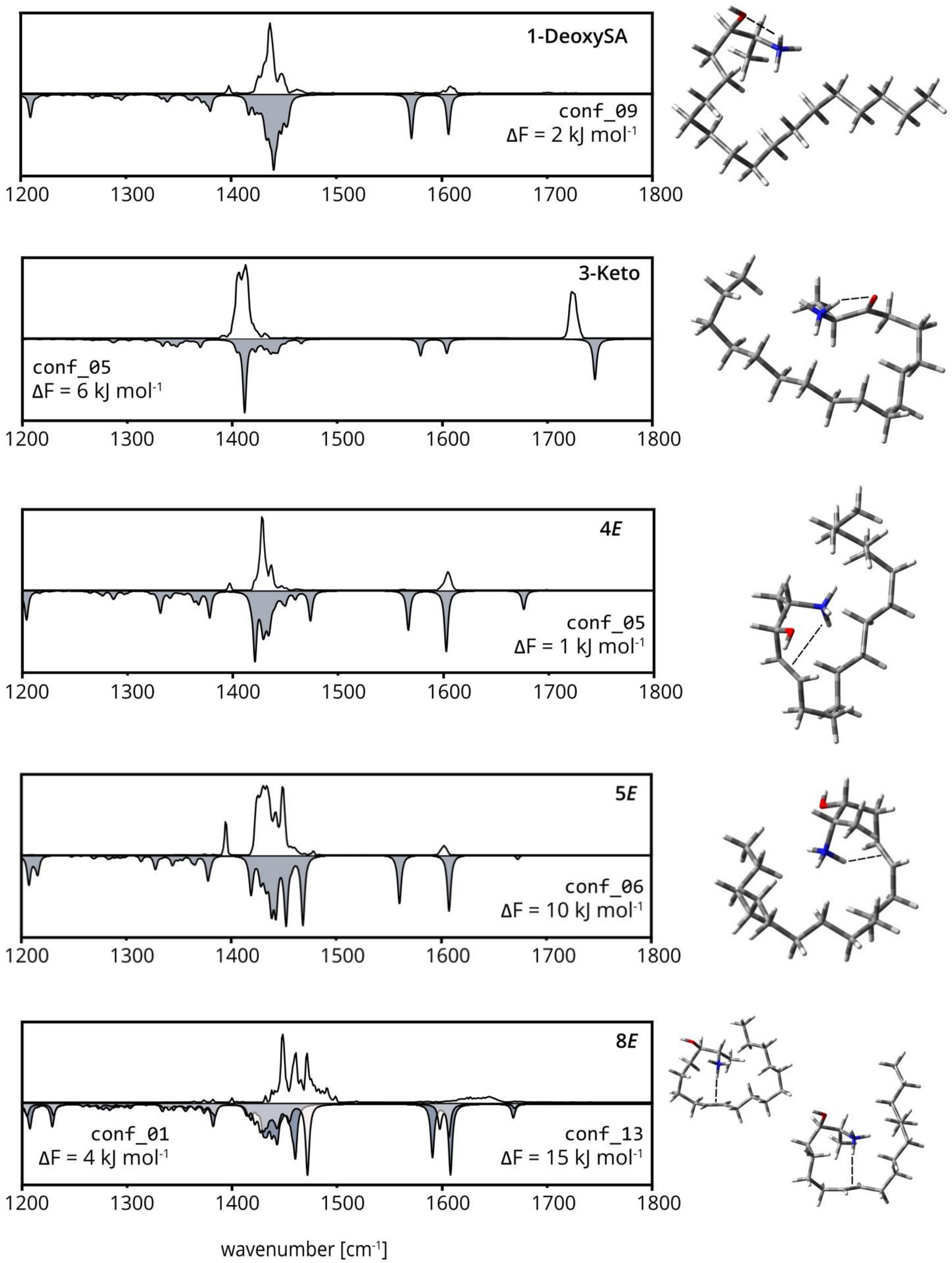
1-DeoxySA
conf_09
ΔF = 2 kJ mol-1
3-Keto
conf_05
ΔF = 6 kJ mol-1
4E
conf_05
ΔF = 1 kJ mol-1
5E
conf_06
ΔF = 10 kJ mol-1
8E
conf_01
ΔF = 4 kJ mol-1
conf_13
ΔF = 15 kJ mol-1
1200
1300
1400
1500
1600
1700
1800
wavenumber [cm-1]

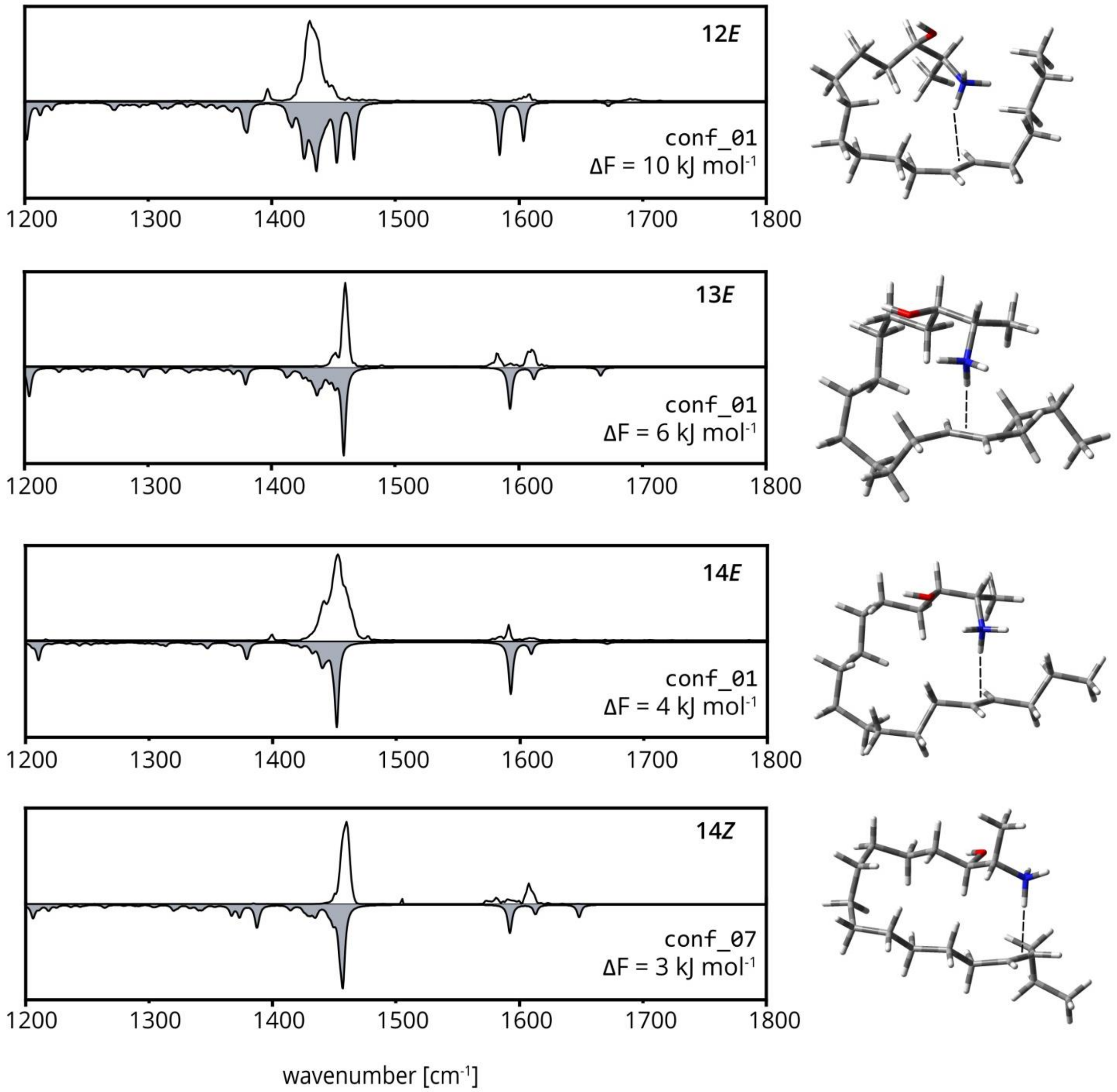

Figure S1: Experimental (transparent) and computed (grey) IR spectra and corresponding structures of saturated and monounsaturated 1-deoxysphingolipids (1200–1800 cm^{-1}).

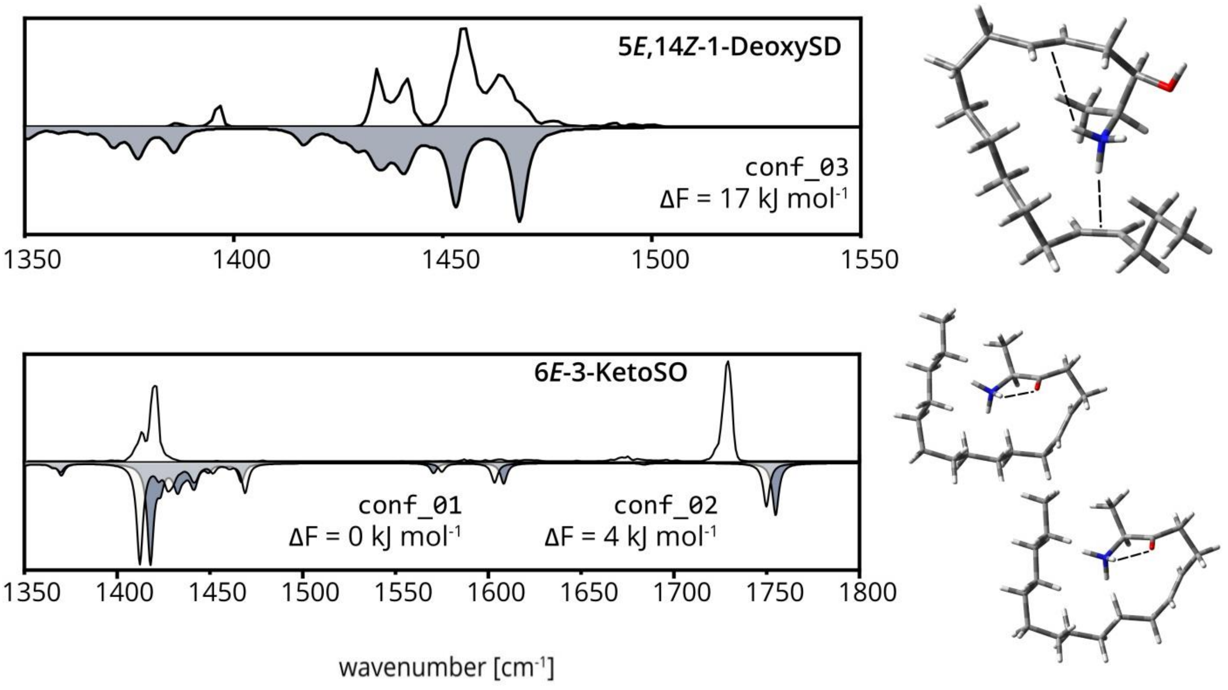

Figure S2: Experimental (transparent) and computed (grey) IR spectra and corresponding structures of the isomers 5*E*,14*Z*-1-deoxysphingadiene and 6*E*-3-keto-1-deoxySO (1350–1550 cm^{-1}).

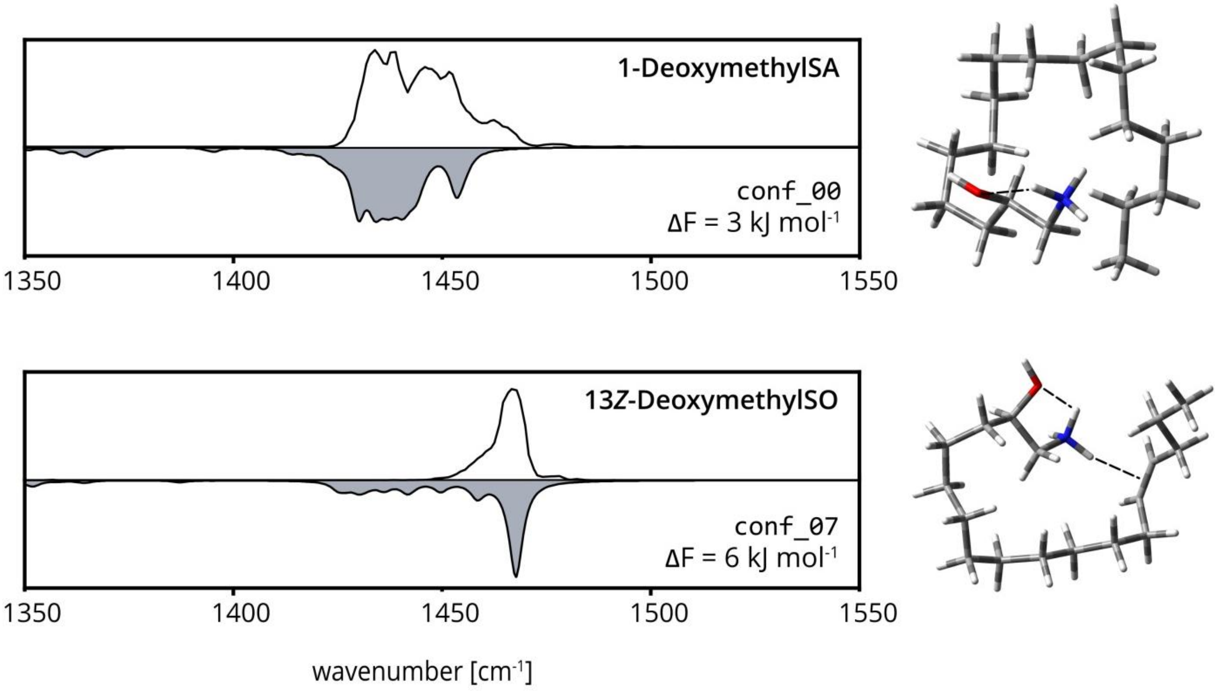

Figure S3: Experimental (transparent) and computed (grey) IR spectra and corresponding structures of 1-deoxymethylsphingolipids (1350–1550 cm^{-1}).

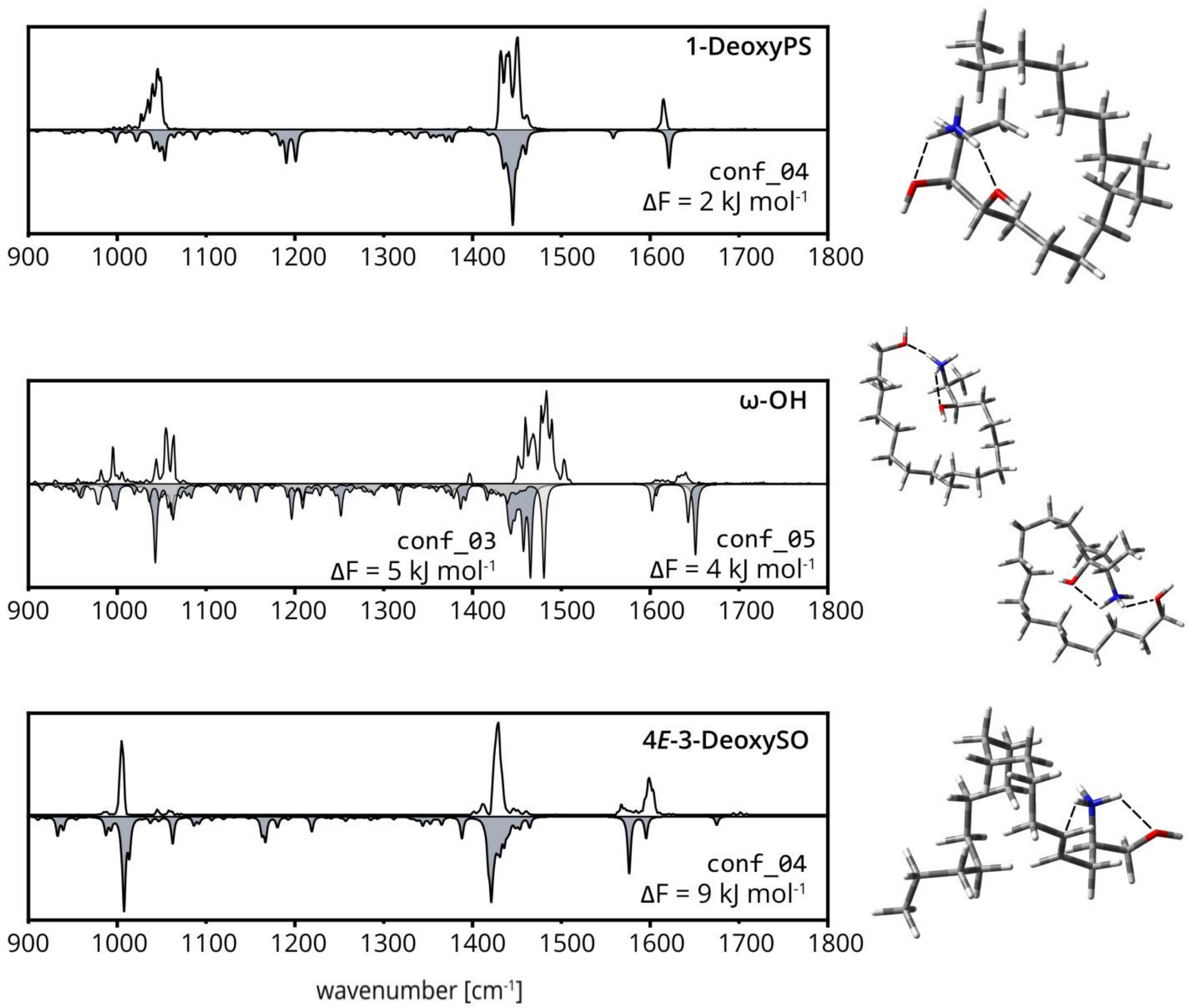

Figure S4: Experimental (transparent) and computed (grey) IR spectra and corresponding structures of the OH-regioisomers 1-deoxyPS and ω-OH and of 4*E*-3-deoxySO (900–1800 cm^{-1}).

Comparison between 1-Deoxysphingolipids and 1-Deoxymethylsphingolipids

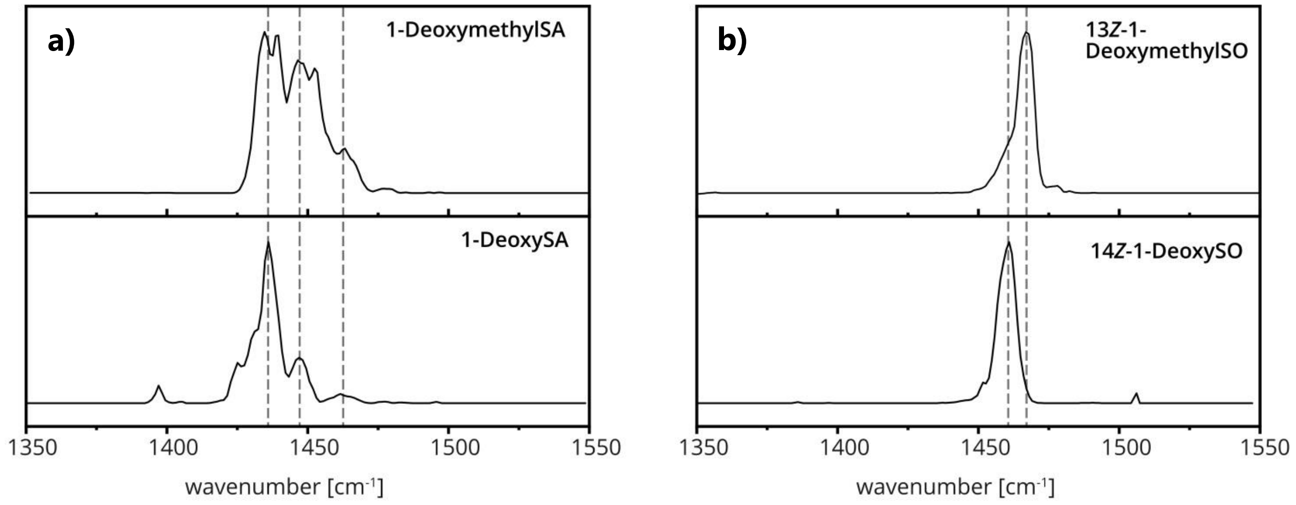

Figure S5: IR spectra of 1-deoxysphingolipids and their 1-deoxymethylsphingolipid analogues. a) The absorption bands of 1-deoxySA and 1-deoxymethylSA overlap largely. b) The main absorption band of 14*Z*-1-deoxySO is slightly shifted compared to 13*Z*-1-deoxymethylSO.

Comparison between 4E-1-deoxySO and 4E-3-deoxySO

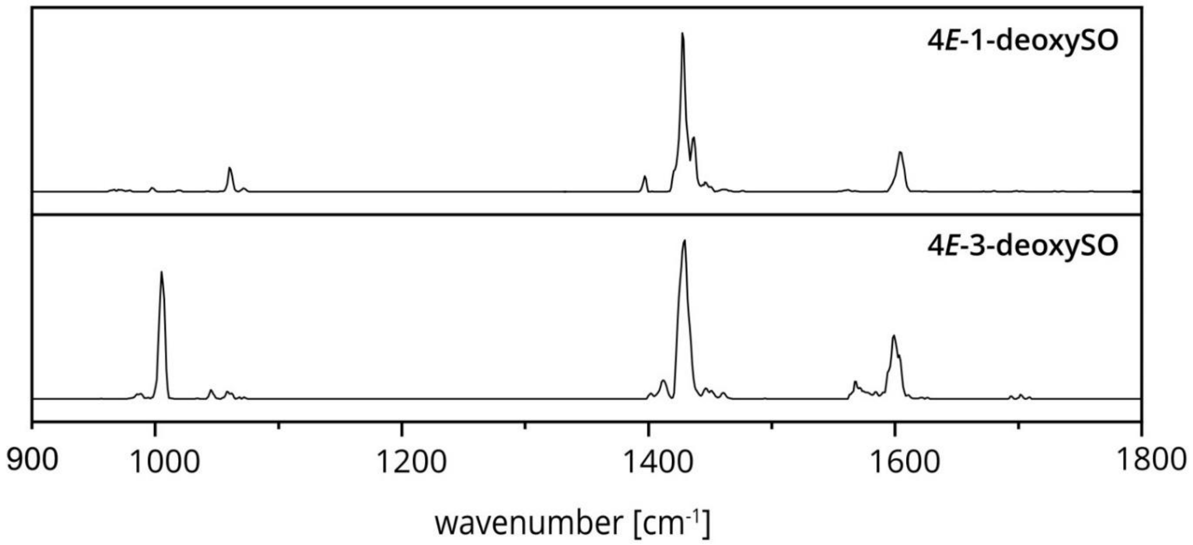

Figure S6: IR spectra of the OH-regioisomers 4*E*-1-deoxySO and 4*E*-3-deoxySO. The frequencies of N–H bending vibrations coincide, whereas differences occur in the low wavenumber range.

Calculated Spectra in the 3 Micron Range

The following calculated spectra illustrate the expected N–H stretching vibrations of the deoxysphingolipids in the 3 micron range. Experimental data are not available for this wavenumber range. For each structure, only the spectrum of the selected conformer according to the best match in the experimentally accessible region is shown (numbering according to Tables S3–S18).

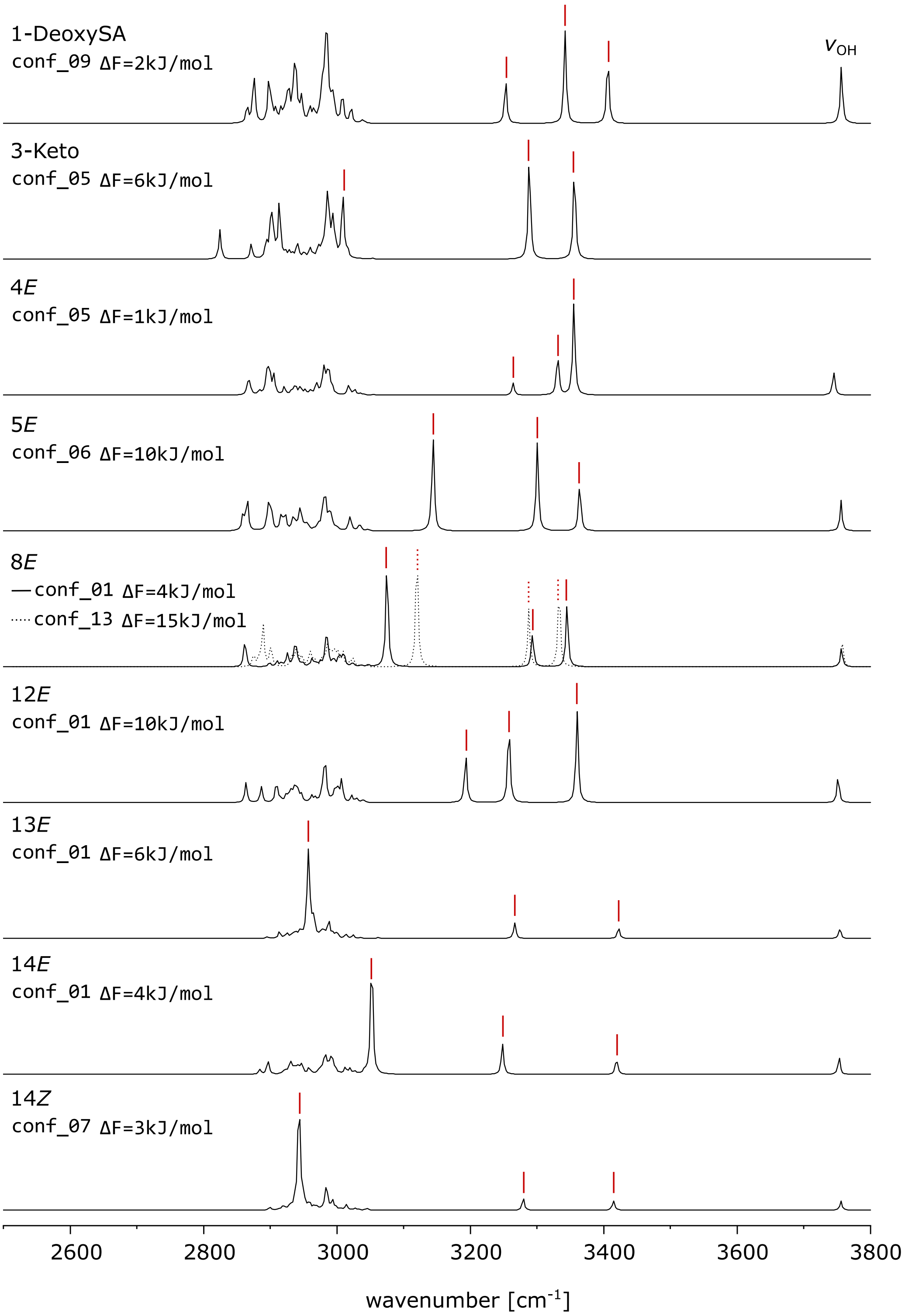

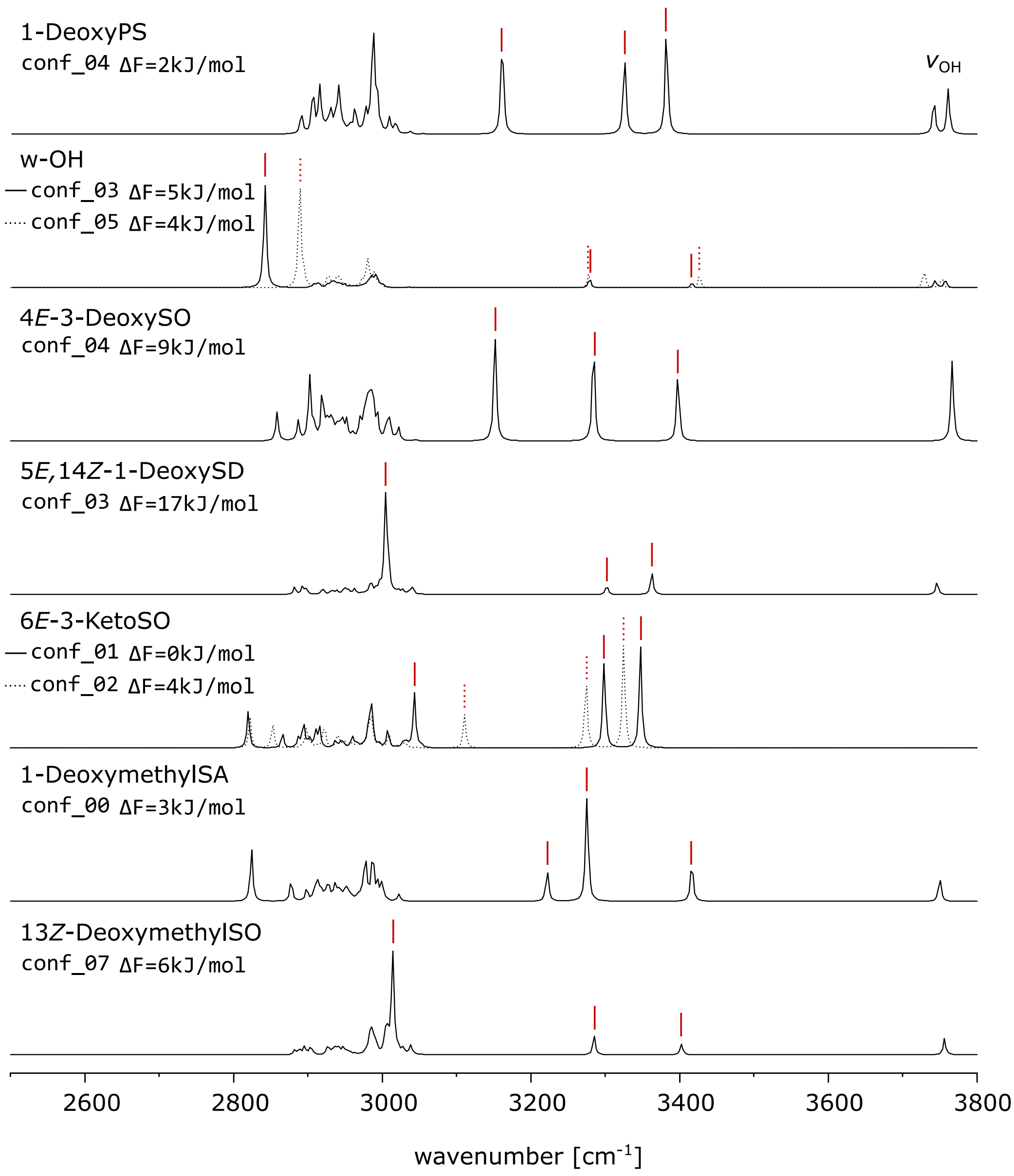

Figure S7: Calculated IR spectra of the selected conformers of all investigated deoxysphingolipids in the region between 2500–3800 cm^{-1}. The expected N–H stretching vibrations of the NH_3^+ group are indicated by red markers in each spectrum. According to the calculated spectra, the band patterns derived from the NH_3^+ vibrations in the 3 micron range are very diagnostic and allow for isomer distinction. O–H Stretching vibrations are located at higher wavenumbers (3700–3800 cm^{-1}).

Energetics of all Conformers

Table S3: List of distinct structures of **1-deoxySA** re-optimized at PBE0-D3/6-311+G(d,p) level of theory in Gaussian 16. Each conformer has a distinct label (conf_XX or ref_XX) and the final energy ΔE (including zero-point energy) and harmonic free energy ΔF at 78 K assigned to it. The energetics are relative to the lowest-energy conformer. All energies are displayed in kJ mol^{-1}.

ID	ΔE(PBE0-D3) [kJ mol^{-1}]	ΔF(PBE0-D3, 78 K) [kJ mol^{-1}]
conf_00	0	0
conf_01	1.42827	2.09797
conf_02	3.91725	5.18383
conf_03	4.35308	4.61134
conf_04	26.45454	25.87002
conf_05	1.73283	2.20333
conf_06	2.39971	2.92859
conf_07	4.36096	4.62471
conf_08	2.53098	1.37911
conf_09	2.33932	1.78365

Table S4: List of distinct structures of **3-keto-1-deoxySA** re-optimized at PBE0-D3/6-311+G(d,p) level of theory in Gaussian 16.

ID	ΔE(PBE0-D3) [kJ mol^{-1}]	ΔF(PBE0-D3, 78 K) [kJ mol^{-1}]
conf_00	0	0
conf_01	3.52342	4.50564
conf_02	5.0042	5.41151
conf_03	4.89393	5.89607
conf_04	15.59022	16.49001
conf_05	5.66583	6.00995
conf_06	5.95988	6.95996
conf_07	5.06459	5.64774

Table S5: List of distinct structures of **4*E*-1-deoxySO** re-optimized at PBE0-D3/6-311+G(d,p) level of theory in Gaussian 16. The reference conformers are structures provided by the authors of ref. [8] optimized by the same procedure.

ID	ΔE(PBE0-D3) [kJ mol^{-1}]	ΔF(PBE0-D3, 78 K) [kJ mol^{-1}]
conf_00	0.80865	1.64594
conf_01	9.54894	9.93221
conf_02	15.37755	15.16325
conf_03	22.05157	20.84131
conf_04	0.37019	0.23694
conf_05	0.57235	0.68157
conf_06	0	0
conf_07	2.67538	2.22961
conf_08	6.60575	5.3536
conf_09	8.04715	7.14667
ref_01[8]	4.57099	3.65677
ref_02[8]	-0.28093	0.32832

Table S6: List of distinct structures of **5*E*-1-deoxySO** re-optimized at PBE0-D3/6-311+G(d,p) level of theory in Gaussian 16. in kJ mol^{-1}.

ID	ΔE(PBE0-D3) [kJ mol^{-1}]	ΔF(PBE0-D3, 78 K) [kJ mol^{-1}]
conf_00	8.34384	8.74978
conf_01	10.33134	10.78124
conf_02	14.73693	13.96558
conf_03	20.82284	21.23634
conf_04	30.01472	30.37876
conf_05	29.07216	28.09201
conf_06	10.44161	10.42857
conf_07	14.07006	14.1724
conf_08	11.37104	11.51254
conf_09	12.21383	11.49674
conf_10	12.2217	11.51011
conf_11	13.43994	13.59998
conf_12	16.2571	16.77362
conf_13	14.76844	14.64755
conf_14	14.76581	14.78093
conf_15	15.83439	14.9868
ref_01[8]	0	0

Table S7: List of distinct structures of **8*E*-1-deoxySO** re-optimized at PBE0-D3/6-311+G(d,p) level of theory in Gaussian 16. The functional group interacting with the double bond and the interaction distances are specified for each conformer (OH or NH_3^+).

ID	ΔE(PBE0-D3) [kJ mol^{-1}]	ΔF(PBE0-D3, 78 K) [kJ mol^{-1}]	Interaction	Distance [Å]
conf_00	0	0	OH	2.17
conf_01	3.95925	3.55812	NH_3^+	2.27
conf_02	5.45054	5.7349	OH	2.12
conf_03	7.91063	6.73266	none	–
conf_04	26.19986	26.77477	NH_3^+	2.13
conf_05	3.46566	3.68134	OH	2.15
conf_06	4.21655	4.29829	OH	2.17
conf_07	6.87618	6.84253	OH	2.13
conf_08	6.88931	6.67707	OH	2.17
conf_09	6.0229	5.25223	OH	2.15
conf_10	6.4246	7.21518	OH	2.12
conf_11	5.69996	6.13063	OH	2.13
conf_12	9.30215	7.903	(NH_3^+)	(3.32)[1]
conf_13	15.44844	15.06242	NH_3^+	2.30
ref_01[8]	6.67402	5.90611	(NH_3^+)	(3.87)[1]
ref_02[8]	11.07173	11.33412	NH_3^+	2.27

[1] The interaction distance is too long for a specific interaction.

Table S8: List of distinct structures of **12*E*-1-deoxySO** re-optimized at PBE0-D3/6-311+G(d,p) level of theory in Gaussian 16.

ID	ΔE(PBE0-D3) [kJ mol^{-1}]	ΔF(PBE0-D3, 78 K) [kJ mol^{-1}]
conf_00	1.55692	0.66606
conf_01	9.66709	9.52011
conf_02	9.3494	8.03681
conf_03	10.28933	8.69924
conf_04	34.39142	33.39891
conf_05	10.28933	10.09976
conf_06	9.3494	8.04162
conf_07	11.14525	10.34299
conf_08	16.22296	17.22923
conf_09	13.09862	13.05054
conf_10	0	0
conf_11	2.13978	1.43437
conf_12	4.82304	3.19105
conf_13	10.7908	9.83744
conf_14	8.45673	7.30143
conf_15	12.03792	11.71166
ref_01[8]	20.75458	20.77038

Table S9: List of distinct structures of **13*E*-1-deoxySO** re-optimized at PBE0-D3/6-311+G(d,p) level of theory in Gaussian 16.

ID	ΔE(PBE0-D3) [kJ mol^{-1}]	ΔF(PBE0-D3, 78 K) [kJ mol^{-1}]
conf_00	3.57593	5.71552
conf_01	4.99895	6.15701
conf_02	2.77253	2.77116
conf_03	5.13023	6.59051
conf_04	29.34259	30.03426
conf_05	5.32451	7.05679
conf_06	5.47154	6.83291
conf_07	8.54338	10.25367
conf_08	9.04747	10.64375
conf_09	9.63821	10.73788
ref_01[8]	0	0

Table S10: List of distinct structures of **14*E*-1-deoxySO** re-optimized at PBE0-D3/6-311+G(d,p) level of theory in Gaussian 16.

ID	ΔE(PBE0-D3) [kJ mol^{-1}]	ΔF(PBE0-D3, 78 K) [kJ mol^{-1}]
conf_00	0.21529	0.64046
conf_01	4.41346	4.05904
conf_02	6.89981	7.7845
conf_03	6.74491	6.15283
conf_04	21.33744	21.55105
conf_05	7.18862	6.66179
conf_06	7.76098	7.17577
conf_07	9.13149	9.1885
conf_08	9.63296	9.54367
conf_09	10.90895	10.5037
ref_01[8]	0	0

Table S11: List of distinct structures of **14*Z*-1-deoxySO** re-optimized at PBE0-D3/6-311+G(d,p) level of theory in Gaussian 16.

ID	ΔE(PBE0-D3) [kJ mol^{-1}]	ΔF(PBE0-D3, 78 K) [kJ mol^{-1}]
conf_00	-0.0105	1.31171
conf_01	0.9058	2.52886
conf_02	0	0
conf_03	0.58811	1.75303
conf_04	3.21099	5.19328
conf_05	1.8326	1.99813
conf_06	23.52973	24.35946
conf_07	2.3682	2.63401
conf_08	0.1339	0.74521
conf_09	6.01765	7.90927
conf_10	8.31233	9.71147
conf_11	4.34783	5.96471
conf_12	0.18904	1.65274
conf_13	3.33964	4.30811
conf_14	9.04747	11.05861
conf_15	6.49549	7.67827
conf_16	7.96577	8.63202

Table S12: List of distinct structures of **1-deoxyPS** re-optimized at PBE0-D3/6-311+G(d,p) level of theory in Gaussian 16.

ID	ΔE(PBE0-D3) [kJ mol^{-1}]	ΔF(PBE0-D3, 78 K) [kJ mol^{-1}]
conf_00	0	0
conf_01	20.67319	21.59977
conf_02	11.25289	11.5819
conf_03	22.55305	23.12383
conf_04	0.85329	2.14459
conf_05	3.84636	5.6013
conf_06	3.61794	4.38448

Table S13: List of distinct structures of **ω-OH-1-deoxySA** re-optimized at PBE0-D3/6-311+G(d,p) level of theory in Gaussian 16.

ID	ΔE(PBE0-D3) [kJ mol^{-1}]	ΔF(PBE0-D3, 78 K) [kJ mol^{-1}]
conf_00	0	0
conf_01	14.35361	15.28225
conf_02	16.15995	15.51361
conf_03	4.04327	4.53919
conf_04	4.87818	5.08355
conf_05	3.98813	3.7182
conf_06	2.97207	2.69389
conf_07	6.72916	6.6536
conf_08	15.64273	16.02943
conf_09	16.97386	17.78024
conf_10	17.45432	17.36022
conf_11	4.91756	4.14278
conf_12	5.91525	5.2174
conf_13	5.73409	5.48819
conf_14	5.931	5.79707
conf_15	8.24932	7.16201

Table S14: List of distinct structures of ***4E*-3-deoxySO** re-optimized at PBE0-D3/6-311+G(d,p) level of theory in Gaussian 16.

ID	ΔE(PBE0-D3) [kJ mol^{-1}]	ΔF(PBE0-D3, 78 K) [kJ mol^{-1}]
conf_00	0	0
conf_01	5.27726	5.39746
conf_02	7.28839	6.85154
conf_03	7.29364	7.49352
conf_04	9.08948	9.30378
conf_05	7.03371	6.67586
conf_06	8.23094	7.52141
conf_07	8.76129	7.4693
conf_08	9.80624	7.87615
conf_09	7.29889	7.49877

Table S15: List of distinct structures of **5*E*-14*Z*-1-deoxySD** re-optimized at PBE0-D3/6-311+G(d,p) level of theory in Gaussian 16.

ID	ΔE(PBE0-D3) [kJ mol^{-1}]	ΔF(PBE0-D3, 78 K) [kJ mol^{-1}]
conf_00	0	0
conf_01	10.22317	10.14493
conf_02	14.69388	15.67686
conf_03	15.99553	17.1734
conf_04	14.69388	15.67686
conf_05	1.95042	2.2448
conf_06	2.26541	2.32619
conf_07	4.72431	5.16698
conf_08	4.55492	5.63695
conf_09	6.72107	7.76885
conf_10	5.60411	6.07803

Table S16: List of distinct structures of **6*E*-3-keto-1-deoxySO** re-optimized at PBE0-D3/6-311+G(d,p) level of theory in Gaussian 16.

ID	ΔE(PBE0-D3) [kJ mol^{-1}]	ΔF(PBE0-D3, 78 K) [kJ mol^{-1}]
conf_00	0	0
conf_01	0.61437	0.26269
conf_02	4.03014	3.97039
conf_03	4.26644	3.68741
conf_04	3.79647	3.65429
conf_05	5.96514	6.40404
conf_06	5.2405	5.29613
conf_07	10.2237	10.99505
conf_08	10.54926	10.90574
conf_09	32.09936	32.47989

Table S17: List of distinct structures of **1-deoxymethylSA** re-optimized at PBE0-D3/6-311+G(d,p) level of theory in Gaussian 16.

ID	ΔE(PBE0-D3) [kJ mol^{-1}]	ΔF(PBE0-D3, 78 K) [kJ mol^{-1}]
conf_00	1.75646	2.7634
conf_01	0	0
conf_02	2.45222	2.48518
conf_03	7.81611	8.13207
conf_04	6.36421	6.79625
conf_05	2.28944	1.68568
conf_06	4.55262	4.42692
conf_07	4.57362	4.06877
conf_08	5.25363	5.5421
conf_09	6.31695	6.29566

Table S18: List of distinct structures of **13*Z*-1-deoxymethylSO** re-optimized at PBE0-D3/6-311+G(d,p) level of theory in Gaussian 16.

ID	ΔE(PBE0-D3) [kJ mol^{-1}]	ΔF(PBE0-D3, 78 K) [kJ mol^{-1}]
conf_00	2.17391	2.64098
conf_01	2.58611	3.50239
conf_02	1.65406	2.20424
conf_03	3.15322	3.39912
conf_04	2.58349	3.49702
conf_05	0	0
conf_06	1.6068	1.28054
conf_07	6.04915	6.21812
conf_08	5.78922	5.95545
conf_09	4.92543	4.19186

Table S19: Overview of energetics, interactions and interaction distances in selected conformers. Energies are rounded to whole numbers.

Sample	Conformer	ΔF(PBE0-D3, 78 K) [kJ mol^{-1}]	Interaction	Distance [Å]
**1-DeoxySA**	conf_09	2	NH_3^+--OH	1.98
**3-Keto-1-deoxySA**	conf_05	6	NH_3^+--O=C	1.81
**4*E*-1-DeoxySO**	conf_05	1	NH_3^+--C=C	2.69
			NH_3^+--OH	2.05
**5*E*-1-DeoxySO**	conf_06	10	NH_3^+--C=C	2.14
			NH_3^+--OH	2.21
**8*E*-1-DeoxySO**	conf_01	4	NH_3^+--C=C	2.27
			NH_3^+--OH	2.11
	conf_13	15	NH_3^+--C=C	2.30
			NH_3^+--OH	2.07
**12*E*-1-DeoxySO**	conf_01	10	NH_3^+--C=C	2.39
			NH_3^+--OH	1.96
**13*E*-1-DeoxySO**	conf_01	6	NH_3^+--C=C	2.09
			NH_3^+--OH	1.99
**14*E*-1-DeoxySO**	conf_01	4	NH_3^+--C=C	2.16
			NH_3^+--OH	1.94
**14*Z*-1-DeoxySO**	conf_07	3	NH_3^+--C=C	2.06
			NH_3^+--OH	2.04
**1-DeoxyPS**	conf_04	2	NH_3^+--2 OH	2.11, 1.79
**ω-OH-1-DeoxySA**	conf_03	5	NH_3^+--2 OH	1.98, 1.66[2]
	conf_05	4	NH_3^+--2 OH	2.01, 1.69[2]
**4*E*-3-DeoxySO**	conf_04	9	NH_3^+--C=C	2.23
			NH_3^+--OH	2.02
**5*E*,14*Z*-1-Deoxysphingadiene**	conf_03	17	NH_3^+--C=C	2.12 (14*Z*)
			NH_3^+--C=C	2.89 (5*E*)
			NH_3^+--OH	2.30
**6*E*-3-keto-1-deoxySO**	conf_01	0	NH_3^+--O=C	1.82
	conf_02	4	NH_3^+--O=C	1.89
**1-DeoxymethylSA**	conf_00	3	NH_3^+--OH	2.03
**13*Z*-1-DeoxymethylSO**	conf_07	6	NH_3^+--C=C	2.16
			NH_3^+--OH	2.05

[2] The shorter distance refers to the interaction between the amine and the terminal ω-OH group.

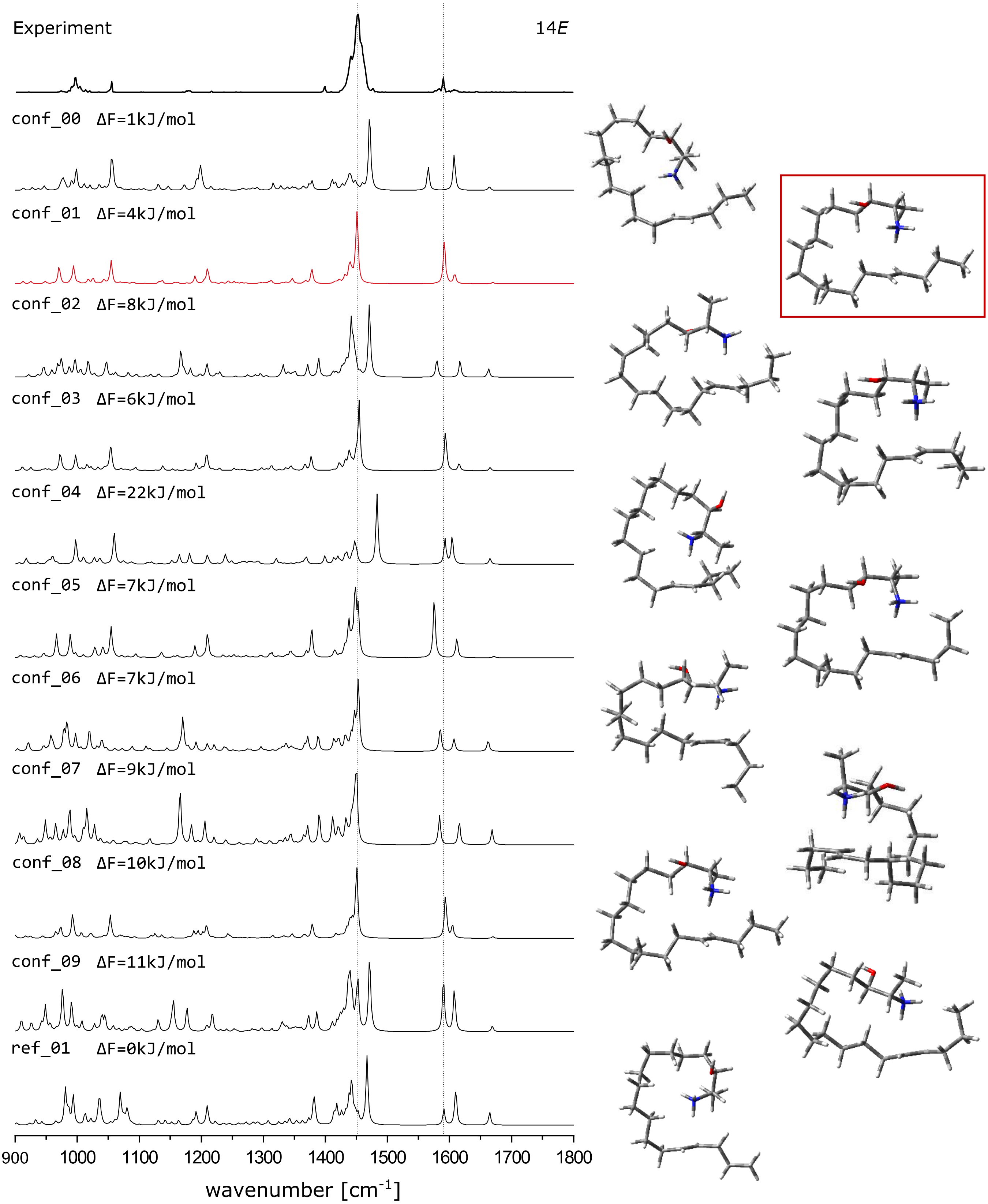

Figure S8: Comparison of the experimental IR spectrum of **14*E*-1-deoxySO** with the computed spectra of all optimized conformers (Table S10). The best match in the region 1400–1650 cm^{-1} was found for conf_01 (red).

Coordinates of all Conformers

Cartesian coordinates of all re-optimized conformers are given below. All geometries are obtained from optimization at PBE0-D3/6-311+G(d,p) level of theory in Gaussian 16 using default settings.

1-DeoxySA

```
conf_00

1 1
C -1.713018 -2.22972 -2.483398
C -1.105308 -1.602758 -1.247185
H -2.198165 -1.458317 -3.082427
H -2.463849 -2.978757 -2.221131
H -0.946078 -2.690201 -3.112975
C -3.024431 0.006583 -0.848974
H -3.703819 -0.493149 -1.54717
H -2.423272 0.707562 -1.439361
C -2.079388 2.632868 0.637954
C -0.662517 2.127099 0.385982
H -2.014305 3.477423 1.332399
H -2.495921 3.047159 -0.289873
C 0.297465 3.19666 -0.119453
H -0.678989 1.300605 -0.333668
H -0.264726 1.709732 1.322023
C 1.690606 2.64996 -0.423766
H 0.37029 3.988811 0.634064
H -0.117349 3.669883 -1.018996
C 1.759687 1.817703 -1.700806
H 2.026063 2.055808 0.436429
H 2.402171 3.478784 -0.510693
H 1.518123 2.462659 -2.553664
H 0.972936 1.050894 -1.688968
C 2.644838 -1.005347 -0.665998
C 3.014547 -1.871871 0.539559
H 2.649604 -1.613132 -1.581564
H 1.620826 -0.624747 -0.549709
C 2.685599 -1.240121 1.891494
H 4.084957 -2.098948 0.493519
H 2.516865 -2.853134 0.478253
C 1.191913 -1.117589 2.170244
H 3.142821 -0.246468 1.959414
H 3.143575 -1.834228 2.689436
C 0.871319 -0.416899 3.482479
H 0.750974 -2.128954 2.196547
H 0.709099 -0.555259 1.356475
H 1.318567 -0.946272 4.328052
H -0.206092 -0.356652 3.658733
H 1.268164 0.602412 3.482658
N -0.345183 -2.66298 -0.486712
H 0.227348 -2.249706 0.259584
H 0.265421 -3.210759 -1.092725
H -0.375872 -0.833924 -1.51246
```

H -1.045411 -3.272095 -0.04421
C -2.103176 -1.034878 -0.241506
H -1.525389 -0.571821 0.571102
O -2.782309 -2.177991 0.264812
H -3.462934 -1.910953 0.887458
C 3.576231 0.185447 -0.871189
H 3.698375 0.723114 0.074793
H 4.572359 -0.191403 -1.128609
C 3.114625 1.157715 -1.955309
H 3.081009 0.634428 -2.919097
H 3.873677 1.940362 -2.065937
C -3.043446 1.590078 1.215592
H -2.490507 0.923434 1.892145
H -3.776471 2.093575 1.85309
C -3.838037 0.781475 0.187369
H -4.515199 0.096009 0.715622
H -4.499179 1.462571 -0.358589

conf_01

1 1
C 3.371354 -2.32343 0.723871
C 2.60793 -2.256767 -0.577165
H 3.29348 -3.332433 1.134808
H 2.979455 -1.626175 1.468222
H 4.433147 -2.112607 0.574433
C 0.325933 -1.923451 0.558151
H 0.432721 -0.836319 0.436265
H 0.743094 -2.172667 1.539701
C -3.461218 -1.521108 1.432473
C -4.08494 -1.367406 0.04183
H -3.906635 -0.79307 2.118642
H -3.729955 -2.506209 1.831179
C -3.627366 -0.142206 -0.745942
H -5.174735 -1.342933 0.153593
H -3.875448 -2.264358 -0.554293
C -3.928157 1.19901 -0.088101
H -4.097832 -0.163641 -1.736753
H -2.548505 -0.218688 -0.932898
C -3.424083 2.398259 -0.893258
H -3.505128 1.225067 0.923171
H -5.01177 1.29571 0.046699
H -3.720213 3.322192 -0.382497
H -3.934778 2.412359 -1.863419
C 0.392612 2.24188 -0.074182
C 1.23668 2.480782 1.17271
H 0.784983 2.821909 -0.918373
H 0.463678 1.182331 -0.377235
C 2.617529 1.83617 1.142007
H 0.695773 2.105189 2.049433
H 1.346509 3.560352 1.328963
C 3.493638 2.235956 -0.040322
H 2.496947 0.736758 1.177922

H 3.15117 2.071867 2.069915
C 4.889692 1.630457 0.017682
H 3.574926 3.328282 -0.063748
H 3.002115 1.977597 -0.989899
H 5.431431 1.982103 0.899774
H 5.485585 1.893802 -0.859865
H 4.865713 0.53449 0.095445
N 2.657246 -0.865523 -1.163724
H 1.962122 -0.845417 -1.924757
H 3.581344 -0.627784 -1.523593
H 3.076506 -2.889731 -1.335428
H 2.404306 -0.148765 -0.474154
C 1.127382 -2.641708 -0.517026
H 1.089185 -3.726176 -0.349707
O 0.672577 -2.33342 -1.829307
H -0.263984 -2.530153 -1.913461
C -1.080094 2.568832 0.131238
H -1.470249 1.915368 0.920711
H -1.176774 3.59075 0.518373
C -1.914792 2.422744 -1.135873
H -1.613748 1.508786 -1.66476
H -1.676058 3.24697 -1.81786
C -1.945696 -1.344779 1.482774
H -1.695586 -0.305872 1.23347
H -1.599193 -1.498611 2.511621
C -1.161947 -2.258795 0.547688
H -1.312198 -3.30957 0.823978
H -1.56949 -2.14132 -0.464435

conf_02

1 1
C 0.750184 3.815439 -1.216167
C 1.372449 2.886251 -0.194556
H -0.087444 4.347567 -0.764318
H 0.37304 3.260096 -2.078337
H 1.465946 4.569735 -1.555945
C -0.896375 2.169781 0.726028
H -1.466172 2.499596 -0.148416
H -0.793145 3.037179 1.390324
C -3.93813 1.444629 0.372533
C -3.664352 0.359291 -0.667256
H -3.756612 2.423676 -0.087229
H -5.00123 1.441635 0.637744
C -3.921291 -1.067065 -0.184542
H -2.628058 0.454186 -1.016404
H -4.280865 0.5521 -1.552413
C -3.290036 -2.135915 -1.077357
H -5.002474 -1.232488 -0.125815
H -3.554149 -1.191191 0.840726
C -1.764919 -2.20237 -0.980376
H -3.579668 -1.950978 -2.118346
H -3.700006 -3.118922 -0.818116

H -1.363064 -1.188384 -1.135377
H -1.371511 -2.792704 -1.814152
C 1.204644 -2.946135 -0.39427
C 2.662924 -2.749797 0.011663
H 1.022944 -2.450151 -1.35719
H 1.028694 -4.013376 -0.572376
C 3.113902 -1.294496 0.074979
H 3.310501 -3.28345 -0.693192
H 2.82951 -3.217447 0.990542
C 4.549543 -1.129496 0.555506
H 2.449118 -0.730021 0.744778
H 3.004657 -0.852567 -0.928125
C 5.013568 0.318944 0.594656
H 5.220147 -1.705743 -0.09139
H 4.643802 -1.565539 1.55604
H 5.033056 0.756324 -0.413101
H 6.026943 0.416994 0.990616
H 4.369588 0.922652 1.253465
N 2.575655 2.222553 -0.823076
H 3.198592 1.780984 -0.138102
H 3.123362 2.867627 -1.391942
H 1.728386 3.439127 0.677574
H 2.189282 1.464297 -1.409847
C 0.475081 1.727123 0.249032
H 0.98783 1.19168 1.064701
O 0.415485 0.888546 -0.898364
H 0.007995 0.043499 -0.679108
C 0.205983 -2.462017 0.651423
H 0.466193 -2.908558 1.619106
H 0.313078 -1.376494 0.804725
C -1.254559 -2.77393 0.34067
H -1.867236 -2.399455 1.168368
H -1.399087 -3.861061 0.333647
C -3.1229 1.317479 1.664663
H -3.254889 2.227043 2.262427
H -3.52684 0.503749 2.2752
C -1.634646 1.05194 1.456594
H -1.154553 0.881837 2.427287
H -1.525541 0.112766 0.901059

conf_03

1 1
C 1.264017 -0.994915 2.342897
C 2.194196 -1.658828 1.355184
H 1.023334 -1.701161 3.140522
H 0.327017 -0.681245 1.875824
H 1.731449 -0.123595 2.80804
C 0.235115 -2.94833 0.22305
H -0.4289 -2.853033 1.088493
H 0.038662 -3.951873 -0.177071
C -2.646521 -2.441671 -0.710157
C -3.109218 -1.189058 0.030118

```
H -2.47044 -3.248523 0.011288
H -3.468275 -2.793617 -1.344045
C -3.596332 -0.074228 -0.891136
H -2.312671 -0.815641 0.688599
H -3.930182 -1.472062 0.700012
C -4.413312 1.004365 -0.181773
H -4.231761 -0.520609 -1.666564
H -2.755537 0.378634 -1.429999
C -3.73569 1.773534 0.952651
H -5.316115 0.529991 0.222632
H -4.765276 1.726801 -0.92818
H -3.3916 1.080343 1.731685
H -4.508898 2.384179 1.430162
C -0.09746 3.005877 0.113115
C 1.253051 2.318576 -0.018319
H -0.039754 3.772904 0.895076
H -0.326911 3.53626 -0.818956
C 2.428184 3.258757 -0.252128
H 1.183597 1.595573 -0.847621
H 1.43668 1.750011 0.907821
C 3.775912 2.5522 -0.375522
H 2.470411 3.974546 0.575962
H 2.247446 3.85034 -1.157747
C 3.950315 1.751398 -1.661237
H 3.932008 1.916121 0.514415
H 4.579381 3.292746 -0.318076
H 3.153601 1.014942 -1.821557
H 4.90893 1.225384 -1.685583
H 3.92463 2.415949 -2.528999
N 2.545915 -0.726743 0.226147
H 2.999119 -1.296306 -0.50186
H 3.166785 0.033174 0.511
H 3.153733 -1.891462 1.824858
H 1.710258 -0.294869 -0.177348
C 1.67479 -2.954739 0.722968
H 1.764944 -3.721282 1.503226
O 2.614194 -3.209206 -0.315218
H 2.437355 -4.054661 -0.735032
C -1.2191 2.029543 0.437885
H -1.24145 1.242825 -0.326165
H -0.995978 1.529395 1.3921
C -2.585473 2.696209 0.544638
H -2.828796 3.182119 -0.409387
H -2.504058 3.506331 1.279173
C -1.413022 -2.264669 -1.592973
H -1.240415 -3.193637 -2.149668
H -1.602571 -1.493652 -2.346931
C -0.126316 -1.914775 -0.843148
H 0.682398 -1.845163 -1.582025
H -0.245932 -0.920808 -0.386044
```

conf_04

1 1
C -2.466019 3.361225 0.211049
C -1.360362 2.359587 -0.033863
H -2.332872 4.195761 -0.477388
H -2.426523 3.758416 1.231005
H -3.454532 2.927843 0.036112
C 1.142508 1.885943 0.044263
H 0.953909 1.233615 -0.817459
H 1.104968 1.261284 0.949855
C 3.617292 0.296722 -0.857233
C 4.540341 -0.88134 -0.555543
H 3.874891 0.712158 -1.838976
H 2.595719 -0.087196 -0.958726
C 4.274448 -1.588878 0.778688
H 5.584058 -0.54769 -0.573127
H 4.447796 -1.60093 -1.376471
C 2.802978 -1.847694 1.093896
H 4.699467 -0.999578 1.599346
H 4.824087 -2.536741 0.784979
C 2.030039 -2.646491 0.051314
H 2.295513 -0.881756 1.229598
H 2.730442 -2.353398 2.064048
H 2.381574 -3.684887 0.034507
H 2.225224 -2.247793 -0.951398
C -1.809609 -3.209167 -0.558131
C -2.375366 -1.836716 -0.210408
H -2.043518 -3.905695 0.256224
H -2.3256 -3.592845 -1.445316
C -3.894995 -1.760393 -0.219752
H -1.973037 -1.097731 -0.924656
H -2.0147 -1.55812 0.791826
C -4.431181 -0.393333 0.186739
H -4.304728 -2.522357 0.453685
H -4.265121 -2.006849 -1.221705
C -5.948508 -0.285641 0.139979
H -4.002727 0.372252 -0.482529
H -4.10104 -0.172261 1.217889
H -6.317694 -0.474846 -0.871474
H -6.295864 0.705054 0.443424
H -6.409446 -1.020349 0.805434
N -1.528145 1.201271 0.91465
H -0.85319 0.454005 0.7282
H -2.466278 0.79054 0.818391
H -1.441163 1.926296 -1.035508
H -1.411854 1.501252 1.885109
C 0.047982 2.944604 0.108221
H 0.111711 3.476634 1.074516
O 0.108875 3.859513 -0.957018
H 0.94529 4.330717 -0.928338
C -0.305951 -3.203534 -0.821179
H 0.026279 -4.229306 -1.014872
H -0.103404 -2.642951 -1.744074
C 0.529 -2.612548 0.306147
H 0.296955 -3.119373 1.252581

H 0.244256 -1.556944 0.455267
C 3.641023 1.417144 0.176479
H 3.527197 1.003593 1.186014
H 4.619969 1.908049 0.162285
C 2.55333 2.467762 -0.044459
H 2.664197 3.261406 0.706193
H 2.69411 2.932223 -1.028121

conf_05

1 1
C -0.687287 -2.518805 -1.094379
C -0.962133 -1.477708 -0.030592
H -1.395358 -2.394526 -1.914589
H -0.798129 -3.530915 -0.698526
H 0.315329 -2.402902 -1.513264
C -3.497166 -1.661158 -0.242007
H -4.396292 -1.7938 0.372909
H -3.424124 -2.550036 -0.876693
C -3.419806 2.149046 -0.827582
C -1.902269 2.25424 -0.671175
H -3.88505 3.000373 -0.31794
H -3.678629 2.254741 -1.887511
C -1.426863 2.295659 0.777924
H -1.560806 3.156328 -1.188181
H -1.409971 1.424444 -1.200928
C 0.085134 2.191658 0.953126
H -1.904214 1.490545 1.352214
H -1.777718 3.225855 1.241485
C 0.882439 3.293901 0.263934
H 0.431817 1.220451 0.56154
H 0.322498 2.197467 2.025762
H 0.418465 4.260488 0.493746
H 0.807578 3.177577 -0.822917
C 3.158634 1.470598 -0.871099
C 3.893069 0.137484 -0.983569
H 3.648125 2.207157 -1.518168
H 2.142022 1.352896 -1.272377
C 3.143172 -1.03015 -0.352484
H 4.881386 0.221089 -0.515096
H 4.073447 -0.096177 -2.038753
C 3.864224 -2.368284 -0.435577
H 2.168042 -1.110771 -0.865924
H 2.9501 -0.800223 0.707738
C 3.092409 -3.500897 0.22601
H 4.84733 -2.270721 0.037341
H 4.054392 -2.612834 -1.486438
H 2.9178 -3.295336 1.289965
H 3.635679 -4.446936 0.173735
H 2.12414 -3.668982 -0.26261
N 0.091107 -1.584766 1.04528
H 0.057848 -0.777538 1.671699
H 1.03737 -1.638302 0.651632

H -0.875334 -0.467627 -0.433719
H -0.114439 -2.428797 1.594378
C -2.29951 -1.634253 0.694319
H -2.402412 -0.786185 1.388421
O -2.145299 -2.833087 1.44311
H -2.970634 -3.069285 1.873496
C 3.107153 2.02514 0.548318
H 2.664678 1.286018 1.22906
H 4.13349 2.169681 0.905493
C 2.350538 3.34401 0.68512
H 2.860664 4.116089 0.097955
H 2.407842 3.671303 1.730163
C -4.031879 0.856455 -0.289431
H -3.754897 0.739637 0.764441
H -5.1219 0.951693 -0.283785
C -3.646827 -0.396896 -1.088415
H -4.403633 -0.58502 -1.854844
H -2.720048 -0.219109 -1.647387

conf_06

1 1
C -0.197913 -3.108169 0.020725
C -0.516646 -1.65431 0.301205
H -0.921378 -3.510089 -0.689282
H -0.244791 -3.70757 0.932858
H 0.790668 -3.214845 -0.431975
C -3.036239 -2.043958 0.436528
H -3.869063 -1.953276 1.1452
H -2.862914 -3.116473 0.308915
C -3.582755 1.077339 -1.640004
C -2.181843 1.567728 -1.277082
H -4.2682 1.932278 -1.632222
H -3.58675 0.697726 -2.668196
C -2.144363 2.417938 -0.009709
H -1.767987 2.138892 -2.113804
H -1.510141 0.70344 -1.15924
C -0.756871 2.588821 0.596066
H -2.805861 1.986834 0.751288
H -2.561695 3.407168 -0.232851
C 0.27106 3.228494 -0.327339
H -0.39666 1.596981 0.921934
H -0.832711 3.180766 1.51619
H -0.162365 4.146305 -0.741889
H 0.463591 2.577686 -1.188798
C 2.811353 1.371509 -0.056959
C 3.811609 0.373201 0.518888
H 3.283365 1.886984 -0.902775
H 1.943362 0.856016 -0.496046
C 4.297673 -0.685223 -0.46677
H 3.394261 -0.117093 1.41496
H 4.673084 0.937106 0.892124
C 3.266527 -1.75759 -0.794611

H 5.189768 -1.176448 -0.063451
H 4.614863 -0.19562 -1.395587
C 3.750342 -2.777706 -1.814989
H 2.347 -1.287096 -1.175348
H 3.017303 -2.294227 0.138391
H 3.986113 -2.290871 -2.765158
H 2.999246 -3.547371 -2.012942
H 4.656635 -3.278608 -1.464322
N 0.579747 -1.07374 1.158967
H 0.522522 -0.051313 1.202605
H 1.508859 -1.322081 0.802956
H -0.519266 -1.070215 -0.620592
H 0.448167 -1.44061 2.109923
C -1.813295 -1.413823 1.080696
H -1.966189 -0.325589 1.151437
O -1.527446 -1.934735 2.373504
H -2.311139 -1.905453 2.927455
C 2.35516 2.409135 0.962969
H 1.763456 1.928478 1.757779
H 3.24083 2.802794 1.47456
C 1.582412 3.584024 0.36837
H 2.23402 4.101005 -0.345467
H 1.374348 4.307051 1.165723
C -4.11589 -0.007979 -0.70644
H -4.01543 0.328017 0.333194
H -5.192225 -0.121094 -0.863242
C -3.437647 -1.378949 -0.881185
H -4.11144 -2.054542 -1.414046
H -2.559961 -1.283886 -1.533207

conf_07

1 1
C 1.264476 -0.994494 2.342876
C 2.194643 -1.658333 1.355095
H 1.024135 -1.700624 3.140701
H 0.32732 -0.681118 1.875909
H 1.731817 -0.12296 2.807724
C 0.235606 -2.94818 0.223227
H -0.428249 -2.853002 1.088808
H 0.039299 -3.951768 -0.176852
C -2.64625 -2.441979 -0.709892
C -3.109121 -1.189338 0.030206
H -2.469996 -3.248696 0.011666
H -3.467949 -2.794156 -1.343725
C -3.596621 -0.074784 -0.891172
H -2.312552 -0.815645 0.68851
H -3.929941 -1.472378 0.700269
C -4.413541 1.003854 -0.181816
H -4.232222 -0.521412 -1.666325
H -2.756048 0.378068 -1.430389
C -3.735918 1.772985 0.952621
H -5.316356 0.529529 0.222622

H -4.765499 1.726291 -0.928232
H -3.391745 1.079769 1.731609
H -4.509176 2.38352 1.430206
C -0.097889 3.005755 0.113082
C 1.252639 2.318515 -0.018428
H -0.040232 3.77262 0.895211
H -0.327287 3.536333 -0.818897
C 2.427683 3.258786 -0.252297
H 1.183154 1.59549 -0.847714
H 1.43635 1.749963 0.907708
C 3.775482 2.552319 -0.375479
H 2.46977 3.974694 0.5757
H 2.246938 3.850217 -1.158017
C 3.950008 1.751431 -1.661129
H 3.931533 1.916314 0.514519
H 4.57888 3.292936 -0.31804
H 3.153189 1.0151 -1.821555
H 4.908557 1.225282 -1.6853
H 3.92457 2.415956 -2.528922
N 2.546139 -0.726223 0.226091
H 2.999315 -1.29561 -0.50205
H 3.166934 0.033814 0.51085
H 3.154245 -1.890894 1.824683
H 1.710366 -0.29445 -0.177312
C 1.675412 -2.954305 0.722845
H 1.765835 -3.720874 1.503065
O 2.614663 -3.208416 -0.315495
H 2.438397 -4.05419 -0.734919
C -1.219469 2.029281 0.437558
H -1.241854 1.242866 -0.326805
H -0.996261 1.52875 1.391559
C -2.585846 2.695847 0.544718
H -2.829345 3.182093 -0.409096
H -2.504398 3.505702 1.279551
C -1.412776 -2.264878 -1.592732
H -1.239995 -3.193898 -2.14929
H -1.60244 -1.494001 -2.346808
C -0.126157 -1.91467 -0.842914
H 0.682533 -1.844847 -1.581799
H -0.246023 -0.920764 -0.385725

conf_08

1 1
C -0.78512 1.163014 2.016159
C -1.385007 2.052962 0.953363
H -0.502961 1.770445 2.878849
H 0.110035 0.646453 1.660274
H -1.504647 0.416926 2.361892
C 0.950332 2.874301 0.158628
H 1.445907 2.583501 1.090793
H 1.436701 3.811148 -0.143551
C 3.648459 1.481183 -0.268442

C 3.405169 0.187433 0.504349
H 3.669048 2.323264 0.433433
H 4.65086 1.444601 -0.709353
C 3.509389 -1.076794 -0.343213
H 2.430528 0.223485 1.011731
H 4.145538 0.126223 1.310591
C 3.552137 -2.357753 0.485894
H 4.429392 -1.015843 -0.937477
H 2.690234 -1.126494 -1.072611
C 2.253445 -2.75313 1.188985
H 4.344593 -2.251913 1.23676
H 3.859163 -3.191594 -0.156204
H 1.87219 -1.915915 1.789031
H 2.49796 -3.536482 1.912735
C -1.003856 -2.919349 -1.009135
C -2.209183 -2.042778 -1.330763
H -1.359231 -3.846476 -0.542002
H -0.522313 -3.222908 -1.945326
C -3.09502 -1.731793 -0.129726
H -2.820539 -2.538918 -2.092788
H -1.869272 -1.109366 -1.810643
C -4.337951 -0.913485 -0.471596
H -2.513526 -1.228406 0.658984
H -3.41113 -2.672837 0.335246
C -5.095757 -0.42481 0.754744
H -4.999108 -1.520784 -1.098326
H -4.076211 -0.060253 -1.120975
H -5.408527 -1.265242 1.380178
H -5.991391 0.136703 0.48
H -4.478268 0.227015 1.387512
N -1.7707 1.256388 -0.264789
H -1.951359 1.935787 -1.017101
H -2.598519 0.675006 -0.113256
H -2.325707 2.488534 1.30102
H -1.009279 0.643166 -0.565957
C -0.501591 3.210199 0.475095
H -0.5182 3.951202 1.284413
O -1.207533 3.711517 -0.654387
H -0.781376 4.499903 -0.999606
C 0.04709 -2.28592 -0.103186
H 0.482246 -1.40608 -0.602939
H -0.413927 -1.928606 0.82776
C 1.151219 -3.275775 0.257429
H 1.605322 -3.655033 -0.666857
H 0.667093 -4.142025 0.722231
C 2.646584 1.7666 -1.385098
H 2.902205 2.72025 -1.861674
H 2.734185 1.005526 -2.167221
C 1.192137 1.823401 -0.922184
H 0.557331 2.03003 -1.792649
H 0.925276 0.82412 -0.54613

conf_09

1 1
C -1.898206 -1.333754 2.238954
C -1.544462 -2.395814 1.226072
H -2.838364 -1.600393 2.726778
H -2.027355 -0.353025 1.773793
H -1.136615 -1.253416 3.018023
C -3.279007 -1.486155 -0.491931
H -3.805205 -0.916142 0.281306
H -4.069003 -1.875182 -1.147371
C -3.883912 1.504988 -1.207333
C -3.029025 2.167694 -0.128861
H -4.743718 1.018467 -0.731846
H -4.307567 2.277979 -1.857831
C -1.821721 2.930871 -0.662028
H -2.700881 1.409304 0.595206
H -3.658433 2.855594 0.447241
C -0.939536 3.537171 0.42757
H -2.17797 3.734678 -1.316669
H -1.214025 2.282253 -1.304826
C -0.229386 2.528831 1.329986
H -1.548599 4.200508 1.052616
H -0.183894 4.176754 -0.04342
H -0.965841 1.919577 1.871394
H 0.316073 3.079193 2.105224
C 2.493723 -0.254225 0.842373
C 3.763678 0.445907 0.371186
H 2.030081 -0.726275 -0.040655
H 2.784721 -1.068406 1.521968
C 4.743619 -0.493967 -0.316392
H 4.249349 0.914274 1.236352
H 3.505091 1.261963 -0.313782
C 6.020261 0.196924 -0.777443
H 4.254902 -0.962001 -1.182689
H 5.003524 -1.314191 0.366754
C 6.991651 -0.750942 -1.464354
H 6.508356 0.663147 0.087119
H 5.760248 1.017009 -1.458199
H 7.294105 -1.561801 -0.794066
H 7.898122 -0.231002 -1.783759
H 6.540645 -1.205628 -2.352324
N -0.272731 -2.046209 0.494974
H -0.198226 -2.694429 -0.301551
H 0.558236 -2.116393 1.083584
H -1.317654 -3.343203 1.722505
H -0.303766 -1.092523 0.124017
C -2.615658 -2.689436 0.169067
H -3.389936 -3.265209 0.692363
O -1.934312 -3.524806 -0.75893
H -2.537796 -3.854944 -1.429168
C 1.491847 0.66606 1.539572
H 0.75155 0.078162 2.103853
H 2.020509 1.246147 2.305042
C 0.754917 1.618517 0.605358

H 0.231701 1.039486 -0.173673
H 1.477685 2.234186 0.057902
C -3.137354 0.49946 -2.086513
H -3.849323 0.012178 -2.76268
H -2.428594 1.028512 -2.731072
C -2.373337 -0.571606 -1.31067
H -1.783192 -1.170085 -2.014309
H -1.663878 -0.046346 -0.654202

3-Keto-1-deoxySA

conf_00

1 1
C 0.358184 -2.911412 -0.047428
C -0.423563 -1.614182 0.086058
H -0.228639 -3.639209 -0.610374
H 0.575394 -3.34175 0.934495
H 1.295475 -2.752142 -0.584219
C -2.849622 -2.548613 0.139049
C -4.1905 -1.795177 0.183547
H -2.968003 -3.513647 0.64701
H -2.565959 -2.757566 -0.897887
C -4.244511 -0.58845 -0.74788
H -4.393436 -1.497224 1.217449
H -4.970578 -2.507716 -0.09644
C -3.347576 0.560646 -0.313947
H -5.279211 -0.234596 -0.806979
H -3.977263 -0.903927 -1.765653
C -3.186614 1.669032 -1.344514
H -2.347971 0.167671 -0.090636
H -3.712612 0.974 0.635379
C -2.326323 2.831149 -0.852227
H -4.172424 2.04651 -1.637008
H -2.742237 1.246573 -2.256243
C -0.973674 2.402088 -0.292089
H -2.865299 3.387291 -0.075743
H -2.165416 3.535178 -1.675896
C -0.028441 3.553694 0.026791
H -0.500532 1.712695 -1.010581
H -1.153109 1.848025 0.64475
C 1.199715 3.119072 0.820508
H -0.577779 4.304755 0.605013
H 0.278053 4.047684 -0.902897
C 2.174264 2.227469 0.056933
H 0.864656 2.603556 1.734754
H 1.741436 4.002727 1.173895
C 3.266148 1.6459 0.948132
H 2.637542 2.807001 -0.750754
H 1.63547 1.420725 -0.461257
C 4.244225 0.718061 0.235583
H 2.809684 1.113216 1.798636
H 3.820484 2.474707 1.401547

C 3.65195 -0.619802 -0.191491
H 5.098767 0.522755 0.892392
H 4.650749 1.226395 -0.647219
C 4.653127 -1.531544 -0.891145
H 2.797642 -0.458671 -0.867927
H 3.279527 -1.146646 0.707422
C 4.067278 -2.869156 -1.313867
H 5.50727 -1.695585 -0.224709
H 5.04874 -1.007805 -1.768692
H 3.695892 -3.433521 -0.451103
H 4.815374 -3.492633 -1.808534
H 3.240507 -2.736537 -2.021448
N 0.33552 -0.620116 0.904485
H 0.106993 0.343677 0.63381
H 1.351558 -0.736465 0.839798
H -0.623437 -1.171217 -0.892037
H -0.006277 -0.751757 1.876029
C -1.736367 -1.848374 0.851826
O -1.774484 -1.508516 2.013427

conf_01

1 1
C 0.230401 -2.359027 -1.430615
C -0.260454 -1.464126 -0.306219
H -0.620616 -2.74562 -1.992763
H 0.798766 -3.210384 -1.045635
H 0.857956 -1.799066 -2.127784
C -2.404451 -2.842725 0.256729
C -3.259111 -1.907053 -0.604495
H -2.94209 -3.175409 1.147356
H -2.130935 -3.742229 -0.310605
C -3.621311 -0.618009 0.123554
H -4.16079 -2.44453 -0.910513
H -2.73162 -1.667075 -1.537042
C -4.182408 0.459939 -0.794768
H -2.724391 -0.2259 0.622705
H -4.325236 -0.83376 0.936035
C -4.300526 1.835931 -0.136117
H -5.165342 0.152775 -1.167925
H -3.543372 0.534973 -1.684381
C -3.028686 2.313979 0.566375
H -5.115072 1.822272 0.596523
H -4.595413 2.562291 -0.901502
C -1.774043 2.297822 -0.30124
H -2.853639 1.700705 1.459698
H -3.191302 3.329242 0.943643
C -0.492235 2.449938 0.505473
H -1.83267 3.088645 -1.058686
H -1.724369 1.35895 -0.871061
C 0.778846 2.397273 -0.333419
H -0.467809 1.671704 1.287459
H -0.512581 3.395622 1.059765

C 2.058713 2.536129 0.486915
H 0.751665 3.199108 -1.080318
H 0.795198 1.475822 -0.943938
C 3.340208 2.417821 -0.33669
H 2.071815 1.807782 1.313584
H 2.034058 3.511661 0.983515
C 3.752686 0.99088 -0.698281
H 4.16682 2.882404 0.211648
H 3.220553 3.005933 -1.253601
C 4.323595 0.201604 0.479241
H 4.52064 1.034548 -1.478812
H 2.908086 0.462162 -1.166998
C 4.924926 -1.150684 0.103525
H 3.569239 0.063036 1.26986
H 5.104991 0.813147 0.943814
C 3.905819 -2.202347 -0.312362
H 5.500904 -1.536005 0.950435
H 5.643551 -1.004611 -0.710652
H 3.261453 -2.481218 0.535154
H 4.387476 -3.12929 -0.63274
H 3.292249 -1.866466 -1.157837
N 0.873061 -0.896543 0.487483
H 0.932307 0.122977 0.422653
H 1.781284 -1.285474 0.214075
H -0.831733 -0.623507 -0.707835
H 0.663998 -1.185199 1.470865
C -1.138763 -2.200111 0.726325
O -0.753087 -2.213453 1.874483

conf_02

1 1
C -1.206445 -2.822051 -1.237848
C -1.370671 -1.500513 -0.503248
H -2.166072 -3.14454 -1.643074
H -0.840144 -3.605985 -0.569592
H -0.514827 -2.717095 -2.076828
C -3.795457 -1.801582 0.407258
C -4.602701 -0.496724 0.577609
H -4.167312 -2.547788 1.114708
H -3.945561 -2.180214 -0.607914
C -4.219269 0.609559 -0.402532
H -4.502188 -0.146399 1.610086
H -5.651831 -0.771291 0.441892
C -3.034246 1.460664 0.046861
H -5.080064 1.26695 -0.558871
H -4.014744 0.168307 -1.389058
C -2.422241 2.284465 -1.078153
H -2.264767 0.828071 0.507578
H -3.357059 2.125621 0.856923
C -1.253495 3.162871 -0.646867
H -3.202597 2.913049 -1.521344
H -2.093403 1.614553 -1.888295

C -0.037796 2.391518 -0.145004
H -1.58457 3.852738 0.138867
H -0.945056 3.789541 -1.490756
C 1.148068 3.285558 0.199447
H 0.261906 1.676145 -0.93162
H -0.309163 1.822794 0.758886
C 2.311948 2.55902 0.863954
H 0.795258 4.078619 0.868325
H 1.495474 3.788163 -0.711539
C 3.000189 1.520606 -0.012628
H 1.961443 2.081721 1.790465
H 3.057159 3.298254 1.177601
C 4.170793 0.840906 0.684964
H 3.347652 1.995384 -0.939935
H 2.275415 0.756824 -0.332236
C 4.91809 -0.174093 -0.17725
H 3.814262 0.362847 1.606743
H 4.879429 1.609644 1.012808
C 4.055095 -1.294475 -0.752491
H 5.724768 -0.615834 0.418977
H 5.407466 0.349255 -1.006641
C 3.29053 -2.09673 0.292782
H 4.695026 -1.97735 -1.322996
H 3.346422 -0.880904 -1.484385
C 2.494617 -3.244201 -0.31593
H 2.633237 -1.425332 0.865999
H 3.989289 -2.498108 1.035075
H 1.85241 -2.906729 -1.144273
H 1.877232 -3.766331 0.42256
H 3.160891 -3.988083 -0.760483
N -0.077159 -1.057572 0.104815
H 0.107241 -0.059096 -0.046356
H 0.730098 -1.599616 -0.214655
H -1.720344 -0.718778 -1.181852
H -0.218871 -1.209625 1.129684
C -2.34072 -1.621708 0.686444
O -1.86639 -1.574363 1.800952

conf_03

1 1
C 0.629612 -2.796158 -0.599196
C -0.190433 -1.644054 -0.040994
H 0.211393 -3.114044 -1.555799
H 0.615357 -3.655384 0.077604
H 1.662864 -2.488483 -0.77505
C -2.53556 -2.28127 -0.968956
C -3.954566 -1.736046 -0.762366
H -2.582664 -3.368765 -1.116775
H -2.076342 -1.858195 -1.869357
C -4.051747 -0.222715 -0.920443
H -4.304894 -2.043767 0.227931
H -4.609185 -2.219427 -1.491951

C -3.220492 0.571725 0.076203
H -5.103183 0.066996 -0.817844
H -3.761186 0.057026 -1.941945
C -3.328822 2.079771 -0.103227
H -2.161071 0.291602 -0.031887
H -3.505028 0.293935 1.099828
C -2.584004 2.88276 0.96257
H -4.385814 2.36707 -0.081197
H -2.969071 2.350597 -1.103407
C -1.110675 2.520826 1.135855
H -3.090023 2.754585 1.926481
H -2.660133 3.948877 0.721152
C -0.264288 2.654759 -0.122779
H -1.043247 1.486914 1.51825
H -0.681543 3.145254 1.927594
C 1.180008 2.226306 0.085018
H -0.29166 3.695533 -0.467407
H -0.699735 2.062412 -0.938736
C 2.063345 2.332808 -1.153509
H 1.192472 1.179845 0.432542
H 1.627642 2.804581 0.902653
C 3.373336 1.559478 -1.013363
H 2.268603 3.389851 -1.350511
H 1.51612 1.962459 -2.031156
C 3.183582 0.0548 -1.178372
H 3.817005 1.784501 -0.035493
H 4.096631 1.905545 -1.759072
C 4.397697 -0.799212 -0.825107
H 2.892712 -0.149881 -2.215999
H 2.327448 -0.266218 -0.56723
C 4.852683 -0.713731 0.630903
H 5.234467 -0.51843 -1.474194
H 4.176982 -1.848275 -1.064981
C 3.759668 -1.031145 1.644855
H 5.255093 0.281726 0.844988
H 5.685549 -1.408304 0.776533
H 2.995384 -0.242907 1.650641
H 4.157615 -1.093249 2.660295
H 3.29763 -2.005183 1.420623
N 0.321267 -1.225533 1.300755
H 0.181321 -0.221919 1.451843
H 1.315765 -1.429425 1.438289
H -0.151661 -0.77759 -0.704677
H -0.280681 -1.731372 1.983093
C -1.643572 -2.076289 0.212128
O -1.969303 -2.28818 1.359216

conf_04

1 1
C 0.362575 0.907474 1.745615
C 0.586465 1.734027 0.493887
H -0.579339 0.362939 1.664688

H 0.316923 1.537547 2.639206
H 1.155086 0.164946 1.864293
C -1.879435 2.508668 0.203167
C -2.162159 1.576613 -0.986382
H -2.44822 3.436662 0.115985
H -2.178097 2.015565 1.135948
C -3.644107 1.243059 -1.127385
H -1.593608 0.645188 -0.877649
H -1.810602 2.056315 -1.906419
C -4.252719 0.510166 0.067408
H -3.762947 0.636746 -2.031059
H -4.205623 2.166166 -1.309464
C -3.516191 -0.759409 0.490138
H -5.286967 0.253473 -0.186924
H -4.326436 1.188709 0.926437
C -3.347352 -1.784308 -0.624904
H -4.062473 -1.21861 1.32251
H -2.529806 -0.500572 0.901644
C -2.795211 -3.128727 -0.156349
H -2.701191 -1.379097 -1.41387
H -4.320781 -1.953164 -1.101017
C -1.450147 -3.068938 0.564075
H -2.70382 -3.791349 -1.025047
H -3.526504 -3.602084 0.509251
C -0.329675 -2.41114 -0.231997
H -1.148168 -4.091859 0.817583
H -1.560877 -2.550258 1.525652
C 1.030281 -2.54859 0.438805
H -0.557141 -1.344622 -0.381291
H -0.28567 -2.846542 -1.238793
C 2.143823 -1.780535 -0.259368
H 1.301575 -3.610097 0.486466
H 0.95765 -2.21984 1.485383
C 3.496718 -1.938193 0.423017
H 1.864417 -0.7179 -0.301106
H 2.218126 -2.107102 -1.304764
C 4.681 -1.257005 -0.258695
H 3.716045 -3.009043 0.50311
H 3.425725 -1.576898 1.459508
C 4.645048 0.275015 -0.28653
H 4.785559 -1.634713 -1.283589
H 5.588926 -1.568805 0.266422
C 3.951558 0.883233 -1.503533
H 5.669891 0.657882 -0.265059
H 4.18871 0.627642 0.652756
H 4.438103 0.555808 -2.426151
H 4.023268 1.980615 -1.51499
H 2.902424 0.578577 -1.601238
N 1.913975 2.426375 0.516677
H 1.671135 3.42884 0.321109
H 2.56534 2.066281 -0.188451
H 0.569256 1.092177 -0.391133
H 2.364173 2.365449 1.429473
C -0.439437 2.867741 0.289937

O -0.002262 3.993185 0.177303

conf_05

1 1
C 0.633617 -2.620289 -1.091864
C -0.121665 -1.541474 -0.333378
H -0.005028 -3.04748 -1.866095
H 0.944005 -3.430227 -0.425237
H 1.516257 -2.20429 -1.583303
C -2.478463 -2.618018 -0.50728
C -3.890788 -2.188104 -0.089564
H -2.391107 -3.710609 -0.427577
H -2.292683 -2.363834 -1.556367
C -4.254344 -0.783801 -0.559946
H -3.983136 -2.273388 0.997862
H -4.592613 -2.906033 -0.521529
C -3.517772 0.344266 0.149777
H -5.331037 -0.63817 -0.421758
H -4.082228 -0.712201 -1.642832
C -3.749253 1.703628 -0.499422
H -2.436492 0.143135 0.160154
H -3.819024 0.368367 1.204868
C -3.245555 2.889919 0.32194
H -4.822106 1.836308 -0.679362
H -3.283371 1.706095 -1.493432
C -1.807242 2.771529 0.818258
H -3.89723 3.021964 1.193197
H -3.351459 3.802892 -0.274999
C -0.766976 2.543901 -0.270763
H -1.741986 1.952171 1.548264
H -1.549277 3.679596 1.37518
C 0.643511 2.417746 0.290764
H -0.800309 3.359672 -1.002488
H -1.016321 1.634869 -0.837826
C 1.677237 1.889957 -0.697122
H 0.610834 1.777401 1.194226
H 0.976303 3.384169 0.686015
C 3.021861 1.566208 -0.058828
H 1.81295 2.616069 -1.50615
H 1.287805 0.989009 -1.20181
C 3.994271 0.873597 -1.004324
H 2.860185 0.938063 0.831105
H 3.475505 2.48496 0.331431
C 5.290723 0.412769 -0.34058
H 4.238029 1.55291 -1.828457
H 3.493076 0.016474 -1.476772
C 5.110978 -0.570957 0.815626
H 5.837322 1.28913 0.025752
H 5.930715 -0.045493 -1.103377
C 4.387103 -1.853906 0.430148
H 4.589138 -0.083205 1.647706
H 6.098107 -0.832196 1.209381

H 3.401223 -1.646676 -0.010938
H 4.251577 -2.517846 1.288618
H 4.938329 -2.410071 -0.334271
N 0.708189 -0.972629 0.773621
H 0.741086 0.052325 0.754033
H 1.670475 -1.319254 0.769622
H -0.402574 -0.723357 -1.001538
H 0.210459 -1.279779 1.641838
C -1.387309 -2.078162 0.356369
O -1.410291 -2.075739 1.567831

conf_06

1 1
C -0.445069 -2.955716 1.239094
C 0.079008 -1.708279 0.547197
H 0.338138 -3.393429 1.859684
H -0.761262 -3.709381 0.512007
H -1.287278 -2.713217 1.891503
C 2.519177 -2.556272 0.191851
C 3.788423 -2.094197 -0.531042
H 2.418819 -3.64793 0.103049
H 2.563057 -2.339646 1.265414
C 4.278447 -0.72362 -0.073205
H 3.591576 -2.091767 -1.607431
H 4.572083 -2.835666 -0.356942
C 3.240284 0.384154 -0.180341
H 5.158807 -0.45679 -0.667961
H 4.625296 -0.789043 0.966426
C 3.777828 1.75643 0.204212
H 2.397115 0.143314 0.483935
H 2.835519 0.419447 -1.200672
C 2.743795 2.880951 0.180001
H 4.602415 2.012805 -0.470798
H 4.215734 1.699945 1.209566
C 1.584015 2.686406 1.165972
H 2.353694 2.999408 -0.839319
H 3.259915 3.818033 0.40783
C 0.331493 2.045853 0.562121
H 1.291464 3.650652 1.595342
H 1.936005 2.084256 2.012847
C -0.516621 3.007638 -0.263971
H -0.276805 1.627878 1.376712
H 0.641062 1.212536 -0.087402
C -1.686167 2.337332 -0.978608
H 0.119878 3.49813 -1.008057
H -0.89226 3.805267 0.388268
C -2.756143 1.769361 -0.049768
H -1.297716 1.546511 -1.642432
H -2.162455 3.054928 -1.655024
C -3.868449 1.02682 -0.782474
H -3.193127 2.589635 0.53223
H -2.307639 1.116615 0.716114

C -4.859071 0.325652 0.146186
H -3.434639 0.303972 -1.490298
H -4.406946 1.742102 -1.41274
C -4.313792 -0.929443 0.828425
H -5.756925 0.051706 -0.418824
H -5.189004 1.037717 0.910727
C -4.161014 -2.117992 -0.11637
H -4.986531 -1.220356 1.640619
H -3.360855 -0.69672 1.327704
H -5.138579 -2.459379 -0.46702
H -3.684781 -2.976791 0.369328
H -3.603272 -1.872716 -1.031646
N -0.961219 -1.093468 -0.331506
H -1.086156 -0.092246 -0.155026
H -1.876577 -1.546054 -0.261996
H 0.390267 -0.958971 1.279292
H -0.572111 -1.21174 -1.296034
C 1.260707 -2.004474 -0.394058
O 1.08894 -1.816545 -1.578722

conf_07

1 1
C 0.555455 -3.007082 -0.950838
C -0.077362 -1.738279 -0.401423
H -0.166224 -3.544152 -1.568242
H 0.871976 -3.673392 -0.143179
H 1.418438 -2.771775 -1.578545
C -2.507173 -2.636955 -0.110951
C -3.811446 -2.150022 0.530951
H -2.40612 -3.721297 0.040046
H -2.504513 -2.474103 -1.194703
C -4.296623 -0.813658 -0.022004
H -3.660631 -2.087292 1.613022
H -4.579737 -2.909244 0.365174
C -3.28336 0.318436 0.073955
H -5.208503 -0.530713 0.515116
H -4.592224 -0.939706 -1.071809
C -3.832163 1.655421 -0.409388
H -2.405379 0.059969 -0.536705
H -2.929303 0.416272 1.109217
C -2.833092 2.811485 -0.397948
H -4.695849 1.923624 0.209799
H -4.218271 1.533013 -1.429974
C -1.61719 2.59792 -1.308955
H -2.500779 3.001492 0.631102
H -3.363711 3.71662 -0.707124
C -0.388411 2.023372 -0.598821
H -1.323049 3.545661 -1.772412
H -1.90658 1.942632 -2.139698
C 0.408928 3.054599 0.193657
H 0.263868 1.548986 -1.345741
H -0.731493 1.242104 0.098415

C 1.529928 2.446979 1.031932
H -0.272289 3.601494 0.854159
H 0.823604 3.796993 -0.498685
C 2.664001 1.819711 0.223999
H 1.097398 1.701061 1.72054
H 1.954278 3.212611 1.689869
C 3.67494 1.069461 1.085911
H 3.181821 2.607397 -0.335703
H 2.265242 1.155635 -0.556998
C 4.716563 0.281425 0.296616
H 3.14976 0.387399 1.774634
H 4.179418 1.78981 1.738271
C 4.172174 -0.966547 -0.391891
H 5.526259 -0.022573 0.968628
H 5.176452 0.933195 -0.456265
C 5.228127 -1.738298 -1.170072
H 3.3615 -0.688386 -1.082713
H 3.751448 -1.635425 0.380818
H 5.657032 -1.116183 -1.960343
H 4.815623 -2.635722 -1.638599
H 6.044603 -2.049882 -0.513167
N 0.879272 -0.995813 0.471308
H 0.794933 0.018654 0.348243
H 1.860291 -1.249418 0.320738
H -0.386709 -1.072959 -1.21086
H 0.57214 -1.213621 1.442645
C -1.28145 -2.044515 0.505685
O -1.150087 -1.841101 1.692422

4E-1-DeoxySO

conf_00

1 1
C 0.346698 -1.684378 1.434611
C 0.096048 -2.047401 -0.009832
H 0.300165 -2.586753 2.048321
H -0.401338 -0.980842 1.809162
H 1.339863 -1.249743 1.567935
C -2.413893 -1.991012 0.277619
C -3.229684 -1.199783 -0.420657
H -2.595986 -2.142998 1.339962
C -4.448457 -0.524014 0.116975
H -3.053439 -1.07348 -1.490936
C -4.530853 0.989471 -0.135219
H -4.553323 -0.735205 1.187014
H -5.307344 -0.99982 -0.3734
C -3.919852 1.859079 0.962072
H -5.586687 1.261988 -0.219654
H -4.090218 1.227954 -1.111322
C -2.412833 1.743983 1.171001
H -4.425022 1.627913 1.907208
H -4.159681 2.906736 0.744586

C -1.582822 2.158778 -0.036759
H -2.151844 0.71918 1.468322
H -2.133942 2.378673 2.020016
C -0.093254 2.288131 0.260977
H -1.951459 3.117712 -0.421672
H -1.748181 1.45435 -0.870124
H 0.274647 1.369755 0.74362
H 0.050168 3.065661 1.019975
C 2.803762 1.162173 -0.630969
C 4.302281 1.092308 -0.366065
H 2.594674 0.626558 -1.57459
H 2.290411 0.632453 0.186911
C 4.885414 -0.314909 -0.472171
H 4.505638 1.514829 0.624635
H 4.818249 1.742643 -1.080903
C 4.218207 -1.366058 0.41308
H 5.951659 -0.270524 -0.223702
H 4.833945 -0.646071 -1.516962
C 4.218987 -1.023573 1.896448
H 4.729045 -2.322583 0.261301
H 3.185851 -1.535879 0.070929
H 5.238154 -0.851136 2.254766
H 3.797081 -1.835177 2.496089
H 3.645625 -0.115566 2.112737
N 0.096765 -0.819249 -0.873458
H -0.139624 -1.11129 -1.828343
H 0.996731 -0.326615 -0.861978
H 0.897283 -2.678153 -0.404011
H -0.630803 -0.164862 -0.561507
C -1.242093 -2.74089 -0.292559
H -1.18498 -3.735197 0.169568
O -1.265105 -2.845353 -1.704657
H -2.064307 -3.298205 -1.98746
C 0.743668 2.633637 -0.967326
H 0.480209 1.96769 -1.805463
H 0.459469 3.635181 -1.306667
C 2.252249 2.578352 -0.741408
H 2.508918 3.139768 0.165259
H 2.762446 3.085272 -1.56706

conf_01

1 1
C 2.889159 -1.102735 1.727107
C 3.196899 -1.477501 0.298437
H 3.035772 -1.976967 2.36507
H 1.850707 -0.781842 1.841364
H 3.555579 -0.316208 2.089674
C 0.855753 -2.38254 -0.153716
C 0.069931 -3.156498 0.596603
H 0.423066 -1.570748 -0.738954
C -1.404675 -3.023271 0.770354
H 0.535787 -3.975956 1.146401

C -2.091756 -1.966459 -0.077901
H -1.601698 -2.837109 1.836269
H -1.855736 -4.006256 0.57868
C -3.583218 -1.861593 0.218781
H -1.945202 -2.198645 -1.141383
H -1.617525 -0.992261 0.094401
C -4.339955 -0.946273 -0.742493
H -3.719614 -1.520203 1.252182
H -4.030423 -2.861681 0.173847
C -3.772679 0.465418 -0.868098
H -5.386846 -0.890033 -0.422583
H -4.351574 -1.409974 -1.736498
C -3.751453 1.25098 0.437386
H -4.36493 1.019636 -1.606286
H -2.760815 0.408752 -1.288333
H -3.18196 0.702149 1.197594
H -4.773105 1.313787 0.830543
C -0.741074 2.106477 0.74532
C 0.664263 1.987004 0.162138
H -1.083625 1.103553 1.023901
H -0.694527 2.679127 1.680211
C 1.316374 3.306708 -0.236125
H 0.601816 1.344413 -0.736553
H 1.293543 1.476251 0.906353
C 2.756867 3.16439 -0.722312
H 1.287511 3.99333 0.619359
H 0.719579 3.774917 -1.024013
C 3.759152 2.841741 0.381897
H 3.068459 4.09384 -1.207889
H 2.792076 2.408273 -1.52299
H 3.844238 3.678919 1.079795
H 4.763599 2.661518 -0.016457
H 3.463649 1.978784 0.992327
N 3.030857 -0.295272 -0.621026
H 3.061198 -0.660814 -1.582949
H 3.757382 0.409769 -0.48625
H 4.241999 -1.77702 0.182254
H 2.131118 0.178408 -0.473772
C 2.328614 -2.599747 -0.281347
H 2.615062 -3.516862 0.248259
O 2.742074 -2.652869 -1.645045
H 2.207458 -3.295072 -2.120375
C -3.195182 2.667123 0.297085
H -3.831712 3.231294 -0.394471
H -3.271207 3.179659 1.263751
C -1.751196 2.751368 -0.197269
H -1.496058 3.806589 -0.340977
H -1.670569 2.286995 -1.189042

conf_02

1 1
C 0.957311 1.077825 2.55985

C 1.506215 0.927676 1.158347
H -0.130665 1.137959 2.513216
H 1.334369 1.980985 3.045359
H 1.20037 0.2058 3.173948
C -0.087899 2.605628 0.146558
C -0.742733 2.614029 -1.01524
H -0.569076 2.951641 1.059144
C -2.16824 2.989026 -1.226596
H -0.214148 2.266914 -1.905888
C -2.99507 1.753993 -1.617006
H -2.220855 3.731004 -2.031337
H -2.582802 3.454237 -0.326174
C -3.253793 0.814425 -0.44198
H -2.465574 1.213527 -2.412728
H -3.94355 2.078172 -2.055262
C -3.468883 -0.629906 -0.875507
H -4.120972 1.169919 0.128318
H -2.402593 0.859487 0.248459
C -3.854664 -1.582412 0.253363
H -2.550879 -0.984641 -1.364882
H -4.246015 -0.666462 -1.64807
C -2.920061 -1.561908 1.463766
H -3.915273 -2.59915 -0.150548
H -4.866447 -1.340357 0.598813
H -3.232522 -2.346901 2.162293
H -3.048749 -0.616316 2.004085
C 0.425655 -3.213393 0.247831
C 0.997819 -2.298866 -0.832245
H 0.662257 -4.252819 -0.003508
H 0.937032 -3.014086 1.201469
C 2.512933 -2.16338 -0.764466
H 0.542063 -1.301572 -0.759939
H 0.707194 -2.671374 -1.821585
C 3.085158 -1.12089 -1.716611
H 2.983337 -3.133416 -0.964015
H 2.803519 -1.925638 0.27339
C 4.594432 -0.950598 -1.601996
H 2.5732 -0.156315 -1.55919
H 2.831083 -1.394391 -2.746168
H 4.916276 -0.68604 -0.583503
H 4.971401 -0.179164 -2.277604
H 5.110319 -1.88367 -1.842925
N 3.004153 0.720658 1.23773
H 3.379293 0.347897 0.359852
H 3.262747 0.083116 1.990764
H 1.095093 0.042514 0.671219
H 3.421456 1.647838 1.40341
C 1.326951 2.150022 0.251146
H 1.678618 1.879913 -0.756512
O 2.204539 3.122557 0.817508
H 2.056439 3.976763 0.403054
C -1.438581 -1.739703 1.14374
H -1.108931 -0.899131 0.518594
H -0.869485 -1.66803 2.081609

C -1.077146 -3.055985 0.464262
H -1.597661 -3.142575 -0.497112
H -1.441679 -3.884209 1.08247

conf_03

1 1
C 1.972864 -2.313133 1.906913
C 0.996319 -1.823118 0.864067
H 2.560215 -3.126228 1.480459
H 1.45167 -2.703744 2.787949
H 2.660883 -1.523013 2.219289
C -0.952414 -2.312103 -0.624015
C -2.272539 -2.258875 -0.429017
H -0.514072 -1.939328 -1.551009
C -3.285994 -1.730732 -1.389125
H -2.674163 -2.673818 0.499127
C -4.359491 -0.830584 -0.764507
H -3.792669 -2.605216 -1.819189
H -2.790383 -1.223138 -2.224274
C -3.945166 0.61293 -0.493185
H -4.716041 -1.291334 0.166239
H -5.22112 -0.816172 -1.438172
C -2.792738 0.777793 0.493882
H -4.816709 1.151378 -0.102807
H -3.690407 1.099076 -1.44206
C -2.596167 2.204159 1.002791
H -1.876107 0.441306 -0.012099
H -2.962705 0.107096 1.348766
C -2.072445 3.182483 -0.047974
H -1.919371 2.198608 1.866712
H -3.554981 2.568057 1.388443
H -2.138129 4.199641 0.354378
H -2.733607 3.166267 -0.920882
C 1.852476 3.031363 0.09961
C 2.276653 1.571869 -0.00317
H 2.539105 3.546263 0.780033
H 1.980755 3.509345 -0.879115
C 3.651095 1.360862 -0.623169
H 1.535906 1.008656 -0.591814
H 2.29965 1.145591 1.01822
C 4.064404 -0.1017 -0.714996
H 4.396788 1.916637 -0.041654
H 3.657278 1.803652 -1.62656
C 5.445568 -0.294491 -1.322965
H 3.325036 -0.649802 -1.316133
H 4.044812 -0.548428 0.288946
H 5.488899 0.115067 -2.336403
H 5.714544 -1.352025 -1.380009
H 6.210704 0.212794 -0.728009
N 0.19597 -0.672769 1.41308
H -0.659507 -0.531913 0.851904
H 0.744558 0.194145 1.381117

H 1.526641 -1.42365 -0.004548
H -0.087227 -0.844736 2.379178
C 0.009164 -2.88786 0.369774
H -0.552753 -3.276444 1.237441
O 0.838929 -3.882555 -0.178057
H 0.292046 -4.556154 -0.590866
C -0.634821 2.930639 -0.499838
H -0.529599 1.900173 -0.868095
H -0.424623 3.565345 -1.368092
C 0.41371 3.231892 0.568438
H 0.236707 2.627478 1.472347
H 0.281899 4.270031 0.893057

conf_04

1 1
C 0.825137 -1.725054 1.891461
C 0.789438 -2.119878 0.433555
H 0.952185 -2.618052 2.507453
H -0.09892 -1.229339 2.200513
H 1.666206 -1.060712 2.100778
C -1.651786 -2.68715 0.488051
C -2.603837 -2.218594 -0.31895
H -1.840624 -2.740044 1.558746
C -3.921083 -1.693858 0.148069
H -2.422903 -2.189483 -1.39498
C -4.17794 -0.258955 -0.327577
H -3.961982 -1.7382 1.241739
H -4.723352 -2.340988 -0.225774
C -3.032127 0.696657 -0.022616
H -5.095824 0.103775 0.146626
H -4.37135 -0.254824 -1.407033
C -3.360782 2.161918 -0.291042
H -2.170863 0.422858 -0.655901
H -2.710226 0.559927 1.018174
C -2.121373 3.04329 -0.403548
H -4.026638 2.536356 0.495857
H -3.927336 2.230596 -1.227025
C -1.297907 3.169899 0.878911
H -2.412733 4.045686 -0.734573
H -1.494596 2.640046 -1.21146
H -1.289333 2.217644 1.426736
H -1.783994 3.888656 1.546386
C 2.385209 2.817012 -0.392483
C 3.161153 1.641988 -0.982727
H 2.953937 3.259074 0.434602
H 2.290138 3.601735 -1.150593
C 3.639479 0.618729 0.043514
H 4.033956 2.011387 -1.530903
H 2.536888 1.149357 -1.747288
C 4.214152 -0.649727 -0.574731
H 2.829451 0.352697 0.742668
H 4.39684 1.085595 0.684388

C 4.724332 -1.645591 0.455958
H 5.028099 -0.376058 -1.255209
H 3.457979 -1.127763 -1.217211
H 5.528066 -1.209936 1.056036
H 5.118427 -2.548248 -0.017063
H 3.934804 -1.951368 1.152747
N 0.52412 -0.917526 -0.428547
H 0.461814 -1.236515 -1.401725
H 1.24895 -0.200281 -0.338137
H 1.757474 -2.504744 0.103108
H -0.38339 -0.502175 -0.183221
C -0.289155 -3.142011 0.05066
H -0.024782 -4.08288 0.551607
O -0.148328 -3.259627 -1.351729
H -0.75002 -3.928386 -1.68984
C 0.147946 3.588752 0.617761
H 0.167416 4.41799 -0.100291
H 0.605336 3.968496 1.537554
C 0.990177 2.435247 0.084777
H 0.456798 1.972789 -0.761048
H 1.055692 1.678985 0.882967

conf_05

1 1
C 0.078831 -1.216702 2.296757
C 0.298174 -1.812154 0.926753
H -0.033677 -2.018805 3.029422
H -0.822731 -0.599141 2.328164
H 0.93234 -0.607746 2.604451
C -2.176828 -2.105721 0.49044
C -2.82387 -1.572756 -0.546995
H -2.653459 -2.12183 1.468908
C -4.213767 -1.02736 -0.515752
H -2.345556 -1.583805 -1.528604
C -4.376665 0.391642 -1.082327
H -4.614619 -1.077151 0.502781
H -4.826959 -1.709499 -1.118519
C -4.231655 1.512453 -0.055017
H -5.377074 0.471455 -1.517031
H -3.682774 0.538993 -1.919433
C -2.860319 1.676663 0.593032
H -4.977006 1.356029 0.733654
H -4.502114 2.459578 -0.536914
C -1.746135 2.039908 -0.380821
H -2.592765 0.764298 1.143645
H -2.931001 2.467465 1.348775
C -0.458032 2.472529 0.31091
H -2.084962 2.851428 -1.036596
H -1.552779 1.20011 -1.070237
H -0.154259 1.712995 1.047899
H -0.665776 3.363583 0.914852
C 2.68841 1.987356 0.747384

C 3.309516 0.959855 -0.1954
H 1.978537 1.494913 1.430456
H 3.475471 2.375944 1.401897
C 3.808063 -0.296147 0.514889
H 4.134621 1.431644 -0.738385
H 2.601619 0.682319 -0.995934
C 4.192105 -1.441343 -0.417914
H 3.043589 -0.647926 1.226616
H 4.666569 -0.032086 1.143634
C 5.338547 -1.107725 -1.362306
H 3.314681 -1.746952 -1.009149
H 4.463662 -2.313265 0.187138
H 5.077994 -0.30349 -2.055903
H 5.620029 -1.977068 -1.960927
H 6.223831 -0.790874 -0.802327
N 0.417255 -0.731286 -0.10707
H 0.450203 -1.191213 -1.02491
H 1.254376 -0.155279 0.017606
H 1.242782 -2.360262 0.876852
H -0.407488 -0.120709 -0.091612
C -0.814458 -2.737934 0.420788
H -0.79308 -3.632427 1.057134
O -0.401003 -3.051529 -0.897212
H -1.033264 -3.649363 -1.305605
C 0.682773 2.782942 -0.652227
H 0.837238 1.938916 -1.342123
H 0.373014 3.612343 -1.29704
C 1.997972 3.149111 0.035011
H 1.803871 3.950824 0.756146
H 2.688183 3.569578 -0.704827

conf_06

1 1
C -1.230034 -1.087517 3.022914
C -0.111269 -1.498273 2.094569
H -1.535809 -1.943065 3.628849
H -2.103951 -0.735213 2.468469
H -0.907851 -0.299964 3.70888
C -1.693182 -2.390572 0.332381
C -1.716088 -1.960178 -0.930091
H -2.629189 -2.60423 0.844966
C -2.941777 -1.698386 -1.741947
H -0.766599 -1.773244 -1.43607
C -3.148302 -0.195567 -1.981485
H -3.823427 -2.123308 -1.251132
H -2.834971 -2.208541 -2.705448
C -3.730131 0.535845 -0.767136
H -3.808488 -0.056334 -2.842093
H -2.187986 0.249188 -2.273949
C -3.208978 1.961566 -0.601164
H -3.495178 -0.024734 0.149278
H -4.821654 0.536868 -0.83934

C -1.797663 1.96689 -0.030354
H -3.866946 2.531546 0.063131
H -3.222396 2.480145 -1.56748
C -1.117915 3.325842 0.063912
H -1.181301 1.29604 -0.649299
H -1.850467 1.534329 0.982975
H -1.754549 4.000692 0.646143
H -1.039565 3.766429 -0.937302
C 2.627917 2.283514 0.581404
C 3.643643 1.481023 -0.227372
H 2.434804 1.762955 1.535656
H 3.068651 3.242662 0.873885
C 3.250341 0.02794 -0.468451
H 4.609895 1.499572 0.288295
H 3.806433 1.975023 -1.193144
C 4.269151 -0.76367 -1.278129
H 2.286864 -0.019753 -1.000265
H 3.120822 -0.475666 0.504274
C 3.839946 -2.201736 -1.525925
H 5.231527 -0.747337 -0.75394
H 4.434926 -0.2553 -2.234962
H 3.682867 -2.736396 -0.58333
H 4.592798 -2.749868 -2.097002
H 2.903766 -2.24003 -2.094209
N 0.300528 -0.345858 1.228279
H 1.033702 -0.661811 0.578524
H 0.658753 0.449484 1.759205
H 0.785583 -1.780254 2.652297
H -0.496413 -0.013367 0.670358
C -0.440526 -2.643037 1.127054
H -0.572737 -3.543488 1.741628
O 0.726433 -2.741432 0.33423
H 0.635274 -3.457351 -0.300591
C 0.266899 3.255964 0.704347
H 0.620586 4.266606 0.933345
H 0.179218 2.758406 1.685221
C 1.315718 2.548458 -0.14864
H 1.520328 3.155392 -1.038685
H 0.911013 1.60774 -0.544259

conf_07

1 1
C 0.628545 -1.205407 2.205027
C 0.341734 -2.050583 0.986668
H 0.516956 -1.814591 3.104548
H -0.054885 -0.356328 2.281396
H 1.651532 -0.824013 2.1887
C -2.143932 -1.713966 1.075295
C -3.00577 -1.403593 0.1071
H -2.224891 -1.221078 2.04292
C -4.107427 -0.399485 0.233209
H -2.935801 -1.925269 -0.848517

C -4.472258 0.27999 -1.086066
H -3.851096 0.338979 1.001186
H -5.000592 -0.918354 0.605088
C -3.351202 1.044779 -1.786226
H -5.305851 0.96533 -0.898953
H -4.85953 -0.481056 -1.774396
C -2.751864 2.217145 -0.999888
H -3.755859 1.415782 -2.732579
H -2.547484 0.349806 -2.071161
C -1.457461 1.890832 -0.261879
H -3.490721 2.607603 -0.288967
H -2.540098 3.042625 -1.68787
C -0.823885 3.096872 0.421667
H -0.754323 1.481395 -1.00417
H -1.641907 1.105516 0.487117
H -1.546268 3.519872 1.128877
H -0.643699 3.876745 -0.328795
C 2.891508 1.979949 1.000134
C 3.982308 1.362377 0.129133
H 2.656337 1.29073 1.823945
H 3.280676 2.884775 1.479182
C 3.600924 0.018654 -0.482368
H 4.886346 1.225052 0.73257
H 4.255359 2.056911 -0.674449
C 4.730488 -0.700964 -1.205921
H 2.782889 0.169343 -1.207004
H 3.232769 -0.641404 0.323314
C 4.287525 -2.026121 -1.807004
H 5.555714 -0.86649 -0.5045
H 5.123984 -0.047853 -1.99273
H 3.914185 -2.70848 -1.034016
H 5.110387 -2.534444 -2.314312
H 3.492306 -1.881181 -2.547636
N 0.415427 -1.217646 -0.262524
H 0.153121 -1.815364 -1.054269
H 1.353302 -0.827415 -0.410987
H 1.096896 -2.830683 0.859535
H -0.264305 -0.447106 -0.215774
C -1.03613 -2.720655 0.953541
H -1.066262 -3.420482 1.799978
O -1.032884 -3.416655 -0.276847
H -1.835109 -3.938149 -0.365646
C 0.477747 2.791732 1.157622
H 0.801438 3.685443 1.702326
H 0.29169 2.02933 1.928061
C 1.611548 2.328883 0.251248
H 1.824799 3.10639 -0.493567
H 1.278684 1.462244 -0.333742

conf_08

1 1
C -0.603306 -1.745852 2.687484

C 0.342624 -2.114803 1.568794
H -0.947781 -2.653614 3.18734
H -1.479891 -1.209981 2.314222
H -0.107286 -1.126808 3.439383
C -1.534045 -2.507425 -0.090589
C -1.629359 -1.833923 -1.239397
H -2.435335 -2.738642 0.473735
C -2.894431 -1.332576 -1.856163
H -0.716403 -1.633047 -1.804095
C -3.069334 0.184173 -1.679812
H -3.756502 -1.853727 -1.426988
H -2.873576 -1.576495 -2.923355
C -3.65525 0.546871 -0.320174
H -3.726389 0.566149 -2.46773
H -2.101029 0.677811 -1.832853
C -3.635824 2.040546 0.000271
H -3.131523 -0.011314 0.467262
H -4.691227 0.191214 -0.284628
C -2.240411 2.654159 0.110111
H -4.172347 2.204257 0.942279
H -4.202399 2.579707 -0.767391
C -1.380854 1.992676 1.178539
H -2.335703 3.721566 0.339878
H -1.73428 2.603051 -0.860374
H -1.334197 0.913416 0.963588
H -1.888751 2.068765 2.147882
C 2.364832 2.618114 0.263586
C 3.23392 2.282887 -0.946664
H 2.690875 2.027454 1.13193
H 2.534238 3.660983 0.552322
C 3.30857 0.796931 -1.295647
H 4.251656 2.647682 -0.768158
H 2.863144 2.838438 -1.815767
C 4.01722 -0.060285 -0.25174
H 3.837918 0.684104 -2.248385
H 2.299246 0.400477 -1.490677
C 4.106171 -1.530181 -0.639108
H 3.530965 0.042041 0.729959
H 5.027496 0.33538 -0.098626
H 3.121537 -1.988847 -0.800493
H 4.620707 -2.119621 0.124161
H 4.658126 -1.650232 -1.575537
N 0.801267 -0.881749 0.846168
H 1.465309 -1.148828 0.109519
H 1.24056 -0.194931 1.460128
H 1.252008 -2.584894 1.951803
H -0.002688 -0.428899 0.394331
C -0.245083 -3.029821 0.486539
H -0.428721 -4.000654 0.96543
O 0.798927 -3.127093 -0.463244
H 0.532265 -3.707185 -1.181965
C 0.036507 2.539272 1.301368
H 0.002193 3.590641 1.609261
H 0.548323 2.022201 2.128553

C 0.872564 2.412632 0.030991
H 0.525862 3.131193 -0.719152
H 0.696244 1.429803 -0.434347

conf_09

1 1
C -1.571933 2.293464 -2.513214
C -0.205257 2.306421 -1.869459
H -1.910106 3.319167 -2.674658
H -2.309073 1.789596 -1.881866
H -1.54806 1.800288 -3.488237
C -1.136924 2.541944 0.471546
C -0.884649 1.675197 1.454467
H -2.135565 2.962864 0.371327
C -1.873512 1.197605 2.466701
H 0.127622 1.277973 1.556938
C -2.258796 -0.273916 2.258538
H -2.771533 1.823838 2.442165
H -1.428639 1.317041 3.460733
C -3.271939 -0.463356 1.135357
H -2.676247 -0.670401 3.189746
H -1.348649 -0.855547 2.065984
C -3.520453 -1.920579 0.746126
H -2.9559 0.112867 0.255134
H -4.222029 -0.013499 1.44546
C -2.307137 -2.639417 0.157278
H -4.346986 -1.959622 0.026782
H -3.864118 -2.469902 1.629884
C -1.836155 -2.04635 -1.165342
H -2.551672 -3.696294 0.004172
H -1.48818 -2.628283 0.884294
H -1.807402 -0.949146 -1.064798
H -2.588547 -2.239537 -1.938733
C 2.044098 -2.197436 -1.286714
C 3.150051 -1.743446 -0.338373
H 2.030302 -1.533732 -2.167059
H 2.282377 -3.189828 -1.684303
C 3.077867 -0.263033 0.022642
H 4.12506 -1.940258 -0.797707
H 3.113409 -2.35221 0.571907
C 4.188746 0.225187 0.948965
H 2.114546 -0.040211 0.507407
H 3.127261 0.323328 -0.90979
C 4.126398 -0.373207 2.34746
H 4.1341 1.317534 1.018185
H 5.159026 2.7e-05 0.491033
H 3.158058 -0.174184 2.820565
H 4.900485 0.052517 2.990221
H 4.273882 -1.456219 2.337262
N 0.255425 0.904535 -1.599098
H 1.167262 0.928313 -1.11973
H 0.333969 0.337495 -2.443387

H 0.546447 2.740184 -2.533902
H -0.403848 0.434394 -0.967289
C -0.11786 3.032567 -0.521167
H -0.293306 4.096717 -0.727843
O 1.21954 2.811512 -0.118074
H 1.38149 3.242781 0.725507
C -0.469938 -2.532697 -1.637495
H -0.508489 -3.606962 -1.849097
H -0.239252 -2.063669 -2.606306
C 0.660325 -2.245388 -0.648608
H 0.665597 -3.000244 0.144584
H 0.454821 -1.30238 -0.118561

ref_01

1 1
C 4.233104 -2.534505 0.979409
H 4.166522 -2.294401 2.045569
H 4.955551 -3.346616 0.869837
H 3.25819 -2.925347 0.663358
C 4.633584 -1.316456 0.161391
H 5.642094 -0.997294 0.447362
H 4.693677 -1.585012 -0.900525
C 3.68414 -0.137714 0.329625
H 2.661456 -0.490232 0.115152
H 3.672743 0.180221 1.381181
C 4.022694 1.04693 -0.565404
H 5.083754 1.287738 -0.435915
H 3.911895 0.747205 -1.616534
C 3.2008 2.305525 -0.308517
H 3.415091 2.689729 0.696319
H 3.528054 3.085046 -1.00575
C 1.693934 2.124377 -0.456979
H 1.494232 1.544377 -1.374954
H 1.310625 1.547977 0.400046
C 0.930578 3.441216 -0.556933
H 1.220855 4.09183 0.277424
H 1.257312 3.954567 -1.467538
C -0.587544 3.279332 -0.57079
H -1.046941 4.159687 -1.032578
H -0.85102 2.437754 -1.229685
C -1.204976 3.091335 0.813917
H -0.627306 2.358466 1.396568
H -1.114874 4.035655 1.36055
C -2.667801 2.651595 0.773514
H -3.158074 2.879437 1.725805
H -3.202894 3.227146 0.00784
C -2.811932 1.162437 0.493576
H -2.184815 0.919567 -0.379079
H -2.406666 0.602565 1.350339
C -4.228306 0.685801 0.194573
H -4.930675 1.147439 0.896022
H -4.52096 1.032101 -0.80311

C -4.384425 -0.841091 0.288458
H -4.396737 -1.144802 1.340554
H -5.350864 -1.129869 -0.139072
C -3.278175 -1.557168 -0.413302
H -3.165487 -1.360948 -1.481006
C -2.405903 -2.359634 0.197496
H -2.532725 -2.573459 1.257215
C -1.188433 -2.964324 -0.440595
H -1.066257 -4.008477 -0.124247
C 0.080451 -2.214148 -0.007346
H 0.937657 -2.681561 -0.499146
O -1.170243 -2.855937 -1.850629
H -1.905409 -3.345562 -2.229356
N -0.04265 -0.848385 -0.621121
H 0.78647 -0.262695 -0.480892
H -0.85793 -0.36127 -0.223249
H -0.22142 -0.966804 -1.624205
C 0.299238 -2.099713 1.483157
H 0.331848 -3.097964 1.925087
H -0.504375 -1.539747 1.968512
H 1.251728 -1.613023 1.703484

ref_02

1 1
C 3.913699 -2.061536 -0.294334
H 4.92178 -2.43807 -0.102136
H 3.321978 -2.896917 -0.680498
H 3.507368 -1.785138 0.687078
C 3.960164 -0.890363 -1.270858
H 4.493275 -1.21237 -2.170389
H 2.952507 -0.630647 -1.630011
C 4.641261 0.356109 -0.705404
H 5.606392 0.061441 -0.278664
H 4.870559 1.047893 -1.523576
C 3.831097 1.106784 0.350123
H 3.507884 0.408516 1.142525
H 4.483352 1.813881 0.873659
C 2.635742 1.875225 -0.205772
H 3.002226 2.650557 -0.888602
H 2.021258 1.222331 -0.84184
C 1.784358 2.532543 0.876319
H 1.516399 1.795508 1.652381
H 2.400992 3.272687 1.39722
C 0.509619 3.200108 0.36768
H 0.097526 3.834758 1.15967
H 0.753761 3.872419 -0.463652
C -0.573637 2.221639 -0.071689
H -0.206941 1.60296 -0.908313
H -0.797813 1.552247 0.773159
C -1.865515 2.892018 -0.527157
H -2.226135 3.552952 0.270863
H -1.63817 3.540379 -1.380431

C -2.965777 1.907932 -0.914155
H -3.76187 2.445161 -1.441824
H -2.558007 1.192802 -1.640767
C -3.579581 1.171799 0.272898
H -2.792124 0.687199 0.863138
H -4.041784 1.907677 0.940755
C -4.625287 0.127204 -0.103745
H -5.095834 -0.259159 0.807532
H -5.425853 0.600804 -0.682353
C -4.076309 -1.049804 -0.924962
H -4.911131 -1.708152 -1.189098
H -3.651446 -0.688871 -1.866375
C -3.054546 -1.846235 -0.185779
H -3.418437 -2.459407 0.640754
C -1.748501 -1.842277 -0.452601
H -1.380934 -1.248416 -1.292067
C -0.720413 -2.649739 0.278612
H -1.206088 -3.42563 0.883308
C 0.161898 -1.839886 1.232598
H 0.934204 -2.514544 1.611947
O 0.221114 -3.215953 -0.625667
H -0.243466 -3.712219 -1.305425
N 0.895597 -0.845899 0.378943
H 0.274655 -0.089285 0.069816
H 1.257945 -1.344238 -0.443614
H 1.686868 -0.410185 0.858389
C -0.56585 -1.149644 2.359169
H -1.032868 -1.90027 3.000061
H -1.354077 -0.493058 1.983261
H 0.119416 -0.567944 2.981194

5E-1-DeoxySO

conf_00

1 1
C 1.977427 -0.826924 -2.552599
C 0.676708 -1.43279 -2.076967
H 2.490728 -1.53238 -3.209415
H 2.642475 -0.591215 -1.718437
H 1.802273 0.086689 -3.126155
C 1.813548 -2.824992 -0.209421
C 1.583708 -1.810887 0.871654
H 2.824676 -2.72068 -0.615938
H 1.765124 -3.837397 0.213604
C 2.496152 -0.924749 1.279196
H 0.613201 -1.837551 1.371483
C 2.31378 0.0893 2.361715
H 3.480373 -0.943038 0.805378
C 2.974282 1.431415 2.030774
H 2.76962 -0.29835 3.281648
H 1.247615 0.210639 2.584599
C 2.554952 2.044107 0.698181

H 4.06114 1.290806 2.023417
H 2.766669 2.136642 2.842517
C 1.08467 2.429488 0.599329
H 2.804319 1.349311 -0.118115
H 3.164456 2.935517 0.512995
C 0.743218 3.039625 -0.758366
H 0.843075 3.152625 1.388084
H 0.454653 1.557641 0.817595
H 0.972282 2.319256 -1.561838
H 1.446287 3.863514 -0.926623
C -2.258158 1.722878 -0.078503
C -3.33631 0.712474 -0.452842
H -2.654528 2.401471 0.686463
H -1.42183 1.213641 0.412526
C -3.775325 -0.180129 0.704106
H -2.99038 0.079404 -1.286888
H -4.203218 1.25239 -0.849135
C -2.703911 -1.154173 1.181563
H -4.657889 -0.754162 0.400677
H -4.096569 0.447003 1.545164
C -3.173258 -2.051419 2.31781
H -1.814781 -0.600276 1.515049
H -2.3952 -1.787851 0.336254
H -3.466923 -1.458237 3.188604
H -2.389883 -2.744146 2.637894
H -4.040821 -2.644878 2.015295
N -0.023303 -0.491765 -1.138138
H -0.855362 -0.956552 -0.751958
H -0.30749 0.385041 -1.581881
H -0.014459 -1.57427 -2.912016
H 0.601358 -0.279818 -0.346011
C 0.803326 -2.778538 -1.359802
H 1.109388 -3.504698 -2.123845
O -0.518847 -3.047327 -0.921955
H -0.578084 -3.930966 -0.550244
C -1.765387 2.553271 -1.257971
H -1.421492 1.897291 -2.08014
H -2.629186 3.073631 -1.687239
C -0.675222 3.580054 -0.953268
H -0.966114 4.166365 -0.072846
H -0.646809 4.29048 -1.785448

conf_01

1 1
C -1.037134 -0.360333 2.44235
C -1.24734 -1.336114 1.307345
H -1.514431 -0.745248 3.346167
H -1.463871 0.620867 2.224467
H 0.026335 -0.234587 2.660332
C -3.660429 -0.518707 0.836024
C -3.325108 0.40329 -0.298668
H -3.70442 0.035874 1.77876

H -4.664454 -0.93513 0.685815
C -2.914171 1.666152 -0.155729
H -3.44229 0.000654 -1.3056
C -2.532812 2.574968 -1.283313
H -2.82779 2.072365 0.8531
C -1.02511 2.557538 -1.568268
H -2.840071 3.597487 -1.040028
H -3.073129 2.28747 -2.190259
C -0.145765 2.840716 -0.357447
H -0.806672 3.289621 -2.353199
H -0.754274 1.581398 -2.000393
C 1.336233 2.94627 -0.699405
H -0.290289 2.063419 0.408781
H -0.470423 3.772549 0.122654
C 2.23423 2.942062 0.538037
H 1.495897 3.861893 -1.278194
H 1.621897 2.122467 -1.368327
H 1.76295 3.548861 1.319935
H 3.185625 3.434023 0.309341
C 3.946259 -0.585965 0.766508
C 2.839753 -1.626561 0.612704
H 4.240937 -0.532472 1.821128
H 4.82832 -0.947631 0.225567
C 2.375089 -1.839228 -0.824675
H 1.98524 -1.364473 1.259788
H 3.200188 -2.583152 1.007932
C 1.476985 -3.059637 -1.003197
H 3.253143 -1.946693 -1.472761
H 1.871035 -0.937306 -1.210426
C 0.815167 -3.130333 -2.37134
H 0.712529 -3.0926 -0.21214
H 2.074787 -3.962188 -0.837786
H 0.186056 -2.252219 -2.572071
H 0.185745 -4.017447 -2.47144
H 1.564269 -3.16113 -3.167352
N -0.643959 -0.805934 0.037348
H -0.829463 -1.48159 -0.71231
H 0.366255 -0.667753 0.118804
H -0.712617 -2.26944 1.503621
H -1.106336 0.079791 -0.218949
C -2.701548 -1.702417 1.002124
H -3.04484 -2.313566 1.846657
O -2.605029 -2.49024 -0.174637
H -3.449103 -2.899654 -0.379536
C 3.609425 0.816896 0.26875
H 4.52125 1.423234 0.307875
H 3.330702 0.781056 -0.791634
C 2.534305 1.541029 1.072564
H 2.859254 1.602488 2.117793
H 1.60183 0.954455 1.093366

conf_02

1 1
C -1.228805 -1.07836 2.344174
C -0.731779 -1.839251 1.136175
H -1.530385 -1.785793 3.119811
H -2.0892 -0.449616 2.105771
H -0.443538 -0.446154 2.763964
C -3.133847 -2.379326 0.337948
C -3.269542 -1.271476 -0.665182
H -3.561519 -2.078603 1.299559
H -3.72514 -3.241768 0.005223
C -3.648176 -0.022542 -0.378235
H -3.07065 -1.531245 -1.705778
C -3.785258 1.078728 -1.387423
H -3.876269 0.225576 0.659401
C -2.480375 1.849521 -1.61855
H -4.562723 1.774527 -1.056218
H -4.121258 0.660153 -2.340958
C -1.863955 2.461771 -0.36748
H -2.664072 2.639756 -2.354463
H -1.751195 1.174518 -2.092485
C -0.593876 3.249901 -0.669749
H -1.651851 1.673378 0.370216
H -2.594235 3.1165 0.124276
C 0.205764 3.664159 0.563768
H -0.867231 4.148053 -1.23392
H 0.043234 2.667868 -1.348827
H -0.436646 4.251897 1.229617
H 1.011735 4.337692 0.251104
C 2.426977 0.53061 1.387215
C 3.207472 -0.49329 0.56737
H 1.639507 0.016022 1.961697
H 3.082418 0.961197 2.15297
C 4.406272 0.088554 -0.17322
H 2.537988 -0.976781 -0.161575
H 3.55377 -1.295137 1.231022
C 5.254103 -0.957219 -0.8919
H 5.03222 0.627682 0.548566
H 4.067512 0.834846 -0.902925
C 4.523184 -1.683877 -2.013447
H 5.627139 -1.685445 -0.161105
H 6.139944 -0.462756 -1.303556
H 3.693535 -2.292104 -1.638726
H 5.195888 -2.357771 -2.549459
H 4.118464 -0.974342 -2.743757
N -0.389227 -0.895225 0.017379
H -0.110078 -1.458206 -0.793196
H 0.363852 -0.248291 0.267889
H 0.204483 -2.355652 1.365114
H -1.232069 -0.365479 -0.253983
C -1.703091 -2.87682 0.567865
H -1.732283 -3.69753 1.295798
O -1.075052 -3.300051 -0.632678
H -1.524957 -4.063297 -1.002995
C 1.811611 1.66481 0.569876

H 2.606548 2.311688 0.183822
H 1.337839 1.262576 -0.342428
C 0.815582 2.508898 1.356475
H 1.322664 2.914379 2.239133
H 0.018008 1.865912 1.757103

conf_03

1 1
C 3.526114 -2.647381 -0.313325
C 2.461397 -2.045047 -1.198879
H 3.407001 -3.732816 -0.289177
H 3.45787 -2.279835 0.714173
H 4.528691 -2.43741 -0.694033
C 0.65443 -2.195263 0.6491
C -0.813501 -2.402217 0.863738
H 0.930877 -1.19291 1.000793
H 1.229254 -2.919513 1.237908
C -1.641121 -1.470317 1.338379
H -1.205311 -3.390539 0.619939
C -3.091756 -1.667382 1.640894
H -1.226086 -0.493703 1.597734
C -4.00981 -0.483651 1.322009
H -3.162597 -1.858232 2.720632
H -3.455197 -2.577425 1.149439
C -4.34966 -0.275987 -0.152719
H -3.581311 0.431533 1.748881
H -4.949405 -0.642468 1.860225
C -3.196934 0.129199 -1.068047
H -5.128115 0.493676 -0.217268
H -4.805485 -1.19469 -0.541641
C -2.502873 1.425259 -0.673386
H -3.580386 0.224853 -2.090666
H -2.461641 -0.685992 -1.104072
H -2.075429 1.323857 0.330716
H -3.251035 2.225586 -0.598083
C -0.04757 3.20042 0.070184
C 1.052991 2.160885 0.24313
H -0.788311 3.074099 0.868578
H 0.385232 4.195733 0.222035
C 1.764088 2.254616 1.588468
H 1.77412 2.287651 -0.578814
H 0.615393 1.155704 0.140129
C 2.92983 1.283517 1.758518
H 1.031655 2.082314 2.385173
H 2.132144 3.277543 1.733677
C 4.15135 1.616755 0.907131
H 2.575069 0.254148 1.567855
H 3.236955 1.262112 2.808683
H 3.914175 1.734903 -0.157281
H 4.936408 0.860635 1.003994
H 4.582598 2.570174 1.222713
N 2.565527 -0.542188 -1.22412

H 1.673346 -0.203559 -1.622743
H 3.351147 -0.215518 -1.784605
H 2.604621 -2.347359 -2.239905
H 2.660197 -0.140895 -0.285359
C 1.011289 -2.36765 -0.826255
H 0.837366 -3.412517 -1.116239
O 0.247384 -1.501677 -1.646756
H -0.654567 -1.45919 -1.30018
C -0.731549 3.166275 -1.294732
H -1.475416 3.970253 -1.331025
H 0.007013 3.407942 -2.069865
C -1.414995 1.847664 -1.651691
H -0.669231 1.043813 -1.730652
H -1.8497 1.938607 -2.653875

conf_04

1 1
C 1.003904 4.322619 -0.54304
C 1.496572 2.964646 -0.092253
H 0.508441 4.825232 0.288589
H 0.286567 4.230715 -1.361909
H 1.832198 4.964305 -0.857315
C -0.633349 2.41445 1.244547
C -1.577152 1.281513 1.50388
H -1.172987 3.27951 0.847716
H -0.123518 2.722266 2.165747
C -2.826412 1.212267 1.043668
H -1.172098 0.435993 2.060467
C -3.74114 0.044585 1.220009
H -3.233403 2.068063 0.500241
C -4.480977 -0.327337 -0.07054
H -4.493553 0.303462 1.976239
H -3.179375 -0.803663 1.622455
C -3.612396 -0.706283 -1.273238
H -5.107413 0.528675 -0.347947
H -5.176824 -1.145723 0.14535
C -3.192866 -2.172721 -1.379053
H -2.725782 -0.056261 -1.309159
H -4.173573 -0.467426 -2.182572
C -2.217677 -2.7049 -0.332444
H -2.745332 -2.325403 -2.369453
H -4.098883 -2.790234 -1.36495
H -2.020241 -3.759599 -0.559595
H -2.678225 -2.701934 0.66292
C 1.401038 -1.843749 0.882811
C 2.140229 -1.332873 -0.348518
H 2.080258 -2.45041 1.490877
H 1.119754 -0.989766 1.517699
C 3.414892 -0.552752 -0.023622
H 1.464173 -0.701485 -0.940554
H 2.390535 -2.176779 -1.001479
C 4.513148 -1.338323 0.696161

H 3.160333 0.311174 0.61624
H 3.848945 -0.16995 -0.964084
C 4.983433 -2.5678 -0.066713
H 4.15861 -1.630094 1.690259
H 5.361845 -0.667432 0.867997
H 4.193599 -3.318025 -0.161015
H 5.822264 -3.04336 0.446406
H 5.318644 -2.307412 -1.076268
N 2.27591 2.327646 -1.222448
H 2.794608 1.498796 -0.902066
H 2.926299 2.978276 -1.662475
H 2.185432 3.052027 0.751609
H 1.576815 2.007271 -1.907966
C 0.404221 1.946845 0.229823
H 0.892564 1.045949 0.631594
O -0.177211 1.642886 -1.024787
H -0.966028 1.104178 -0.875986
C 0.156107 -2.670322 0.571802
H 0.453235 -3.600422 0.071169
H -0.302109 -2.975842 1.52011
C -0.889992 -1.958771 -0.279159
H -0.514688 -1.826332 -1.302599
H -1.054273 -0.951501 0.124418

conf_05

1 1
C 0.940697 -2.739533 1.751831
C 0.296126 -1.711154 0.849091
H 2.012987 -2.763498 1.553734
H 0.532869 -3.738009 1.579064
H 0.816583 -2.474498 2.805956
C 1.686761 -2.311271 -1.191358
C 2.555061 -1.106259 -1.000238
H 1.577772 -2.53336 -2.261821
H 2.125877 -3.198999 -0.726546
C 3.773126 -1.120241 -0.461739
H 2.131716 -0.162041 -1.340608
C 4.67203 0.067214 -0.279591
H 4.187952 -2.080221 -0.151094
C 3.976033 1.424284 -0.276138
H 5.43551 0.033571 -1.068034
H 5.228623 -0.058746 0.657715
C 3.179256 1.667756 1.006674
H 3.333421 1.513883 -1.158826
H 4.728155 2.211967 -0.385082
C 2.09904 2.739913 0.900172
H 3.880321 1.92894 1.806918
H 2.70619 0.726641 1.321904
C 0.901101 2.299415 0.066764
H 2.522065 3.66212 0.483085
H 1.74988 2.996991 1.907616
H 0.599584 1.301339 0.415177

H 1.191183 2.175215 -0.984919
C -2.042851 1.439051 -0.375871
C -3.270572 1.057814 -1.192726
H -2.277598 1.4208 0.700227
H -1.257735 0.686705 -0.559743
C -3.796589 -0.347041 -0.906284
H -3.018697 1.130675 -2.256717
H -4.062012 1.795974 -1.024045
C -4.376179 -0.559741 0.494012
H -2.992114 -1.075579 -1.098273
H -4.577028 -0.592842 -1.63493
C -5.633626 0.255481 0.764443
H -3.635682 -0.30959 1.269925
H -4.605623 -1.624701 0.62174
H -5.436155 1.330213 0.735327
H -6.050893 0.02516 1.747489
H -6.402591 0.038508 0.017281
N -1.159817 -1.566242 1.22125
H -1.579847 -0.731631 0.794965
H -1.29766 -1.518285 2.230597
H 0.756316 -0.730579 0.984967
H -1.655179 -2.386158 0.846187
C 0.290167 -2.067009 -0.637129
H -0.150894 -1.216567 -1.182335
O -0.576348 -3.186864 -0.727509
H -0.613995 -3.51256 -1.630242
C -1.475631 2.812769 -0.720559
H -1.178485 2.817208 -1.777004
H -2.277457 3.553752 -0.627795
C -0.29388 3.23963 0.145535
H -0.61843 3.324513 1.191505
H 0.020151 4.245833 -0.153367

conf_06

1 1
C -2.011583 0.010145 2.621366
C -1.571105 -1.104523 1.698991
H -2.729097 -0.376559 3.348154
H -2.488106 0.828152 2.076652
H -1.164246 0.414126 3.18125
C -3.710267 -0.957256 0.244871
C -3.132071 -0.133092 -0.867851
H -4.213598 -0.316394 0.97567
H -4.486671 -1.619361 -0.158524
C -3.043852 1.200446 -0.868347
H -2.769738 -0.680848 -1.738903
C -2.416185 2.007503 -1.963551
H -3.435024 1.747989 -0.009809
C -0.9397 2.323904 -1.690517
H -2.969826 2.944092 -2.088611
H -2.494151 1.462821 -2.909363
C -0.684805 3.137467 -0.425091

H -0.532908 2.869851 -2.54857
H -0.380755 1.376709 -1.661332
C 0.798141 3.328123 -0.11281
H -1.173698 2.667761 0.441925
H -1.167972 4.114275 -0.537531
C 1.465345 2.08439 0.463468
H 0.922654 4.152787 0.596642
H 1.325712 3.631654 -1.025694
H 1.247646 1.226033 -0.187911
H 1.02317 1.876114 1.453432
C 3.25453 -0.349243 0.916172
C 3.544324 -0.621333 -0.55489
H 2.191528 -0.53921 1.128513
H 3.795347 -1.08037 1.528926
C 3.24192 -2.048402 -1.001821
H 4.601417 -0.403962 -0.747062
H 2.978826 0.076719 -1.187031
C 1.772805 -2.448078 -0.900741
H 3.842748 -2.754883 -0.415426
H 3.562234 -2.170048 -2.042814
C 1.47629 -3.813263 -1.503121
H 1.169105 -1.682992 -1.41765
H 1.474358 -2.457193 0.157982
H 1.726986 -3.833341 -2.567405
H 0.420956 -4.080227 -1.400084
H 2.066221 -4.591313 -1.010398
N -0.642421 -0.569467 0.648916
H -0.285835 -1.34158 0.074273
H 0.150493 -0.055454 1.040162
H -0.989637 -1.8493 2.248928
H -1.172229 0.063482 0.025884
C -2.696158 -1.847091 0.974895
H -3.228322 -2.419459 1.745539
O -2.009132 -2.718165 0.091031
H -2.61709 -3.351835 -0.297502
C 3.620682 1.056096 1.389663
H 3.349984 1.151025 2.448661
H 4.709307 1.172167 1.347688
C 2.977036 2.199187 0.605421
H 3.417306 2.264501 -0.396411
H 3.220816 3.145771 1.100465

conf_07

1 1
C 1.735979 -0.086354 2.481691
C 1.928682 1.060464 1.514367
H 2.503789 -0.043707 3.257152
H 1.808573 -1.056477 1.985356
H 0.76425 -0.02413 2.977003
C 3.861817 -0.134601 0.280377
C 3.068585 -0.694805 -0.865028
H 3.959466 -0.879189 1.076718

H 4.884039 0.072681 -0.06046
C 2.397489 -1.850704 -0.834835
H 3.087236 -0.124816 -1.794729
C 1.633074 -2.430353 -1.98385
H 2.408761 -2.432549 0.088222
C 0.120861 -2.191458 -1.898585
H 1.822428 -3.509068 -2.021576
H 2.008603 -2.01044 -2.922032
C -0.541417 -2.667487 -0.611984
H -0.35614 -2.67947 -2.755549
H -0.077131 -1.116162 -2.028234
C -2.054493 -2.472514 -0.642758
H -0.111807 -2.127583 0.243648
H -0.306091 -3.724841 -0.436837
C -2.757174 -2.626912 0.704717
H -2.488967 -3.183326 -1.353928
H -2.277582 -1.480212 -1.057628
H -2.66304 -3.660603 1.055634
H -3.82926 -2.462118 0.554397
C -3.359134 0.500923 0.998274
C -3.07861 1.859781 0.359215
H -3.964473 0.626537 1.902075
H -3.959699 -0.104593 0.309396
C -2.634272 1.772534 -1.100056
H -2.327282 2.391889 0.962164
H -3.978558 2.482106 0.403844
C -2.178871 3.104783 -1.69184
H -3.471451 1.379508 -1.687454
H -1.826706 1.033222 -1.22042
C -0.78425 3.530992 -1.251207
H -2.901211 3.882949 -1.419979
H -2.197757 3.043954 -2.784477
H -0.69259 3.568766 -0.159067
H -0.528009 4.525351 -1.623957
H -0.028415 2.843458 -1.656449
N 0.936515 0.964705 0.391289
H 1.024009 1.786522 -0.214603
H -0.027797 0.886501 0.729068
H 1.717754 2.014519 2.004798
H 1.159069 0.131181 -0.178996
C 3.317636 1.165389 0.882316
H 3.994832 1.485703 1.684524
O 3.1699 2.191717 -0.085483
H 4.025671 2.458976 -0.429326
C -2.079957 -0.241425 1.362431
H -1.427378 -0.241852 0.477249
H -1.571864 0.323343 2.159653
C -2.241879 -1.692482 1.804259
H -2.919466 -1.739026 2.663761
H -1.274992 -2.058311 2.170848

conf_08

1 1
C 2.089099 -0.40633 2.48272
C 2.561073 0.293441 1.228609
H 2.936517 -0.569633 3.151938
H 1.637673 -1.376335 2.262024
H 1.359173 0.202368 3.022434
C 3.455184 -1.89133 0.156537
C 2.225749 -2.167274 -0.65609
H 3.426253 -2.450533 1.097117
H 4.343735 -2.250032 -0.379113
C 1.195065 -2.916873 -0.255008
H 2.192698 -1.724279 -1.652968
C -0.063395 -3.130579 -1.027031
H 1.245263 -3.395444 0.725512
C -1.294607 -2.710575 -0.218578
H -0.157732 -4.188918 -1.299
H -0.018934 -2.570488 -1.96875
C -2.554248 -2.601025 -1.066626
H -1.099119 -1.740979 0.262552
H -1.452691 -3.420559 0.602935
C -3.822647 -2.316507 -0.263064
H -2.692342 -3.531971 -1.628652
H -2.405293 -1.81801 -1.821243
C -3.734032 -1.126059 0.689757
H -4.085135 -3.20819 0.318229
H -4.650031 -2.159576 -0.964491
H -3.03159 -1.354623 1.502725
H -4.707389 -0.994463 1.176184
C -2.712959 2.644009 0.473558
C -1.215925 2.578405 0.194004
H -2.900181 3.458911 1.181775
H -3.236626 2.916474 -0.451164
C -0.609131 3.890164 -0.287953
H -1.029618 1.788789 -0.547097
H -0.715881 2.284807 1.137105
C 0.876982 3.796106 -0.626674
H -0.760742 4.651552 0.484636
H -1.159482 4.23832 -1.170604
C 1.172504 3.035 -1.915272
H 1.414051 3.347595 0.224389
H 1.294174 4.803472 -0.717026
H 0.671395 2.059041 -1.958787
H 2.245964 2.879182 -2.059943
H 0.806931 3.591265 -2.782528
N 1.418924 0.473578 0.269819
H 1.778903 0.911172 -0.58679
H 0.665183 1.058842 0.642298
H 2.905128 1.305935 1.456569
H 1.050735 -0.455599 0.013115
C 3.686385 -0.407023 0.461852
H 4.585833 -0.312576 1.083485
O 3.815379 0.36946 -0.719567
H 4.590607 0.100222 -1.218336
C -3.288865 1.34547 1.030063

H -2.709948 1.047521 1.916746
H -4.306407 1.528897 1.39178
C -3.322449 0.187738 0.038071
H -2.33704 0.067329 -0.431522
H -4.01222 0.429239 -0.781002

conf_09

1 1
C -0.708134 -1.981398 -1.745848
C -0.901031 -2.090301 -0.251304
H -1.071323 -2.891402 -2.228335
H 0.343126 -1.850149 -2.013063
H -1.280357 -1.144186 -2.153573
C 1.223621 -3.539399 0.083025
C 2.121172 -2.388719 0.425481
H 1.31344 -3.79255 -0.978184
H 1.538881 -4.435627 0.633432
C 2.892679 -1.731553 -0.444857
H 2.148015 -2.089518 1.474979
C 3.736006 -0.544558 -0.11753
H 2.90469 -2.059762 -1.486496
C 3.506762 0.607713 -1.10678
H 4.795684 -0.826589 -0.139157
H 3.527132 -0.226684 0.909995
C 3.846123 1.98249 -0.535505
H 2.461743 0.603332 -1.443582
H 4.100649 0.426305 -2.008332
C 2.867715 2.489429 0.524599
H 3.878927 2.707306 -1.356864
H 4.857649 1.957092 -0.11398
C 1.45158 2.707176 0.004728
H 3.246198 3.436702 0.924327
H 2.841011 1.802689 1.381778
H 1.056225 1.760806 -0.388563
H 1.482894 3.387235 -0.856923
C -1.666425 2.231018 0.157131
C -3.093448 2.449194 -0.329408
H -1.678689 1.576062 1.043053
H -1.112466 1.720266 -0.649164
C -3.772098 1.178054 -0.830856
H -3.075814 3.188243 -1.137987
H -3.688491 2.893903 0.477461
C -4.04456 0.129132 0.241888
H -3.161579 0.734976 -1.632943
H -4.724478 1.441727 -1.303886
C -4.694632 -1.129768 -0.313347
H -4.687262 0.565562 1.015292
H -3.115179 -0.138887 0.765889
H -5.66116 -0.900283 -0.770424
H -4.869733 -1.875433 0.466934
H -4.077368 -1.59078 -1.093919
N -0.359056 -0.866639 0.435416

H -0.43623 -1.018726 1.447244
H -0.855122 -0.006441 0.177961
H -1.964686 -2.108098 -0.001068
H 0.643921 -0.774871 0.214737
C -0.252384 -3.3039 0.419899
H -0.831817 -4.178758 0.099211
O -0.452446 -3.057656 1.804808
H -0.199246 -3.822995 2.326749
C -0.922384 3.506253 0.535277
H -0.889626 4.173363 -0.33491
H -1.502517 4.032588 1.301295
C 0.49517 3.269015 1.049958
H 0.460964 2.596951 1.920225
H 0.900557 4.215071 1.425102

conf_10

1 1
C 0.710665 -1.98011 1.745076
C 0.902698 -2.089029 0.250391
H 1.07494 -2.889753 2.227417
H -0.340522 -1.849703 2.012987
H 1.282467 -1.142369 2.1523
C -1.220988 -3.539857 -0.082173
C -2.119612 -2.389967 -0.424617
H -1.309995 -3.792624 0.979184
H -1.535932 -4.436527 -0.632035
C -2.890922 -1.732788 0.445886
H -2.147339 -2.091397 -1.474267
C -3.73553 -0.546711 0.118404
H -2.901932 -2.060359 1.487747
C -3.507621 0.606166 1.107188
H -4.79488 -0.830013 0.140048
H -3.527129 -0.228952 -0.90927
C -3.847765 1.980362 0.534965
H -2.462768 0.602804 1.444521
H -4.101799 0.424895 2.008574
C -2.869378 2.487321 -0.525119
H -3.881282 2.705673 1.355861
H -4.85916 1.954006 0.113183
C -1.453477 2.705852 -0.004946
H -3.248161 3.434316 -0.925238
H -2.842215 1.800334 -1.382081
H -1.057738 1.759818 0.388752
H -1.485368 3.386168 0.856479
C 1.665131 2.23148 -0.157066
C 3.091965 2.450584 0.329561
H 1.67799 1.576739 -1.043168
H 1.111509 1.720107 0.649068
C 3.771425 1.179884 0.831011
H 3.073845 3.189603 1.138164
H 3.6867 2.895735 -0.477289
C 4.044122 0.130936 -0.241651

H 3.16133 0.736603 1.63328
H 4.723769 1.444145 1.303799
C 4.695069 -1.127517 0.313538
H 4.686271 0.567615 -1.015373
H 3.114658 -0.137697 -0.765205
H 5.661346 -0.897382 0.770818
H 4.870862 -1.872958 -0.466806
H 4.07798 -1.589135 1.093888
N 0.359283 -0.865879 -0.436049
H 0.436319 -1.01766 -1.447915
H 0.85449 -0.005199 -0.17848
H 1.966234 -2.105987 -0.000423
H -0.643739 -0.775246 -0.215052
C 0.254568 -3.303209 -0.420255
H 0.835008 -4.177545 -0.099948
O 0.453175 -3.056833 -1.805273
H 0.201971 -3.823002 -2.326934
C 0.920164 3.506195 -0.53514
H 0.886731 4.173185 0.335111
H 1.500073 4.033057 -1.300964
C -0.497118 3.267938 -1.050117
H -0.462262 2.595707 -1.920233
H -0.903028 4.213636 -1.425598

conf_11

1 1
C 2.57119 0.607315 1.921226
C 2.731907 0.920438 0.451072
H 3.521816 0.771704 2.433326
H 2.270495 -0.429263 2.08729
H 1.831941 1.263158 2.386777
C 3.85501 -1.358715 -0.04624
C 2.603785 -2.050853 -0.497055
H 4.048187 -1.564171 1.01134
H 4.717643 -1.761074 -0.592619
C 1.75286 -2.697846 0.304411
H 2.392956 -2.024798 -1.567229
C 0.480194 -3.336045 -0.139508
H 1.978056 -2.758704 1.371389
C -0.748746 -2.680295 0.495995
H 0.491142 -4.399437 0.128702
H 0.403421 -3.289057 -1.231854
C -2.051868 -3.241841 -0.054632
H -0.718988 -1.595031 0.314411
H -0.714947 -2.805704 1.585863
C -3.311012 -2.669218 0.589527
H -2.051165 -4.330174 0.076837
H -2.080046 -3.07174 -1.138077
C -3.460405 -1.150336 0.503338
H -3.350681 -2.972984 1.642438
H -4.179213 -3.134261 0.108463
H -2.741928 -0.663967 1.176576

H -4.449709 -0.883592 0.894145
C -3.393052 1.870556 -0.104882
C -1.888288 2.119767 -0.127609
H -3.693531 1.609232 0.916802
H -3.89941 2.816327 -0.33042
C -1.500456 3.342159 0.697791
H -1.561277 2.240088 -1.169373
H -1.378752 1.224935 0.271435
C -0.004428 3.626156 0.784961
H -1.900064 3.223852 1.711521
H -2.002449 4.22207 0.277437
C 0.653085 3.965234 -0.548087
H 0.498862 2.772216 1.269379
H 0.159609 4.458688 1.476573
H 0.542201 3.173085 -1.297755
H 1.72027 4.177211 -0.434213
H 0.193441 4.857528 -0.981935
N 1.455228 0.638035 -0.293244
H 1.637707 0.786105 -1.291863
H 0.685854 1.241215 0.01062
H 2.913808 1.987696 0.301096
H 1.204498 -0.354855 -0.168752
C 3.848861 0.157661 -0.266727
H 4.793176 0.571817 0.108609
O 3.66279 0.503788 -1.631119
H 4.395605 0.189085 -2.166058
C -3.893726 0.813842 -1.084325
H -4.983963 0.752031 -0.988327
H -3.701346 1.15392 -2.109497
C -3.313233 -0.587798 -0.907561
H -2.25499 -0.594821 -1.205032
H -3.817199 -1.255956 -1.616536

conf_12

1 1
C -2.895607 0.874455 -1.776184
C -2.934977 1.133725 -0.287537
H -3.888852 1.034426 -2.201203
H -2.58926 -0.148817 -2.004408
H -2.20938 1.562473 -2.275538
C -3.955512 -1.186305 0.244844
C -2.664353 -1.868564 0.588191
H -4.219909 -1.370789 -0.80122
H -4.768841 -1.620314 0.840365
C -1.865631 -2.478624 -0.292179
H -2.382066 -1.87541 1.641905
C -0.561225 -3.127816 0.029159
H -2.16524 -2.501056 -1.342365
C 0.614533 -2.385775 -0.610771
H -0.572063 -4.160298 -0.34056
H -0.424207 -3.182422 1.114762
C 1.956608 -3.05944 -0.358819

H 0.644388 -1.358523 -0.218008
H 0.448274 -2.295142 -1.692787
C 3.129183 -2.374672 -1.057279
H 1.898343 -4.098138 -0.704222
H 2.137617 -3.112721 0.721403
C 3.35912 -0.915457 -0.669533
H 2.980461 -2.435342 -2.142406
H 4.041269 -2.944637 -0.845518
H 2.493179 -0.311102 -0.966888
H 4.198347 -0.530769 -1.261165
C 2.868291 1.569357 1.725377
C 1.694451 1.769122 0.768971
H 3.244918 2.55514 2.023509
H 2.494151 1.099635 2.64346
C 2.035882 2.538457 -0.501946
H 0.904809 2.299571 1.320558
H 1.291321 0.781108 0.49429
C 0.847844 2.749284 -1.437634
H 2.821711 2.005551 -1.045034
H 2.460265 3.513159 -0.230966
C -0.190252 3.7419 -0.92055
H 0.390163 1.770049 -1.661669
H 1.208086 3.106473 -2.407225
H -0.573726 3.495822 0.078333
H -1.043675 3.832301 -1.59952
H 0.251833 4.737297 -0.82688
N -1.591774 0.855141 0.32623
H -1.667035 0.995266 1.339211
H -0.854417 1.455381 -0.051286
H -3.125519 2.191371 -0.087052
H -1.350036 -0.136929 0.173888
C -3.966424 0.324208 0.502366
H -4.949593 0.721227 0.21967
O -3.671742 0.642768 1.854258
H -4.354659 0.307423 2.440229
C 4.032599 0.731945 1.1966
H 4.507788 1.233891 0.345173
H 4.793599 0.71323 1.982872
C 3.676922 -0.708774 0.809225
H 2.839314 -1.051911 1.43222
H 4.51627 -1.366757 1.062585

conf_13

1 1
C 1.197478 1.252957 -1.890387
C 2.446263 0.41985 -1.727754
H 1.472827 2.275881 -2.15626
H 0.606913 1.28722 -0.972924
H 0.563019 0.863951 -2.690469
C 2.904138 1.318778 0.650512
C 2.342375 0.174688 1.445979
H 2.154643 2.10774 0.531711

H 3.723792 1.773118 1.222147
C 1.075483 0.082925 1.865037
H 3.051637 -0.591481 1.761219
C 0.531255 -0.973784 2.771365
H 0.371852 0.86758 1.576031
C -0.628584 -1.767425 2.16164
H 0.176913 -0.471551 3.679895
H 1.33358 -1.652136 3.082846
C -0.172253 -2.784532 1.123719
H -1.347098 -1.065337 1.723139
H -1.166606 -2.290052 2.959166
C -1.297355 -3.455708 0.339473
H 0.428987 -3.55468 1.620671
H 0.513969 -2.296869 0.417812
C -2.199244 -2.500333 -0.439266
H -1.914941 -4.037547 1.03288
H -0.857336 -4.184527 -0.352513
H -2.762557 -1.874116 0.260671
H -2.951191 -3.086648 -0.979372
C -3.202744 0.287672 -1.396622
C -2.358553 1.192289 -0.509227
H -3.918947 -0.263043 -0.775797
H -3.809175 0.906201 -2.067845
C -3.170029 2.179384 0.319402
H -1.64564 1.739485 -1.139293
H -1.758056 0.579395 0.178325
C -2.346982 2.910587 1.376597
H -3.987148 1.641854 0.815287
H -3.647 2.909399 -0.346803
C -1.19812 3.737962 0.814398
H -1.955614 2.177202 2.095474
H -3.009301 3.565512 1.95206
H -0.449659 3.112939 0.314049
H -0.685513 4.293534 1.604191
H -1.558395 4.464423 0.078804
N 2.087984 -0.969638 -1.273812
H 2.946907 -1.524211 -1.207364
H 1.411101 -1.42299 -1.889004
H 2.956061 0.290525 -2.686082
H 1.688179 -0.915146 -0.319115
C 3.469827 0.954194 -0.725743
H 3.898714 1.856991 -1.178593
O 4.439662 -0.079766 -0.657468
H 5.217193 0.212412 -0.175478
C -2.384284 -0.6896 -2.240323
H -3.064657 -1.30528 -2.839402
H -1.782916 -0.11969 -2.961335
C -1.465946 -1.608083 -1.434172
H -0.73755 -0.989767 -0.890915
H -0.896523 -2.245614 -2.129163

conf_14

1 1
C -0.247912 1.743931 1.699362
C -0.393683 1.987464 0.215354
H -0.453209 2.668567 2.243089
H 0.758795 1.409786 1.961092
H -0.965813 0.99544 2.044562
C 1.952275 3.062026 0.016385
C 2.673954 1.806304 -0.374605
H 2.045796 3.233646 1.093572
H 2.433129 3.924164 -0.464762
C 3.323233 1.009357 0.479316
H 2.71117 1.574102 -1.440527
C 4.091618 -0.216993 0.115219
H 3.322912 1.274013 1.539119
C 3.754564 -1.434084 1.002565
H 5.160432 0.010102 0.205873
H 3.92703 -0.44644 -0.943172
C 3.478087 -2.709362 0.209179
H 2.888802 -1.204568 1.63533
H 4.577825 -1.619378 1.697659
C 2.209248 -2.667843 -0.639258
H 3.410225 -3.553014 0.905593
H 4.336445 -2.920822 -0.438627
C 0.921186 -2.545006 0.16544
H 2.159825 -3.579108 -1.245636
H 2.26712 -1.842046 -1.364026
H 0.941804 -1.628518 0.775325
H 0.864355 -3.363476 0.893345
C -2.85475 -2.196799 -0.811602
C -4.149307 -1.920343 -0.050388
H -2.96009 -3.140057 -1.358874
H -2.703904 -1.43033 -1.584901
C -4.180166 -0.607396 0.729615
H -4.338029 -2.748356 0.642695
H -4.981713 -1.928003 -0.763262
C -3.979431 0.637959 -0.124844
H -3.432937 -0.624353 1.535137
H -5.147641 -0.528107 1.238153
C -4.099272 1.930125 0.670071
H -4.711544 0.639104 -0.941067
H -2.99952 0.596585 -0.624575
H -5.096415 2.024902 1.10858
H -3.936019 2.812716 0.043988
H -3.384917 1.960538 1.501277
N -0.066391 0.735911 -0.550584
H -0.120119 0.955421 -1.550944
H -0.697253 -0.038311 -0.323955
H -1.433454 2.20994 -0.035182
H 0.904614 0.455921 -0.34622
C 0.471696 3.106144 -0.367387
H 0.042239 4.048256 -0.00389
O 0.274128 2.980093 -1.768717
H 0.665528 3.722368 -2.235613
C -1.617432 -2.284516 0.071807

H -1.516146 -1.362776 0.673438
H -1.75833 -3.068333 0.82607
C -0.33196 -2.555743 -0.700376
H -0.224016 -1.829355 -1.523084
H -0.416953 -3.5237 -1.206865

conf_15

1 1
C 4.127926 -0.76751 0.201539
C 3.192477 -0.13337 1.205313
H 5.118827 -0.31696 0.289886
H 3.780042 -0.625825 -0.824327
H 4.239397 -1.838884 0.38677
C 2.795019 1.952814 -0.270976
C 1.523999 1.484856 -0.917331
H 3.652902 1.734642 -0.915029
H 2.767922 3.04625 -0.174205
C 1.459862 0.84162 -2.087093
H 0.592963 1.751835 -0.414634
C 0.206065 0.449671 -2.795845
H 2.393218 0.607358 -2.60502
C 0.164596 -1.031609 -3.184689
H 0.136379 1.046189 -3.714079
H -0.664888 0.720322 -2.190077
C 0.248291 -1.998374 -2.007102
H 0.980613 -1.243277 -3.885007
H -0.763724 -1.217458 -3.735263
C -0.852052 -1.802667 -0.972113
H 1.240067 -1.911003 -1.532688
H 0.208663 -3.025431 -2.386847
C -0.852627 -2.855217 0.132048
H -1.823369 -1.834766 -1.4817
H -0.791163 -0.794821 -0.546911
H 0.131406 -2.896382 0.6304
H -0.956301 -3.837625 -0.342579
C -1.613475 -0.210671 1.929094
C -2.834612 0.32859 1.197988
H -0.740514 -0.009121 1.291617
H -1.452579 0.377049 2.841605
C -2.692655 1.801211 0.834075
H -3.725086 0.188695 1.82373
H -3.006534 -0.258919 0.290451
C -3.851186 2.360349 0.013292
H -1.758498 1.943164 0.268169
H -2.580467 2.386876 1.754747
C -3.95013 1.774757 -1.389384
H -3.739537 3.447468 -0.059883
H -4.790575 2.189565 0.552849
H -3.023174 1.941917 -1.950233
H -4.76192 2.239206 -1.954498
H -4.142722 0.697935 -1.374423
N 1.805556 -0.689836 1.053071

H 1.179231 -0.197492 1.7024
H 1.754047 -1.693577 1.225862
H 3.492202 -0.383512 2.226462
H 1.458435 -0.49516 0.099831
C 3.066057 1.389711 1.126081
H 4.018786 1.79599 1.488962
O 2.020626 1.681719 2.040197
H 1.927322 2.629834 2.161013
C -1.68331 -1.68973 2.309262
H -0.757995 -1.974664 2.83268
H -2.476794 -1.804874 3.056583
C -1.943652 -2.703367 1.192788
H -2.894764 -2.479186 0.696232
H -2.089264 -3.676642 1.672396

ref_01

1 1
C 4.544762 -2.004563 0.290532
H 3.841683 -2.373688 1.04721
H 5.447431 -1.6876 0.820549
H 4.814852 -2.848192 -0.349013
C 3.951871 -0.858678 -0.515649
H 4.673718 -0.528496 -1.271167
H 3.080371 -1.218226 -1.082569
C 3.553082 0.333223 0.344281
H 2.849821 0.002464 1.128224
H 4.427534 0.693452 0.899186
C 2.943936 1.488327 -0.440246
H 3.69418 1.900325 -1.124878
H 2.152897 1.107187 -1.104022
C 2.404464 2.605199 0.447938
H 1.808614 2.174505 1.267682
H 3.247959 3.092398 0.948417
C 1.58179 3.658331 -0.292336
H 1.401505 4.507153 0.376896
H 2.175785 4.051249 -1.125154
C 0.239105 3.160475 -0.826496
H -0.204205 3.937218 -1.458911
H 0.395674 2.304173 -1.499194
C -0.76243 2.785085 0.259267
H -0.306502 2.094284 0.98565
H -1.005495 3.674995 0.851925
C -2.048954 2.167807 -0.273777
H -2.600367 2.915078 -0.856995
H -1.806362 1.37465 -0.998836
C -2.942344 1.61958 0.831278
H -2.39169 0.852944 1.399926
H -3.14558 2.418847 1.552534
C -4.266999 1.036345 0.352563
H -4.845627 0.709523 1.224011
H -4.860584 1.821015 -0.128376
C -4.128833 -0.137735 -0.621152

H -5.133729 -0.517785 -0.844463
H -3.710112 0.189867 -1.578705
C -3.316559 -1.264242 -0.072508
H -3.585384 -1.60402 0.930572
C -2.337809 -1.901995 -0.720146
H -2.097512 -1.593058 -1.739005
C -1.589402 -3.085941 -0.184183
H -1.951489 -3.345285 0.81582
H -1.798615 -3.962722 -0.811804
C -0.068905 -2.925594 -0.138569
H 0.369211 -3.839009 0.283252
C 0.449964 -1.769367 0.718508
H 1.539128 -1.850591 0.746898
O 0.483251 -2.659574 -1.420475
H 0.316878 -3.390393 -2.020878
N 0.178169 -0.50153 -0.040724
H 0.672282 0.30385 0.34882
H -0.834027 -0.311987 -0.059562
H 0.484977 -0.649399 -1.009026
C -0.117727 -1.677611 2.115342
H 0.372312 -0.885366 2.687222
H 0.056724 -2.61746 2.643551
H -1.193521 -1.48646 2.105207

8E-1-DeoxySO

conf_00

1 1
C -0.263583 -3.534611 -1.264087
C -0.814321 -2.368535 -0.470246
H 0.235312 -4.23395 -0.592499
H 0.464189 -3.197445 -2.006034
H -1.063323 -4.086303 -1.766955
C 1.249096 -2.213007 1.013519
H 1.875285 -2.816816 0.348086
H 0.702381 -2.91687 1.651512
C 4.045543 -0.815286 0.241076
C 3.66291 0.62269 0.071038
H 3.635605 -1.412332 -0.582797
H 5.136116 -0.905049 0.162875
C 3.187541 1.186934 -1.042395
H 3.815588 1.261692 0.943413
C 2.803193 2.625997 -1.186735
H 3.063278 0.565479 -1.933271
C 1.282014 2.809787 -1.273117
H 3.267065 3.031672 -2.092256
H 3.196387 3.204401 -0.343408
H 0.881671 2.116686 -2.026387
H 1.055682 3.816698 -1.639106
C -1.616382 2.166328 1.27107
C -3.114505 1.89208 1.179265
H -1.452235 3.04015 1.911017
H -1.129642 1.326612 1.789341

C -3.458993 0.620837 0.412735
H -3.621694 2.74263 0.70821
H -3.527338 1.811928 2.190987
C -4.924164 0.212661 0.456695
H -2.852892 -0.203404 0.831886
H -3.174648 0.761885 -0.643477
C -5.200617 -1.050996 -0.34461
H -5.537809 1.035458 0.074144
H -5.225188 0.064845 1.499497
H -4.959754 -0.909408 -1.405733
H -6.252004 -1.342189 -0.294786
H -4.616858 -1.899147 0.03599
N -1.580008 -1.46653 -1.406805
H -2.179924 -0.806654 -0.894495
H -2.156556 -1.987052 -2.067053
H -1.520703 -2.707214 0.291486
H -0.861399 -0.917003 -1.904701
C 0.243356 -1.458427 0.163485
H -0.281251 -0.72622 0.798902
O 0.82619 -0.789521 -0.940691
H 1.508131 -0.158205 -0.653107
C -0.926118 2.401314 -0.067073
H -1.108267 1.55661 -0.746922
H -1.369567 3.276709 -0.55863
C 0.578937 2.582784 0.059574
H 1.001947 1.697069 0.55385
H 0.795522 3.421952 0.731961
C 3.619801 -1.375665 1.600597
H 3.951078 -2.416585 1.685728
H 4.151162 -0.82468 2.384656
C 2.122371 -1.290008 1.884077
H 1.951518 -1.525592 2.937714
H 1.797187 -0.247737 1.764307

conf_01

1 1
C 0.861245 0.705389 2.122917
C 1.05149 1.53585 0.875694
H 1.188018 1.276945 2.994109
H 1.433935 -0.223703 2.08878
H -0.192054 0.452792 2.269329
C 3.578316 1.004593 0.548495
H 3.400016 0.26975 1.340043
H 4.522138 1.486772 0.823451
C 3.533585 -2.106139 0.00159
C 2.165414 -2.264065 -0.590374
H 3.446308 -1.896056 1.074013
H 4.04337 -3.075858 -0.062071
C 1.051143 -2.488194 0.116035
H 2.107177 -2.294498 -1.681439
C -0.293747 -2.843178 -0.437352
H 1.131878 -2.509085 1.205706

C -1.455087 -2.173157 0.292392
H -0.418131 -3.930911 -0.345073
H -0.333997 -2.630235 -1.51261
H -1.375303 -1.081266 0.199178
H -1.376733 -2.383938 1.367401
C -4.000578 -0.488522 0.649414
C -4.111548 0.206503 -0.704073
H -3.103824 -0.14406 1.181495
H -4.843412 -0.173116 1.274705
C -3.921842 1.719126 -0.634592
H -5.09196 -0.022558 -1.135665
H -3.376381 -0.207157 -1.408462
C -2.465873 2.135651 -0.454562
H -4.520308 2.131973 0.187032
H -4.300167 2.184116 -1.551591
C -2.264504 3.639353 -0.337063
H -1.90625 1.759259 -1.326962
H -2.066894 1.642796 0.447045
H -2.667551 4.153628 -1.213772
H -1.207617 3.913769 -0.256263
H -2.7805 4.033747 0.542624
N 0.597902 0.775411 -0.340274
H 0.953653 1.277643 -1.162133
H -0.424232 0.750513 -0.392139
H 0.405567 2.417503 0.905763
H 0.95572 -0.199257 -0.341426
C 2.466317 2.055234 0.613144
H 2.673859 2.742668 1.443324
O 2.316955 2.780772 -0.599134
H 3.147267 3.190876 -0.853239
C -3.988608 -2.013778 0.572953
H -3.973292 -2.417042 1.592527
H -4.927421 -2.359739 0.125738
C -2.822205 -2.612564 -0.212503
H -2.912154 -2.363863 -1.276934
H -2.884721 -3.705416 -0.15784
C 4.408888 -1.067289 -0.701792
H 5.371201 -0.997439 -0.183451
H 4.634664 -1.421232 -1.713603
C 3.766276 0.311007 -0.80592
H 4.376477 0.944549 -1.456303
H 2.818615 0.198144 -1.342722

conf_02

1 1
C -0.040987 -3.906033 -0.571235
C 0.131794 -2.446443 -0.228254
H -0.547685 -3.993653 -1.534849
H -0.646101 -4.433503 0.171271
H 0.923785 -4.411679 -0.661715
C -2.303249 -2.226907 0.591938
H -1.970253 -2.549454 1.588888

H -2.624405 -3.128164 0.057427
C -4.239189 0.685506 -0.712632
C -2.906264 1.346429 -0.872154
H -4.762778 1.112867 0.149585
H -4.850118 0.914684 -1.593917
C -2.393785 2.270141 -0.051432
H -2.338042 1.075152 -1.766335
C -1.091739 2.981052 -0.241734
H -2.979411 2.565396 0.821522
C -0.23367 2.99761 1.024005
H -1.30022 4.019483 -0.534109
H -0.546858 2.531425 -1.079542
H -0.042394 1.967851 1.350208
H -0.8074 3.465419 1.832549
C 2.34535 1.691863 -0.025099
C 3.435095 1.204664 -0.97401
H 1.434328 1.099423 -0.18861
H 2.64575 1.513616 1.01651
C 3.579038 -0.312308 -1.034873
H 4.395031 1.652711 -0.688815
H 3.220337 1.57376 -1.983847
C 3.915717 -0.971257 0.297226
H 4.352941 -0.577206 -1.764735
H 2.643933 -0.740895 -1.427874
C 4.063768 -2.484559 0.190938
H 3.155006 -0.70389 1.044377
H 4.847549 -0.548084 0.688603
H 3.19519 -2.954846 -0.296107
H 4.225739 -2.959591 1.164381
H 4.917786 -2.751449 -0.437132
N 0.811361 -2.282687 1.110452
H 0.666276 -1.292263 1.371211
H 1.812047 -2.488822 1.053024
H 0.815147 -1.959749 -0.928746
H 0.38849 -2.875997 1.8249
C -1.140928 -1.595336 -0.164312
H -1.437951 -1.407463 -1.203708
O -0.69948 -0.393551 0.439412
H -1.352564 0.317358 0.297543
C 2.029835 3.173089 -0.196888
H 2.965326 3.744162 -0.172899
H 1.611872 3.336049 -1.198195
C 1.091359 3.739078 0.867023
H 0.889362 4.792524 0.640204
H 1.60705 3.732238 1.834894
C -4.153408 -0.837515 -0.558376
H -5.162629 -1.252991 -0.62022
H -3.618387 -1.251281 -1.422852
C -3.491363 -1.280626 0.758003
H -3.165165 -0.400714 1.324841
H -4.228504 -1.775884 1.395004

conf_03

1 1
C 0.394518 -1.995752 2.640487
C 0.045614 -1.234044 1.37883
H 1.190383 -2.711586 2.433557
H -0.466993 -2.550045 3.020338
H 0.762743 -1.32184 3.419651
C 0.272214 -3.271444 -0.138821
H -0.210886 -3.750119 -0.999397
H 0.246429 -4.001256 0.677153
C 3.239801 -1.263998 -1.696475
C 3.372287 -0.26769 -0.585018
H 3.364845 -0.751795 -2.655036
H 4.040385 -2.011678 -1.632628
C 3.360244 1.053765 -0.752929
H 3.485117 -0.65688 0.428831
C 3.451638 2.078516 0.331817
H 3.283666 1.445962 -1.769517
C 2.321945 3.10832 0.25897
H 4.407606 2.609263 0.244593
H 3.459555 1.586725 1.3126
H 2.356128 3.603765 -0.719458
H 2.492882 3.893226 1.003492
C -1.594051 2.94518 0.392919
C -1.979151 1.901675 -0.653106
H -2.325775 3.758687 0.350078
H -1.703099 2.532723 1.410402
C -3.342305 1.260778 -0.425478
H -1.208733 1.113695 -0.735238
H -1.960454 2.372326 -1.643147
C -3.668103 0.155967 -1.421609
H -4.115979 2.036163 -0.469955
H -3.410617 0.857749 0.598143
C -5.006195 -0.514945 -1.152875
H -2.866529 -0.596435 -1.402196
H -3.657837 0.573572 -2.434965
H -5.028885 -0.975641 -0.159247
H -5.218619 -1.295025 -1.888092
H -5.825191 0.208706 -1.196505
N -1.000714 -0.203727 1.719124
H -1.109851 0.491217 0.971482
H -0.785264 0.291201 2.583949
H 0.906328 -0.68472 0.988452
H -1.894059 -0.705817 1.813487
C -0.579279 -2.077486 0.265596
H -0.728937 -1.42067 -0.603688
O -1.849584 -2.452465 0.786142
H -2.32985 -2.98712 0.149082
C -0.188746 3.515374 0.225167
H -0.090899 3.931472 -0.785122
H -0.059896 4.359121 0.911903
C 0.93448 2.510916 0.456186
H 0.830495 1.656855 -0.226767
H 0.86769 2.11214 1.482827

C 1.880733 -1.972697 -1.676869
H 1.10649 -1.195366 -1.67275
H 1.739617 -2.536205 -2.605574
C 1.716748 -2.916899 -0.485936
H 2.262776 -3.843832 -0.685967
H 2.202532 -2.485944 0.397095

conf_04

1 1
C -0.939893 -1.829859 2.562976
C 0.090729 -1.646559 1.468982
H -1.25391 -2.873233 2.575055
H -1.826664 -1.213726 2.382777
H -0.531786 -1.586448 3.548494
C 0.480677 -1.728704 -1.101422
H 0.679743 -0.65173 -1.162747
H -0.117617 -1.940472 -1.995971
C 3.719286 -0.867144 -0.778353
C 2.98789 0.438484 -0.642362
H 4.024381 -0.989682 -1.823665
H 4.642665 -0.808479 -0.193439
C 3.111133 1.254967 0.411872
H 2.385336 0.762081 -1.490017
C 2.556595 2.639957 0.573507
H 3.761757 0.926403 1.226644
C 1.743095 3.20698 -0.585372
H 3.421052 3.292737 0.750862
H 1.987957 2.706355 1.513176
H 2.290714 3.039187 -1.519113
H 1.692384 4.294356 -0.464607
C -2.090081 2.786028 0.149064
C -2.405701 1.298952 0.068185
H -2.399839 3.265592 -0.787407
H -2.701885 3.237626 0.937812
C -3.878481 0.994794 -0.183049
H -2.130463 0.822157 1.026554
H -1.798613 0.833826 -0.719598
C -4.185414 -0.490097 -0.361363
H -4.206667 1.541771 -1.075787
H -4.465265 1.395001 0.651266
C -3.699318 -1.066647 -1.685571
H -5.267253 -0.639659 -0.291035
H -3.756772 -1.055545 0.478383
H -4.189383 -0.570205 -2.528236
H -3.924625 -2.13422 -1.760563
H -2.619902 -0.942487 -1.827662
N 0.525646 -0.208051 1.443799
H 1.358829 -0.017817 0.842881
H 0.76129 0.104903 2.386117
H 0.983195 -2.235405 1.688177
H -0.237945 0.391225 1.109185
C -0.441851 -2.023009 0.084629

H -1.370059 -1.449462 -0.061366
O -0.738066 -3.394591 0.202815
H -1.250751 -3.676608 -0.558832
C -0.619938 3.104895 0.405038
H -0.512444 4.185332 0.553661
H -0.310617 2.658594 1.364102
C 0.315521 2.681645 -0.725792
H -0.099562 3.059736 -1.667364
H 0.322774 1.589226 -0.840604
C 2.968561 -2.130667 -0.337797
H 3.688859 -2.954586 -0.367039
H 2.704331 -2.032862 0.722815
C 1.768299 -2.551806 -1.186141
H 2.090995 -2.565644 -2.233016
H 1.514114 -3.588019 -0.941182

conf_05

1 1
C -0.345602 -3.403111 -1.526397
C -0.885479 -2.385787 -0.543317
H 0.193437 -4.18418 -0.98929
H 0.344228 -2.939094 -2.235433
H -1.155172 -3.891621 -2.076756
C 1.257271 -2.387545 0.850093
H 1.84469 -2.885384 0.071452
H 0.772347 -3.179348 1.432169
C 3.969552 -0.884701 0.043186
C 3.600074 0.56701 0.034499
H 3.488117 -1.397052 -0.798852
H 5.049151 -0.971464 -0.131983
C 3.027129 1.223073 -0.978697
H 3.866386 1.133215 0.929391
C 2.703277 2.683924 -0.998971
H 2.789202 0.668008 -1.889769
C 1.20144 2.972997 -0.871693
H 3.071352 3.109172 -1.939052
H 3.239572 3.195511 -0.192016
H 0.649074 2.329958 -1.569564
H 1.008309 4.003208 -1.19089
C -1.679352 1.888572 0.174866
C -3.1741 2.127839 0.351478
H -1.380513 0.968687 0.697281
H -1.464091 1.733628 -0.892338
C -4.049476 0.946737 -0.058637
H -3.461224 3.009303 -0.231623
H -3.381148 2.378362 1.399431
C -3.984236 -0.246948 0.893361
H -3.792401 0.64136 -1.087953
H -5.094479 1.268001 -0.123434
C -4.815679 -1.437731 0.435749
H -4.33102 0.078588 1.880041
H -2.940753 -0.555494 1.076047

H -5.868473 -1.160096 0.338902
H -4.757685 -2.269651 1.141978
H -4.505597 -1.812576 -0.548962
N -1.701217 -1.367579 -1.303166
H -2.329375 -0.840291 -0.687927
H -2.252016 -1.787671 -2.05064
H -1.555312 -2.853364 0.182088
H -1.009474 -0.702385 -1.688976
C 0.181613 -1.554942 0.179962
H -0.327613 -0.95402 0.951194
O 0.68467 -0.699224 -0.829209
H 1.382772 -0.109921 -0.493967
C -0.816242 3.023517 0.7126
H -1.101004 3.962625 0.221618
H -1.048262 3.156275 1.775604
C 0.684375 2.798241 0.551515
H 0.944433 1.798111 0.92735
H 1.221476 3.502458 1.197241
C 3.649236 -1.576189 1.370437
H 3.998646 -2.613996 1.331876
H 4.227739 -1.092059 2.165136
C 2.174281 -1.549024 1.76114
H 2.076024 -1.900315 2.791511
H 1.831322 -0.505227 1.777903

conf_06

1 1
C -0.371464 -3.204455 -1.51485
C -0.715787 -2.254885 -0.386782
H 0.224828 -4.031866 -1.128709
H 0.209848 -2.700144 -2.290346
H -1.272382 -3.636151 -1.960518
C 1.577469 -2.434201 0.710547
H 2.074964 -2.877592 -0.158727
H 1.106846 -3.260359 1.255993
C 4.172464 -0.728418 -0.158867
C 3.736582 0.682191 0.10549
H 3.638482 -1.130652 -1.028593
H 5.232791 -0.719485 -0.440761
C 3.170814 1.506119 -0.781163
H 3.954883 1.072701 1.101538
C 2.813844 2.938759 -0.537552
H 2.981434 1.135552 -1.792649
C 1.327793 3.224982 -0.773144
H 3.405895 3.56235 -1.218172
H 3.099984 3.228136 0.479895
H 1.043571 2.81511 -1.751936
H 1.168209 4.305787 -0.841649
C -1.89717 1.753382 0.865493
C -3.289811 1.419921 0.339723
H -1.981861 2.328622 1.794321
H -1.378943 0.826562 1.165587

C -4.041328 0.407153 1.198998
H -3.211641 1.061307 -0.698819
H -3.884093 2.336794 0.260875
C -5.349202 -0.091353 0.591358
H -4.234438 0.853007 2.180464
H -3.395957 -0.464569 1.414462
C -5.160306 -0.980675 -0.631709
H -5.975674 0.767932 0.326688
H -5.90527 -0.648951 1.351229
H -4.673788 -0.448805 -1.457854
H -6.117709 -1.34016 -1.015234
H -4.568424 -1.871083 -0.37806
N -1.606371 -1.164911 -0.928681
H -2.077257 -0.639481 -0.185825
H -2.32203 -1.515789 -1.565109
H -1.277163 -2.762845 0.400865
H -0.973247 -0.509577 -1.415199
C 0.478806 -1.509943 0.216926
H 0.106298 -0.925151 1.074513
O 0.889269 -0.628335 -0.812269
H 1.598093 -0.035362 -0.506712
C -1.0251 2.49584 -0.136744
H -1.043156 1.960325 -1.098831
H -1.456926 3.481473 -0.347456
C 0.423808 2.63837 0.305382
H 0.800415 1.648596 0.596151
H 0.481879 3.252783 1.21181
C 4.016194 -1.639918 1.058808
H 4.375145 -2.644314 0.808194
H 4.676863 -1.273842 1.852835
C 2.600702 -1.730256 1.621798
H 2.644223 -2.256387 2.578963
H 2.245898 -0.719088 1.863415

conf_07

1 1
C 0.175415 -3.503923 -1.517638
C -0.49618 -2.413637 -0.709323
H 0.669917 -4.207037 -0.846671
H 0.929417 -3.088481 -2.190456
H -0.554293 -4.074208 -2.100003
C 1.445257 -2.218211 0.929949
H 2.15596 -2.752115 0.290031
H 0.893477 -2.982539 1.489269
C 4.148887 -0.53006 0.417692
C 3.631352 0.871607 0.308912
H 3.854576 -1.108266 -0.46667
H 5.245648 -0.505767 0.408303
C 3.196105 1.4665 -0.80521
H 3.639821 1.456032 1.231369
C 2.672044 2.865259 -0.893122
H 3.216519 0.90316 -1.742024

C 1.15503 2.898497 -1.123292
H 3.172402 3.383672 -1.718124
H 2.917824 3.415433 0.021883
H 0.906038 2.226988 -1.957035
H 0.85652 3.901863 -1.444562
C -1.888494 1.80488 1.07359
C -3.362078 1.486904 0.840447
H -1.814675 2.613512 1.809144
H -1.402405 0.937299 1.544817
C -3.609512 0.274844 -0.051879
H -3.863001 2.357099 0.398393
H -3.840649 1.318651 1.809794
C -5.076463 -0.121469 -0.21206
H -3.059183 -0.583926 0.377374
H -3.20347 0.49308 -1.052588
C -5.729154 -0.609867 1.073428
H -5.155318 -0.905209 -0.975426
H -5.630067 0.737741 -0.607977
H -5.182929 -1.458889 1.498959
H -6.754596 -0.937661 0.888308
H -5.772377 0.172748 1.834674
N -1.243498 -1.502829 -1.653563
H -1.940247 -0.926411 -1.163052
H -1.707779 -2.013098 -2.403976
H -1.232067 -2.830202 -0.017562
H -0.521766 -0.869571 -2.038113
C 0.461294 -1.480698 0.040118
H -0.148566 -0.809463 0.66657
O 1.079501 -0.729545 -0.989339
H 1.673816 -0.050223 -0.624331
C -1.109047 2.210794 -0.171273
H -1.159378 1.421532 -0.9357
H -1.583201 3.089164 -0.6274
C 0.356821 2.503244 0.112777
H 0.812511 1.619929 0.581334
H 0.432809 3.300247 0.862623
C 3.702187 -1.221344 1.708088
H 4.12592 -2.231104 1.744315
H 4.135925 -0.682171 2.55778
C 2.190912 -1.294223 1.911509
H 1.996129 -1.630311 2.93325
H 1.772947 -0.279922 1.856858

conf_08

1 1
C 0.264397 -3.48784 -1.654288
C -0.492868 -2.598413 -0.690746
H 0.892871 -4.183062 -1.096803
H 0.908136 -2.898649 -2.311722
H -0.419269 -4.088763 -2.261284
C 1.526072 -2.289154 0.841669
H 2.233617 -2.670492 0.09797

H 1.138328 -3.160413 1.381483
C 4.060126 -0.432889 0.25129
C 3.502614 0.956201 0.202855
H 3.718218 -1.001977 -0.622009
H 5.152037 -0.374995 0.16143
C 2.912381 1.527304 -0.850811
H 3.634731 1.555385 1.106126
C 2.403898 2.933616 -0.906615
H 2.807471 0.943428 -1.76889
C 0.872641 3.026402 -0.882918
H 2.776563 3.40246 -1.823948
H 2.814202 3.510817 -0.070526
H 0.456226 2.320708 -1.613806
H 0.570955 4.024507 -1.219564
C -1.908026 1.573025 -0.031951
C -3.429873 1.640405 -0.014395
H -1.560842 0.703329 0.54425
H -1.567924 1.423478 -1.066981
C -4.11143 0.361231 -0.494919
H -3.753007 2.473902 -0.64717
H -3.773159 1.88565 0.99711
C -3.994209 -0.832445 0.458699
H -3.715213 0.105916 -1.491391
H -5.177042 0.552041 -0.661598
C -4.802686 -0.66966 1.739919
H -2.943397 -0.999233 0.753728
H -4.331895 -1.742584 -0.055235
H -4.463267 0.188425 2.324913
H -4.723327 -1.555074 2.374926
H -5.860521 -0.518202 1.507826
N -1.42281 -1.70989 -1.482536
H -2.164261 -1.310549 -0.896154
H -1.852443 -2.196107 -2.268654
H -1.1154 -3.188132 -0.013854
H -0.832997 -0.929816 -1.818067
C 0.377844 -1.622733 0.109162
H -0.269608 -1.123951 0.849315
O 0.803257 -0.679685 -0.85702
H 1.397326 -0.010224 -0.476244
C -1.24007 2.81161 0.552638
H -1.605172 3.703839 0.028406
H -1.565895 2.919046 1.593657
C 0.284789 2.781404 0.502063
H 0.643947 1.821802 0.901171
H 0.67992 3.546824 1.179826
C 3.730264 -1.164241 1.554821
H 4.217652 -2.145608 1.554055
H 4.171168 -0.608096 2.389556
C 2.237391 -1.335062 1.81974
H 2.09623 -1.695289 2.842028
H 1.759127 -0.346191 1.792747

conf_09

1 1
C 0.144615 -3.140662 1.796826
C 0.589467 -2.212867 0.685909
H -0.396243 -3.98729 1.372607
H -0.521281 -2.628318 2.495263
H 1.000835 -3.546647 2.343507
C -1.578756 -2.463024 -0.630596
H -2.155539 -2.890853 0.196469
H -1.041438 -3.295312 -1.099942
C -4.249428 -0.731102 -0.084463
C -3.779388 0.666378 -0.360108
H -3.810077 -1.098245 0.851149
H -5.332964 -0.708083 0.086632
C -3.306259 1.525684 0.547194
H -3.885788 1.014522 -1.389524
C -2.907874 2.943691 0.283614
H -3.230792 1.19781 1.587962
C -1.446429 3.222199 0.648599
H -3.552684 3.600454 0.879673
H -3.090879 3.195186 -0.767106
H -1.260781 2.84528 1.663668
H -1.278728 4.303029 0.693584
C 1.902028 1.679317 -0.640404
C 3.264082 1.408773 -0.008865
H 2.041078 2.203146 -1.592803
H 1.421793 0.72714 -0.924413
C 4.081552 0.354217 -0.744447
H 3.140058 1.114227 1.04757
H 3.835037 2.342339 0.041077
C 5.43701 0.071649 -0.111441
H 4.219292 0.665729 -1.787064
H 3.513451 -0.592427 -0.812475
C 6.226757 -0.999664 -0.847594
H 5.296852 -0.224084 0.937419
H 6.014452 1.002817 -0.082313
H 5.688241 -1.953421 -0.864594
H 7.195705 -1.176223 -0.375048
H 6.413232 -0.708388 -1.885516
N 1.398421 -1.09003 1.290949
H 1.9662 -0.594601 0.596396
H 2.009014 -1.407015 2.043232
H 1.238971 -2.728578 -0.02502
H 0.697364 -0.420328 1.652647
C -0.549121 -1.505815 -0.057127
H -0.101181 -0.938724 -0.889667
O -1.072145 -0.602866 0.900342
H -1.741158 -0.016697 0.503685
C 0.950463 2.462663 0.252312
H 0.884939 1.971822 1.23619
H 1.367281 3.456301 0.455707
C -0.453995 2.588152 -0.319485
H -0.809439 1.588305 -0.602125
H -0.428148 3.164374 -1.252131

C -3.976295 -1.693565 -1.240715
H -4.359812 -2.686868 -0.982336
H -4.55687 -1.364287 -2.109943
C -2.514198 -1.806777 -1.663593
H -2.471359 -2.379974 -2.593383
H -2.135806 -0.80846 -1.922099

conf_10

1 1
C 0.579748 -3.587599 1.248367
C 0.228214 -2.279247 0.582921
H 1.099292 -3.387147 2.188021
H 1.23986 -4.200434 0.628437
H -0.315863 -4.166666 1.487667
C 2.598967 -1.996035 -0.377741
H 2.25584 -2.644484 -1.197372
H 3.07453 -2.654815 0.358042
C 3.857142 0.823845 0.870107
C 3.078401 1.821647 0.069951
H 4.659611 1.360581 1.392537
H 3.226889 0.410365 1.665175
C 1.889361 2.330389 0.410812
H 3.564419 2.200393 -0.831531
C 1.167121 3.405905 -0.338333
H 1.424173 1.979499 1.334621
C -0.203755 2.996733 -0.886604
H 1.79817 3.757484 -1.160843
H 1.030339 4.261468 0.336378
H -0.076906 2.190807 -1.622588
H -0.614599 3.846826 -1.443174
C -3.647316 1.88909 0.60997
C -3.334978 0.611869 1.390983
H -4.612125 1.782376 0.102036
H -3.782233 2.70564 1.328627
C -3.049987 -0.609583 0.520794
H -4.177237 0.387257 2.055412
H -2.475162 0.785617 2.048262
C -4.211351 -1.054098 -0.358946
H -2.772548 -1.44737 1.179891
H -2.175303 -0.391173 -0.110166
C -3.883906 -2.280234 -1.198395
H -4.510509 -0.236466 -1.022463
H -5.079197 -1.265983 0.2754
H -3.101301 -2.061179 -1.937258
H -4.748251 -2.633719 -1.765147
H -3.566196 -3.12322 -0.566147
N -0.483913 -2.51057 -0.728203
H -0.416494 -1.610314 -1.234656
H -1.471824 -2.746571 -0.604333
H -0.495241 -1.719742 1.17953
H -0.035622 -3.239167 -1.284221
C 1.388978 -1.327207 0.267366

H 1.673578 -0.867256 1.219023
O 0.799478 -0.360136 -0.583869
H 1.293919 0.478088 -0.530023
C -2.591672 2.296321 -0.415177
H -2.509331 1.529138 -1.19779
H -2.938266 3.19948 -0.930508
C -1.210752 2.563163 0.171158
H -0.845254 1.663403 0.680904
H -1.289717 3.341469 0.942183
C 4.520001 -0.278156 0.034379
H 5.328743 0.18255 -0.543878
H 5.008818 -0.988799 0.71086
C 3.63902 -1.036789 -0.957282
H 3.142479 -0.331533 -1.634009
H 4.300681 -1.631408 -1.594511

conf_11

1 1
C -0.272475 -3.259624 1.24484
C -0.267653 -1.967587 0.464969
H 0.222329 -3.104013 2.206039
H 0.261889 -4.057104 0.7213
H -1.291659 -3.596401 1.451866
C 2.159378 -2.310813 -0.325623
H 1.72726 -2.930983 -1.124813
H 2.42651 -2.997324 0.486112
C 3.978029 0.128691 0.948508
C 3.433528 1.271296 0.148174
H 4.862043 0.488018 1.49119
H 3.261404 -0.153707 1.727936
C 2.354285 1.991188 0.473123
H 4.011776 1.566654 -0.729703
C 1.867134 3.204459 -0.254634
H 1.804954 1.71275 1.37527
C 0.455659 3.078467 -0.838069
H 2.571882 3.456468 -1.053468
H 1.877412 4.049017 0.447142
H 0.452322 2.319566 -1.632345
H 0.206892 4.025157 -1.331056
C -3.148015 2.498932 0.573459
C -3.251117 1.033286 0.992219
H -4.108985 2.798318 0.138574
H -3.019915 3.115888 1.470576
C -3.645981 0.094183 -0.141957
H -4.001213 0.951048 1.787472
H -2.305611 0.700273 1.4433
C -3.967155 -1.322104 0.321376
H -2.855354 0.074954 -0.907431
H -4.526609 0.501159 -0.654653
C -4.407514 -2.249389 -0.806162
H -4.763544 -1.280058 1.071874
H -3.108902 -1.744387 0.869671

H -5.338612 -1.892952 -1.254165
H -4.580683 -3.270598 -0.456892
H -3.687913 -2.292499 -1.638257
N -0.935496 -2.144783 -0.876196
H -0.68942 -1.300639 -1.419624
H -1.95287 -2.221357 -0.791713
H -0.877735 -1.209137 0.962268
H -0.583234 -2.965815 -1.369441
C 1.09804 -1.335253 0.176049
H 1.42425 -0.871006 1.111942
O 0.805738 -0.335545 -0.783833
H 1.439528 0.396584 -0.683581
C -2.030068 2.829152 -0.413085
H -2.093368 2.176384 -1.293927
H -2.189448 3.845084 -0.792055
C -0.628941 2.749978 0.180724
H -0.455463 1.748996 0.594492
H -0.559623 3.447524 1.026339
C 4.419135 -1.083827 0.120704
H 5.315624 -0.800593 -0.442272
H 4.73942 -1.876573 0.806646
C 3.421472 -1.653593 -0.88643
H 3.132462 -0.88159 -1.60865
H 3.948334 -2.413908 -1.471031

conf_12

1 1
C -0.190185 -1.498529 2.142747
C 0.091187 -2.075155 0.775174
H -0.32828 -2.312284 2.857921
H -1.094816 -0.885311 2.1513
H 0.646758 -0.890727 2.495647
C -2.424587 -2.565935 0.316091
H -2.71173 -2.544011 1.373711
H -3.047815 -3.347856 -0.136419
C -4.368194 0.594736 -0.904494
C -3.68766 1.608075 -0.039898
H -5.438188 0.819228 -0.966663
H -3.972997 0.651942 -1.925668
C -2.586006 2.286575 -0.362959
H -4.128237 1.77272 0.945422
C -1.896035 3.258447 0.541302
H -2.1606 2.142521 -1.358333
C -0.523157 2.787712 1.03215
H -2.534466 3.45407 1.408499
H -1.770937 4.217923 0.023337
H -0.642803 1.835445 1.570923
H -0.156914 3.5036 1.777079
C 3.023928 2.222054 -0.539416
C 3.001744 0.921611 -1.342723
H 3.998996 2.324788 -0.052875
H 2.946588 3.069767 -1.229062

C 3.45025 -0.313201 -0.555976
H 3.655506 1.020354 -2.215719
H 1.996801 0.774224 -1.766054
C 4.95223 -0.37889 -0.295541
H 3.162264 -1.227145 -1.096098
H 2.955908 -0.350927 0.432725
C 5.355702 -1.610621 0.499479
H 5.273568 0.52328 0.235196
H 5.471887 -0.36902 -1.260424
H 4.877523 -1.622416 1.484952
H 6.435509 -1.64296 0.660501
H 5.073406 -2.531039 -0.022141
N 0.314649 -0.980448 -0.236162
H 0.233775 -1.416299 -1.165367
H 1.245643 -0.562158 -0.140732
H 1.035605 -2.626551 0.780494
H -0.389228 -0.241771 -0.165483
C -0.974342 -3.026096 0.214245
H -0.860974 -3.962715 0.774865
O -0.557598 -3.215184 -1.133496
H -1.120733 -3.855187 -1.575757
C 1.920683 2.346458 0.510331
H 1.884029 1.442844 1.139246
H 2.182395 3.152889 1.203962
C 0.535554 2.645175 -0.055136
H 0.236175 1.879792 -0.787136
H 0.585137 3.577594 -0.631361
C -4.18944 -0.832203 -0.366905
H -4.609965 -0.900357 0.64408
H -4.754639 -1.534013 -0.989258
C -2.720447 -1.223866 -0.336004
H -2.332561 -1.211422 -1.363587
H -2.21071 -0.422291 0.214582

conf_13

1 1
C 0.68237 -1.510526 -2.119
C 0.25521 -1.765047 -0.693128
H 0.768099 -2.461113 -2.6497
H 1.648268 -1.003836 -2.175279
H -0.057932 -0.905104 -2.648327
C 2.63074 -2.40663 0.165429
H 2.95623 -2.170352 -0.853123
H 3.158605 -3.333096 0.414933
C 4.024489 0.38803 -0.500699
C 3.09204 1.510716 -0.166283
H 3.657113 -0.159737 -1.377305
H 4.974785 0.835893 -0.818267
C 2.095022 1.963346 -0.935198
H 3.324207 2.062335 0.747107
C 1.337871 3.238121 -0.715072
H 1.886863 1.443945 -1.875429

C -0.171331 3.084603 -0.498017
H 1.503428 3.863211 -1.600669
H 1.774525 3.783274 0.128976
H -0.566467 2.342099 -1.202804
H -0.666486 4.023442 -0.767165
C -2.68425 1.280659 1.04193
C -2.91888 0.861705 -0.408065
H -3.652624 1.279495 1.551195
H -2.097375 0.510999 1.569833
C -3.468479 -0.554571 -0.567925
H -2.001504 0.967964 -1.009573
H -3.608506 1.574766 -0.876029
C -4.87446 -0.7528 -0.0125
H -2.798115 -1.275367 -0.071672
H -3.4712 -0.82089 -1.63233
C -5.406575 -2.155404 -0.264208
H -5.542341 -0.014022 -0.4721
H -4.88435 -0.547963 1.063472
H -5.446201 -2.378096 -1.335128
H -6.416003 -2.275375 0.135776
H -4.77225 -2.911423 0.210431
N 0.124633 -0.474974 0.064515
H 0.053516 -0.714884 1.060586
H -0.72086 0.036851 -0.205217
H -0.750476 -2.193337 -0.673973
H 0.929837 0.162116 -0.071715
C 1.135635 -2.716673 0.121464
H 0.995779 -3.699274 -0.347967
O 0.525927 -2.696186 1.404757
H 0.974233 -3.298926 2.002962
C -2.033455 2.645832 1.263096
H -2.581589 3.407952 0.695242
H -2.175305 2.905529 2.316719
C -0.537757 2.761239 0.949089
H -0.013375 1.858084 1.297955
H -0.121576 3.566275 1.565344
C 4.310222 -0.555142 0.668742
H 5.095026 -1.263217 0.381585
H 4.717381 0.029371 1.50081
C 3.077835 -1.314216 1.147513
H 3.284 -1.767667 2.121035
H 2.285306 -0.585078 1.347135

ref_01

1 1
C 4.342303 -1.825606 -0.913486
H 5.284624 -1.984467 -0.382043
H 4.439174 -2.280216 -1.902176
H 3.573806 -2.387606 -0.36673
C 4.012552 -0.342598 -1.005006
H 3.091244 -0.197455 -1.589258
H 4.784847 0.168029 -1.590039

C 3.913442 0.340877 0.357858
H 4.895727 0.285065 0.838867
H 3.251728 -0.232922 1.03324
C 3.445025 1.793087 0.30635
H 4.041512 2.338276 -0.435045
H 3.64446 2.274276 1.269824
C 1.964319 1.94696 -0.01849
H 1.380833 1.443061 0.767541
H 1.733963 1.447088 -0.972415
C 1.492607 3.390963 -0.134583
H 2.069605 3.882595 -0.925605
H 1.727418 3.926812 0.793666
C 0.002181 3.515792 -0.43826
H -0.236313 2.888738 -1.308157
H -0.219905 4.544163 -0.744042
C -0.901374 3.158809 0.73758
H -0.708611 3.86666 1.552221
H -0.649694 2.165506 1.134323
C -2.396516 3.179311 0.403791
H -2.637985 4.140229 -0.06949
H -2.97802 3.134359 1.33008
C -2.821385 2.05955 -0.492612
H -2.389258 2.032513 -1.494433
C -3.698764 1.114377 -0.156184
H -4.152601 1.155984 0.836334
C -4.132373 -0.024055 -1.024029
H -3.682364 0.07071 -2.019519
H -5.218244 0.018694 -1.16628
C -3.77493 -1.38363 -0.415151
H -4.220241 -2.187477 -1.011113
H -4.218241 -1.457451 0.585953
C -2.269512 -1.595055 -0.315118
H -1.856794 -0.681617 0.138811
H -1.856179 -1.678113 -1.329392
C -1.890134 -2.817488 0.512012
H -2.464468 -3.685263 0.162797
H -2.198537 -2.671133 1.553669
C -0.42014 -3.219895 0.496453
H -0.282811 -4.061829 1.187053
C 0.588515 -2.15286 0.936968
H 1.554826 -2.651446 1.052288
O 0.035211 -3.580184 -0.802679
H -0.473242 -4.317762 -1.148454
N 0.795678 -1.218138 -0.225278
H 0.003063 -0.581358 -0.336245
H 0.857989 -1.801265 -1.07043
H 1.64625 -0.658225 -0.122118
C 0.2372 -1.387215 2.191146
H 1.04347 -0.707797 2.478268
H 0.092555 -2.089833 3.014643
H -0.683118 -0.809029 2.076349

ref_02

1 1
C 5.087114 -2.470981 -0.437813
H 5.184816 -2.701221 -1.503166
H 4.331819 -3.146136 -0.02147
H 6.040328 -2.70709 0.040898
C 4.703568 -1.014602 -0.223054
H 5.485861 -0.361414 -0.628877
H 4.658974 -0.80529 0.85178
C 3.375787 -0.658116 -0.881572
H 2.588542 -1.291863 -0.440273
H 3.419898 -0.940641 -1.940218
C 2.987711 0.815632 -0.781303
H 2.0879 0.993761 -1.384598
H 3.76107 1.425006 -1.263729
C 2.803541 1.322472 0.649868
H 3.772529 1.25967 1.153639
H 2.163248 0.632352 1.230255
C 2.26968 2.749487 0.803537
H 2.765716 3.404284 0.076639
H 2.57519 3.115837 1.788453
C 0.752097 2.922786 0.70082
H 0.480487 3.875354 1.170912
H 0.267157 2.157148 1.324978
C 0.157823 2.928616 -0.705168
H 0.378832 2.001491 -1.248791
H 0.648159 3.716753 -1.286914
C -1.354549 3.166698 -0.73908
H -1.685426 3.241954 -1.779571
H -1.56293 4.140702 -0.277084
C -2.181426 2.132494 -0.035234
H -2.074534 2.067983 1.049755
C -3.113979 1.376526 -0.626444
H -3.251994 1.484135 -1.704883
C -4.079957 0.463166 0.062614
H -5.040036 0.988764 0.141766
H -3.760826 0.285514 1.097006
C -4.316271 -0.849614 -0.686714
H -4.723564 -0.617877 -1.676813
H -5.086936 -1.431369 -0.169786
C -3.054466 -1.689149 -0.854775
H -2.278345 -1.055464 -1.297525
H -3.232084 -2.46656 -1.603114
C -2.58768 -2.343869 0.452348
H -3.088642 -3.312321 0.552982
H -2.926904 -1.766552 1.318821
C -1.083594 -2.574711 0.597481
H -0.919476 -3.333731 1.373657
C -0.242642 -1.37548 1.04296
H 0.775139 -1.75274 1.177859
O -0.460008 -2.97186 -0.615094
H -0.874101 -3.763366 -0.967349
N -0.13294 -0.413612 -0.105919
H 0.682475 0.195454 0.000589

H -0.96655 0.195645 -0.203391
H -0.017738 -0.972064 -0.960312
C -0.706749 -0.667983 2.29428
H -0.808062 -1.391782 3.105832
H -1.673468 -0.179014 2.15433
H 0.018065 0.084624 2.613401

12E-1-DeoxySO

conf_00

1 1
C 0.793227 -1.944809 1.847405
C 0.547731 -2.262888 0.390887
H 0.450012 -2.777257 2.465572
H 0.257333 -1.046409 2.163323
H 1.858079 -1.803924 2.047792
C -1.939513 -1.616976 0.559273
C -3.337904 -1.853743 -0.0036
H -1.633539 -0.585342 0.351232
H -1.967374 -1.721209 1.650046
C -4.407943 -0.957706 0.624338
H -3.620858 -2.902681 0.150504
H -3.33241 -1.687045 -1.088104
C -4.087004 0.53531 0.592541
H -4.560642 -1.264165 1.665694
H -5.357566 -1.143414 0.110651
C -3.877122 1.109865 -0.80484
H -3.201889 0.720002 1.212593
H -4.900202 1.080269 1.084908
C -3.3781 2.555539 -0.819488
H -4.820834 1.055867 -1.359634
H -3.178138 0.480213 -1.370028
C -2.048704 2.78035 -0.099447
H -4.132661 3.201579 -0.356667
H -3.290825 2.890751 -1.859679
C -0.874275 1.987941 -0.684899
H -2.158001 2.524333 0.960823
H -1.796981 3.8454 -0.116926
C 0.266378 1.87725 0.279681
H -0.533998 2.443554 -1.621003
H -1.233667 0.984167 -0.953022
C 1.519029 2.293811 0.066438
H 0.013353 1.501224 1.276657
C 2.620841 2.289932 1.078193
H 1.760122 2.728306 -0.906307
C 3.969386 1.817166 0.534138
H 2.326602 1.685142 1.945188
H 2.740149 3.315446 1.450923
C 3.936688 0.402373 -0.024439
H 4.712898 1.873453 1.336009
H 4.311404 2.504939 -0.248413
C 5.288512 -0.157487 -0.440568

H 3.273023 0.394806 -0.904882
H 3.496868 -0.265172 0.735797
C 5.180875 -1.564359 -1.009488
H 5.960156 -0.156202 0.425101
H 5.742697 0.509907 -1.181444
H 4.75606 -2.258819 -0.274474
H 6.156843 -1.960871 -1.297697
H 4.549449 -1.584231 -1.905958
N 0.999864 -1.128591 -0.489484
H 0.575511 -1.283238 -1.414397
H 2.017697 -1.097953 -0.575528
H 1.15858 -3.113177 0.075479
H 0.725091 -0.18841 -0.15052
C -0.901776 -2.5846 0.010124
H -1.10886 -3.594934 0.387573
O -0.86373 -2.601177 -1.412023
H -1.724635 -2.837174 -1.766099

conf_01

1 1
C 0.859438 -1.33712 1.842546
C 0.746897 -1.831077 0.419487
H 0.589861 -2.14348 2.528349
H 0.191085 -0.494127 2.035128
H 1.881644 -1.03378 2.078451
C -1.807764 -1.566613 0.372046
C -3.148152 -2.169069 -0.039069
H -1.693438 -0.581582 -0.09627
H -1.823671 -1.400577 1.454616
C -4.299385 -1.187048 0.168899
H -3.32105 -3.085774 0.536271
H -3.139405 -2.461561 -1.098765
C -4.312283 -0.030373 -0.845885
H -4.241619 -0.795395 1.191883
H -5.245792 -1.731864 0.116902
C -4.513131 1.350166 -0.222236
H -5.103796 -0.205061 -1.580524
H -3.381426 -0.033518 -1.427998
C -3.340188 1.833196 0.628229
H -5.418708 1.331189 0.394788
H -4.704645 2.082084 -1.015601
C -2.082903 2.158067 -0.16839
H -3.100016 1.081003 1.392205
H -3.642299 2.727767 1.184431
C -0.901217 2.535693 0.722061
H -2.290568 2.981183 -0.862892
H -1.803497 1.308225 -0.805837
C 0.371322 2.742386 -0.035625
H -0.756263 1.782315 1.508365
H -1.147533 3.4663 1.250966
C 1.58937 2.348313 0.350155
H 0.286137 3.317143 -0.959955

C 2.857575 2.67537 -0.380524
H 1.684302 1.822091 1.30324
C 3.598248 1.467333 -0.960252
H 3.531257 3.201273 0.30822
H 2.632046 3.378102 -1.188226
C 4.091185 0.467037 0.080485
H 4.456105 1.820788 -1.541797
H 2.947044 0.974074 -1.699945
C 4.638295 -0.824633 -0.517571
H 3.293021 0.229467 0.800105
H 4.870704 0.939449 0.68917
C 5.091273 -1.827205 0.532532
H 5.469965 -0.583552 -1.188665
H 3.885464 -1.294819 -1.1742
H 5.884048 -1.40665 1.157376
H 5.479469 -2.740125 0.075344
H 4.269084 -2.112216 1.199221
N 1.049184 -0.730844 -0.566416
H 0.629192 -1.018212 -1.462513
H 2.056702 -0.611995 -0.69419
H 1.504291 -2.593144 0.216795
H 0.68838 0.196926 -0.295131
C -0.606932 -2.429617 0.02629
H -0.682622 -3.397799 0.539073
O -0.473074 -2.630878 -1.375726
H -1.283022 -3.000039 -1.736738

conf_02

1 1
C -0.87049 -2.08688 -1.333811
C -0.384248 -2.328244 0.076453
H -0.583785 -2.92986 -1.966421
H -0.438421 -1.179265 -1.762169
H -1.959359 -2.004333 -1.370287
C 2.018052 -1.613113 -0.523028
C 3.49771 -1.782387 -0.189745
H 1.710809 -0.582937 -0.312407
H 1.870984 -1.769553 -1.597848
C 4.416683 -0.870246 -1.005671
H 3.792641 -2.82521 -0.361305
H 3.66269 -1.582374 0.876473
C 4.068393 0.61552 -0.934871
H 4.393095 -1.18976 -2.054036
H 5.446088 -1.024906 -0.66424
C 4.108946 1.210309 0.469116
H 3.078075 0.768492 -1.379965
H 4.760128 1.169897 -1.579046
C 3.592424 2.647736 0.56008
H 5.139332 1.181384 0.841569
H 3.539103 0.577452 1.161397
C 2.171236 2.84993 0.033529
H 4.261646 3.308794 -0.001879

H 3.648636 2.978137 1.603846
C 1.115303 1.984205 0.73079
H 2.149815 2.638933 -1.041672
H 1.890155 3.903192 0.129145
C -0.103805 1.778297 -0.114147
H 0.838137 2.41557 1.698669
H 1.568059 1.008641 0.9565
C -1.372109 1.957149 0.272369
H 0.081512 1.507222 -1.157571
C -2.576362 1.805859 -0.599336
H -1.560504 2.27375 1.300994
C -3.578391 0.768081 -0.091057
H -2.27209 1.566164 -1.624505
H -3.087246 2.776372 -0.64916
C -4.878254 0.754583 -0.885833
H -3.788777 0.954554 0.969288
H -3.131117 -0.236742 -0.154851
C -5.838725 -0.361896 -0.487715
H -4.64022 0.663963 -1.952688
H -5.375818 1.725241 -0.765981
C -6.345462 -0.267512 0.945303
H -5.350714 -1.333638 -0.646007
H -6.693023 -0.344742 -1.172122
H -5.540776 -0.378908 1.679168
H -7.082566 -1.046616 1.154195
H -6.82649 0.698646 1.128349
N -0.73519 -1.168068 0.97331
H -0.148736 -1.259424 1.815131
H -1.71961 -1.18493 1.239095
H -0.899683 -3.184301 0.520066
H -0.571877 -0.232522 0.546459
C 1.120114 -2.582151 0.231488
H 1.302717 -3.604097 -0.126822
O 1.311402 -2.525585 1.640327
H 2.226241 -2.716566 1.861362

conf_03

1 1
C 2.243636 -2.934948 1.699183
C 1.551179 -1.7798 1.006686
H 3.251075 -3.056004 1.29945
H 1.702945 -3.870844 1.541802
H 2.343151 -2.750056 2.772777
C 2.552099 -2.245843 -1.292191
C 3.642062 -1.19388 -1.082036
H 2.26817 -2.279979 -2.351105
H 2.930825 -3.241873 -1.040289
C 3.200404 0.238246 -1.370089
H 4.502728 -1.451639 -1.706463
H 4.010902 -1.252866 -0.049192
C 4.133776 1.284267 -0.775459
H 2.199367 0.404384 -0.956525

H 3.098531 0.388581 -2.452029
C 3.630482 2.720093 -0.927055
H 5.126844 1.199975 -1.230327
H 4.279293 1.058289 0.289443
C 2.184832 2.941698 -0.480416
H 3.716979 3.027492 -1.975308
H 4.293781 3.384671 -0.362148
C 1.882067 2.5047 0.949875
H 1.511119 2.431168 -1.179261
H 1.945494 4.006824 -0.576496
C 0.398735 2.606481 1.314414
H 2.462639 3.121957 1.644141
H 2.222895 1.473528 1.115531
C -0.475197 1.59996 0.633768
H 0.041632 3.606307 1.029803
H 0.259809 2.535833 2.398483
C -1.51277 0.964987 1.195416
H -0.29118 1.430211 -0.429444
C -2.4835 0.089715 0.462325
H -1.745184 1.176531 2.242116
C -3.851485 0.758034 0.299956
H -2.637886 -0.856785 1.002977
H -2.084649 -0.167096 -0.52751
C -4.866153 -0.136792 -0.39709
H -3.722697 1.689167 -0.263929
H -4.231665 1.047546 1.287494
C -6.227473 0.524077 -0.570501
H -4.985973 -1.067811 0.173832
H -4.478572 -0.43057 -1.382105
C -7.236106 -0.375515 -1.268733
H -6.106141 1.454149 -1.139163
H -6.613153 0.817513 0.413574
H -6.890623 -0.65632 -2.268784
H -8.202819 0.121432 -1.380524
H -7.401636 -1.298365 -0.703711
N 0.193116 -1.57551 1.621559
H -0.192463 -0.629583 1.398349
H 0.214567 -1.679823 2.635475
H 2.097083 -0.845243 1.152813
H -0.430462 -2.287558 1.221031
C 1.28735 -1.991391 -0.484218
H 0.788937 -1.087452 -0.86276
O 0.365047 -3.073171 -0.522988
H 0.150515 -3.29628 -1.432036

conf_04

1 1
C 0.486012 2.842819 -1.249886
C -0.528358 1.843632 -0.740522
H 0.503726 3.697002 -0.573941
H 0.213438 3.211563 -2.24512
H 1.488933 2.413831 -1.288502

C -2.996432 1.431569 -0.140061
C -2.758522 0.854929 1.251063
H -3.128478 0.630798 -0.882515
H -3.963386 1.950603 -0.151508
C -3.841792 -0.130833 1.685405
H -2.718042 1.680431 1.967925
H -1.777153 0.371232 1.313629
C -4.008814 -1.399411 0.847013
H -4.802597 0.397929 1.704247
H -3.643741 -0.423731 2.722645
C -2.844846 -2.392661 0.870811
H -4.255833 -1.149311 -0.193782
H -4.895948 -1.91446 1.228663
C -1.691478 -2.109372 -0.088561
H -3.234285 -3.387381 0.62629
H -2.450641 -2.471185 1.892126
C -0.652135 -3.22203 -0.111737
H -1.193412 -1.174959 0.201836
H -2.099265 -1.977363 -1.105505
C 0.476492 -3.023249 -1.119927
H -1.152534 -4.171395 -0.329174
H -0.224054 -3.329866 0.892683
C 1.344873 -1.825525 -0.870009
H 0.080592 -2.996065 -2.143045
H 1.124381 -3.908708 -1.079676
C 2.000442 -1.147902 -1.821705
H 1.513416 -1.566687 0.176505
C 2.99735 -0.042671 -1.626391
H 1.855286 -1.465172 -2.857407
C 3.230757 0.417886 -0.191503
H 2.708842 0.806071 -2.263791
H 3.949678 -0.378681 -2.059462
C 4.120881 -0.523892 0.617296
H 2.26823 0.545206 0.32799
H 3.687706 1.412023 -0.213477
C 4.166814 -0.198309 2.106855
H 5.136435 -0.481936 0.203531
H 3.786338 -1.558591 0.484055
C 4.724544 1.182816 2.423864
H 4.774487 -0.957215 2.610674
H 3.156811 -0.293487 2.528849
H 5.725424 1.308859 1.998267
H 4.803942 1.336559 3.502769
H 4.092401 1.983796 2.028496
N -0.564411 0.656556 -1.660584
H -1.338435 0.02874 -1.424968
H 0.297153 0.065305 -1.589322
H -0.224736 1.453925 0.234292
H -0.673958 0.952252 -2.632
C -1.942103 2.425522 -0.627
H -2.233834 2.784938 -1.630104
O -1.785949 3.512428 0.251851
H -2.620523 3.979929 0.337086

conf_05

1 1
C 0.852642 -1.760011 2.076118
C 0.665607 -2.104821 0.617278
H 0.543789 -2.607306 2.692269
H 0.257457 -0.892182 2.369577
H 1.901411 -1.554783 2.303307
C -1.856257 -1.605383 0.605495
C -3.258666 -2.075359 0.213149
H -1.66538 -0.626672 0.148563
H -1.833474 -1.453264 1.689473
C -4.24269 -0.906917 0.167254
H -3.592349 -2.840791 0.921069
H -3.258148 -2.558037 -0.774835
C -4.086481 -0.059374 -1.101802
H -4.093732 -0.29371 1.06383
H -5.267796 -1.282391 0.23211
C -4.403787 1.425616 -0.92973
H -4.731268 -0.473463 -1.884042
H -3.065752 -0.164079 -1.492601
C -3.447541 2.174541 -0.001895
H -5.425161 1.534564 -0.547364
H -4.398334 1.905172 -1.915508
C -1.997001 2.181454 -0.468762
H -3.491461 1.739904 1.004997
H -3.793952 3.20784 0.111342
C -1.037515 2.760236 0.571982
H -1.907144 2.734807 -1.411532
H -1.68173 1.156794 -0.700312
C 0.391935 2.426567 0.291079
H -1.304566 2.393817 1.570269
H -1.154481 3.850968 0.60515
C 1.234797 1.90076 1.187266
H 0.75344 2.662599 -0.710673
C 2.686458 1.587933 0.983172
H 0.856991 1.725142 2.196229
C 3.219693 1.726578 -0.439198
H 2.884204 0.577746 1.372539
H 3.273145 2.235371 1.648143
C 4.568237 1.042248 -0.646513
H 3.296408 2.789246 -0.688912
H 2.501191 1.31645 -1.166008
C 4.483843 -0.48056 -0.693037
H 5.262141 1.343711 0.147634
H 5.011107 1.392079 -1.58485
C 5.834096 -1.154915 -0.889389
H 3.819489 -0.771606 -1.523405
H 4.040237 -0.861964 0.241005
H 6.302814 -0.820264 -1.818822
H 5.741045 -2.242665 -0.934943
H 6.513469 -0.910575 -0.06813
N 1.030851 -0.939026 -0.264662

H 0.59963 -1.121082 -1.181777
H 2.043966 -0.858144 -0.385255
H 1.351423 -2.90292 0.320394
H 0.699642 -0.025244 0.098048
C -0.739248 -2.55435 0.206009
H -0.899901 -3.541694 0.65912
O -0.62936 -2.676703 -1.20745
H -1.478171 -2.922017 -1.584719

conf_06

1 1
C -0.87036 -2.086777 -1.333846
C -0.384149 -2.328288 0.076395
H -0.583539 -2.92965 -1.96655
H -0.438372 -1.179055 -1.762065
H -1.959236 -2.004343 -1.370353
C 2.018124 -1.613018 -0.523043
C 3.497796 -1.782354 -0.18986
H 1.710904 -0.582852 -0.312334
H 1.870989 -1.769402 -1.597861
C 4.416744 -0.870083 -1.005657
H 3.792712 -2.825143 -0.36163
H 3.662872 -1.582503 0.876385
C 4.068406 0.615653 -0.934698
H 4.3932 -1.189472 -2.054063
H 5.446146 -1.024757 -0.664213
C 4.108772 1.210259 0.469364
H 3.078144 0.768678 -1.379913
H 4.760238 1.170105 -1.578705
C 3.592298 2.647691 0.560408
H 5.139106 1.181283 0.841963
H 3.538817 0.577329 1.161498
C 2.171249 2.850028 0.033554
H 4.261692 3.308782 -0.00131
H 3.648288 2.97795 1.604234
C 1.115121 1.98445 0.73065
H 2.150024 2.639012 -1.041653
H 1.890288 3.90333 0.129096
C -0.103867 1.778431 -0.114431
H 0.837796 2.415928 1.698438
H 1.567784 1.008879 0.956584
C -1.372202 1.957154 0.272037
H 0.08155 1.507325 -1.157821
C -2.576442 1.805667 -0.599645
H -1.560653 2.273808 1.300639
C -3.578447 0.768004 -0.091107
H -2.27217 1.565727 -1.624756
H -3.087329 2.776172 -0.649708
C -4.878337 0.754338 -0.885845
H -3.788819 0.954713 0.969203
H -3.131181 -0.236841 -0.154706
C -5.8388 -0.362037 -0.487438

H -4.640335 0.663467 -1.952687
H -5.375883 1.725033 -0.766196
C -6.345475 -0.267336 0.945577
H -5.350811 -1.333823 -0.64554
H -6.693125 -0.345021 -1.171821
H -5.540759 -0.378614 1.679432
H -7.082599 -1.046375 1.154649
H -6.82646 0.698878 1.128443
N -0.735089 -1.168209 0.973377
H -0.148538 -1.259634 1.815134
H -1.719491 -1.185077 1.23923
H -0.899562 -3.184419 0.519895
H -0.571817 -0.232658 0.546508
C 1.120236 -2.582171 0.231406
H 1.302868 -3.604059 -0.127066
O 1.311544 -2.525825 1.64024
H 2.226532 -2.716295 1.861139

conf_07

1 1
C -1.336009 -2.028917 -0.725772
C -0.608436 -2.238105 0.58182
H -1.198937 -2.907458 -1.360282
H -0.956552 -1.158447 -1.266784
H -2.409379 -1.901171 -0.569033
C 1.664304 -1.693728 -0.494149
C 3.169831 -1.937271 -0.44418
H 1.45945 -0.638254 -0.279154
H 1.299784 -1.884816 -1.509882
C 3.948821 -1.171735 -1.517971
H 3.372506 -3.009623 -0.55876
H 3.554645 -1.650348 0.542587
C 3.602714 0.312785 -1.612326
H 3.765467 -1.638163 -2.492685
H 5.019088 -1.294144 -1.319857
C 3.724677 1.080882 -0.300835
H 2.575208 0.41852 -1.987172
H 4.234756 0.778499 -2.376024
C 3.115539 2.485347 -0.366266
H 4.775705 1.136269 0.004241
H 3.22795 0.509025 0.490708
C 2.447166 2.943305 0.929655
H 2.380598 2.5236 -1.17964
H 3.882663 3.215037 -0.641876
C 1.298945 2.043 1.412731
H 2.059497 3.958083 0.791704
H 3.191545 3.002202 1.731784
C 0.22526 1.830049 0.389853
H 0.859044 2.474069 2.318027
H 1.720079 1.071466 1.711451
C -1.069752 2.125025 0.555034
H 0.540776 1.456059 -0.586501

C -2.136906 1.967654 -0.478794
H -1.386848 2.545178 1.512526
C -3.351502 1.185329 0.019706
H -1.721995 1.49568 -1.377751
H -2.473375 2.966095 -0.787997
C -4.427641 1.013433 -1.043929
H -3.778108 1.691178 0.895452
H -3.029948 0.194353 0.367881
C -5.658357 0.247335 -0.567793
H -3.996322 0.503043 -1.916225
H -4.733007 2.005843 -1.395069
C -5.385426 -1.20537 -0.199361
H -6.413454 0.27576 -1.359783
H -6.101726 0.768216 0.289562
H -4.933259 -1.745719 -1.03896
H -6.309087 -1.726455 0.063415
H -4.716534 -1.293249 0.664372
N -0.723678 -1.022509 1.46642
H -0.026577 -1.137207 2.215439
H -1.654562 -0.943133 1.874172
H -1.076305 -3.04097 1.158119
H -0.535674 -0.119131 0.982578
C 0.88298 -2.573858 0.468635
H 0.943149 -3.61957 0.138354
O 1.343292 -2.466126 1.810535
H 2.271916 -2.704699 1.865483

conf_08

1 1
C 0.949861 -1.932172 1.944925
C 0.477779 -2.087837 0.518432
H 0.566649 -2.758885 2.547177
H 0.6026 -0.997959 2.391972
H 2.040547 -1.961931 2.005298
C -1.868913 -1.144193 0.973711
C -3.386992 -1.33783 0.91363
H -1.599761 -0.168825 0.555197
H -1.591833 -1.114312 2.032924
C -4.073863 -1.315058 -0.456882
H -3.834883 -0.564064 1.543831
H -3.635861 -2.287373 1.402945
C -3.779354 -0.156177 -1.41523
H -5.154226 -1.342 -0.273838
H -3.883607 -2.264196 -0.983883
C -4.212999 1.243212 -0.968793
H -4.307725 -0.38352 -2.347251
H -2.717147 -0.14477 -1.688612
C -3.302233 1.960368 0.028204
H -5.224599 1.185469 -0.549866
H -4.299007 1.870931 -1.863422
C -1.883297 2.19472 -0.476564
H -3.256232 1.403116 0.970629

H -3.753168 2.925893 0.283909
C -0.942584 2.721653 0.613762
H -1.895584 2.882519 -1.329912
H -1.475587 1.255205 -0.871837
C 0.489359 2.392889 0.339959
H -1.225856 2.303663 1.587084
H -1.059742 3.808416 0.703876
C 1.313521 1.813912 1.220828
H 0.864647 2.662216 -0.648093
C 2.755728 1.464212 1.008882
H 0.92236 1.605307 2.218576
C 3.292441 1.628335 -0.409713
H 2.917273 0.434981 1.363182
H 3.362794 2.069554 1.694686
C 4.60082 0.879837 -0.649026
H 3.424827 2.693764 -0.621442
H 2.551545 1.282055 -1.146903
C 4.429521 -0.632298 -0.761708
H 5.310461 1.106364 0.156097
H 5.063305 1.244706 -1.572071
C 5.738885 -1.373635 -0.989956
H 3.749746 -0.846969 -1.603105
H 3.962085 -1.026877 0.154496
H 6.227402 -1.025847 -1.904262
H 5.583735 -2.451243 -1.08339
H 6.430361 -1.205488 -0.159663
N 0.92909 -0.928497 -0.328528
H 0.402421 -0.993628 -1.210434
H 1.932617 -0.963527 -0.524681
H 0.93981 -2.964811 0.056613
H 0.73589 -0.003965 0.105433
C -1.034063 -2.227599 0.307601
H -1.3163 -3.210432 0.709743
O -1.14662 -2.230608 -1.110841
H -2.072317 -2.263232 -1.366957

conf_09

1 1
C 1.156206 -2.826865 -1.399452
C 2.208799 -2.367366 -0.418598
H 1.58923 -2.877662 -2.400879
H 0.304167 -2.144149 -1.43207
H 0.791474 -3.826715 -1.150809
C 1.927982 0.104908 -1.080952
C 2.628045 1.453873 -1.219976
H 1.146084 0.17724 -0.315898
H 1.424823 -0.140932 -2.023218
C 1.700523 2.579949 -1.684514
H 3.459258 1.363833 -1.930436
H 3.070066 1.739323 -0.256932
C 0.404181 2.699515 -0.886971
H 1.45052 2.424222 -2.7403

H 2.255449 3.523306 -1.640048
C 0.588301 2.852079 0.619142
H -0.217213 1.815152 -1.079
H -0.175955 3.543701 -1.275488
C -0.725267 2.717145 1.40132
H 1.067146 3.812241 0.840558
H 1.297222 2.093213 0.968494
C -0.611267 1.920575 2.701622
H -1.480543 2.245942 0.760618
H -1.128532 3.706781 1.634641
C -0.077236 0.48968 2.532784
H -1.596779 1.872495 3.176635
H 0.045443 2.446514 3.403735
C -0.834541 -0.320688 1.526375
H -0.088726 -0.019452 3.502382
H 0.979683 0.550638 2.231385
C -1.384071 -1.519602 1.753933
H -0.978183 0.135956 0.547897
C -2.175082 -2.342845 0.780385
H -1.27689 -1.948134 2.753305
C -2.563407 -1.663214 -0.527854
H -3.082302 -2.687503 1.293525
H -1.618884 -3.270214 0.572361
C -3.669482 -0.623267 -0.370735
H -2.902692 -2.431065 -1.232188
H -1.679376 -1.206199 -0.991274
C -4.041876 0.079999 -1.672705
H -3.380944 0.12607 0.376831
H -4.555527 -1.126377 0.033833
C -2.953641 0.997554 -2.213774
H -4.950282 0.66765 -1.505131
H -4.303995 -0.670585 -2.42866
H -2.693672 1.77052 -1.482637
H -3.28129 1.505857 -3.124053
H -2.038144 0.450912 -2.464336
N 1.636045 -2.256957 0.97131
H 2.322162 -1.730516 1.530054
H 1.491966 -3.174354 1.39061
H 3.004883 -3.111775 -0.330425
H 0.722833 -1.751572 1.019005
C 2.881771 -1.027293 -0.732702
H 3.560439 -1.208278 -1.576974
O 3.636438 -0.764586 0.444881
H 4.143624 0.044494 0.342711

conf_10

1 1
C 0.883991 -3.065278 0.558892
C 0.357875 -1.655799 0.385924
H 0.518458 -3.698509 -0.25023
H 0.550422 -3.496256 1.505609
H 1.976238 -3.085462 0.513927

```
C -1.948896 -2.339221 -0.42637
C -3.460224 -2.095228 -0.351919
H -1.746182 -3.399997 -0.2506
H -1.585023 -2.109252 -1.434719
C -3.878636 -0.62588 -0.35521
H -3.86578 -2.582064 0.54405
H -3.925143 -2.616876 -1.194716
C -3.400811 0.171709 -1.564996
H -3.523891 -0.147159 0.566883
H -4.971258 -0.572296 -0.297911
C -3.649231 1.677235 -1.456044
H -3.901365 -0.208563 -2.462712
H -2.331876 -0.005797 -1.736925
C -2.979854 2.349377 -0.257295
H -4.728472 1.858568 -1.398077
H -3.311622 2.159193 -2.38092
C -1.451668 2.285584 -0.257429
H -3.359663 1.901659 0.669096
H -3.292541 3.397887 -0.216357
C -0.875192 2.427999 1.142897
H -1.050526 3.067654 -0.911642
H -1.111261 1.337283 -0.693751
C 0.61697 2.372443 1.269066
H -1.322889 1.664406 1.798778
H -1.208175 3.378696 1.581965
C 1.508593 2.331977 0.270536
H 1.002022 2.46226 2.288181
C 2.99086 2.468484 0.447565
H 1.149098 2.296582 -0.759045
C 3.834627 1.648397 -0.524659
H 3.267161 2.240043 1.484749
H 3.238551 3.528284 0.302488
C 3.652226 0.143498 -0.398688
H 4.889739 1.89598 -0.368137
H 3.602132 1.951999 -1.552934
C 4.569266 -0.665284 -1.305961
H 2.608663 -0.11194 -0.63723
H 3.834932 -0.154419 0.647787
C 4.338424 -2.164931 -1.206453
H 5.610693 -0.433531 -1.056352
H 4.425407 -0.336957 -2.341872
H 4.496192 -2.525247 -0.183345
H 5.022659 -2.719554 -1.852409
H 3.319538 -2.431649 -1.509921
N 0.974915 -0.772704 1.43916
H 0.901675 0.24178 1.204635
H 1.967264 -0.976959 1.559353
H 0.658499 -1.239924 -0.578692
H 0.471714 -0.951158 2.316501
C -1.150458 -1.501214 0.555084
H -1.388973 -0.44193 0.387878
O -1.390828 -1.817924 1.920555
H -2.33136 -1.767666 2.108416
```

conf_11

1 1
C -1.194675 -2.671986 -1.345831
C -0.544258 -1.356147 -0.975134
H -1.090261 -3.379615 -0.522551
H -0.729326 -3.10849 -2.232443
H -2.266471 -2.544353 -1.523677
C 1.462536 -2.365043 0.23147
C 2.985386 -2.346812 0.411273
H 1.143977 -3.380943 -0.02094
H 0.969589 -2.101824 1.174762
C 3.608639 -0.958469 0.551477
H 3.455783 -2.869261 -0.431323
H 3.226788 -2.951731 1.291247
C 3.101827 -0.149406 1.742058
H 3.451476 -0.391321 -0.37581
H 4.695319 -1.07005 0.631467
C 3.53668 1.317196 1.729177
H 3.458478 -0.618926 2.665663
H 2.007629 -0.202155 1.798957
C 3.028491 2.1276 0.535784
H 4.631371 1.366726 1.745347
H 3.199717 1.792859 2.657463
C 1.508518 2.222062 0.44855
H 3.417811 1.697995 -0.395981
H 3.450057 3.137259 0.585944
C 1.02236 2.74211 -0.895598
H 1.1299 2.861069 1.255005
H 1.069392 1.233574 0.631075
C -0.460077 2.701183 -1.1186
H 1.522052 2.188199 -1.706263
H 1.356971 3.778156 -1.038441
C -1.379055 2.276183 -0.241551
H -0.80762 3.114595 -2.068357
C -2.860505 2.332417 -0.446424
H -1.040914 1.91778 0.729677
C -3.584297 0.992015 -0.280464
H -3.082564 2.757712 -1.431013
H -3.274599 3.033928 0.289999
C -3.410346 0.330321 1.080076
H -3.264435 0.291046 -1.067381
H -4.652117 1.151179 -0.465466
C -4.122315 -1.011594 1.188941
H -3.783961 1.007083 1.858854
H -2.344459 0.186689 1.306608
C -3.916599 -1.686564 2.536312
H -3.770417 -1.675593 0.386682
H -5.193386 -0.866965 1.005858
H -2.854912 -1.874883 2.730153
H -4.438512 -2.645018 2.587949
H -4.292543 -1.061319 3.351429
N -0.798459 -0.347781 -2.068127

H -0.732933 0.626245 -1.698589
H -1.725434 -0.459894 -2.477516
H -0.989514 -0.940791 -0.067878
H -0.084339 -0.503084 -2.791687
C 0.975057 -1.397924 -0.830089
H 1.300952 -0.385398 -0.55491
O 1.446326 -1.690396 -2.140608
H 2.405844 -1.735694 -2.140728

conf_12

1 1
C -2.104768 -3.536078 -0.704043
C -1.515851 -2.195946 -0.317519
H -2.690919 -3.431874 -1.617779
H -2.761481 -3.919937 0.079905
H -1.319155 -4.269357 -0.908928
C -3.559913 -0.816928 -0.963427
C -4.501959 0.328625 -0.576016
H -4.152162 -1.712172 -1.17631
H -3.023188 -0.563546 -1.885151
C -3.813098 1.606384 -0.098674
H -5.198089 -0.01812 0.198688
H -5.132047 0.550707 -1.443435
C -2.88459 2.247302 -1.125725
H -3.25878 1.400334 0.826482
H -4.582751 2.330784 0.189429
C -2.0524 3.408939 -0.579179
H -3.48422 2.606206 -1.969952
H -2.213161 1.491955 -1.552081
C -1.140087 3.049339 0.59446
H -2.724071 4.215294 -0.263572
H -1.448576 3.821801 -1.395553
C -0.121533 1.957273 0.281932
H -1.750115 2.737769 1.451435
H -0.614792 3.951292 0.926324
C 0.51487 1.366542 1.530266
H 0.65352 2.350943 -0.386286
H -0.610594 1.153255 -0.281189
C 1.379683 0.16215 1.32226
H -0.271585 1.111525 2.257138
H 1.115205 2.129326 2.04422
C 1.688083 -0.40602 0.149298
H 1.830146 -0.256069 2.225274
C 2.619524 -1.564606 -0.029782
H 1.298192 0.035953 -0.768952
C 3.885371 -1.180706 -0.805979
H 2.114065 -2.36849 -0.586705
H 2.901687 -1.97293 0.948915
C 4.755182 -0.164314 -0.080825
H 3.599895 -0.788382 -1.790486
H 4.464507 -2.091074 -0.996691
C 6.027843 0.181341 -0.842766

H 5.020743 -0.556156 0.910827
H 4.181181 0.753412 0.100387
C 6.893648 1.198405 -0.114751
H 5.76033 0.566628 -1.834626
H 6.603922 -0.735387 -1.018691
H 6.353442 2.137408 0.043351
H 7.799524 1.429238 -0.680586
H 7.202509 0.824638 0.866622
N -0.619178 -2.37047 0.881873
H 0.054024 -1.573443 0.95794
H -0.081332 -3.235092 0.835624
H -0.884709 -1.796631 -1.115762
H -1.227625 -2.38993 1.710434
C -2.530174 -1.134485 0.10417
H -1.963916 -0.220188 0.330072
O -3.088334 -1.639347 1.311549
H -3.757697 -1.034923 1.641978

conf_13

1 1
C 0.096969 -1.751855 3.171176
C 0.178199 -0.822378 1.978182
H 0.991128 -2.375098 3.21363
H -0.770062 -2.41205 3.097005
H 0.047107 -1.19113 4.108987
C 1.38266 -2.466142 0.466135
C 1.866023 -2.613238 -0.978099
H 1.096654 -3.440328 0.87528
H 2.21091 -2.106168 1.085859
C 2.413394 -1.326704 -1.599944
H 1.052058 -3.000383 -1.604918
H 2.642898 -3.384905 -0.998154
C 3.626854 -0.740087 -0.877583
H 1.618512 -0.574531 -1.67188
H 2.691473 -1.536 -2.638495
C 4.002592 0.685472 -1.306082
H 4.47535 -1.413888 -1.037656
H 3.46413 -0.734285 0.208281
C 3.440792 1.776995 -0.394238
H 3.677735 0.861362 -2.339555
H 5.091775 0.785066 -1.315741
C 1.919814 1.846793 -0.367035
H 3.836735 2.75001 -0.707075
H 3.816337 1.611055 0.624951
C 1.367254 2.85241 0.629781
H 1.519591 0.856157 -0.12287
H 1.555918 2.080806 -1.374926
C -0.125888 2.986598 0.660843
H 1.780259 3.849272 0.420919
H 1.73303 2.61725 1.639992
C -0.991529 2.42866 -0.196298
H -0.521947 3.666594 1.417815

C -2.463498 2.708169 -0.240586
H -0.598743 1.792404 -0.988903
C -3.368403 1.477495 -0.117135
H -2.677202 3.190591 -1.203578
H -2.724345 3.439045 0.531678
C -3.075779 0.372596 -1.124144
H -4.408078 1.800954 -0.234866
H -3.318083 1.07806 0.907541
C -3.988519 -0.837919 -0.983783
H -2.032301 0.037288 -1.035395
H -3.16493 0.782693 -2.138356
C -3.718906 -1.904911 -2.033689
H -5.033471 -0.514544 -1.052208
H -3.873496 -1.269731 0.019856
H -3.907161 -1.523768 -3.041528
H -4.350166 -2.78435 -1.88783
H -2.673788 -2.238614 -2.012328
N -1.040242 0.061543 1.951731
H -0.900545 0.906466 1.354404
H -1.311412 0.373065 2.883967
H 1.043746 -0.159095 2.049468
H -1.804035 -0.496663 1.54679
C 0.195092 -1.525823 0.624044
H 0.242907 -0.745564 -0.148545
O -1.07185 -2.167914 0.545767
H -1.204707 -2.541314 -0.329124

conf_14

1 1
C 1.239127 3.660489 -0.560904
C 0.971208 2.179197 -0.398851
H 2.086055 3.811809 -1.231073
H 1.476405 4.125994 0.398355
H 0.381077 4.171147 -1.007975
C 3.419996 1.475139 -0.341518
C 4.482063 0.597699 0.330863
H 3.763116 2.514275 -0.344804
H 3.297849 1.176812 -1.389343
C 4.080214 -0.860158 0.551059
H 4.766915 1.043394 1.292758
H 5.388814 0.642662 -0.281126
C 3.752549 -1.627659 -0.726191
H 3.2275 -0.904641 1.241294
H 4.89473 -1.370793 1.076356
C 3.163414 -3.019304 -0.487252
H 4.66578 -1.726459 -1.323948
H 3.063285 -1.046218 -1.3507
C 1.862626 -3.036418 0.317056
H 3.902882 -3.638278 0.033358
H 2.996964 -3.50073 -1.457829
C 0.726595 -2.239265 -0.317337
H 2.05143 -2.652112 1.327235

H 1.544146 -4.074815 0.457558
C -0.407719 -1.949499 0.653079
H 0.346013 -2.770466 -1.19766
H 1.11796 -1.287785 -0.697408
C -1.459264 -0.993936 0.177019
H 0.009238 -1.563173 1.595751
H -0.903672 -2.885145 0.94448
C -1.539169 -0.452755 -1.045284
H -2.240928 -0.766902 0.902867
C -2.591689 0.494796 -1.544109
H -0.784239 -0.72826 -1.781724
C -3.705092 0.866581 -0.570588
H -3.033522 0.058635 -2.44991
H -2.089897 1.403265 -1.915308
C -4.746386 -0.230399 -0.371823
H -4.210805 1.764835 -0.941653
H -3.283945 1.146841 0.407967
C -5.793645 0.123399 0.675233
H -4.260018 -1.17386 -0.096995
H -5.241284 -0.419375 -1.332834
C -6.852013 -0.956596 0.840558
H -6.273103 1.071356 0.40093
H -5.296787 0.302507 1.638072
H -7.388971 -1.129225 -0.097125
H -7.589345 -0.680285 1.598081
H -6.403585 -1.907724 1.144678
N -0.267662 1.982775 0.436222
H -0.662255 1.025382 0.286584
H -0.994835 2.661841 0.214379
H 0.767967 1.70634 -1.36331
H 0.012011 2.089802 1.41949
C 2.061367 1.393952 0.328207
H 1.741264 0.342821 0.348942
O 2.040856 1.90726 1.654852
H 2.717486 1.477449 2.183922

conf_15

1 1
C -0.745935 -2.808605 1.345251
C -0.174763 -1.503513 0.830606
H -0.066005 -3.248251 2.075753
H -0.884071 -3.525859 0.532957
H -1.702711 -2.647732 1.850395
C 2.247533 -2.288814 0.763734
C 3.570863 -2.287695 -0.013092
H 1.975394 -3.318155 1.015545
H 2.372549 -1.754134 1.712519
C 3.910589 -0.959632 -0.688747
H 3.554457 -3.081921 -0.769452
H 4.36829 -2.570079 0.68141
C 3.894191 0.249806 0.24096
H 3.203853 -0.774418 -1.511784

H 4.887792 -1.046134 -1.174618
C 3.852461 1.587538 -0.500063
H 4.760053 0.215622 0.911655
H 3.022289 0.187205 0.90115
C 3.12406 2.687304 0.270292
H 3.369615 1.448635 -1.475761
H 4.86959 1.920818 -0.727612
C 1.624087 2.456918 0.471357
H 3.259318 3.641027 -0.252762
H 3.593169 2.813896 1.252907
C 0.836018 2.37585 -0.830226
H 1.227374 3.284529 1.069487
H 1.456376 1.553175 1.07393
C -0.659105 2.396106 -0.704382
H 1.141968 1.499448 -1.420825
H 1.111724 3.229162 -1.464921
C -1.371614 2.343416 0.428256
H -1.202456 2.554533 -1.637778
C -2.851515 2.563338 0.536539
H -0.84459 2.219381 1.376163
C -3.659562 1.30774 0.891386
H -3.233362 2.987906 -0.398203
H -3.011591 3.324322 1.307869
C -4.051602 0.489845 -0.335076
H -4.569746 1.592197 1.428001
H -3.089092 0.69296 1.605234
C -4.599357 -0.894955 -0.009852
H -3.203401 0.411338 -1.033527
H -4.808423 1.044219 -0.901432
C -4.927819 -1.721493 -1.243403
H -5.491903 -0.784704 0.615903
H -3.895114 -1.452047 0.635002
H -5.676628 -1.217332 -1.86033
H -5.3255 -2.702991 -0.975958
H -4.044399 -1.87995 -1.872766
N -1.169421 -0.863964 -0.098839
H -1.034021 0.163091 -0.189959
H -2.135144 -1.011697 0.204751
H -0.011847 -0.797016 1.648405
H -1.041869 -1.304095 -1.019496
C 1.105938 -1.633504 0.00994
H 1.407691 -0.615098 -0.273604
O 0.708271 -2.337803 -1.160691
H 1.463336 -2.459576 -1.741721

ref_01

1 1
C 3.954391 -2.236955 -0.601179
H 4.43302 -3.08112 -1.103577
H 3.118394 -1.92437 -1.235437
H 3.54287 -2.613263 0.343302
C 4.949388 -1.11114 -0.349211

H 5.352221 -0.752761 -1.304184
H 5.803696 -1.510546 0.20656
C 4.370072 0.065873 0.430215
H 5.178493 0.74849 0.716009
H 3.941682 -0.303084 1.373552
C 3.312465 0.857391 -0.327935
H 2.525353 0.175548 -0.678174
H 3.752629 1.281368 -1.238721
C 2.698949 1.97901 0.510351
H 3.489613 2.702247 0.74944
H 2.354788 1.58788 1.476509
C 1.582299 2.702386 -0.173344
H 1.794601 3.075234 -1.177562
C 0.386541 2.970794 0.36437
H 0.198084 2.636723 1.386854
C -0.700506 3.759592 -0.305744
H -0.268031 4.350542 -1.11852
H -1.11501 4.474507 0.414668
C -1.830995 2.894787 -0.87266
H -1.401294 2.233784 -1.640773
H -2.542051 3.533098 -1.407062
C -2.573695 2.056049 0.163304
H -3.215745 2.697015 0.777119
H -1.855216 1.625766 0.876819
C -3.394864 0.929326 -0.460166
H -2.806991 0.468847 -1.271162
H -4.264346 1.343802 -0.98105
C -3.844863 -0.147627 0.528536
H -4.755025 0.179853 1.039352
H -3.096008 -0.248779 1.327064
C -4.081383 -1.511948 -0.118732
H -4.779008 -1.396099 -0.955573
H -4.577429 -2.175422 0.598139
C -2.806773 -2.189041 -0.626646
H -2.274918 -1.517758 -1.309714
H -3.088593 -3.031816 -1.263809
C -1.878512 -2.698998 0.491522
H -2.065984 -3.766842 0.640421
H -2.138331 -2.239709 1.451309
C -0.369607 -2.526298 0.272948
H 0.155377 -3.244773 0.916342
C 0.239905 -1.169693 0.637488
H 1.319639 -1.27225 0.500807
O 0.024918 -2.703688 -1.079556
H -0.227942 -3.574269 -1.396011
N -0.173602 -0.168025 -0.402966
H 0.313919 0.74308 -0.305244
H -1.177292 0.027443 -0.360199
H 0.024964 -0.576803 -1.322092
C -0.057379 -0.661725 2.028528
H 0.519553 0.23935 2.247147
H 0.229757 -1.421383 2.758824
H -1.11616 -0.438547 2.175974

13E-1-DeoxySO

conf_00

```
1 1
C -0.022275 1.485691 2.296157
C -0.364165 2.102362 0.959188
H 0.192152 2.282444 3.011907
H 0.856942 0.839966 2.243335
H -0.859267 0.905943 2.690537
C 2.145625 2.49603 0.424959
H 2.448549 2.396609 1.473679
H 2.794729 3.277852 0.011361
C 4.121135 -0.461274 -1.207363
C 3.074519 -1.574557 -1.185428
H 5.105627 -0.879268 -0.970841
H 4.193277 -0.081476 -2.233281
C 2.856195 -2.242987 0.167676
H 3.373409 -2.343276 -1.907147
H 2.122867 -1.182743 -1.568894
C 1.76977 -3.318956 0.137157
H 2.622682 -1.495376 0.938248
H 3.798237 -2.700867 0.489858
C 0.335866 -2.808019 -0.021871
H 1.824521 -3.918181 1.053435
H 1.989523 -4.008797 -0.685366
H -0.312441 -3.654363 -0.2727
H 0.271839 -2.125789 -0.88143
C -2.485156 -1.799701 0.158984
C -3.943717 -1.463598 0.207337
H -2.131675 -2.255469 -0.767217
C -4.479491 -0.814428 -1.070148
H -4.155525 -0.844676 1.086339
H -4.495657 -2.399132 0.365453
C -3.779561 0.479552 -1.472807
H -5.550187 -0.621847 -0.941953
H -4.397015 -1.531677 -1.894651
C -3.88127 1.588615 -0.433217
H -4.206188 0.835132 -2.415817
H -2.728101 0.251541 -1.704873
H -4.927066 1.84936 -0.248405
H -3.379285 2.504885 -0.762731
H -3.462761 1.293972 0.536954
N -0.670103 1.049132 -0.070468
H -1.025738 1.510141 -0.910814
H -1.34404 0.328939 0.250215
H -1.289036 2.679491 1.037907
H 0.185587 0.557544 -0.33904
C 0.712568 3.025525 0.380811
H 0.66915 3.945387 0.97877
O 0.260756 3.279909 -0.942092
H 0.795632 3.963344 -1.352846
C -1.632532 -1.669468 1.182437
```

H -2.022645 -1.256581 2.115981
C -0.207318 -2.137299 1.232546
H -0.124814 -2.840108 2.073121
H 0.442264 -1.30064 1.530076
C 2.402631 1.19362 -0.323291
H 2.089083 1.301477 -1.369964
H 1.810984 0.378929 0.119416
C 3.84764 0.71462 -0.265972
H 4.085223 0.439065 0.768524
H 4.521056 1.540672 -0.520072

conf_01

1 1
C -1.812356 2.865717 0.050464
C -0.56141 3.025993 -0.780814
H -1.808156 3.599742 0.859238
H -1.882214 1.870639 0.495223
H -2.710274 3.043912 -0.546694
C 0.918499 1.705328 0.859358
H 0.751368 0.798858 0.265838
H 0.138937 1.728344 1.629493
C 2.227532 -0.934298 1.803671
C 3.139431 -1.181632 0.606931
H 1.176445 -1.053465 1.513382
H 2.408062 -1.71264 2.554196
C 3.120274 -2.620331 0.089692
H 4.169813 -0.943936 0.901148
H 2.901066 -0.482227 -0.203406
C 1.789784 -3.183579 -0.416817
H 3.468814 -3.266892 0.903942
H 3.867143 -2.72053 -0.707339
C 1.334716 -2.733219 -1.806862
H 0.996309 -3.016813 0.32131
H 1.901741 -4.271707 -0.464549
H 0.577531 -3.437448 -2.168829
H 2.180483 -2.811391 -2.499945
C -1.714502 -0.907506 -1.541212
C -2.953647 -0.780889 -0.709903
H -1.863005 -0.865494 -2.62375
C -2.762919 -0.918975 0.793675
H -3.666462 -1.534999 -1.065909
H -3.444536 0.179607 -0.934114
C -4.035474 -0.663079 1.596953
H -1.981981 -0.225216 1.134355
H -2.386245 -1.924716 1.018667
C -5.154342 -1.658194 1.32071
H -4.391428 0.356714 1.39825
H -3.783104 -0.69225 2.662202
H -5.52098 -1.593593 0.292256
H -6.00783 -1.47843 1.978576
H -4.816983 -2.685688 1.490851
N -0.510236 1.994657 -1.877122

H 0.444862 2.017975 -2.259989
H -1.177586 2.199332 -2.619304
H -0.564164 3.987954 -1.300675
H -0.706044 1.024741 -1.539181
C 0.766912 2.930608 -0.027727
H 0.847788 3.840379 0.582311
O 1.729009 2.960968 -1.075837
H 2.617597 2.932487 -0.712615
C -0.474785 -1.124353 -1.08451
H -0.33363 -1.222574 -0.0103
C 0.739766 -1.321876 -1.934947
H 1.506447 -0.582812 -1.666956
H 0.483107 -1.147578 -2.987046
C 2.282275 1.627762 1.536887
H 2.454531 2.547297 2.109421
H 3.076326 1.577266 0.780559
C 2.428138 0.426503 2.469371
H 1.720483 0.530107 3.300473
H 3.42799 0.460821 2.915944

conf_02

1 1
C -0.91011 3.458113 0.010609
C -0.492356 2.005447 0.106401
H -0.53884 3.893207 -0.917542
H -0.511749 4.039989 0.84485
H -2.000151 3.55147 -0.006701
C 1.854141 2.362249 -0.830046
H 1.627163 3.426616 -0.939404
H 1.5667 1.874045 -1.768774
C 3.562229 -0.258178 -1.304499
C 4.057741 -1.647401 -0.902661
H 4.053286 0.051477 -2.234361
H 2.489388 -0.304382 -1.536877
C 3.205202 -2.325098 0.16974
H 5.092308 -1.56055 -0.551559
H 4.093062 -2.293408 -1.787269
C 1.910344 -2.924324 -0.373656
H 2.984076 -1.608952 0.97247
H 3.782015 -3.124152 0.64768
C 0.912659 -3.30561 0.718484
H 2.15864 -3.812379 -0.964232
H 1.440955 -2.229039 -1.081946
H 1.44587 -3.807933 1.533212
H 0.186216 -4.028962 0.334148
C -2.200477 -1.516375 0.574276
C -3.243049 -1.142951 -0.430629
H -2.566251 -1.794958 1.565798
C -4.051941 0.105658 -0.068335
H -3.93216 -1.99124 -0.518439
H -2.785083 -1.015803 -1.418295
C -5.259355 0.346236 -0.973041

H -3.396931 0.987362 -0.134754
H -4.391561 0.034584 0.974577
C -6.356221 -0.699459 -0.829348
H -4.923056 0.39308 -2.016151
H -5.673491 1.333904 -0.743436
H -6.024541 -1.694911 -1.137093
H -7.218132 -0.443581 -1.449779
H -6.703818 -0.767289 0.206548
N -1.125842 1.388174 1.326648
H -1.204615 0.354738 1.205613
H -2.06526 1.748848 1.491472
H -0.857892 1.434259 -0.750783
H -0.521142 1.613769 2.127214
C 1.008229 1.765123 0.278993
H 1.168738 0.676191 0.293279
O 1.295041 2.292547 1.568654
H 2.231659 2.198194 1.760105
C -0.885625 -1.599457 0.334453
H -0.537287 -1.36039 -0.672919
C 0.14369 -2.107584 1.295954
H -0.34403 -2.389226 2.234775
H 0.867785 -1.316529 1.543562
C 3.358375 2.205067 -0.590925
H 3.670756 2.901459 0.197632
H 3.877925 2.547077 -1.491905
C 3.832489 0.797204 -0.237701
H 3.39264 0.476058 0.717473
H 4.911571 0.837789 -0.052357

conf_03

1 1
C -0.031933 -1.221469 2.340735
C 0.358327 -1.89174 1.044001
H -0.220894 -1.985799 3.097743
H -0.937912 -0.619802 2.238061
H 0.771733 -0.582284 2.713912
C -2.128146 -2.454196 0.490819
H -2.44504 -2.320346 1.531861
H -2.718895 -3.30143 0.120052
C -4.321664 0.305718 -1.217987
C -3.365873 1.497963 -1.188384
H -5.337855 0.637441 -0.978505
H -4.362929 -0.072603 -2.246012
C -3.196909 2.164952 0.176682
H -3.714629 2.246855 -1.90744
H -2.389997 1.173436 -1.571223
C -1.935919 3.022202 0.286734
H -3.170896 1.408501 0.973107
H -4.079452 2.777753 0.386731
C -0.664977 2.183055 0.353129
H -1.990505 3.645444 1.186119
H -1.876972 3.712669 -0.563734

H -0.605812 1.565224 -0.555868
H -0.747228 1.503934 1.214965
C 2.885923 1.979851 -0.065233
C 4.169396 1.279057 0.255984
H 2.85511 2.488397 -1.031505
C 4.748656 0.482454 -0.914869
H 4.050455 0.653656 1.148954
H 4.902559 2.047214 0.534095
C 3.881292 -0.680014 -1.386466
H 5.736802 0.10312 -0.632441
H 4.915039 1.167603 -1.753749
C 3.763642 -1.805702 -0.366129
H 4.299539 -1.086332 -2.312452
H 2.889919 -0.29193 -1.66588
H 4.743261 -2.25045 -0.170852
H 3.1106 -2.612108 -0.71672
H 3.391565 -1.453912 0.604264
N 0.648802 -0.877884 -0.026878
H 0.840641 -1.389334 -0.895002
H 1.436318 -0.253014 0.192198
H 1.300643 -2.431323 1.164641
H -0.160812 -0.274325 -0.191466
C -0.664901 -2.8901 0.486982
H -0.574976 -3.782222 1.120825
O -0.175414 -3.172103 -0.818063
H -0.686058 -3.876249 -1.224729
C 1.836206 2.124179 0.749311
H 1.87549 1.660103 1.739498
C 0.635252 2.976067 0.465218
H 0.797033 3.550157 -0.452927
H 0.529903 3.709064 1.275259
C -2.47062 -1.213222 -0.323844
H -2.14877 -1.347446 -1.364506
H -1.931201 -0.343772 0.073538
C -3.950035 -0.847565 -0.280369
H -4.217168 -0.597951 0.75361
H -4.553201 -1.724878 -0.53965

conf_04

1 1
C 0.892945 -2.204287 2.826765
C 0.918319 -1.616677 1.431737
H 0.972059 -3.288122 2.7546
H -0.04632 -1.972254 3.340141
H 1.728933 -1.840175 3.431735
C -0.169014 -1.588866 -0.885033
H 0.823967 -1.799458 -1.300318
H -0.289007 -0.498289 -0.881284
C -3.32416 -0.744642 -1.400148
C -3.057088 0.109605 -0.166566
H -4.408209 -0.849287 -1.517973
H -2.979821 -0.207677 -2.294363

C -3.599454 1.52744 -0.287496
H -1.976886 0.160557 0.005694
H -3.485001 -0.378714 0.720015
C -3.211103 2.445086 0.872109
H -4.69138 1.496258 -0.36591
H -3.251113 1.960124 -1.233364
C -1.71397 2.478291 1.176737
H -3.74369 2.139268 1.779763
H -3.551789 3.462506 0.650072
H -1.425443 1.497372 1.586327
H -1.522179 3.195978 1.982123
C 1.556893 2.350411 -0.632594
C 3.039595 2.489522 -0.455991
H 1.220283 1.965164 -1.598807
C 3.860448 1.35277 -1.059168
H 3.278959 2.614229 0.607916
H 3.343931 3.427101 -0.939036
C 3.546364 -0.013649 -0.469404
H 4.924514 1.571759 -0.921843
H 3.695421 1.320127 -2.14336
C 4.421384 -1.129984 -1.018616
H 2.494069 -0.251267 -0.682118
H 3.657032 0.034825 0.625499
H 4.319364 -1.206914 -2.104911
H 4.161359 -2.1027 -0.591038
H 5.476073 -0.943613 -0.798642
N 0.824138 -0.119958 1.548383
H 0.99005 0.413749 0.676457
H 1.511454 0.22243 2.221337
H 1.87495 -1.818194 0.943156
H -0.098826 0.166377 1.888469
C -0.215517 -2.129432 0.540345
H -1.170888 -1.841997 1.013761
O -0.057678 -3.526494 0.581888
H -0.716167 -3.936769 0.016022
C 0.631969 2.779529 0.234764
H 0.970446 3.219182 1.177186
C -0.841365 2.823684 -0.028435
H -1.075891 2.177496 -0.881325
H -1.103803 3.841643 -0.348844
C -1.221021 -2.212028 -1.808939
H -0.954772 -3.264086 -1.973045
H -1.11854 -1.746117 -2.794954
C -2.691104 -2.136843 -1.382526
H -2.838359 -2.592184 -0.392869
H -3.247047 -2.77626 -2.075309

conf_05

1 1
C -2.624406 -1.559671 1.481627
C -1.242743 -2.052921 1.838103
H -3.204077 -2.385751 1.063547

H -2.594857 -0.758464 0.740714
H -3.15921 -1.201264 2.36487
C -0.354281 -1.818532 -0.560432
H 0.01663 -0.821573 -0.292929
H -1.363883 -1.682619 -0.963588
C 1.255867 -0.097615 -2.496947
C 2.442251 -0.091724 -1.542206
H 0.443045 0.497807 -2.058493
H 1.522931 0.422006 -3.42356
C 2.953284 1.304277 -1.193136
H 3.26311 -0.686969 -1.961587
H 2.156661 -0.60423 -0.615896
C 3.695613 1.353399 0.141279
H 2.120892 2.018291 -1.183584
H 3.620964 1.657751 -1.985493
C 2.85539 1.077791 1.391796
H 4.161868 2.338661 0.258197
H 4.52163 0.632422 0.113745
H 3.539423 1.046807 2.245793
H 2.411636 0.073374 1.33691
C -0.742688 2.128461 1.6223
C -2.087896 2.111806 0.962119
H -0.737927 2.427193 2.673678
C -2.103107 1.727372 -0.508584
H -2.525078 3.112505 1.081827
H -2.767817 1.45792 1.528698
C -3.505501 1.637473 -1.095003
H -1.597408 0.762718 -0.650923
H -1.51538 2.454246 -1.082357
C -3.498363 1.220751 -2.557902
H -4.006527 2.606376 -0.987063
H -4.102509 0.925162 -0.50974
H -2.942004 1.937382 -3.169589
H -4.511435 1.157652 -2.961668
H -3.027347 0.240122 -2.689235
N -0.396443 -0.947385 2.419038
H 0.572564 -1.296392 2.430373
H -0.681234 -0.71814 3.370325
H -1.296284 -2.802098 2.632812
H -0.407757 -0.056275 1.876825
C -0.436535 -2.673175 0.694742
H -0.910284 -3.636041 0.461259
O 0.837945 -2.889989 1.288946
H 1.432798 -3.306603 0.660645
C 0.439546 1.916208 1.028759
H 0.452425 1.665265 -0.030017
C 1.768087 2.120473 1.693386
H 1.610269 2.20021 2.775146
H 2.146249 3.10478 1.383012
C 0.5388 -2.415709 -1.642774
H 0.1162 -3.369061 -1.980241
H 1.5286 -2.646545 -1.228427
C 0.738325 -1.494138 -2.847162
H -0.205499 -1.400376 -3.396636

H 1.440321 -1.981804 -3.531976

conf_06

1 1
C -0.385172 1.414591 1.825418
C -0.661106 1.855964 0.406141
H -0.4534 2.278222 2.490556
H 0.610416 0.980363 1.941243
H -1.125692 0.684815 2.159035
C 1.762863 2.756884 0.128453
H 1.934268 2.878981 1.204271
H 2.290295 3.594855 -0.344525
C 4.330496 -0.128994 -0.316867
C 3.904112 -1.055932 0.830057
H 5.418491 -0.163325 -0.423089
H 3.930227 -0.494903 -1.271122
C 3.59318 -2.488097 0.39889
H 3.030655 -0.640755 1.35063
H 4.696197 -1.073897 1.585164
C 2.327783 -2.646433 -0.443358
H 3.506054 -3.122943 1.288451
H 4.444488 -2.881211 -0.168534
C 1.024887 -2.358351 0.295242
H 2.285417 -3.673723 -0.823217
H 2.386612 -2.008715 -1.336089
H 1.018374 -1.323909 0.669675
H 0.967453 -2.980618 1.196777
C -2.624865 -1.915854 -0.57791
C -3.993786 -1.810299 0.019653
H -2.564562 -1.775639 -1.66179
C -4.843066 -0.661605 -0.5236
H -3.921548 -1.769344 1.111716
H -4.518005 -2.748398 -0.205862
C -4.300485 0.735975 -0.243339
H -5.848302 -0.7423 -0.095837
H -4.964796 -0.789797 -1.605644
C -4.156553 1.054749 1.238376
H -4.9672 1.469711 -0.707584
H -3.337204 0.862482 -0.760366
H -5.113717 0.932128 1.753493
H -3.834928 2.087548 1.403253
H -3.44015 0.39354 1.736873
N -0.620544 0.689537 -0.540701
H -0.765197 1.055194 -1.487675
H -1.319818 -0.046034 -0.322469
H -1.683895 2.23137 0.324124
H 0.299454 0.243975 -0.526019
C 0.272612 2.941896 -0.144231
H -0.050081 3.879332 0.32715
O -0.032288 2.968237 -1.533438
H 0.412878 3.703028 -1.962024
C -1.520753 -2.301838 0.073546

H -1.592068 -2.493011 1.146251
C -0.204734 -2.62481 -0.57199
H -0.119085 -2.109089 -1.539435
H -0.206253 -3.69313 -0.826973
C 2.368983 1.441792 -0.342302
H 2.14965 1.282837 -1.406437
H 1.913641 0.612055 0.215579
C 3.873009 1.315843 -0.126242
H 4.129767 1.653481 0.885918
H 4.399252 1.981281 -0.818668

conf_07

1 1
C 0.751651 -1.907789 2.180416
C 0.688361 -2.270818 0.715346
H 0.364039 -2.734849 2.779227
H 0.156498 -1.018589 2.403463
H 1.780943 -1.724978 2.498382
C -1.805384 -1.673123 0.524221
H -1.486627 -0.651701 0.280781
H -1.97226 -1.701781 1.606895
C -3.922203 0.444045 0.145648
C -3.384323 0.916909 -1.20287
H -3.215346 0.684915 0.945521
H -4.826398 1.015809 0.381884
C -2.944538 2.382755 -1.247291
H -4.163607 0.750914 -1.956118
H -2.535908 0.293966 -1.520735
C -1.508831 2.65672 -0.796826
H -3.635776 2.992644 -0.651606
H -3.039794 2.744101 -2.276251
C -1.225413 2.42827 0.684456
H -1.269148 3.701571 -1.031072
H -0.82713 2.051221 -1.411594
H -1.301309 1.362876 0.934175
H -2.000634 2.930087 1.274898
C 2.231656 1.570815 1.257775
C 3.492573 0.943864 0.74514
H 2.097502 1.521528 2.339672
C 3.732622 1.016424 -0.758907
H 3.54406 -0.096705 1.104056
H 4.336906 1.42402 1.257236
C 4.878033 0.123682 -1.231151
H 3.933538 2.057219 -1.031244
H 2.822666 0.749302 -1.318909
C 4.54595 -1.363047 -1.194141
H 5.767272 0.31823 -0.621192
H 5.14633 0.39912 -2.255277
H 4.31236 -1.714131 -0.179818
H 5.386584 -1.972039 -1.533928
H 3.705691 -1.597598 -1.861583
N 1.1933 -1.135523 -0.139732

H 0.741264 -1.239086 -1.060195
H 2.204701 -1.188002 -0.276405
H 1.357849 -3.106166 0.494247
H 0.999821 -0.198132 0.252934
C -0.694008 -2.650644 0.175581
H -0.931654 -3.644238 0.577504
O -0.472534 -2.736838 -1.22807
H -1.288162 -2.973705 -1.676958
C 1.313545 2.236087 0.547513
H 1.445931 2.34444 -0.529081
C 0.138994 2.937035 1.154171
H 0.214962 4.005841 0.913911
H 0.200975 2.859797 2.244618
C -3.114402 -1.972853 -0.199322
H -3.410508 -3.011839 -0.008402
H -2.964707 -1.883192 -1.282953
C -4.261321 -1.04466 0.204028
H -4.584848 -1.294056 1.221086
H -5.116681 -1.25548 -0.447209

conf_08

1 1
C -1.638199 -2.404782 -1.375492
C -1.335691 -2.463631 0.103862
H -1.327817 -3.338942 -1.848623
H -1.108381 -1.585014 -1.86625
H -2.709693 -2.288151 -1.555883
C 1.171218 -1.976578 -0.306494
H 0.960237 -0.902046 -0.258222
H 1.099383 -2.268952 -1.360557
C 3.656342 -0.293352 -1.111327
C 3.784564 0.644888 0.081588
H 2.728145 -0.058641 -1.652364
H 4.46292 -0.086485 -1.823733
C 3.720056 2.130676 -0.305459
H 4.725078 0.441955 0.607663
H 2.996555 0.420882 0.807915
C 3.021362 3.023619 0.726266
H 3.20344 2.236383 -1.269573
H 4.735634 2.496259 -0.482433
C 1.509393 3.136628 0.52045
H 3.439548 4.03399 0.688131
H 3.232438 2.648755 1.73621
H 1.312017 3.683159 -0.409684
H 1.069651 3.731747 1.328966
C -1.306765 1.4563 -0.859601
C -2.786666 1.449609 -1.093448
H -0.696173 1.109188 -1.696241
C -3.662816 1.792572 0.107607
H -3.06045 0.461455 -1.493269
H -3.015892 2.136041 -1.918756
C -5.101673 1.303662 -0.038812

H -3.645079 2.874517 0.270459
H -3.24327 1.356447 1.02709
C -5.237157 -0.205061 0.126588
H -5.497486 1.606521 -1.01479
H -5.727497 1.798476 0.709643
H -4.655249 -0.757312 -0.621907
H -6.272786 -0.533871 0.015362
H -4.917397 -0.516376 1.131095
N -1.727671 -1.178757 0.780284
H -1.349129 -1.215972 1.735304
H -2.742248 -1.061858 0.810551
H -1.949548 -3.227507 0.589221
H -1.339326 -0.32552 0.322442
C 0.123189 -2.754764 0.47499
H 0.275884 -3.828513 0.298679
O 0.166631 -2.487373 1.871381
H 1.035419 -2.696921 2.222903
C -0.670152 1.850436 0.250284
H -1.246213 2.258157 1.08151
C 0.808883 1.779968 0.435287
H 1.239786 1.212934 -0.395621
H 1.024713 1.217545 1.355432
C 2.589356 -2.253391 0.191195
H 2.711911 -3.334646 0.339652
H 2.735729 -1.784757 1.172411
C 3.687381 -1.779049 -0.762566
H 3.622433 -2.363432 -1.688212
H 4.657615 -2.025716 -0.317065

conf_09

1 1
C -1.196234 3.110497 -0.201968
C 0.052163 2.72808 -0.960618
H -0.987063 3.986916 0.415528
H -1.542972 2.309437 0.454267
H -2.008279 3.377817 -0.882783
C 1.126726 1.438505 0.980487
H 0.917938 0.460772 0.529064
H 0.235635 1.712069 1.555225
C 3.614306 -0.477331 0.651261
C 2.784891 -1.596316 1.268386
H 4.646069 -0.820353 0.513347
H 3.240605 -0.271158 -0.358598
C 2.776326 -2.899073 0.469138
H 1.751524 -1.254827 1.4024
H 3.152678 -1.806171 2.279859
C 2.444539 -2.764688 -1.019526
H 2.068806 -3.58814 0.944123
H 3.756456 -3.38186 0.551361
C 1.214088 -1.927295 -1.360851
H 2.317304 -3.769717 -1.437393
H 3.301818 -2.33303 -1.548279

H 1.074665 -1.943497 -2.449552
H 1.409661 -0.88015 -1.093632
C -2.021996 -0.763521 -0.418485
C -3.251191 -0.062027 -0.911254
H -1.791762 -0.674496 0.644549
C -4.537615 -0.571029 -0.249101
H -3.328644 -0.188796 -1.997678
H -3.182277 1.018169 -0.715451
C -4.621416 -0.294736 1.247052
H -4.630522 -1.647681 -0.433438
H -5.390487 -0.09707 -0.748581
C -5.949423 -0.734467 1.845526
H -4.476447 0.77994 1.423365
H -3.804468 -0.806367 1.769856
H -6.787385 -0.21328 1.372794
H -5.992057 -0.528782 2.91781
H -6.107109 -1.808302 1.706832
N -0.171826 1.495449 -1.80027
H 0.755826 1.194712 -2.130684
H -0.770132 1.687939 -2.602598
H 0.314366 3.507761 -1.681172
H -0.600095 0.699612 -1.28231
C 1.307283 2.468815 -0.123245
H 1.592934 3.435122 0.315047
O 2.258601 2.074939 -1.103999
H 3.089433 1.842426 -0.680585
C -1.257873 -1.569775 -1.165805
H -1.514741 -1.688903 -2.221912
C -0.086821 -2.364247 -0.686486
H -0.265869 -3.423899 -0.909751
H -0.004056 -2.284592 0.402065
C 2.281247 1.319152 1.979705
H 1.940971 0.649421 2.7755
H 2.428717 2.292792 2.46166
C 3.636922 0.813687 1.473565
H 4.267157 0.66121 2.35657
H 4.160241 1.601922 0.910371

ref_01

1 1
C 6.084246 -1.197153 -0.599602
H 5.808625 -1.893227 0.199782
H 7.167894 -1.25441 -0.723077
H 5.630377 -1.555233 -1.529838
C 5.639338 0.22025 -0.275988
H 5.950374 0.902158 -1.07564
H 6.137211 0.570117 0.635433
C 4.132496 0.339251 -0.083337
H 3.831457 -0.322135 0.744426
H 3.65458 -0.031552 -1.007737
C 3.65491 1.766588 0.189342
H 3.794111 2.375022 -0.710132

H 4.307665 2.200064 0.957922
C 2.239625 1.880699 0.666479
H 2.032623 1.47609 1.661403
C 1.2561 2.511989 0.018756
H 1.478595 2.947502 -0.957594
C -0.11993 2.763127 0.546717
H -0.225097 3.844125 0.710681
H -0.237208 2.29831 1.533804
C -1.243173 2.313131 -0.38671
H -1.217559 1.218213 -0.490785
H -1.068333 2.71075 -1.394649
C -2.618463 2.751977 0.102046
H -2.641728 3.847502 0.125613
H -2.751356 2.433488 1.143159
C -3.784856 2.255203 -0.749983
H -3.677941 2.647543 -1.768331
H -4.711367 2.688093 -0.355711
C -3.938099 0.736257 -0.816935
H -3.056206 0.30301 -1.307155
H -4.782614 0.495473 -1.473576
C -4.161658 0.08044 0.54073
H -5.061577 0.506888 0.998452
H -3.341726 0.339906 1.221713
C -4.313041 -1.438349 0.493126
H -5.221867 -1.700422 -0.05946
H -4.457775 -1.813311 1.513008
C -3.132944 -2.173873 -0.143605
H -3.122421 -1.956412 -1.219115
H -3.290824 -3.255009 -0.04155
C -1.789362 -1.794296 0.469785
H -1.838985 -1.925031 1.556827
H -1.601039 -0.728292 0.290648
C -0.630556 -2.62308 -0.061489
H -0.727007 -3.658271 0.291924
C 0.754432 -2.133682 0.372915
H 1.480371 -2.884693 0.049781
O -0.546992 -2.604191 -1.481456
H -1.369601 -2.9151 -1.867732
N 1.072282 -0.920969 -0.460022
H 2.080701 -0.750073 -0.521927
H 0.674534 -0.047823 -0.094771
H 0.68757 -1.09569 -1.399431
C 0.917377 -1.831494 1.844133
H 1.949872 -1.564886 2.08166
H 0.663161 -2.71787 2.429421
H 0.263805 -1.016405 2.16607

14E-1-DeoxySO

conf_00

1 1
C 0.973274 -1.502244 1.558534

```
C 0.715739 -1.857734 0.111802
H 0.851271 -2.391803 2.180228
H 0.280482 -0.739578 1.922725
H 1.994145 -1.138264 1.695976
C -1.809719 -1.694095 0.400193
C -3.189677 -2.150947 -0.060579
H -1.695412 -0.629628 0.170797
H -1.760318 -1.779343 1.491319
C -4.329383 -1.311627 0.528867
H -3.322585 -3.201893 0.223293
H -3.261515 -2.108712 -1.155856
C -4.706164 -0.063566 -0.273334
H -4.085021 -1.04455 1.564311
H -5.224097 -1.937963 0.593053
C -3.622071 0.993712 -0.480671
H -5.563298 0.409684 0.219998
H -5.069215 -0.384313 -1.257306
C -3.108753 1.633629 0.805624
H -4.036611 1.785142 -1.1167
H -2.796581 0.564924 -1.064568
C -2.073036 2.734145 0.591219
H -2.692011 0.872705 1.478794
H -3.964641 2.058244 1.342603
C -0.726875 2.257187 0.052342
H -1.897711 3.252784 1.540652
H -2.478875 3.48767 -0.095074
H -0.877525 1.776642 -0.928059
H -0.324214 1.503315 0.749654
C 2.467973 2.059814 0.020929
C 3.395964 1.248552 -0.501201
H 2.385047 2.11692 1.108998
C 4.382601 0.437665 0.279872
H 3.516706 1.231948 -1.588509
C 4.531084 -1.003468 -0.206366
H 4.124595 0.458617 1.345747
H 5.364025 0.924722 0.200314
C 5.593629 -1.776405 0.559512
H 4.773607 -0.999125 -1.276001
H 3.569903 -1.529768 -0.115081
H 6.571973 -1.298667 0.455903
H 5.685067 -2.801592 0.193262
H 5.360331 -1.821122 1.627862
N 0.824418 -0.635254 -0.752992
H 0.663574 -0.915797 -1.724835
H 1.725234 -0.132425 -0.647048
H 1.48718 -2.536452 -0.261365
H 0.103428 0.051584 -0.508834
C -0.648432 -2.48195 -0.189829
H -0.640676 -3.499611 0.222746
O -0.674796 -2.530683 -1.610013
H -1.46874 -2.977084 -1.913274
C 0.281897 3.388292 -0.105145
H 0.482288 3.82682 0.880102
H -0.180106 4.18427 -0.698643
```

C 1.606316 3.006936 -0.762949
H 2.184759 3.929431 -0.903556
H 1.436782 2.61714 -1.775502

conf_01

1 1
C 2.296905 2.399587 -0.84074
C 1.701243 2.469299 0.545592
H 2.099712 3.336461 -1.36656
H 1.865759 1.584626 -1.426981
H 3.379922 2.264246 -0.802914
C -0.693193 2.052283 -0.354075
C -2.156666 2.469604 -0.21531
H -0.588811 0.971199 -0.207938
H -0.36225 2.272803 -1.375396
C -3.045172 1.979884 -1.357819
H -2.207386 3.565872 -0.184552
H -2.565322 2.110891 0.738292
C -3.216141 0.466164 -1.45572
H -2.637038 2.361576 -2.301677
H -4.033505 2.440397 -1.248373
C -3.980095 -0.149477 -0.287327
H -2.235557 -0.013555 -1.568339
H -3.75746 0.242518 -2.382375
C -4.273061 -1.639753 -0.460662
H -4.929487 0.387427 -0.172695
H -3.433068 0.011808 0.651087
C -3.035847 -2.532176 -0.390305
H -4.776227 -1.787906 -1.423237
H -4.987879 -1.959508 0.306732
C -2.462797 -2.673486 1.022962
H -2.271437 -2.141357 -1.072771
H -3.280738 -3.527736 -0.774275
H -2.979053 -3.488679 1.539356
H -2.676127 -1.770365 1.609772
C 1.305042 -1.858183 0.68537
C 2.092895 -1.672905 -0.380579
H 1.756339 -2.253307 1.598827
C 3.555545 -1.975255 -0.437216
H 1.634434 -1.323154 -1.309432
C 4.427595 -0.774022 -0.803711
H 3.714746 -2.756448 -1.192232
H 3.885888 -2.400291 0.517298
C 5.893005 -1.142932 -0.975861
H 4.338745 -0.006823 -0.020106
H 4.048726 -0.316461 -1.726155
H 6.30087 -1.582876 -0.061029
H 6.499668 -0.269473 -1.22653
H 6.01726 -1.87517 -1.778633
N 1.913092 1.178963 1.294714
H 1.255425 1.19245 2.087938
H 2.865987 1.10571 1.648298

H 2.218943 3.221057 1.147672
H 1.742354 0.316867 0.737781
C 0.20662 2.802909 0.613552
H 0.128594 3.87907 0.4059
O -0.124494 2.551828 1.974577
H -1.047892 2.761237 2.136249
C -0.957703 -2.920245 1.051271
H -0.689883 -3.710183 0.339852
H -0.650517 -3.275462 2.041338
C -0.17403 -1.648814 0.713994
H -0.439728 -0.892888 1.470246
H -0.504571 -1.256831 -0.25455

conf_02

1 1
C 1.221561 3.782087 0.871392
C 0.979237 2.287871 0.933102
H 0.42749 4.308654 1.401956
H 1.230405 4.136171 -0.162268
H 2.166316 4.0518 1.35201
C -1.54329 2.534045 0.631862
C -2.812102 1.709454 0.415449
H -1.592919 3.482616 0.084825
H -1.47002 2.789156 1.695781
C -2.985485 1.150677 -0.99302
H -3.678084 2.333393 0.661084
H -2.821949 0.885565 1.137181
C -4.29099 0.385253 -1.201829
H -2.154575 0.471099 -1.237372
H -2.951799 1.976441 -1.718299
C -4.479137 -0.874533 -0.354882
H -4.359281 0.105614 -2.259574
H -5.129861 1.066153 -1.01478
C -3.416434 -1.96459 -0.542896
H -5.459468 -1.28605 -0.613932
H -4.548608 -0.613179 0.708443
C -2.255909 -1.905861 0.446081
H -3.029409 -1.931079 -1.570869
H -3.890319 -2.947129 -0.441036
C -1.240973 -3.025307 0.256136
H -2.658038 -1.952891 1.467566
H -1.751942 -0.935408 0.369018
H -0.775313 -2.93693 -0.734032
H -1.769663 -3.985164 0.245583
C 1.449041 -1.494528 0.235981
C 2.765938 -1.673063 0.071829
H 0.859581 -1.187573 -0.632294
C 3.517528 -1.530673 -1.217328
H 3.344242 -2.032134 0.926424
C 4.967769 -1.079324 -1.053746
H 2.976352 -0.864732 -1.903208
H 3.519826 -2.513366 -1.706853

C 5.128297 0.307311 -0.448945
H 5.456437 -1.105862 -2.032096
H 5.498172 -1.80851 -0.430854
H 4.637992 1.067004 -1.071426
H 6.180594 0.589845 -0.373534
H 4.717204 0.346002 0.568034
N 2.123614 1.583941 0.257599
H 2.101314 0.548358 0.385077
H 3.034215 1.924244 0.563845
H 0.95504 1.933236 1.96651
H 2.019079 1.753006 -0.750363
C -0.274907 1.809739 0.201496
H -0.389796 0.735979 0.411039
O 0.040797 1.984422 -1.175274
H -0.729236 1.777403 -1.711926
C -0.168656 -3.087187 1.341117
H 0.494798 -3.936544 1.146116
H -0.64759 -3.282187 2.307652
C 0.695704 -1.825235 1.487445
H 1.404758 -1.975464 2.308254
H 0.049236 -0.987309 1.781852

conf_03

1 1
C -2.582064 2.493654 0.72094
C -1.783391 2.790602 -0.526819
H -2.46712 3.316296 1.430177
H -2.248013 1.575503 1.209419
H -3.647932 2.402507 0.497207
C 0.448416 2.082012 0.578223
C 1.929658 2.432465 0.711018
H 0.331414 1.060124 0.199692
H -0.009883 2.111169 1.573474
C 2.648878 1.667214 1.820534
H 2.017818 3.506445 0.9211
H 2.448313 2.255936 -0.240245
C 2.780218 0.160471 1.611534
H 2.130391 1.858768 2.767991
H 3.652323 2.092523 1.934884
C 3.642012 -0.229757 0.413262
H 1.785124 -0.296589 1.536676
H 3.227692 -0.267386 2.516302
C 3.992663 -1.717113 0.3648
H 4.572563 0.349702 0.453322
H 3.148318 0.064245 -0.523055
C 2.810665 -2.632068 0.051547
H 4.431671 -2.002464 1.327939
H 4.776424 -1.879846 -0.3844
C 2.358511 -2.576545 -1.414663
H 1.974363 -2.379712 0.715329
H 3.073432 -3.663773 0.304682
H 2.838896 -3.385059 -1.973426

H 2.709058 -1.646757 -1.881889
C -1.319189 -1.408418 -1.176016
C -2.096629 -1.20762 -0.104211
H -1.796216 -1.673695 -2.123111
C -3.587083 -1.330247 -0.082468
H -1.606193 -0.992033 0.847183
C -4.082975 -2.477827 0.807628
H -3.956217 -1.475911 -1.104024
H -4.032081 -0.396251 0.29328
C -3.74514 -2.305002 2.281348
H -3.664627 -3.418646 0.433933
H -5.168596 -2.552673 0.688153
H -2.66636 -2.322728 2.462317
H -4.182871 -3.109662 2.876283
H -4.13797 -1.359254 2.670386
N -1.897411 1.664836 -1.520163
H -1.153038 1.812926 -2.21613
H -2.802986 1.666822 -1.987175
H -2.193568 3.660936 -1.046432
H -1.77212 0.716478 -1.099036
C -0.290161 3.062369 -0.318953
H -0.221533 4.067172 0.120212
O 0.224412 3.08103 -1.645492
H 1.165135 3.274694 -1.634078
C 0.845051 -2.661766 -1.58898
H 0.443795 -3.486621 -0.988428
H 0.590862 -2.881344 -2.631881
C 0.172684 -1.352974 -1.168961
H 0.526834 -0.565811 -1.853071
H 0.516735 -1.074809 -0.167054

conf_04

1 1
C 0.831574 -2.715341 -1.419629
C -0.257502 -1.853894 -0.819722
H 0.368636 -3.458526 -2.06761
H 1.387181 -3.252259 -0.643745
H 1.530241 -2.122992 -2.014702
C -2.397487 -1.846691 0.620873
C -3.363331 -1.229825 -0.385696
H -1.979485 -1.076152 1.28417
H -2.953444 -2.50437 1.301093
C -4.387734 -0.293082 0.254888
H -3.886699 -2.029677 -0.916541
H -2.81297 -0.684895 -1.161566
C -3.785388 0.879227 1.030188
H -5.038214 -0.867698 0.924205
H -5.036905 0.093909 -0.538112
C -2.783138 1.695227 0.227196
H -3.303731 0.518323 1.948931
H -4.595971 1.532613 1.369824
C -2.10133 2.798624 1.023273

H -3.27405 2.123 -0.656986
H -2.016183 1.020035 -0.179824
C -1.026385 3.548533 0.241879
H -1.65015 2.3654 1.928155
H -2.856426 3.507852 1.378802
C 0.094508 2.649494 -0.2666
H -0.595011 4.325296 0.883252
H -1.480108 4.072632 -0.608094
H -0.286868 1.987401 -1.057777
H 0.428427 2.007101 0.565691
C 2.844156 1.414212 -0.549653
C 3.179609 0.174677 -0.92853
H 3.006917 1.707202 0.48879
C 3.813894 -0.873535 -0.06201
H 3.067721 -0.08409 -1.984033
C 3.873672 -0.574151 1.429233
H 4.83271 -1.046047 -0.434973
H 3.309646 -1.837568 -0.22382
C 4.461795 -1.726776 2.229364
H 2.867456 -0.336504 1.809053
H 4.466952 0.330758 1.598277
H 3.872284 -2.642017 2.109659
H 4.501335 -1.492516 3.295458
H 5.481161 -1.949937 1.902328
N 0.361417 -0.8059 0.065279
H -0.318691 -0.093161 0.341215
H 1.159407 -0.30395 -0.385646
H -0.785663 -1.306044 -1.603855
H 0.736735 -1.229078 0.917287
C -1.260151 -2.664872 0.012837
H -0.68476 -3.130834 0.835246
O -1.719824 -3.654119 -0.87279
H -2.369771 -4.205242 -0.429743
C 1.299616 3.403298 -0.808595
H 1.765875 3.970644 0.006049
H 0.967395 4.143843 -1.543607
C 2.340497 2.492662 -1.457598
H 3.198924 3.105132 -1.762469
H 1.943514 2.049953 -2.379195

conf_05

1 1
C 1.929201 2.748757 -1.056766
C 1.506138 2.620819 0.387648
H 1.553172 3.691817 -1.459879
H 1.529764 1.938342 -1.672106
H 3.016594 2.761216 -1.155277
C -0.928336 2.049023 -0.296642
C -2.398221 2.31181 0.029751
H -0.719933 0.972991 -0.285481
H -0.729522 2.404801 -1.314064
C -3.357334 1.872843 -1.07635

H -2.533157 3.388548 0.196441
H -2.673609 1.814004 0.968467
C -3.410149 0.369298 -1.334306
H -3.077498 2.392214 -2.001012
H -4.363499 2.226383 -0.82437
C -4.054385 -0.428557 -0.205257
H -2.400407 -0.006877 -1.541075
H -3.980163 0.194473 -2.254006
C -4.094817 -1.934963 -0.460418
H -5.078056 -0.062687 -0.062817
H -3.536494 -0.230785 0.741954
C -2.724775 -2.609823 -0.433815
H -4.570473 -2.115401 -1.431456
H -4.74142 -2.410463 0.286467
C -2.117147 -2.697023 0.965758
H -2.047754 -2.075338 -1.110681
H -2.807508 -3.621304 -0.845869
H -2.598119 -3.515931 1.510905
H -2.346407 -1.787085 1.5356
C 1.635796 -1.797266 0.65522
C 2.497034 -1.865072 -0.365743
H 2.021904 -1.960501 1.664742
C 3.951167 -2.193877 -0.237901
H 2.110706 -1.755817 -1.382533
C 4.884674 -1.312043 -1.065953
H 4.086292 -3.234382 -0.56077
H 4.242467 -2.164554 0.819134
C 4.825137 0.159892 -0.687788
H 4.646051 -1.423867 -2.129912
H 5.908042 -1.678949 -0.944792
H 3.845473 0.581762 -0.937095
H 5.573067 0.74571 -1.227437
H 5.015189 0.297491 0.385186
N 1.943205 1.303774 0.97422
H 1.440727 1.197858 1.866527
H 2.949974 1.279198 1.136866
H 2.017226 3.367328 1.002062
H 1.736089 0.470337 0.399869
C 0.00492 2.778667 0.655502
H -0.198089 3.856541 0.586389
O -0.138515 2.349442 2.00413
H -1.047714 2.458568 2.293793
C -0.605875 -2.899086 0.976026
H -0.32528 -3.732742 0.321881
H -0.275941 -3.173116 1.98465
C 0.152696 -1.641804 0.536497
H -0.200527 -0.813704 1.171482
H -0.109829 -1.387608 -0.497132

conf_06

1 1
C 1.00333 4.051894 -0.412991

C 0.96684 2.548934 -0.233483
H 0.585298 4.538863 0.469031
H 0.414368 4.359229 -1.279978
H 2.029654 4.413105 -0.525735
C -1.270167 2.537366 0.984421
C -2.319752 1.565759 1.517826
H -1.731844 3.464959 0.626746
H -0.604352 2.817499 1.809636
C -3.360377 1.106894 0.501194
H -2.836351 2.033664 2.362278
H -1.800481 0.69243 1.931049
C -4.236285 -0.033058 1.021528
H -2.870336 0.73956 -0.412754
H -3.980921 1.960812 0.205241
C -3.519368 -1.382922 1.02394
H -5.139836 -0.109921 0.406772
H -4.575969 0.214334 2.033707
C -3.353244 -1.984863 -0.369137
H -4.079046 -2.091202 1.644271
H -2.540675 -1.279764 1.509802
C -2.4338 -3.203128 -0.406062
H -2.983171 -1.229589 -1.074429
H -4.342871 -2.270691 -0.742891
C -0.943626 -2.899383 -0.252496
H -2.579617 -3.738107 -1.351928
H -2.737501 -3.900121 0.383743
H -0.408849 -3.850504 -0.142004
H -0.766287 -2.350694 0.68143
C 1.81629 -1.298305 -0.364279
C 2.988379 -0.658911 -0.469475
H 1.300493 -1.264086 0.597347
C 3.684736 0.063524 0.643011
H 3.543853 -0.749311 -1.407873
C 4.96215 -0.656922 1.096571
H 3.00534 0.168421 1.498468
H 3.964953 1.07859 0.323725
C 4.691015 -2.020064 1.712694
H 5.478801 -0.017029 1.819156
H 5.638703 -0.759331 0.239872
H 4.035919 -1.935218 2.585936
H 5.620291 -2.490329 2.041908
H 4.210706 -2.696998 1.000592
N 1.592357 1.886335 -1.432665
H 1.834806 0.893655 -1.213145
H 2.434941 2.367208 -1.744706
H 1.554004 2.237237 0.633548
H 0.886565 1.902349 -2.180798
C -0.434579 1.947795 -0.144265
H -0.322447 0.864865 0.028365
O -0.972743 2.158313 -1.444985
H -1.893205 1.883633 -1.468218
C -0.338497 -2.149084 -1.438108
H -0.720366 -1.119985 -1.484199
H -0.67262 -2.632727 -2.363082

C 1.191661 -2.137629 -1.435176
H 1.57904 -1.831383 -2.413718
H 1.53622 -3.171642 -1.292902

conf_07

1 1
C -4.231393 -1.071489 -0.485085
C -2.924789 -0.340157 -0.264625
H -4.412303 -1.754215 0.34661
H -4.201818 -1.662196 -1.403053
H -5.075404 -0.377172 -0.525416
C -1.684731 -2.241456 0.905245
C -0.289788 -2.415577 1.500681
H -2.087333 -3.191834 0.537385
H -2.365556 -1.902142 1.694667
C 0.753435 -2.975685 0.53775
H -0.348687 -3.067825 2.377757
H 0.046269 -1.439748 1.876238
C 2.184077 -2.761243 1.035868
H 0.673514 -2.489292 -0.446091
H 0.554461 -4.039656 0.369068
C 2.722684 -1.366856 0.709758
H 2.849383 -3.509189 0.591603
H 2.211424 -2.934796 2.117553
C 3.159262 -1.229684 -0.745572
H 3.577499 -1.140076 1.356598
H 1.959971 -0.613333 0.953628
C 3.665193 0.156104 -1.137936
H 2.340647 -1.523349 -1.415812
H 3.959373 -1.955361 -0.932031
C 2.632388 1.280508 -1.077708
H 4.064702 0.104415 -2.157956
H 4.512637 0.422875 -0.495747
H 3.115444 2.209175 -1.403853
H 2.330015 1.459199 -0.040593
C -0.289201 2.721279 -0.994645
C -0.364463 2.139878 0.208636
H -0.838336 3.65358 -1.145031
C -1.106189 2.684554 1.387948
H 0.221377 1.239343 0.385568
C -0.173457 3.044643 2.551856
H -1.827384 1.940857 1.759195
H -1.68197 3.566535 1.087635
C 0.570598 1.85088 3.134127
H -0.774931 3.516764 3.335195
H 0.540917 3.801652 2.210796
H -0.125484 1.067887 3.455723
H 1.155538 2.145962 4.008068
H 1.268822 1.408104 2.41712
N -2.681446 0.619458 -1.402024
H -1.912369 1.283102 -1.153598
H -3.515217 1.151558 -1.648624

H -2.954475 0.264036 0.644656
H -2.382458 0.05614 -2.207715
C -1.687755 -1.230989 -0.238589
H -0.816705 -0.572148 -0.109795
O -1.646273 -1.795381 -1.543322
H -0.892861 -2.386683 -1.619437
C 1.412228 1.034671 -1.963134
H 0.818248 0.197097 -1.571254
H 1.761945 0.706001 -2.948755
C 0.520683 2.255554 -2.168568
H -0.159018 2.080164 -3.016597
H 1.137735 3.101796 -2.498997

conf_08

1 1
C 2.24289 2.115397 -1.297831
C 1.865739 2.46206 0.123176
H 2.062855 2.979721 -1.940987
H 1.654008 1.27891 -1.681616
H 3.301478 1.859137 -1.376774
C -0.655904 2.150967 -0.332193
C -2.056849 2.71139 -0.090768
H -0.600046 1.113689 0.016875
H -0.471901 2.135041 -1.412309
C -3.148428 1.920573 -0.809953
H -2.080649 3.756392 -0.423332
H -2.288729 2.716611 0.983866
C -3.367895 0.526956 -0.234624
H -2.890148 1.842852 -1.874386
H -4.085744 2.485182 -0.767985
C -4.298214 -0.338374 -1.074291
H -3.759791 0.613373 0.787901
H -2.403384 0.016841 -0.147327
C -4.454002 -1.774562 -0.573816
H -3.911542 -0.368909 -2.102251
H -5.284634 0.136112 -1.136194
C -3.12308 -2.526044 -0.43256
H -5.109238 -2.305705 -1.270553
H -4.978202 -1.777926 0.390388
C -2.565822 -2.538349 0.993742
H -2.392806 -2.083301 -1.123875
H -3.239717 -3.563213 -0.76296
H -3.106429 -3.294406 1.572569
H -2.778185 -1.582839 1.489185
C 1.238451 -1.821071 0.76928
C 2.090138 -1.764487 -0.261569
H 1.625697 -2.127996 1.74393
C 3.543422 -2.110136 -0.204023
H 1.693968 -1.506206 -1.247136
C 4.475272 -1.005349 -0.702599
H 3.705933 -2.997774 -0.830045
H 3.818484 -2.402639 0.815715

C 5.929949 -1.445526 -0.760038
H 4.392644 -0.128257 -0.042781
H 4.147526 -0.673551 -1.695684
H 6.287142 -1.76299 0.224056
H 6.579742 -0.639131 -1.108168
H 6.052508 -2.289848 -1.44423
N 2.059497 1.280027 1.040573
H 1.505072 1.475829 1.887274
H 3.038723 1.168011 1.300029
H 2.534725 3.229595 0.52156
H 1.764141 0.367387 0.640117
C 0.43567 2.97234 0.332159
H 0.414425 3.996223 -0.065357
O 0.307424 3.001675 1.749149
H -0.56286 3.32312 1.997872
C -1.066249 -2.812158 1.075978
H -0.797011 -3.6526 0.425439
H -0.795954 -3.110491 2.095252
C -0.234824 -1.584921 0.687188
H -0.524309 -0.76967 1.368773
H -0.495251 -1.262398 -0.327608

conf_09

1 1
C 1.062403 -3.289508 -0.72929
C 0.590506 -1.860005 -0.551711
H 0.421598 -3.809913 -1.441271
H 1.036063 -3.834658 0.217465
H 2.079133 -3.31921 -1.132298
C -1.875262 -2.533063 -0.487469
C -3.215328 -2.397014 0.243356
H -1.585698 -3.587851 -0.493959
H -1.980429 -2.222465 -1.533503
C -3.701325 -0.965581 0.459621
H -3.152463 -2.913712 1.209939
H -3.965834 -2.955696 -0.325694
C -3.994729 -0.213196 -0.834897
H -2.974379 -0.412835 1.066152
H -4.613755 -0.99479 1.065078
C -4.376398 1.262402 -0.67953
H -4.806986 -0.743301 -1.346542
H -3.133175 -0.272254 -1.514191
C -3.193333 2.228779 -0.579854
H -5.04267 1.389428 0.183319
H -4.968238 1.547101 -1.554921
C -2.560881 2.395511 0.802456
H -3.514179 3.22116 -0.916276
H -2.433695 1.909634 -1.306541
C -1.188027 3.061775 0.750456
H -2.459181 1.429063 1.309102
H -3.237032 2.985029 1.430458
H -0.914229 3.440034 1.741612

H -1.228737 3.936207 0.088609
C 2.25821 1.875362 -0.609968
C 3.487327 1.603523 -0.157509
H 1.972896 1.492238 -1.595351
C 4.548183 0.859576 -0.912007
H 3.790835 2.034544 0.799064
C 5.524835 0.072287 -0.040358
H 5.125918 1.602579 -1.477407
H 4.09152 0.208782 -1.670338
C 4.899356 -1.08597 0.721127
H 5.997773 0.757209 0.672469
H 6.331715 -0.307981 -0.673597
H 4.135266 -0.736113 1.426361
H 5.642301 -1.628681 1.309684
H 4.461824 -1.819446 0.026892
N 1.577162 -1.138114 0.331324
H 1.648493 -0.121134 0.151881
H 2.526465 -1.504532 0.233565
H 0.575204 -1.333531 -1.508885
H 1.262336 -1.289934 1.300769
C -0.768756 -1.705226 0.140133
H -1.045336 -0.643009 0.091775
O -0.510062 -2.055538 1.496613
H -1.319835 -1.998943 2.010153
C -0.099098 2.112414 0.263869
H -0.415115 1.649078 -0.681321
H -0.00237 1.298199 0.998731
C 1.261149 2.772338 0.054873
H 1.659699 3.137294 1.007541
H 1.121671 3.657085 -0.580472

ref_01

1 1
C 5.899096 -1.329528 -0.733279
H 5.547546 -2.329719 -0.459718
H 6.965818 -1.280908 -0.503161
H 5.78929 -1.221678 -1.816814
C 5.128653 -0.249758 0.009764
H 5.516676 0.740938 -0.249916
H 5.274128 -0.362036 1.090663
C 3.628624 -0.281239 -0.300893
H 3.261595 -1.291282 -0.066761
H 3.476969 -0.126703 -1.376268
C 2.871517 0.749565 0.478897
H 2.82801 0.605649 1.561611
C 2.381121 1.889604 -0.023315
H 2.47523 2.063383 -1.098453
C 1.793407 3.007894 0.778846
H 2.556294 3.792146 0.868088
H 1.592826 2.661964 1.800116
C 0.532787 3.621489 0.170262
H 0.778018 4.09627 -0.787517

H 0.176653 4.422649 0.826183
C -0.574962 2.60241 -0.04303
H -0.725669 2.052318 0.898214
H -0.23423 1.876186 -0.796535
C -1.898701 3.182132 -0.520588
H -1.715733 3.782953 -1.418527
H -2.291844 3.873237 0.235063
C -2.938267 2.109755 -0.833939
H -2.495651 1.3848 -1.532911
H -3.777495 2.55952 -1.375354
C -3.491554 1.391619 0.394667
H -4.096002 2.101767 0.96915
H -2.67753 1.101256 1.074233
C -4.335436 0.165005 0.050957
H -5.030619 0.431049 -0.75295
H -4.958383 -0.109285 0.910061
C -3.518209 -1.055172 -0.372802
H -2.790036 -0.769994 -1.140799
H -4.179925 -1.78388 -0.855837
C -2.832866 -1.749904 0.802296
H -3.609425 -2.02701 1.524309
H -2.201092 -1.045005 1.362714
C -2.058373 -3.025872 0.470253
H -2.726837 -3.711972 -0.06614
H -1.812312 -3.547824 1.402039
C -0.767154 -2.94222 -0.339935
H -0.345721 -3.956042 -0.378872
C 0.355372 -2.063191 0.221985
H 1.258709 -2.306907 -0.342952
O -0.939234 -2.448698 -1.662518
H -1.598015 -2.966399 -2.131483
N 0.06992 -0.637332 -0.149756
H -0.702706 -0.246175 0.392079
H -0.222031 -0.631904 -1.134007
H 0.895641 -0.017255 -0.020549
C 0.616524 -2.195318 1.704409
H 1.487919 -1.607867 2.002771
H 0.825602 -3.239823 1.945337
H -0.240714 -1.879142 2.303986

14Z-1-DeoxySO

conf_00

1 1
C -0.05805 -2.566949 -1.340882
C 0.298925 -2.29625 0.102658
H 0.001618 -3.640221 -1.534384
H 0.621689 -2.064908 -2.033324
H -1.079216 -2.249254 -1.560659
C 2.845151 -2.533057 -0.421535
H 2.670259 -3.171782 -1.294715
H 3.746319 -2.94664 0.049992

C 4.117 1.198786 -0.462359
C 2.931545 2.103361 -0.798244
H 4.714086 1.018024 -1.363503
H 4.776443 1.727178 0.234985
C 2.338704 2.798248 0.425664
H 2.161753 1.522021 -1.324829
H 3.245366 2.868657 -1.515947
C 0.973345 3.438263 0.189796
H 3.048511 3.55944 0.766433
H 2.250743 2.086828 1.259877
C -0.136422 2.408072 0.033302
H 1.008165 4.085579 -0.694926
H 0.728413 4.089545 1.03628
H -0.079637 1.728216 0.896868
H 0.038156 1.806677 -0.87249
C -2.611312 0.8859 0.909048
C -2.965787 -0.406771 0.845709
H -2.351837 1.281956 1.892419
C -3.482261 -1.147656 -0.349471
H -2.986475 -0.962807 1.784505
C -5.008457 -1.323175 -0.302445
H -3.030197 -2.147074 -0.383883
H -3.212521 -0.633367 -1.278992
C -5.769698 -0.010684 -0.397625
H -5.279643 -1.846455 0.621851
H -5.300153 -1.983631 -1.125573
H -5.530967 0.65334 0.43814
H -6.848069 -0.184259 -0.382186
H -5.533419 0.516726 -1.327571
N 0.192488 -0.835811 0.429523
H 0.682854 -0.698437 1.323548
H -0.792956 -0.513576 0.531719
H -0.428958 -2.770337 0.767299
H 0.652197 -0.244173 -0.263729
C 1.687599 -2.781131 0.541704
H 1.591719 -3.868811 0.659732
O 1.859688 -2.180357 1.819764
H 2.702617 -2.43663 2.20153
C -2.609082 1.890653 -0.204307
H -2.49128 1.396235 -1.175717
H -3.598935 2.363859 -0.234796
C -1.545721 2.974732 -0.033561
H -1.749057 3.543588 0.881989
H -1.620725 3.687765 -0.860814
C 3.722243 -0.150487 0.139272
H 4.607219 -0.619079 0.585915
H 3.022091 -5.1e-05 0.96999
C 3.149451 -1.112644 -0.899672
H 2.271421 -0.673752 -1.394356
H 3.884445 -1.202127 -1.707754

conf_01

1 1
C 1.924546 -3.116345 -0.756207
C 2.364901 -2.333359 0.457819
H 2.776362 -3.259877 -1.424598
H 1.14189 -2.595569 -1.312431
H 1.555771 -4.106989 -0.478828
C 2.245935 -0.071959 -0.787599
H 1.20145 0.030278 -0.467198
H 2.228512 -0.556172 -1.770684
C 0.709573 2.349433 -1.815419
C 0.25231 2.867938 -0.455951
H 0.228631 1.379392 -2.002367
H 0.336092 3.011253 -2.604237
C -1.253816 2.727711 -0.27143
H 0.556595 3.914113 -0.328659
H 0.76377 2.313129 0.339369
C -1.772483 2.967358 1.144389
H -1.542296 1.718059 -0.592048
H -1.771559 3.404775 -0.959966
C -1.102161 2.129471 2.237947
H -2.852603 2.781761 1.150163
H -1.655484 4.024574 1.407204
H -1.6747 2.239791 3.165751
H -0.107931 2.536368 2.45517
C -1.993286 -1.550009 1.301082
C -1.768996 -2.094777 0.09576
H -2.012894 -2.221162 2.161191
C -1.771804 -1.391464 -1.226012
H -1.656479 -3.179963 0.046339
C -3.024434 -1.711857 -2.053585
H -0.88965 -1.699382 -1.802658
H -1.693089 -0.308388 -1.090696
C -4.299782 -1.148654 -1.446586
H -3.111466 -2.798904 -2.168058
H -2.882646 -1.308054 -3.06128
H -4.474128 -1.540732 -0.440277
H -5.169477 -1.404743 -2.055943
H -4.255228 -0.056563 -1.379922
N 1.212591 -2.104639 1.403522
H 1.482164 -1.315951 2.010747
H 1.038601 -2.924034 1.983671
H 3.089894 -2.906441 1.041928
H 0.308634 -1.875484 0.943911
C 3.001838 -0.96734 0.182068
H 4.004778 -1.16908 -0.217837
O 3.107234 -0.396599 1.481191
H 3.51407 0.471979 1.430477
C -2.237816 -0.099673 1.592267
H -2.729895 0.377238 0.741401
H -2.929003 -0.015712 2.437517
C -0.941211 0.644475 1.921273
H -0.259714 0.532644 1.06729
H -0.454353 0.158666 2.779066
C 2.220535 2.199052 -1.973217

H 2.434399 1.793637 -2.968989
H 2.697508 3.184589 -1.938357
C 2.883547 1.309006 -0.920538
H 3.945141 1.193909 -1.1726
H 2.84795 1.828257 0.04589

conf_02

1 1
C 0.043273 3.590438 -1.554165
C -0.343929 2.167453 -1.207996
H 1.120055 3.65244 -1.714294
H -0.222535 4.279443 -0.749193
H -0.437459 3.915706 -2.481526
C 1.68121 1.739899 0.268957
H 2.012055 2.780591 0.187251
H 2.108408 1.196429 -0.581946
C 4.261543 -0.046941 0.615214
C 3.892354 -1.496847 0.945462
H 5.351394 0.046116 0.574206
H 3.919514 0.207914 -0.394549
C 3.774008 -2.404854 -0.27852
H 2.952811 -1.529353 1.508955
H 4.644184 -1.909949 1.62562
C 2.604528 -2.071949 -1.205648
H 3.676785 -3.444868 0.054495
H 4.706378 -2.357887 -0.853204
C 1.233155 -2.246521 -0.56547
H 2.666249 -2.703916 -2.09895
H 2.701139 -1.04018 -1.570674
H 1.195089 -1.704817 0.388132
H 1.075952 -3.302772 -0.313122
C -2.386962 -1.233163 -1.50933
C -3.430823 -0.566759 -0.990545
H -2.365469 -1.3707 -2.590962
C -3.722958 -0.347066 0.463337
H -4.202772 -0.231435 -1.684638
C -4.731551 -1.366831 1.006337
H -4.142728 0.657712 0.612281
H -2.808166 -0.400768 1.06526
C -5.073537 -1.121483 2.46708
H -4.315827 -2.371932 0.876857
H -5.640866 -1.330704 0.396086
H -4.182592 -1.182903 3.100162
H -5.789396 -1.86126 2.832409
H -5.518616 -0.131918 2.610868
N -1.843914 2.073672 -1.11551
H -2.182461 1.085959 -1.18251
H -2.305303 2.617408 -1.844015
H -0.037574 1.47521 -1.99581
H -2.108691 2.445152 -0.193815
C 0.17294 1.666156 0.141219
H -0.130737 0.612532 0.246079

O -0.531129 2.449868 1.096528
H -0.245549 2.221687 1.98454
C -1.270171 -1.881695 -0.756627
H -1.214537 -1.49447 0.267471
H -1.507976 -2.948844 -0.647799
C 0.089922 -1.754538 -1.440422
H 0.275662 -0.704226 -1.706737
H 0.076342 -2.300535 -2.391228
C 3.716438 0.976695 1.609977
H 4.012967 0.687437 2.624328
H 4.179946 1.953111 1.425454
C 2.195074 1.137594 1.574014
H 1.71767 0.162617 1.736799
H 1.896002 1.773793 2.416825

conf_03

1 1
C -0.855225 -2.87261 2.111762
C -0.903153 -1.522068 1.427698
H 0.171475 -3.108919 2.392828
H -1.219176 -3.662995 1.451406
H -1.446755 -2.870393 3.032076
C 1.250678 -1.845261 0.118738
H 1.351106 -2.882406 0.455953
H 1.727974 -1.21116 0.874935
C 4.189362 -0.779058 -0.304526
C 4.182962 0.613009 -0.944194
H 5.224654 -1.093188 -0.138133
H 3.744359 -0.736065 0.696273
C 4.221273 1.762567 0.062657
H 3.304121 0.729076 -1.588736
H 5.04121 0.699045 -1.618425
C 2.961838 1.896204 0.919444
H 4.386911 2.704309 -0.473672
H 5.08742 1.634571 0.72234
C 1.705114 2.240201 0.129519
H 3.127805 2.664843 1.68276
H 2.791572 0.964311 1.476032
H 1.602291 1.557745 -0.723704
H 1.80448 3.243534 -0.303309
C -2.098162 2.130495 0.854445
C -3.264074 1.772719 0.294039
H -2.07475 2.25694 1.93834
C -3.54462 1.65143 -1.173736
H -4.114975 1.627418 0.960378
C -3.807747 0.221544 -1.661582
H -2.725846 2.08473 -1.753125
H -4.429891 2.261286 -1.393783
C -5.000129 -0.457594 -1.000512
H -2.897602 -0.381091 -1.532764
H -3.975894 0.255342 -2.742467
H -4.898488 -0.532841 0.089052

H -5.151518 -1.466883 -1.392136
H -5.920074 0.104624 -1.184449
N -2.338175 -1.15299 1.165502
H -2.44421 -0.138667 0.937499
H -2.938467 -1.366813 1.961262
H -0.497145 -0.740528 2.074327
H -2.652705 -1.694622 0.350757
C -0.214667 -1.461264 0.064188
H -0.286414 -0.424018 -0.30121
O -1.005729 -2.302061 -0.765184
H -0.608833 -2.370241 -1.637021
C -0.823485 2.454132 0.1364
H -0.775066 1.915653 -0.817398
H -0.830582 3.519551 -0.131123
C 0.430798 2.16181 0.956428
H 0.360915 1.156569 1.395963
H 0.484518 2.851481 1.807285
C 3.477431 -1.848185 -1.131943
H 3.888261 -1.851333 -2.147705
H 3.691824 -2.839477 -0.715197
C 1.960312 -1.668308 -1.220812
H 1.728346 -0.674671 -1.625339
H 1.571169 -2.395879 -1.944883

conf_04

1 1
C -3.585308 0.599922 -0.11141
C -2.688572 1.034795 -1.245296
H -4.154997 1.459293 0.249188
H -3.016675 0.194046 0.727517
H -4.305396 -0.153544 -0.440345
C -0.877249 1.989644 0.322683
H -0.341025 1.033337 0.27423
H -1.548255 1.933721 1.18737
C 1.637033 1.636612 1.988466
C 2.548169 1.244373 0.830507
H 0.882773 0.849897 2.126793
H 2.212495 1.647991 2.920295
C 2.977405 -0.215384 0.90308
H 3.428834 1.89806 0.805589
H 2.028135 1.414244 -0.119731
C 3.724998 -0.732916 -0.323626
H 2.079364 -0.827198 1.066788
H 3.599081 -0.370927 1.791869
C 2.986732 -0.557202 -1.654739
H 3.956718 -1.792575 -0.167856
H 4.695154 -0.229832 -0.402276
H 3.521612 -1.113366 -2.432736
H 3.027058 0.4939 -1.962069
C -0.166843 -2.793822 -1.201129
C -0.91462 -2.925531 -0.095518
H -0.656347 -2.966306 -2.162253

C -0.450227 -2.807953 1.322031
H -1.959309 -3.208851 -0.22984
C -1.210843 -1.762804 2.144608
H 0.618344 -2.584063 1.355471
H -0.576187 -3.785859 1.805675
C -2.678411 -2.096599 2.372552
H -1.110012 -0.781532 1.65935
H -0.71271 -1.660535 3.114086
H -3.239729 -2.18292 1.436688
H -3.169739 -1.33388 2.98286
H -2.78079 -3.05007 2.898695
N -1.872059 -0.120729 -1.768634
H -1.097689 0.286394 -2.314773
H -2.42336 -0.72576 -2.375726
H -3.283289 1.357778 -2.10409
H -1.460198 -0.734663 -1.044946
C -1.710775 2.171244 -0.935607
H -2.314966 3.084261 -0.846856
O -0.911442 2.231103 -2.111013
H -0.255675 2.92836 -2.03175
C 1.290604 -2.45868 -1.271897
H 1.780598 -2.679362 -0.321361
H 1.769912 -3.09763 -2.022201
C 1.523223 -0.991146 -1.640159
H 0.976119 -0.365413 -0.92173
H 1.088542 -0.791956 -2.630346
C 0.93727 2.984368 1.825324
H 0.278431 3.148754 2.685873
H 1.676761 3.792055 1.851203
C 0.121091 3.123575 0.538735
H -0.415574 4.079826 0.557771
H 0.815873 3.175637 -0.309518

conf_05

1 1
C 0.332774 3.359409 1.906919
C -0.172262 2.697053 0.644734
H 0.787807 4.320626 1.658923
H 1.088541 2.753043 2.41352
H -0.48293 3.556568 2.607445
C 2.121704 1.758175 -0.008121
H 1.842849 0.823011 0.494062
H 2.650606 2.368799 0.73234
C 4.028527 -0.608112 0.101797
C 3.168532 -1.633571 -0.630316
H 3.562713 -0.340257 1.057967
H 4.982186 -1.075509 0.371207
C 2.775868 -2.833061 0.230297
H 3.7146 -1.988784 -1.511905
H 2.259791 -1.158751 -1.023422
C 1.83569 -2.507534 1.392954
H 3.682718 -3.293887 0.638252

H 2.317602 -3.597154 -0.406715
C 0.484347 -1.921932 0.984468
H 2.33158 -1.818242 2.086149
H 1.657025 -3.42298 1.968468
H 0.646175 -1.011051 0.388977
H -0.060352 -1.627866 1.895322
C -1.795942 -1.121302 -1.000902
C -2.649221 -0.087981 -0.973937
H -1.051167 -1.13156 -1.797493
C -3.797589 0.103153 -0.030984
H -2.551348 0.659983 -1.760816
C -5.142097 -0.254319 -0.675807
H -3.848853 1.156164 0.285911
H -3.671672 -0.500356 0.875825
C -6.318032 -0.010427 0.256192
H -5.112007 -1.305472 -0.982064
H -5.263379 0.32903 -1.59546
H -6.231788 -0.606527 1.170224
H -7.262327 -0.277515 -0.223665
H -6.382724 1.042637 0.548356
N -0.763385 1.351558 0.966501
H -1.105967 0.85807 0.10809
H -1.552511 1.436034 1.606964
H -0.99949 3.266078 0.212126
H -0.076624 0.729419 1.398754
C 0.868216 2.498833 -0.455778
H 1.14373 3.503755 -0.805677
O 0.154596 1.805938 -1.464967
H 0.714718 1.689893 -2.236127
C -1.791251 -2.323119 -0.107016
H -2.316917 -2.118697 0.832939
H -2.376036 -3.107631 -0.605592
C -0.395293 -2.871724 0.180889
H 0.094855 -3.115557 -0.768697
H -0.48944 -3.816493 0.727133
C 4.318431 0.663702 -0.694112
H 4.942159 1.329237 -0.086127
H 4.913782 0.414337 -1.579256
C 3.073445 1.42563 -1.152036
H 3.381743 2.351225 -1.652909
H 2.552182 0.82073 -1.904263

conf_06

1 1
C -0.230666 3.786377 1.797759
C -0.157117 2.71361 0.733427
H 0.124178 4.726045 1.375313
H 0.407645 3.542385 2.653515
H -1.255959 3.936455 2.149196
C 1.354343 1.405396 -0.860462
H 0.696087 1.636709 -1.707235
H 0.988496 0.47164 -0.420028

C 2.532073 -1.399015 -1.38838
C 3.266761 -1.633953 -0.071474
H 2.710845 -2.257533 -2.045541
H 1.448784 -1.395806 -1.216096
C 2.805187 -2.881601 0.695491
H 3.163098 -0.758467 0.584817
H 4.338278 -1.706571 -0.288592
C 1.836117 -2.596025 1.844163
H 3.675338 -3.387144 1.12418
H 2.365574 -3.602669 -0.003923
C 0.511044 -1.948067 1.448787
H 2.344847 -1.947123 2.56804
H 1.623172 -3.53014 2.377073
H 0.724089 -1.034952 0.873635
H -0.017627 -1.636218 2.362185
C -1.591159 -1.046483 -0.699368
C -2.350515 0.055502 -0.797459
H -0.811718 -1.177587 -1.450983
C -3.53793 0.418103 0.042108
H -2.143934 0.729777 -1.630053
C -4.86256 0.207901 -0.707834
H -3.479042 1.479896 0.323949
H -3.560612 -0.168606 0.968112
C -5.138881 -1.251335 -1.03285
H -4.849171 0.801817 -1.628937
H -5.671643 0.613658 -0.092153
H -4.365683 -1.671726 -1.682423
H -6.096207 -1.360259 -1.547266
H -5.182625 -1.857861 -0.122407
N -0.635522 1.407679 1.309866
H -0.927542 0.728429 0.567035
H -1.450497 1.562319 1.904445
H -0.83733 2.939151 -0.09337
H 0.085404 0.962072 1.881512
C 1.253296 2.51722 0.172018
H 1.916617 2.269703 1.023743
O 1.587244 3.778622 -0.348572
H 2.458678 3.736748 -0.749964
C -1.740273 -2.183829 0.264496
H -2.278956 -1.872578 1.16725
H -2.384379 -2.933218 -0.21436
C -0.415271 -2.84527 0.63845
H 0.089834 -3.173791 -0.277001
H -0.620559 -3.753799 1.214159
C 2.947515 -0.130855 -2.133611
H 3.998206 -0.218204 -2.43138
H 2.374508 -0.060404 -3.065726
C 2.782323 1.167329 -1.34192
H 3.455983 1.154672 -0.475673
H 3.108786 2.000413 -1.975561

conf_07

1 1
C 0.130959 3.566869 -1.57563
C -0.294963 2.17677 -1.150383
H 1.194283 3.570567 -1.817009
H -0.04085 4.294094 -0.778761
H -0.402748 3.88496 -2.476112
C 1.813776 1.700465 0.188174
H 2.184764 2.720156 0.040525
H 2.150553 1.10589 -0.66913
C 4.334566 -0.187452 0.416731
C 3.928062 -1.606299 0.827538
H 5.421589 -0.145996 0.2951
H 3.931674 0.047418 -0.575206
C 3.685391 -2.551329 -0.349044
H 3.030067 -1.576145 1.455203
H 4.707655 -2.02828 1.469964
C 2.469142 -2.199008 -1.206375
H 3.567066 -3.573142 0.030267
H 4.576312 -2.567803 -0.987621
C 1.140072 -2.286695 -0.466772
H 2.440332 -2.865434 -2.075973
H 2.584641 -1.186864 -1.617979
H 1.194173 -1.712278 0.466663
H 0.953793 -3.325257 -0.165543
C -2.488247 -1.132357 -1.190259
C -3.461025 -0.405685 -0.616846
H -2.548109 -1.295678 -2.266882
C -3.620955 -0.136952 0.852474
H -4.261837 -0.048958 -1.264464
C -4.48265 -1.194773 1.558369
H -4.095191 0.84308 0.996576
H -2.643642 -0.095396 1.347824
C -5.900656 -1.284423 1.015579
H -4.508607 -0.955605 2.626621
H -3.98605 -2.167274 1.468422
H -6.414086 -0.31971 1.085198
H -6.488174 -2.010404 1.581943
H -5.914926 -1.599209 -0.031893
N -1.786552 2.159767 -0.944701
H -2.173774 1.187798 -0.951374
H -2.27545 2.701113 -1.656847
H -0.08139 1.444741 -1.932851
H -1.964674 2.573654 -0.020304
C 0.298373 1.69532 0.174456
H -0.044268 0.661487 0.339482
O -0.295288 2.542915 1.149927
H 0.050899 2.335388 2.021295
C -1.351785 -1.811657 -0.497186
H -1.207488 -1.397683 0.507951
H -1.630165 -2.862332 -0.336512
C -0.039199 -1.770285 -1.277431
H 0.173768 -0.738923 -1.592904
H -0.14432 -2.345322 -2.205138
C 3.907894 0.894308 1.407791

H 4.265675 0.62818 2.408632
H 4.397639 1.841614 1.152766
C 2.396267 1.122662 1.475328
H 1.890756 0.177106 1.710548
H 2.18816 1.801895 2.311943

conf_08

1 1
C -0.65991 -3.204135 -1.637298
C -0.879024 -2.332826 -0.420463
H -0.389294 -4.213757 -1.321382
H 0.144147 -2.82732 -2.275131
H -1.571267 -3.283042 -2.235879
C 1.613975 -1.867794 -0.025742
H 1.526185 -0.864902 -0.465216
H 1.874669 -2.555551 -0.838375
C 4.141944 -0.177103 -0.362996
C 3.846659 1.035502 0.514734
H 3.445127 -0.208584 -1.209641
H 5.132215 -0.053177 -0.814639
C 3.60088 2.31726 -0.278337
H 4.688856 1.187533 1.198991
H 2.980374 0.841634 1.15871
C 2.27021 2.359756 -1.02953
H 4.420147 2.460163 -0.992524
H 3.640455 3.175674 0.402384
C 1.046072 2.363434 -0.119931
H 2.205077 1.519847 -1.735944
H 2.247911 3.263268 -1.649733
H 1.151144 3.171603 0.616016
H 1.007918 1.435055 0.464038
C -1.786495 1.603376 0.94101
C -2.963413 0.980398 1.100268
H -0.973302 1.299108 1.598578
C -4.248306 1.282789 0.388199
H -3.021796 0.21585 1.877076
C -5.182708 0.082764 0.251796
H -4.052911 1.712676 -0.601144
H -4.770258 2.067572 0.951532
C -4.604194 -1.055011 -0.577113
H -6.119883 0.41871 -0.201359
H -5.443452 -0.291444 1.248363
H -4.294206 -0.699401 -1.569536
H -5.336246 -1.849982 -0.736325
H -3.746943 -1.51278 -0.069136
N -1.193165 -0.926089 -0.845527
H -1.429328 -0.309447 -0.035014
H -2.002681 -0.905441 -1.467656
H -1.765007 -2.659098 0.130389
H -0.403627 -0.497896 -1.332274
C 0.278292 -2.283145 0.572753
H 0.367401 -3.297757 0.986564

O -0.1703 -1.381658 1.571009
H 0.467062 -1.352397 2.288729
C -1.495673 2.74195 0.013143
H -2.366335 2.964371 -0.609173
H -1.33458 3.636664 0.630302
C -0.261512 2.560947 -0.876736
H -0.170806 3.446774 -1.515005
H -0.422232 1.727108 -1.579786
C 4.105344 -1.512615 0.376443
H 4.396017 -2.313241 -0.314049
H 4.857764 -1.510868 1.17283
C 2.749944 -1.862279 0.992387
H 2.815875 -2.850979 1.46244
H 2.527864 -1.150271 1.797064

conf_09

1 1
C -0.278424 -2.903445 0.400265
C -0.671112 -2.052184 -0.785158
H -0.53745 -3.945318 0.199555
H -0.792841 -2.601537 1.315386
H 0.797669 -2.858869 0.577791
C -3.165824 -1.998772 -0.042639
H -3.108707 -2.935654 0.523002
H -4.163319 -2.007386 -0.500878
C -3.130499 1.67491 1.370133
C -1.66321 2.086517 1.490308
H -3.507912 1.366254 2.351866
H -3.709135 2.562118 1.091526
C -1.157036 2.911095 0.308885
H -1.022906 1.204491 1.663945
H -1.538764 2.687092 2.398111
C 0.273639 3.423061 0.478719
H -1.830247 3.768021 0.196006
H -1.250475 2.353167 -0.633776
C 1.380081 2.439073 0.100411
H 0.407873 3.73644 1.520115
H 0.407875 4.328431 -0.123485
H 1.201083 1.460256 0.569849
H 2.323207 2.790269 0.534727
C 2.581746 -0.034141 -1.680605
C 2.980138 -0.835655 -0.681719
H 2.101972 -0.50796 -2.539967
C 3.688432 -0.426558 0.569593
H 2.809186 -1.906486 -0.803214
C 3.067284 -1.020832 1.839074
H 3.716934 0.662266 0.647017
H 4.732859 -0.761306 0.505343
C 3.31029 -2.514604 2.002828
H 1.98892 -0.804941 1.853718
H 3.481577 -0.49639 2.705616
H 2.939107 -3.098876 1.154372

H 2.827029 -2.899832 2.904226
H 4.38052 -2.723304 2.088198
N -0.29289 -0.615391 -0.576077
H 0.737446 -0.479222 -0.642047
H -0.625191 -0.24994 0.318097
H -0.096845 -2.350215 -1.667333
H -0.749672 -0.070016 -1.316946
C -2.150275 -2.105231 -1.178649
H -2.292448 -3.084787 -1.654494
O -2.266852 -1.081159 -2.15938
H -3.156198 -1.062173 -2.520805
C 2.790901 1.446044 -1.802246
H 3.656337 1.761368 -1.211983
H 3.041738 1.656392 -2.846774
C 1.582161 2.304861 -1.406559
H 0.675206 1.92634 -1.899855
H 1.730205 3.310019 -1.815904
C -3.397188 0.556648 0.363931
H -4.453045 0.566562 0.071423
H -2.842237 0.746202 -0.561131
C -3.076342 -0.824641 0.932026
H -2.100181 -0.818701 1.438912
H -3.782705 -1.022641 1.745764

conf_10

1 1
C -3.863556 -0.346929 -1.631993
C -2.617509 0.162091 -0.939265
H -4.400665 -1.027543 -0.970348
H -3.61457 -0.888185 -2.547476
H -4.546769 0.473372 -1.870913
C -2.013973 -2.043307 0.182916
H -2.807337 -2.571619 -0.355893
H -2.462673 -1.63469 1.097796
C 1.232768 -1.642919 1.166206
C 2.006754 -2.120826 -0.062102
H 1.932664 -1.564751 2.007128
H 0.864707 -0.620956 1.015172
C 3.436255 -1.5911 -0.155291
H 1.467149 -1.864531 -0.985809
H 2.055449 -3.216464 -0.043392
C 3.60113 -0.079279 -0.303609
H 3.937862 -2.079114 -0.999435
H 3.975727 -1.915212 0.742803
C 3.063629 0.499774 -1.62102
H 4.669527 0.14221 -0.225665
H 3.140086 0.428815 0.552678
H 3.042567 -0.291333 -2.379786
H 3.757232 1.256485 -2.003945
C 0.319491 3.048533 -0.565538
C -0.4434 2.987162 0.536775
H -0.05881 3.6286 -1.410468

C -0.089352 2.342873 1.838784
H -1.39945 3.515241 0.524377
C -1.184722 1.442242 2.408334
H 0.108123 3.143717 2.564941
H 0.841564 1.774776 1.753904
C -0.817624 0.869602 3.768451
H -1.372547 0.616153 1.711261
H -2.121714 2.008348 2.482333
H 0.101539 0.278112 3.711452
H -1.60841 0.223076 4.156917
H -0.652886 1.666772 4.498837
N -1.937992 1.182606 -1.81441
H -1.248362 1.741609 -1.266943
H -2.601179 1.819256 -2.254741
H -2.863873 0.672168 -0.004991
H -1.427787 0.658952 -2.53819
C -1.540177 -0.888372 -0.676721
H -0.712001 -0.379328 -0.166718
O -1.116601 -1.282119 -1.975425
H -0.40065 -1.919086 -1.903729
C 1.68365 2.45481 -0.739671
H 2.315184 3.174839 -1.272078
H 2.15398 2.300546 0.234104
C 1.679084 1.137289 -1.521289
H 1.288192 1.317139 -2.53221
H 0.991104 0.434894 -1.035645
C 0.106143 -2.584679 1.606282
H 0.583081 -3.490172 1.998077
H -0.425219 -2.140989 2.457589
C -0.919562 -3.040601 0.564128
H -0.409269 -3.408405 -0.336029
H -1.426993 -3.922169 0.967047

conf_11

1 1
C -1.195543 -2.824917 -1.317738
C -0.877484 -2.662043 0.150512
H -0.803492 -3.780299 -1.673298
H -0.747362 -2.030553 -1.920037
H -2.274293 -2.827943 -1.491853
C 1.55305 -1.94744 -0.346534
H 1.1911 -0.915861 -0.437519
H 1.54351 -2.374204 -1.356007
C 3.585578 0.139744 -1.203038
C 3.320077 1.132231 -0.075613
H 2.686875 0.072115 -1.831466
H 4.365636 0.534965 -1.862483
C 2.63048 2.395704 -0.578288
H 4.258715 1.38842 0.430697
H 2.693719 0.659783 0.690523
C 2.096624 3.327812 0.507273
H 1.802103 2.097398 -1.236423

H 3.318775 2.955294 -1.221257
C 1.127623 2.676934 1.501602
H 1.609277 4.179802 0.020031
H 2.931605 3.755293 1.073274
H 0.631344 3.462482 2.082374
H 1.692298 2.08306 2.229156
C -1.470623 1.440504 -1.082506
C -2.753891 1.061373 -1.137022
H -0.788313 1.026027 -1.827974
C -3.869499 1.565018 -0.267896
H -3.042533 0.367256 -1.928348
C -5.068025 0.622048 -0.188962
H -4.211502 2.527363 -0.670406
H -3.500446 1.783389 0.741689
C -4.751248 -0.739671 0.413862
H -5.486996 0.483588 -1.192146
H -5.852383 1.101869 0.403403
H -4.082672 -1.314827 -0.24033
H -5.652537 -1.343432 0.540677
H -4.29813 -0.632298 1.409621
N -1.388831 -1.342615 0.665604
H -0.861698 -1.13307 1.525399
H -2.387927 -1.380021 0.877527
H -1.404543 -3.413827 0.743944
H -1.282489 -0.556566 0.000594
C 0.605696 -2.75538 0.527882
H 0.875923 -3.81829 0.468324
O 0.619343 -2.331892 1.886062
H 1.512996 -2.369107 2.236005
C -0.858178 2.434504 -0.143779
H -0.286937 3.150087 -0.745438
H -1.623474 3.016013 0.376939
C 0.079123 1.7662 0.868054
H 0.600909 0.940468 0.367604
H -0.524737 1.314397 1.667444
C 3.987167 -1.262222 -0.750912
H 4.129411 -1.891304 -1.637254
H 4.958449 -1.226573 -0.245582
C 2.984373 -1.941756 0.184325
H 3.311099 -2.974535 0.360254
H 3.013989 -1.43478 1.15727

conf_12

1 1
C 3.935055 -0.378876 0.03315
C 3.071851 -1.320319 -0.773183
H 4.51018 -0.951847 0.763763
H 3.339843 0.359959 0.57359
H 4.650058 0.14827 -0.603641
C 1.178937 -1.453351 0.965052
H 0.572676 -0.742527 0.390167
H 1.776177 -0.862294 1.667251

C -1.85129 -0.926688 1.650718
C -2.531906 -1.828497 0.625413
H -1.38434 -0.071955 1.145743
H -2.6153 -0.492625 2.306305
C -3.801771 -1.238509 0.017175
H -2.798223 -2.775762 1.112022
H -1.829145 -2.095602 -0.175021
C -3.665136 0.084859 -0.735642
H -4.532595 -1.098536 0.822893
H -4.242501 -1.979211 -0.661009
C -2.826612 0.039101 -2.018279
H -3.294857 0.866357 -0.061879
H -4.676696 0.402681 -1.007251
H -3.2127 0.787803 -2.719902
H -2.975525 -0.930696 -2.508327
C 0.446909 2.047548 -1.429575
C 1.305691 2.291 -0.427982
H 0.816201 2.175566 -2.450009
C 1.016135 2.289212 1.03995
H 2.311328 2.618401 -0.700603
C 0.904394 3.712875 1.605349
H 1.826609 1.770783 1.567557
H 0.096471 1.738342 1.260258
C -0.307644 4.469814 1.085624
H 1.821241 4.266663 1.371116
H 0.85941 3.643644 2.697013
H -0.28097 4.575237 -0.002866
H -0.353775 5.47392 1.513273
H -1.237427 3.955986 1.351017
N 2.235939 -0.565525 -1.773274
H 1.517447 -1.21973 -2.114388
H 2.792283 -0.236482 -2.560976
H 3.691792 -1.991707 -1.373472
H 1.757782 0.264376 -1.360281
C 2.106749 -2.200813 0.020556
H 2.7225 -2.91232 0.587048
O 1.389616 -2.881293 -1.0031
H 0.7351 -3.467636 -0.615281
C -0.999925 1.688528 -1.321383
H -1.340952 1.782128 -0.288685
H -1.57107 2.428258 -1.897633
C -1.326367 0.295463 -1.867256
H -0.874272 -0.464741 -1.217937
H -0.860457 0.183069 -2.856607
C -0.827363 -1.647061 2.528276
H -0.367267 -0.930755 3.219777
H -1.354073 -2.37623 3.15361
C 0.267918 -2.383005 1.760691
H 0.877349 -2.965558 2.460589
H -0.214553 -3.11389 1.098565

conf_13

1 1
C -3.017722 2.356775 -0.117337
C -1.759712 2.701753 -0.878867
H -3.23089 3.147471 0.605322
H -2.920409 1.415712 0.427584
H -3.87952 2.284823 -0.785712
C -0.146447 1.819019 0.921613
H 0.014222 0.894373 0.353829
H -0.990698 1.633471 1.594065
C 2.095283 -0.238868 1.740693
C 3.201251 0.132164 0.757456
H 1.264169 -0.715927 1.206356
H 2.472932 -1.004184 2.428867
C 3.975519 -1.06311 0.207566
H 3.915287 0.793904 1.264631
H 2.792387 0.721433 -0.074099
C 3.178634 -2.114446 -0.564562
H 4.474222 -1.561881 1.047454
H 4.779171 -0.692583 -0.440249
C 2.563718 -1.644832 -1.888035
H 2.410491 -2.556023 0.080997
H 3.866842 -2.936049 -0.786763
H 2.519092 -2.494803 -2.579021
H 3.237302 -0.917259 -2.356817
C -1.300998 -1.549977 -1.510203
C -2.208351 -1.281823 -0.558928
H -1.619687 -1.460521 -2.551517
C -2.023485 -1.438964 0.919127
H -3.210741 -1.00573 -0.88871
C -2.579366 -2.771024 1.443525
H -2.539849 -0.62243 1.441143
H -0.965671 -1.361487 1.185678
C -4.074608 -2.942686 1.221246
H -2.355943 -2.832984 2.513692
H -2.034304 -3.591388 0.963468
H -4.640323 -2.12306 1.677198
H -4.429678 -3.873956 1.668106
H -4.327519 -2.976687 0.157501
N -1.411093 1.61952 -1.867003
H -0.443162 1.7958 -2.171345
H -2.026788 1.634878 -2.678751
H -1.910754 3.598961 -1.485207
H -1.460471 0.66213 -1.455754
C -0.505238 2.941578 -0.038994
H -0.673681 3.871768 0.520213
O 0.500181 3.139658 -1.026367
H 1.350526 3.293207 -0.606942
C 0.099467 -2.034967 -1.317032
H 0.277945 -2.286268 -0.269245
H 0.208667 -2.97241 -1.87799
C 1.157613 -1.047465 -1.818002
H 1.154114 -0.155797 -1.17863
H 0.879273 -0.71215 -2.827181
C 1.584553 0.93663 2.573822

H 0.783769 0.593998 3.240403
H 2.394688 1.281121 3.225874
C 1.088132 2.130146 1.761711
H 0.858303 2.961646 2.43745
H 1.913436 2.475547 1.125249

conf_14

1 1
C -3.221958 1.863229 -1.116756
C -3.404353 1.090275 0.167792
H -3.920903 1.48928 -1.868153
H -2.209807 1.75649 -1.513513
H -3.433543 2.926126 -0.974985
C -2.028467 -0.890932 -0.746566
H -1.130338 -0.355106 -0.42473
H -2.19967 -0.61685 -1.793901
C 0.655075 -2.41422 -1.48209
C 1.33548 -2.887559 -0.200641
H 0.669453 -1.318074 -1.544186
H 1.249604 -2.756137 -2.337348
C 2.814576 -2.499469 -0.10642
H 1.267237 -3.981799 -0.161328
H 0.787348 -2.52778 0.677126
C 3.13235 -0.999448 -0.053446
H 3.320799 -2.93258 -0.976817
H 3.259536 -2.991757 0.766844
C 3.206751 -0.375931 1.342224
H 2.421256 -0.44703 -0.677739
H 4.105104 -0.833045 -0.527492
H 3.439481 0.691304 1.238172
H 4.056943 -0.815882 1.875955
C 0.669525 1.549911 1.6047
C 0.690537 2.377449 0.547917
H 0.66839 2.012616 2.593229
C 0.798561 2.016262 -0.900595
H 0.697708 3.445521 0.762909
C 2.150683 2.415621 -1.511196
H 0.010838 2.535984 -1.464865
H 0.644082 0.943551 -1.049217
C 2.411079 3.914579 -1.512515
H 2.178862 2.038063 -2.538783
H 2.948345 1.897139 -0.96911
H 1.612907 4.456525 -2.030803
H 3.348315 4.142979 -2.024953
H 2.49107 4.31938 -0.49979
N -2.455479 1.578351 1.232609
H -2.481483 0.878333 1.987583
H -2.741152 2.484991 1.599843
H -4.397313 1.272809 0.587971
H -1.462482 1.673913 0.935493
C -3.230347 -0.429973 0.064346
H -4.146666 -0.815521 -0.40287

O -3.177069 -0.845922 1.423943
H -3.108301 -1.80237 1.47729
C 0.692907 0.053775 1.609454
H -0.162062 -0.315807 2.19404
H 0.572251 -0.338824 0.598579
C 1.969529 -0.538103 2.21829
H 1.795497 -1.600715 2.416867
H 2.153442 -0.076169 3.195071
C -0.773872 -2.922746 -1.663788
H -1.119492 -2.671867 -2.673876
H -0.76834 -4.017186 -1.61016
C -1.798494 -2.397588 -0.657802
H -2.751049 -2.914213 -0.836127
H -1.478343 -2.668171 0.356518

conf_15

1 1
C -2.427905 2.082177 1.720275
C -2.161248 2.335528 0.254946
H -2.238466 2.997223 2.285598
H -1.788388 1.295428 2.125402
H -3.470915 1.803141 1.890143
C 0.40676 2.011683 0.394487
H 0.396289 1.038253 -0.111234
H 0.293738 1.818869 1.466697
C 3.201368 0.820999 -0.745564
C 4.445683 -0.049887 -0.559513
H 3.318354 1.397691 -1.672242
H 2.317359 0.190577 -0.8938
C 4.373095 -1.113099 0.542693
H 5.290227 0.619735 -0.358881
H 4.681207 -0.542591 -1.510644
C 3.84838 -2.478044 0.09195
H 3.786936 -0.740622 1.391577
H 5.379479 -1.270988 0.943033
C 2.437561 -2.497164 -0.490058
H 3.892433 -3.169406 0.942509
H 4.53738 -2.881961 -0.659052
H 2.209806 -3.512299 -0.835936
H 2.398993 -1.865187 -1.386565
C -1.035736 -1.442028 0.845188
C -2.327411 -1.79474 0.915428
H -0.652645 -0.792748 1.634983
C -3.060384 -2.704731 -0.020518
H -2.906007 -1.395381 1.748601
C -3.976693 -1.966054 -1.005131
H -3.670975 -3.395416 0.572903
H -2.354139 -3.316815 -0.586867
C -5.086502 -1.159346 -0.342588
H -4.428315 -2.701672 -1.677428
H -3.361367 -1.32714 -1.655307
H -5.749151 -1.810711 0.234175

H -5.705624 -0.644205 -1.08237
H -4.707226 -0.413477 0.368495
N -2.398418 1.093339 -0.56185
H -1.949406 1.248989 -1.474516
H -3.39273 0.912328 -0.702599
H -2.868816 3.06991 -0.139392
H -2.008802 0.237523 -0.112261
C -0.764562 2.845733 -0.103162
H -0.693513 3.861239 0.309813
O -0.804528 2.897282 -1.524889
H 0.041027 3.196591 -1.868979
C -0.009133 -1.89192 -0.149368
H -0.317538 -2.82308 -0.634703
H 0.081121 -1.154692 -0.964265
C 1.364253 -2.055955 0.494409
H 1.297616 -2.778493 1.316588
H 1.647599 -1.101465 0.955664
C 2.9591 1.804548 0.3951
H 2.841837 1.273246 1.348063
H 3.849762 2.431879 0.509801
C 1.749069 2.707626 0.16823
H 1.801032 3.578992 0.830421
H 1.812815 3.103158 -0.856178

conf_16

1 1
C -1.541096 -3.261153 0.041282
C -1.653961 -2.382131 -1.18155
H -2.535653 -3.613031 0.324214
H -1.116273 -2.72531 0.89275
H -0.927731 -4.143593 -0.158393
C -2.220566 -0.255146 0.119203
H -1.17294 0.053839 0.02072
H -2.298028 -0.827756 1.049265
C -1.097959 2.423652 0.990639
C -0.826543 2.976845 -0.406975
H -0.435292 1.573254 1.18966
H -0.819219 3.178335 1.733825
C 0.649102 2.994599 -0.792478
H -1.242765 3.987587 -0.477572
H -1.358213 2.384889 -1.163108
C 1.224295 1.60917 -1.075024
H 1.238089 3.477499 -0.002482
H 0.779266 3.613065 -1.687895
C 2.645742 1.661234 -1.617336
H 0.578488 1.107969 -1.814703
H 1.196227 0.996325 -0.164609
H 3.266669 2.256381 -0.935557
H 2.64061 2.206451 -2.568277
C 2.624593 -1.573538 -0.209644
C 1.918709 -1.766151 0.913218
H 2.599855 -2.364296 -0.964273

C 1.874315 -0.876586 2.115972
H 1.371267 -2.706238 1.011192
C 0.515262 -0.868613 2.809941
H 2.159922 0.146878 1.85057
H 2.632837 -1.221041 2.832623
C 0.474093 0.057063 4.015121
H 0.26239 -1.891336 3.118008
H -0.253909 -0.562209 2.091192
H 1.22 -0.233339 4.760657
H -0.504606 0.037783 4.500939
H 0.681819 1.091642 3.724532
N -0.300182 -1.866072 -1.609139
H -0.473482 -1.059086 -2.226304
H 0.22327 -2.569776 -2.127958
H -2.014862 -2.957009 -2.038495
H 0.307055 -1.566932 -0.823629
C -2.56368 -1.159027 -1.0521
H -3.59222 -1.532875 -0.963596
O -2.385127 -0.503488 -2.302163
H -2.904422 0.304083 -2.329824
C 3.577862 -0.462203 -0.511962
H 3.628087 0.241251 0.323998
H 4.571616 -0.923626 -0.566956
C 3.314184 0.305585 -1.821362
H 2.712649 -0.31043 -2.505619
H 4.264911 0.467913 -2.336512
C -2.548296 2.005947 1.238902
H -2.630553 1.592524 2.251047
H -3.194895 2.889823 1.219564
C -3.095384 0.988702 0.234063
H -4.110964 0.697912 0.523562
H -3.190738 1.483388 -0.740739

1-Deoxy-PS

conf_00

1 1
C -0.156617 1.374761 1.659243
C -0.070766 2.677635 0.897472
H 0.547828 1.38726 2.493061
H 0.068044 0.515737 1.025638
H -1.156423 1.242928 2.080166
C 2.112193 2.112207 -0.418585
H 2.836753 2.70121 -1.002063
C 4.428881 -0.933686 0.351875
C 3.447617 -2.028748 0.769832
H 4.913783 -0.511926 1.240388
H 5.229963 -1.382627 -0.245527
C 2.9716 -2.899651 -0.38812
H 2.586718 -1.581888 1.283022
H 3.928021 -2.669816 1.517424
C 1.883406 -3.903631 -0.012941

H 3.832233 -3.445266 -0.792193
H 2.610832 -2.272543 -1.212766
C 0.57191 -3.288939 0.476607
H 2.267299 -4.581136 0.758758
H 1.672477 -4.530544 -0.887422
H 0.721468 -2.798738 1.44748
H -0.142812 -4.098668 0.663983
C -1.967589 -0.727405 -1.017603
C -3.167977 0.049202 -0.484309
H -2.241738 -1.203409 -1.966857
H -1.152509 -0.034204 -1.272617
C -4.416362 -0.793793 -0.254446
H -3.425214 0.856218 -1.187548
H -2.902547 0.523374 0.476538
C -5.60929 0.020573 0.227673
H -4.198576 -1.582247 0.475442
H -4.675497 -1.305799 -1.190054
C -6.851446 -0.827099 0.457128
H -5.830706 0.806465 -0.506051
H -5.340949 0.537473 1.15882
H -7.160735 -1.330311 -0.464018
H -7.692011 -0.2195 0.800999
H -6.669225 -1.599265 1.210924
N -0.951534 2.621462 -0.323409
H -0.980605 3.54865 -0.754452
H -1.893217 2.285387 -0.113924
H -0.463506 3.496353 1.505745
H -0.488241 1.984031 -0.99619
C 1.312506 3.125268 0.411105
H 1.902719 3.372227 1.302448
O 1.012012 4.295609 -0.332306
H 1.81633 4.73574 -0.617504
C -1.411288 -1.777621 -0.061728
H -1.335214 -1.352856 0.949543
H -2.110223 -2.616479 0.023656
C -0.046292 -2.294022 -0.496472
H 0.62808 -1.434839 -0.62296
H -0.130686 -2.756046 -1.489371
C 3.796983 0.20653 -0.448022
H 4.58393 0.826535 -0.892462
H 3.240732 -0.229364 -1.289762
C 2.884242 1.088116 0.399875
H 2.18733 0.464034 0.962584
H 3.494775 1.624399 1.136414
O 1.194514 1.495254 -1.325772
H 1.659589 0.874137 -1.892317

conf_01

1 1
C 3.473933 1.861915 -0.018526
C 2.692615 1.244534 -1.155002
H 4.530866 1.612277 -0.135318

H 3.147402 1.495673 0.957672
H 3.393915 2.951384 -0.024683
C 2.755597 -1.161639 -0.099186
H 3.630954 -0.964179 0.536492
C 0.058166 -1.83917 2.661098
C -1.249065 -1.641265 1.898486
H 0.244957 -0.954315 3.282656
H -0.045058 -2.679357 3.356408
C -1.710662 -2.833416 1.061073
H -1.163557 -0.756761 1.253529
H -2.039488 -1.391252 2.614839
C -2.766645 -2.429884 0.032158
H -2.099364 -3.608304 1.730155
H -0.86377 -3.289882 0.532305
C -2.147194 -1.810148 -1.222897
H -3.463841 -1.727044 0.503952
H -3.368002 -3.296723 -0.259245
H -1.802668 -2.617031 -1.879584
H -1.237061 -1.261513 -0.944926
C -2.255122 1.285944 -0.909428
C -2.643521 2.547158 -0.147625
H -1.716539 1.565607 -1.829814
H -1.562421 0.693949 -0.297698
C -1.456762 3.34532 0.383563
H -3.289884 2.271634 0.695106
H -3.251393 3.18554 -0.797423
C -0.713412 2.677554 1.536911
H -1.798543 4.328933 0.723753
H -0.762225 3.565573 -0.445386
C 0.511634 3.45338 1.997731
H -0.439558 1.640732 1.280844
H -1.406396 2.560497 2.377592
H 1.235805 3.602894 1.186767
H 1.028608 2.950294 2.819263
H 0.230372 4.449978 2.348073
N 1.218065 1.537811 -1.048981
H 0.741917 0.882784 -1.69
H 0.987026 2.497474 -1.304503
H 2.985871 1.698895 -2.105271
H 0.847979 1.376508 -0.108104
C 2.84558 -0.264555 -1.339048
H 3.840577 -0.426915 -1.772905
O 1.845336 -0.611828 -2.275921
H 1.837626 -1.574646 -2.352094
C -3.444268 0.408056 -1.280446
H -4.001789 0.173106 -0.366079
H -4.132795 0.985931 -1.907122
C -3.067709 -0.880651 -2.010472
H -2.587208 -0.623557 -2.963141
H -3.984916 -1.419478 -2.273997
C 1.28035 -2.08725 1.775603
H 2.173385 -2.151515 2.409744
H 1.172305 -3.060196 1.282942
C 1.477999 -1.006482 0.717873

H 0.620617 -1.015585 0.036019
H 1.497917 -0.035315 1.235469
O 2.858188 -2.449891 -0.681224
H 2.864654 -3.123425 0.002544

conf_02

1 1
C -3.98508 2.118602 0.961728
C -2.952482 1.02022 1.092627
H -4.983142 1.690251 1.037491
H -3.905073 2.621654 -0.007557
H -3.870334 2.863853 1.754151
C -2.105732 -1.242608 0.214051
H -2.377904 -1.704756 1.174136
C -0.192037 -3.72915 -0.077538
C 0.821393 -3.317317 -1.145347
H 0.027273 -4.756129 0.233733
H -0.061832 -3.146244 0.847116
C 2.233989 -3.085141 -0.614798
H 0.493891 -2.400133 -1.654488
H 0.835959 -4.091017 -1.920496
C 2.343032 -1.850258 0.273871
H 2.913588 -2.964049 -1.465865
H 2.583793 -3.969735 -0.068891
C 3.774306 -1.434634 0.585817
H 1.814117 -2.012917 1.225021
H 1.843626 -1.012976 -0.236101
H 4.287895 -1.206651 -0.355839
H 4.311548 -2.284953 1.020831
C 3.443289 1.50027 -0.284123
C 2.799426 2.831813 -0.656477
H 3.156706 0.748986 -1.031547
H 4.532204 1.600858 -0.364346
C 1.274768 2.837777 -0.594855
H 3.109146 3.100029 -1.673341
H 3.184687 3.625139 -0.003637
C 0.656792 4.059635 -1.263174
H 0.961977 2.8003 0.459953
H 0.888256 1.92492 -1.074616
C -0.858646 4.123887 -1.142512
H 0.934703 4.061634 -2.322774
H 1.092518 4.968976 -0.834276
H -1.328902 3.216686 -1.549999
H -1.279793 4.964216 -1.699428
H -1.171333 4.266196 -0.098457
N -1.576634 1.624562 1.071578
H -0.89073 0.845522 0.926547
H -1.379524 2.12802 1.935142
H -3.045883 0.518789 2.061308
H -1.470249 2.289758 0.298749
C -3.051913 -0.050606 -0.007821
H -2.809667 0.416739 -0.977645

O -4.390505 -0.464874 0.041888
H -4.621618 -0.933048 -0.762856
C 3.094887 0.99683 1.111674
H 3.280378 1.796413 1.840242
H 2.018316 0.783447 1.168689
C 3.878291 -0.244693 1.538899
H 3.538081 -0.551337 2.53582
H 4.935186 0.023337 1.650366
C -1.649974 -3.663318 -0.529892
H -1.781262 -4.316366 -1.399655
H -2.286511 -4.079734 0.259202
C -2.186014 -2.276277 -0.899292
H -1.674337 -1.868857 -1.77896
H -3.23552 -2.408478 -1.184259
O -0.790738 -0.676312 0.31113
H -0.113629 -1.361003 0.334312

conf_03

1 1
C -2.043831 2.394743 2.017594
C -0.963556 1.492663 1.46119
H -2.865243 2.470946 1.306987
H -1.671404 3.407745 2.190537
H -2.439308 1.995734 2.956742
C -1.160291 2.189691 -1.027544
H -0.483296 2.337116 -1.880889
C -2.302085 -1.412011 -2.057426
C -3.338222 -1.808488 -1.004387
H -1.692697 -2.279467 -2.328499
H -2.835971 -1.131615 -2.973302
C -2.834425 -2.140565 0.402056
H -3.89489 -2.672894 -1.384221
H -4.0808 -1.005949 -0.916064
C -1.972393 -3.397802 0.553272
H -3.720651 -2.266292 1.033379
H -2.309089 -1.273655 0.826532
C -0.491184 -3.273543 0.196474
H -2.416904 -4.197742 -0.050345
H -2.041048 -3.741075 1.592887
H -0.370737 -2.961464 -0.846304
H -0.033644 -4.267451 0.261066
C 2.454992 -0.999639 1.342715
C 3.923871 -0.818339 0.968081
H 2.372087 -1.117356 2.433229
H 1.9297 -0.069984 1.062596
C 4.171113 -0.603572 -0.524146
H 4.48662 -1.695369 1.306828
H 4.328007 0.035923 1.525068
C 3.382332 0.549123 -1.135475
H 3.952154 -1.524147 -1.078737
H 5.241097 -0.418968 -0.672005
C 3.77751 0.835018 -2.576734

H 3.521998 1.453335 -0.527412
H 2.307966 0.318203 -1.103554
H 4.83394 1.109961 -2.646185
H 3.193566 1.656648 -3.000556
H 3.624962 -0.045216 -3.209082
N 0.157578 1.424462 2.473822
H 0.810701 0.656636 2.269552
H -0.188561 1.32265 3.427633
H -1.329743 0.471503 1.335601
H 0.673502 2.31277 2.381277
C -0.255656 1.967015 0.180961
H 0.477788 1.201532 -0.096433
O 0.477554 3.132715 0.492836
H -0.073772 3.885125 0.235022
C 1.748066 -2.156125 0.650885
H 1.777442 -1.9993 -0.434012
H 2.300188 -3.084879 0.836352
C 0.298628 -2.322116 1.086946
H -0.184985 -1.333274 1.073091
H 0.254158 -2.664617 2.129513
C -1.370637 -0.268775 -1.661054
H -0.758132 -0.586444 -0.809236
H -0.666051 -0.084498 -2.481394
C -2.098644 1.030592 -1.328355
H -2.724399 1.319362 -2.181566
H -2.788287 0.890182 -0.488493
O -1.846155 3.405312 -0.754928
H -2.224037 3.753627 -1.565935

conf_04

1 1
C -0.148422 1.336616 1.909952
C -0.556463 2.526872 1.070344
H 0.706728 1.599654 2.535391
H 0.121169 0.476292 1.295124
H -0.961957 1.04707 2.580642
C 1.229026 2.189875 -0.794711
H 1.535411 2.781027 -1.67086
C 4.37059 -0.139629 -0.576272
C 4.270688 -0.764773 0.818503
H 5.167791 0.612348 -0.57107
H 4.685112 -0.899344 -1.299139
C 3.034742 -1.627481 1.060628
H 4.292101 0.026926 1.577515
H 5.174905 -1.358485 0.992053
C 2.828837 -2.793231 0.09681
H 2.144575 -0.986213 1.037466
H 3.07622 -2.021918 2.082547
C 1.42512 -3.387467 0.205867
H 3.57908 -3.565066 0.299386
H 3.001075 -2.475082 -0.939409
H 1.197301 -3.575027 1.263674

H 1.391756 -4.364185 -0.288572
C -2.133645 -1.945206 -0.562851
C -3.542055 -2.291918 -0.098726
H -2.099679 -1.929648 -1.6608
H -1.874088 -0.925434 -0.238366
C -4.644419 -1.419567 -0.693849
H -3.57953 -2.251165 0.9975
H -3.751754 -3.335738 -0.357817
C -4.502887 0.08039 -0.438937
H -5.610128 -1.751732 -0.295474
H -4.691019 -1.58486 -1.776508
C -4.4098 0.444695 1.038618
H -5.363844 0.593266 -0.879471
H -3.627801 0.45697 -0.988005
H -5.253511 0.031617 1.598834
H -4.451352 1.530719 1.20048
H -3.505072 0.040943 1.511594
N -1.71176 2.155826 0.182016
H -1.995181 2.986153 -0.34624
H -2.507612 1.773116 0.697539
H -0.925114 3.331914 1.711323
H -1.352214 1.467503 -0.502059
C 0.503886 3.153433 0.154519
H 1.254108 3.625422 0.801118
O -0.239596 4.138069 -0.54806
H 0.337875 4.679106 -1.091739
C -1.07477 -2.900509 -0.02849
H -1.161512 -2.958259 1.064815
H -1.27603 -3.912843 -0.399362
C 0.344645 -2.489599 -0.390882
H 0.49691 -1.455142 -0.052817
H 0.45527 -2.470373 -1.484363
C 3.080717 0.490721 -1.09894
H 3.273933 0.920717 -2.090434
H 2.350992 -0.317355 -1.241765
C 2.479079 1.558233 -0.192667
H 2.259723 1.13222 0.789396
H 3.209047 2.3605 -0.029539
O 0.269792 1.220341 -1.224036
H 0.687851 0.577617 -1.803432

conf_05

1 1
C -0.095941 -1.155992 -1.554908
C 0.011341 -1.323883 -0.056286
H -0.487973 -0.164617 -1.786791
H -0.748908 -1.904722 -2.005286
H 0.893502 -1.222076 -2.015096
C -2.471447 -1.993544 0.324077
H -3.065481 -2.209701 1.22375
C -4.19358 1.184455 -0.792638
C -2.863753 1.935935 -0.842926

H -4.95725 1.845826 -0.36859
H -4.525374 0.954727 -1.811842
C -2.4678 2.590544 0.477738
H -2.925242 2.716406 -1.609514
H -2.068906 1.25964 -1.185187
C -1.119066 3.304447 0.448183
H -2.458881 1.851793 1.292188
H -3.248683 3.310205 0.749195
C 0.093867 2.381783 0.381488
H -1.028394 3.928639 1.344457
H -1.093612 3.996117 -0.403621
H 0.038489 1.758839 -0.520432
H 0.073561 1.698348 1.244899
C 2.853238 1.382443 -0.796215
C 4.25648 0.792939 -0.92365
H 2.64137 1.968058 -1.699605
H 2.109818 0.574445 -0.793511
C 4.731928 -0.037174 0.267388
H 4.965766 1.616265 -1.06615
H 4.314947 0.195104 -1.841149
C 3.888352 -1.268032 0.59421
H 4.78601 0.596508 1.158952
H 5.759584 -0.366282 0.073483
C 3.801206 -2.267079 -0.554984
H 2.884247 -0.946919 0.907711
H 4.324737 -1.7642 1.467376
H 3.219396 -1.884303 -1.401461
H 3.384773 -3.233798 -0.237343
H 4.796164 -2.499793 -0.945641
N 0.573137 -2.681041 0.268905
H 0.623495 -2.763702 1.289666
H 1.500862 -2.816622 -0.139351
H 0.730743 -0.608808 0.349664
H -0.104832 -3.382335 -0.064649
C -1.260509 -1.171419 0.781554
H -1.530603 -0.11112 0.751684
O -0.821759 -1.533149 2.084722
H -1.49822 -1.339017 2.737532
C 2.663378 2.274147 0.427793
H 2.650553 1.665255 1.341892
H 3.539311 2.927878 0.519036
C 1.412517 3.146281 0.386744
H 1.453488 3.793111 -0.499216
H 1.421975 3.819315 1.252093
C -4.151841 -0.110743 0.017095
H -3.736307 0.103862 1.010299
H -5.171701 -0.457329 0.206324
C -3.354597 -1.237576 -0.660603
H -4.043085 -1.951941 -1.124614
H -2.743507 -0.83986 -1.475303
O -1.975794 -3.229347 -0.202059
H -2.709993 -3.811145 -0.413845

conf_06

1 1
C -0.098793 -1.965193 1.512555
C -0.590799 -2.920051 0.448297
H -0.791862 -1.963261 2.35598
H -0.003899 -0.944992 1.138314
H 0.875004 -2.286081 1.892297
C -2.34151 -1.323607 -0.646051
H -3.166658 -1.456854 -1.36237
C -3.320875 2.119513 0.921502
C -2.169262 2.288402 1.91843
H -4.238345 1.88734 1.474077
H -3.512941 3.069721 0.413245
C -0.777697 2.365341 1.294763
H -2.184419 1.463761 2.641653
H -2.354445 3.192593 2.508708
C -0.556278 3.529628 0.333432
H -0.559582 1.422744 0.773621
H -0.034127 2.430952 2.098011
C 0.757281 3.406861 -0.436538
H -0.572607 4.465718 0.902138
H -1.384769 3.60041 -0.383338
H 1.554941 3.141238 0.26839
H 1.038136 4.377766 -0.858778
C 2.967367 1.224585 -1.273813
C 2.44523 -0.171529 -0.947008
H 3.143403 1.778319 -0.345917
H 3.947058 1.12699 -1.755767
C 3.370129 -0.986681 -0.047528
H 2.284872 -0.704916 -1.896172
H 1.462971 -0.082412 -0.462721
C 3.526556 -0.430765 1.36327
H 4.356726 -1.076624 -0.518516
H 3.005886 -2.025934 0.034575
C 4.302324 -1.362207 2.281924
H 2.532897 -0.229169 1.786244
H 4.032573 0.538822 1.318113
H 3.799447 -2.330372 2.388814
H 4.415129 -0.937085 3.282007
H 5.305248 -1.557334 1.890702
N 0.333631 -2.886137 -0.740047
H 0.035459 -3.602123 -1.407998
H 1.310366 -3.018209 -0.476975
H -0.551333 -3.947182 0.820214
H 0.204611 -1.964832 -1.196935
C -2.008872 -2.719033 -0.102619
H -2.70679 -2.941487 0.714335
O -2.085444 -3.713189 -1.112612
H -2.969394 -3.75465 -1.485261
C 2.041579 2.027804 -2.186021
H 2.549525 2.952138 -2.48387
H 1.879324 1.462573 -3.112659
C 0.690027 2.384622 -1.569314

H 0.210045 1.468073 -1.201007
H 0.031543 2.778782 -2.353126
C -3.082153 1.054544 -0.147524
H -3.95035 1.004319 -0.814881
H -2.239021 1.39554 -0.763491
C -2.789058 -0.330986 0.418476
H -2.030323 -0.254588 1.199419
H -3.691948 -0.728955 0.897044
O -1.189989 -0.87266 -1.36303
H -1.355486 -0.008469 -1.750035

ω-OH-1-DeoxySA

conf_00

1 1
C 0.913274 -3.68179 -0.047923
C 0.558953 -2.261169 -0.438898
H 0.256752 -4.383286 -0.563712
H 0.797226 -3.834354 1.027813
H 1.940034 -3.923544 -0.333647
C -1.960708 -2.696128 -0.371437
H -1.848035 -3.648017 0.156802
H -1.887043 -2.90807 -1.445872
C -4.829336 -0.046856 -0.509936
C -4.48756 0.714588 0.777019
H -5.739276 -0.633671 -0.339822
H -5.083943 0.664074 -1.302634
C -3.102268 1.358209 0.794113
H -4.571427 0.042524 1.63941
H -5.249717 1.485196 0.937918
C -2.855367 2.375102 -0.319338
H -2.337091 0.571311 0.720387
H -2.941088 1.827881 1.770292
C -1.378175 2.594009 -0.630007
H -3.326197 3.329296 -0.053939
H -3.349817 2.04416 -1.238707
H -1.285417 3.28822 -1.474111
H -0.958749 1.639585 -0.982332
C 1.731283 2.132501 -0.071448
C 3.157607 2.420691 -0.526314
H 1.235772 1.530766 -0.851503
H 1.736584 1.541202 0.857251
C 4.007249 1.174011 -0.758909
H 3.650249 3.068809 0.209162
H 3.110903 2.999945 -1.454574
C 4.474735 0.495355 0.526029
H 4.893478 1.441793 -1.344545
H 3.451577 0.462696 -1.385817
C 5.172681 -0.826946 0.301856
H 3.64454 0.34545 1.229461
H 5.1756 1.15583 1.048212
H 5.601644 -1.202497 1.23628

H 5.976513 -0.713881 -0.433639
N 1.550504 -1.327194 0.190162
H 1.429104 -0.365856 -0.141166
H 2.550803 -1.597016 0.032432
H 0.636431 -2.124625 -1.520532
H 1.344229 -1.314327 1.194804
C -0.814274 -1.785527 0.035516
H -0.980052 -0.783648 -0.390863
O -0.678144 -1.670738 1.446731
H -1.498319 -1.345716 1.826805
C 0.908092 3.391669 0.180688
H 1.384484 3.957979 0.988878
H 0.955224 4.032421 -0.708927
C -0.552708 3.127232 0.535495
H -1.007349 4.061016 0.886224
H -0.600615 2.432522 1.38461
C -3.71913 -0.958908 -1.026246
H -4.030103 -1.403932 -1.978058
H -2.842915 -0.343089 -1.266136
C -3.326406 -2.079825 -0.066777
H -3.343342 -1.71529 0.969345
H -4.087054 -2.865744 -0.095131
O 4.214239 -1.781265 -0.195121
H 4.666461 -2.58705 -0.455324

conf_01

1 1
C -1.726771 -3.197233 -2.280007
C -1.202967 -1.820131 -1.930789
H -2.813466 -3.209692 -2.192906
H -1.323766 -3.955364 -1.603779
H -1.483489 -3.467046 -3.312073
C -2.780308 -1.539825 0.045433
H -3.079781 -2.591707 0.011532
H -3.439215 -0.996371 -0.642788
C -3.133432 1.45842 0.810127
C -2.544562 2.857143 0.981112
H -4.20645 1.485688 1.032487
H -3.060213 1.163174 -0.244021
C -1.113724 3.010533 0.467968
H -2.576585 3.126964 2.043351
H -3.184006 3.583542 0.466226
C -0.979515 2.963948 -1.052165
H -0.469291 2.24743 0.924409
H -0.714505 3.971172 0.812973
C 0.475465 2.967331 -1.514939
H -1.506045 3.825884 -1.476137
H -1.486623 2.077395 -1.459181
H 1.041054 3.68437 -0.906743
H 0.549444 3.325717 -2.547121
C 3.378465 0.317572 -1.416865
C 3.079749 -0.511616 -0.166464

H 4.458743 0.491525 -1.47101
H 3.140642 -0.275536 -2.313573
C 3.272106 0.243404 1.144041
H 3.72144 -1.401939 -0.177659
H 2.046287 -0.882242 -0.188892
C 3.242195 -0.645885 2.386097
H 2.497104 1.011916 1.235013
H 4.228424 0.778151 1.122393
C 2.072805 -1.610349 2.445326
H 3.249544 -0.016026 3.283244
H 4.155274 -1.252276 2.435483
H 2.06393 -2.153723 3.39557
H 2.14813 -2.356813 1.650635
N 0.288055 -1.805888 -2.155229
H 0.663641 -0.851318 -2.170522
H 0.571474 -2.294908 -3.002947
H -1.636687 -1.053791 -2.577346
H 0.668097 -2.271423 -1.309683
C -1.353926 -1.419219 -0.457326
H -1.031105 -0.370981 -0.374533
O -0.443741 -2.24692 0.245753
H -0.137672 -1.791115 1.058498
C 2.66809 1.666331 -1.49383
H 3.02391 2.310788 -0.68197
H 2.961123 2.171077 -2.421273
C 1.149912 1.600978 -1.41643
H 0.848188 1.108187 -0.482517
H 0.763463 0.986007 -2.251711
C -2.473274 0.411274 1.700491
H -1.384098 0.452325 1.573906
H -2.654291 0.67623 2.748845
C -2.96047 -1.017396 1.471333
H -2.444179 -1.692605 2.162903
H -4.023841 -1.086801 1.723791
O 0.804515 -0.974111 2.234949
H 0.533806 -0.50809 3.029436

conf_02

1 1
C 4.995327 0.523318 -1.432022
C 4.151737 -0.221847 -0.416955
H 5.77959 1.084712 -0.921218
H 4.402986 1.232043 -2.017873
H 5.487016 -0.167697 -2.122128
C 2.30237 1.490138 0.063157
H 1.490028 0.79923 -0.194909
H 2.607283 2.006001 -0.85689
C -0.579808 2.5304 0.06099
C -1.80742 3.359348 -0.300919
H -0.827797 1.828304 0.867356
H -0.318263 1.912661 -0.809145
C -2.927568 2.521944 -0.912164

H -1.512307 4.140196 -1.012311
H -2.172782 3.882269 0.592123
C -3.537283 1.501675 0.04336
H -2.528015 1.995553 -1.791251
H -3.715166 3.181056 -1.293636
C -4.3925 0.457183 -0.661511
H -4.136624 2.019176 0.803415
H -2.737612 0.994496 0.592369
H -3.819789 0.020874 -1.490997
H -5.2526 0.948369 -1.131727
C -2.836915 -2.230079 0.083367
C -1.589357 -1.433701 -0.293486
H -3.340374 -2.570104 -0.831839
H -2.526244 -3.142269 0.605983
C -0.582378 -2.211121 -1.138787
H -1.105899 -1.069761 0.623861
H -1.872188 -0.534589 -0.847118
C 0.00118 -3.463755 -0.484593
H 0.233472 -1.530239 -1.419641
H -1.055097 -2.50574 -2.082564
C 0.666206 -3.23067 0.852546
H 0.72095 -3.938318 -1.162859
H -0.786958 -4.207405 -0.329208
H 1.016138 -4.175637 1.278231
H -0.031251 -2.771416 1.559595
N 3.126469 -1.057785 -1.132205
H 2.515002 -1.62463 -0.44963
H 3.585239 -1.713795 -1.763619
H 4.765806 -0.934586 0.139827
H 2.514533 -0.46879 -1.697658
C 3.476326 0.688935 0.608431
H 4.2615 1.377923 0.947206
O 3.087322 -0.142446 1.698692
H 2.871553 0.414664 2.451051
C -3.837771 -1.485358 0.97531
H -4.368663 -2.22093 1.588492
H -3.287825 -0.857334 1.688091
C -4.900684 -0.656291 0.25002
H -5.566985 -0.222046 1.005495
H -5.524154 -1.33642 -0.342972
C 0.622267 3.3618 0.489147
H 0.981359 3.959339 -0.358617
H 0.311369 4.080937 1.254971
C 1.771115 2.52682 1.049374
H 2.592066 3.187451 1.353832
H 1.41685 2.020623 1.957731
O 1.796685 -2.358702 0.683132
H 2.007462 -1.852915 1.477432

conf_03

1 1
C -1.246246 -2.200411 1.920599

C -1.104985 -1.618898 0.534551
H -0.658146 -1.609458 2.626348
H -0.889937 -3.233124 1.974464
H -2.287475 -2.169895 2.250415
C 1.220814 -2.676119 0.113305
H 0.815544 -3.480981 -0.514383
H 1.2174 -3.037889 1.147664
C 4.68362 -0.863605 0.216445
C 4.479407 0.088312 -0.968713
H 5.409352 -1.632924 -0.07098
H 5.143818 -0.322671 1.049753
C 3.276434 1.020495 -0.846455
H 4.37581 -0.491421 -1.893834
H 5.390837 0.681758 -1.101028
C 3.29093 1.962896 0.358053
H 2.359798 0.415092 -0.787015
H 3.181777 1.598944 -1.771131
C 1.892338 2.392923 0.793744
H 3.901563 2.843814 0.12753
H 3.780604 1.473677 1.207068
H 1.963211 3.025778 1.686424
H 1.342643 1.49436 1.109533
C -1.24154 2.205932 0.215552
C -2.698543 2.55302 0.499893
H -0.8756 1.54219 1.011433
H -1.156765 1.642748 -0.723637
C -3.650145 1.359326 0.532619
H -3.052234 3.2698 -0.252605
H -2.754826 3.076453 1.460919
C -3.870175 0.712428 -0.83176
H -4.623286 1.691199 0.91379
H -3.290686 0.613036 1.253484
C -4.900607 -0.393208 -0.834717
H -2.928338 0.337179 -1.253753
H -4.218211 1.475391 -1.538371
H -5.132112 -0.711018 -1.856821
H -5.825327 -0.049084 -0.360628
N -1.881471 -2.427455 -0.465311
H -1.539181 -2.153697 -1.395398
H -2.906076 -2.201425 -0.382256
H -1.569047 -0.631274 0.493455
H -1.729086 -3.428132 -0.345886
C 0.317905 -1.454139 0.007159
H 0.752426 -0.622385 0.574654
O 0.114396 -1.057087 -1.345171
H 0.949572 -0.798115 -1.743828
C -0.346322 3.4382 0.121454
H -0.779334 4.123531 -0.616733
H -0.361114 3.975348 1.078661
C 1.095038 3.130483 -0.275796
H 1.61277 4.066841 -0.514975
H 1.083419 2.546562 -1.205115
C 3.407519 -1.523914 0.733149
H 3.65119 -2.151472 1.597989

H 2.745985 -0.739192 1.120237
C 2.66475 -2.375466 -0.294916
H 2.680059 -1.890047 -1.279243
H 3.199198 -3.319171 -0.440629
O -4.400896 -1.529263 -0.093301
H -5.139054 -2.086879 0.16438

conf_04

1 1
C 2.785193 -3.366725 -0.630665
C 1.926384 -2.364702 -1.366564
H 2.269503 -4.328776 -0.592153
H 2.989915 -3.052712 0.395362
H 3.73791 -3.525549 -1.141834
C 0.500632 -1.840837 0.709378
H 1.25893 -1.087986 0.957286
H 0.778419 -2.758189 1.241031
C -3.353724 -1.867708 1.520188
C -4.262088 -1.385849 0.389011
H -3.216862 -1.07316 2.263786
H -3.87714 -2.672878 2.044074
C -3.769566 -0.187894 -0.417688
H -5.243668 -1.137216 0.80936
H -4.433472 -2.226406 -0.294943
C -3.691246 1.117062 0.367296
H -4.446581 -0.042745 -1.268306
H -2.795134 -0.422362 -0.873222
C -3.469976 2.35058 -0.507575
H -2.916573 1.054722 1.141542
H -4.635341 1.244827 0.910299
H -3.569373 3.250667 0.110925
H -4.280282 2.400741 -1.244483
C 0.394751 2.580656 -1.073125
C 1.615108 2.89746 -0.213451
H 0.354858 3.284882 -1.912218
H 0.512132 1.583682 -1.52373
C 1.740533 2.010271 1.02246
H 1.579217 3.946423 0.104498
H 2.514521 2.810868 -0.837227
C 3.118114 2.043188 1.677569
H 1.491567 0.974217 0.758087
H 0.98959 2.307071 1.760204
C 4.184386 1.298847 0.900361
H 3.066633 1.625223 2.691061
H 3.452564 3.080536 1.799343
H 5.169481 1.412915 1.361608
H 4.261131 1.66428 -0.126711
N 2.603515 -1.021305 -1.434171
H 1.880271 -0.342728 -1.70948
H 3.346263 -1.018148 -2.130914
H 1.792115 -2.667634 -2.408509
H 3.028413 -0.719489 -0.519959

C 0.529605 -2.132317 -0.787366
H -0.048556 -3.040646 -0.997708
O 0.03407 -1.052664 -1.566044
H -0.853794 -0.814621 -1.282078
C -0.931866 2.614657 -0.325504
H -0.928617 1.853173 0.46373
H -1.037001 3.578647 0.190099
C -2.131633 2.405924 -1.240733
H -1.989571 1.492209 -1.834561
H -2.161448 3.222637 -1.971288
C -1.983277 -2.379685 1.056306
H -1.707756 -3.276507 1.623279
H -2.067466 -2.707587 0.010648
C -0.858583 -1.360081 1.211988
H -1.124436 -0.41483 0.72295
H -0.759903 -1.105985 2.272788
O 3.890153 -0.107632 0.776217
H 3.944663 -0.517155 1.643856

conf_05

1 1
C 3.15712 -3.119095 -0.69271
C 2.311703 -2.09017 -1.406723
H 2.670533 -4.095062 -0.752809
H 3.288043 -2.87563 0.364135
H 4.142314 -3.216231 -1.155977
C 0.733279 -1.772985 0.602694
H 1.382422 -0.959141 0.94561
H 1.088282 -2.690293 1.086697
C -3.101111 -2.389541 1.265207
C -4.049524 -1.853669 0.193466
H -3.080505 -1.712692 2.128091
H -3.52239 -3.328142 1.637721
C -3.718163 -0.483809 -0.391518
H -5.060465 -1.809524 0.615736
H -4.097492 -2.586145 -0.62214
C -3.77198 0.661054 0.615444
H -4.432039 -0.275005 -1.197935
H -2.735576 -0.515427 -0.886108
C -3.829288 2.044903 -0.029627
H -2.926215 0.600882 1.311514
H -4.667973 0.534211 1.235193
H -3.929614 2.803355 0.756212
H -4.745653 2.107811 -0.628501
C -0.164907 2.891893 -1.108523
C 1.149076 3.224461 -0.410677
H -0.448013 3.737356 -1.74607
H -0.007546 2.044183 -1.791095
C 1.663688 2.120761 0.507681
H 1.036069 4.148931 0.168948
H 1.908731 3.441172 -1.172943
C 3.116041 2.332441 0.915821

H 1.537253 1.15378 0.005029
H 1.040895 2.059008 1.407948
C 3.673526 1.292777 1.857092
H 3.210158 3.303449 1.415746
H 3.759951 2.393914 0.028407
H 3.030223 1.183303 2.737669
H 4.676825 1.577359 2.187992
N 2.946012 -0.727879 -1.331094
H 2.240694 -0.057726 -1.663856
H 3.772673 -0.667334 -1.922903
H 2.256028 -2.31705 -2.475026
H 3.225739 -0.455619 -0.355965
C 0.873319 -1.947301 -0.904566
H 0.347095 -2.856488 -1.221588
O 0.38464 -0.827802 -1.631733
H -0.518047 -0.628401 -1.366779
C -1.322899 2.569123 -0.172776
H -1.098007 1.659188 0.398618
H -1.427167 3.369867 0.57193
C -2.641745 2.40518 -0.917663
H -2.525801 1.652995 -1.709763
H -2.864497 3.343564 -1.438834
C -1.669119 -2.648277 0.781341
H -1.271878 -3.546286 1.269715
H -1.699495 -2.88321 -0.291716
C -0.699881 -1.502138 1.054853
H -1.066661 -0.569055 0.60992
H -0.683441 -1.306584 2.13249
O 3.756555 0.019098 1.180252
H 4.188095 -0.612145 1.761294

conf_06

1 1
C -0.00785 1.175184 2.202357
C 0.749073 1.31635 0.897103
H -1.077591 1.091067 2.006722
H 0.156858 2.037865 2.852801
H 0.2906 0.266328 2.732587
C -0.942244 3.018428 0.044952
H -1.003919 4.019423 -0.399085
H -1.361324 3.109416 1.053031
C -4.163931 1.276806 -1.323183
C -3.753477 -0.187296 -1.461545
H -4.211182 1.723332 -2.323607
H -5.18181 1.331671 -0.920609
C -3.541635 -0.935766 -0.150773
H -2.833035 -0.259771 -2.057203
H -4.518811 -0.707206 -2.049415
C -3.275143 -2.419401 -0.382776
H -4.416069 -0.8135 0.501367
H -2.699099 -0.49022 0.394919
C -2.716439 -3.174548 0.820401

H -2.585977 -2.528762 -1.23005
H -4.205717 -2.900703 -0.703441
H -2.634629 -4.235093 0.55431
H -3.429051 -3.126301 1.652159
C 1.113985 -2.369417 0.708432
C 2.092909 -2.028906 -0.407075
H 1.452359 -3.267096 1.239505
H 1.117451 -1.568843 1.465291
C 3.53026 -1.850929 0.062436
H 1.742167 -1.120496 -0.91811
H 2.058216 -2.816955 -1.169692
C 4.514644 -1.440557 -1.030309
H 3.881304 -2.786541 0.510146
H 3.576362 -1.117953 0.879587
C 4.132757 -0.201999 -1.8114
H 4.612397 -2.254243 -1.758936
H 5.506752 -1.295137 -0.59126
H 3.21529 -0.358021 -2.387832
H 4.930913 0.052119 -2.513439
N 2.221596 1.241103 1.178112
H 2.808991 1.103907 0.314854
H 2.430339 0.477356 1.819588
H 0.521468 0.483736 0.229622
H 2.495887 2.131314 1.608197
C 0.523852 2.647815 0.167359
H 0.969599 2.566895 -0.83645
O 1.260319 3.594654 0.933383
H 1.013444 4.488651 0.686172
C -0.306742 -2.578884 0.201777
H -0.574465 -1.753712 -0.473148
H -0.330254 -3.48335 -0.419606
C -1.349048 -2.691264 1.307869
H -1.447752 -1.718949 1.808854
H -0.988427 -3.384515 2.077266
C -3.254853 2.125132 -0.437975
H -3.582767 3.170235 -0.489043
H -3.37791 1.825583 0.611199
C -1.773607 2.044151 -0.789286
H -1.431769 1.01468 -0.629068
H -1.623427 2.2453 -1.856554
O 3.926751 0.917216 -0.919568
H 4.251796 1.716416 -1.33913

conf_07

1 1
C -4.2658 -0.61609 1.837622
C -4.415534 -0.656825 0.334383
H -4.790962 0.255984 2.233509
H -3.218388 -0.551082 2.140636
H -4.704293 -1.502111 2.303193
C -2.608192 1.168662 0.006232
H -1.834682 0.394749 -0.048151

H -2.680687 1.479124 1.055106
C 0.224428 2.426406 0.013619
C 1.37302 3.332164 0.449182
H 0.50065 1.880615 -0.897579
H 0.060627 1.666917 0.79136
C 2.599681 2.584657 0.96264
H 1.012625 4.002275 1.239335
H 1.657231 3.979805 -0.390026
C 3.264588 1.672885 -0.060447
H 2.310477 1.985773 1.838239
H 3.3343 3.311245 1.328572
C 4.449895 0.904165 0.511316
H 3.589162 2.262543 -0.928277
H 2.52371 0.963941 -0.446311
H 4.14975 0.438837 1.458901
H 5.244176 1.612268 0.774055
C 3.404219 -2.003487 0.295503
C 2.410139 -3.082968 -0.129398
H 2.909664 -1.298377 0.975187
H 4.196178 -2.471954 0.891564
C 1.235487 -2.576118 -0.970866
H 2.03851 -3.592093 0.769563
H 2.933281 -3.85482 -0.70505
C 0.491675 -1.384279 -0.37206
H 0.538898 -3.405292 -1.157126
H 1.596012 -2.28467 -1.962095
C -0.164977 -1.646439 0.965673
H -0.270723 -1.02894 -1.077312
H 1.185061 -0.549036 -0.23662
H -0.565632 -0.725314 1.394401
H 0.540794 -2.059943 1.691941
N -3.681675 -1.836919 -0.246292
H -3.574877 -1.648536 -1.252174
H -4.223334 -2.69133 -0.128253
H -5.46186 -0.815923 0.059641
H -2.732718 -2.01387 0.177918
C -3.951485 0.59678 -0.416696
H -4.727192 1.356855 -0.249991
O -3.964389 0.189201 -1.779734
H -3.731968 0.926464 -2.349644
C 4.038364 -1.240058 -0.861705
H 4.557871 -1.95108 -1.515619
H 3.258532 -0.785605 -1.484911
C 5.027264 -0.158949 -0.423085
H 5.432733 0.3282 -1.317853
H 5.880177 -0.636158 0.074068
C -1.065411 3.197738 -0.236218
H -1.410178 3.64254 0.70617
H -0.855317 4.038907 -0.9064
C -2.196355 2.371076 -0.842429
H -3.06763 3.024352 -0.985866
H -1.886727 2.031355 -1.840088
O -1.294903 -2.535328 0.858644
H -0.973219 -3.42508 0.689137

conf_08

1 1
C -2.14232 -3.193297 -2.017178
C -1.353886 -1.9283 -1.751645
H -3.196405 -3.020208 -1.798265
H -1.795712 -4.013708 -1.38379
H -2.075217 -3.493078 -3.067236
C -2.634174 -1.395948 0.383419
H -3.052811 -2.401602 0.488804
H -3.304187 -0.838476 -0.282379
C -2.850022 1.683661 0.898364
C -2.27701 3.099999 0.873808
H -3.876331 1.718988 1.28255
H -2.934506 1.311007 -0.129833
C -0.912958 3.224451 0.197518
H -2.200515 3.468718 1.903526
H -2.987371 3.763981 0.367459
C -0.928208 2.985969 -1.311519
H -0.202317 2.539064 0.677364
H -0.51378 4.228127 0.38202
C 0.470747 2.796508 -1.894405
H -1.426984 3.831018 -1.797719
H -1.535394 2.10282 -1.554541
H 1.148514 3.540881 -1.457636
H 0.4679 2.985788 -2.973197
C 3.078215 -0.132413 -1.576261
C 2.788941 -0.660011 -0.169016
H 4.162467 -0.119038 -1.733798
H 2.702 -0.849628 -2.324809
C 3.28709 0.267711 0.933898
H 3.260734 -1.643625 -0.052528
H 1.714387 -0.830012 -0.031661
C 3.125048 -0.254939 2.358462
H 2.772885 1.234532 0.862899
H 4.348343 0.481523 0.759328
C 1.693074 -0.452215 2.828878
H 3.594538 0.461478 3.042045
H 3.677677 -1.19501 2.488853
H 1.049359 0.354926 2.4677
H 1.642806 -0.440539 3.922479
N 0.086063 -2.172696 -2.128799
H 0.611931 -1.299002 -2.227111
H 0.191743 -2.728094 -2.9763
H -1.715815 -1.096789 -2.360623
H 0.465224 -2.671968 -1.298507
C -1.273209 -1.512282 -0.27672
H -0.772922 -0.533569 -0.248519
O -0.440398 -2.487504 0.326411
H 0.03437 -2.117602 1.101296
C 2.520264 1.25812 -1.878008
H 3.045824 2.000088 -1.266587

H 2.748058 1.519749 -2.917415
C 1.026776 1.401354 -1.628903
H 0.803791 1.12196 -0.591819
H 0.467474 0.693852 -2.268609
C -2.043486 0.707495 1.747828
H -0.996704 0.706452 1.418174
H -2.017606 1.073115 2.780983
C -2.580902 -0.722134 1.754702
H -1.97408 -1.335019 2.430699
H -3.59336 -0.725507 2.172338
O 1.09026 -1.66365 2.355329
H 1.496763 -2.409856 2.80271

conf_09

1 1
C 0.505218 3.800318 -1.988293
C 0.330806 2.313055 -1.760487
H 1.559546 4.065021 -1.905429
H -0.049428 4.380373 -1.246241
H 0.178583 4.090775 -2.991368
C 1.986208 2.271184 0.161862
H 1.995143 3.362092 0.246058
H 2.731145 2.00216 -0.597154
C 3.461383 -0.482545 0.61657
C 3.497072 -2.008713 0.690617
H 4.45301 -0.087282 0.866198
H 3.269418 -0.171094 -0.417583
C 2.21371 -2.700094 0.23329
H 3.715983 -2.30542 1.723305
H 4.335319 -2.376266 0.087321
C 1.93935 -2.591721 -1.265228
H 1.359245 -2.306249 0.798371
H 2.268121 -3.762112 0.497211
C 0.510392 -2.977177 -1.641231
H 2.652774 -3.227511 -1.800241
H 2.132085 -1.568711 -1.617337
H 0.214737 -3.874148 -1.082141
H 0.456211 -3.249758 -2.701009
C -2.925307 -1.104911 -1.527412
C -2.875232 -0.23104 -0.27522
H -3.947148 -1.476652 -1.663559
H -2.735476 -0.488195 -2.423101
C -3.381669 -0.913092 0.990622
H -3.486672 0.667985 -0.439033
H -1.851848 0.109416 -0.083872
C -3.307832 -0.018633 2.230799
H -2.817002 -1.835085 1.173141
H -4.419874 -1.220746 0.825558
C -2.014337 -0.099066 3.009181
H -4.097887 -0.299641 2.935099
H -3.508973 1.025192 1.958331
H -1.840745 -1.13306 3.330113

H -2.074036 0.533779 3.901587
N -1.119992 1.969418 -1.977786
H -1.272613 0.963542 -2.095923
H -1.534294 2.466769 -2.764666
H 0.915688 1.730158 -2.475678
H -1.572638 2.241511 -1.08375
C 0.615752 1.837499 -0.328059
H 0.561136 0.739456 -0.333575
O -0.446452 2.354192 0.457401
H -0.65706 1.722045 1.175704
C -1.946307 -2.276024 -1.540744
H -2.208576 -2.994219 -0.755653
H -2.059659 -2.816516 -2.487282
C -0.4916 -1.861062 -1.368872
H -0.337059 -1.469014 -0.355675
H -0.26299 -1.030657 -2.062404
C 2.43152 0.142973 1.550824
H 1.444358 -0.2754 1.318339
H 2.657121 -0.170382 2.578121
C 2.372553 1.668551 1.51146
H 1.67125 2.034259 2.271138
H 3.34995 2.073019 1.795113
O -0.901707 0.320793 2.201056
H -0.091735 0.127756 2.680726

conf_10

1 1
C 3.625548 -2.062373 1.783204
C 3.110008 -0.653012 1.622679
H 4.647367 -2.115557 1.400758
H 3.024074 -2.786241 1.227733
H 3.655718 -2.362311 2.833689
C 2.617387 -1.011237 -0.906526
H 3.316664 -1.850226 -0.996788
H 2.699646 -0.461465 -1.851464
C -0.756628 -2.754239 -1.820733
C -1.804326 -1.643949 -1.795403
H -0.797589 -3.336152 -0.890941
H -0.998316 -3.459874 -2.622282
C -3.155036 -2.052271 -1.212911
H -1.951587 -1.273747 -2.816436
H -1.421473 -0.785705 -1.229561
C -3.113703 -2.523435 0.244973
H -3.593915 -2.849097 -1.824261
H -3.840511 -1.202187 -1.301802
C -2.324683 -1.619504 1.190351
H -2.694092 -3.534323 0.297997
H -4.142915 -2.614647 0.610141
H -1.262831 -1.64441 0.899133
H -2.371012 -2.035987 2.204523
C -2.228461 2.206051 2.01679
C -2.068286 2.831777 0.631847

H -1.590653 2.74694 2.727178
H -3.257922 2.360597 2.359428
C -0.660464 2.727028 0.058235
H -2.346314 3.89004 0.696228
H -2.780593 2.382427 -0.070347
C -0.532211 3.390869 -1.306739
H -0.370176 1.674044 -0.03507
H 0.051992 3.186817 0.758081
C 0.884556 3.48215 -1.824191
H -0.919952 4.415639 -1.253054
H -1.151011 2.860644 -2.039767
H 1.512049 4.058907 -1.133567
H 0.898323 3.983158 -2.796186
N 1.695483 -0.506736 2.125446
H 1.378132 0.391481 1.705441
H 1.63015 -0.477507 3.141232
H 3.684955 0.038827 2.244386
H 1.082672 -1.24494 1.772984
C 3.110347 -0.076811 0.194984
H 4.156137 0.182431 -0.019509
O 2.332432 1.096109 0.317532
H 2.063525 1.460785 -0.55853
C -1.904138 0.715667 2.090537
H -0.855561 0.56371 1.790439
H -1.973992 0.382147 3.133524
C -2.782667 -0.167999 1.215799
H -2.790067 0.226461 0.194675
H -3.820449 -0.112885 1.56726
C 0.66677 -2.234453 -2.005236
H 1.339323 -3.064569 -2.250339
H 0.704119 -1.542757 -2.856442
C 1.190381 -1.523466 -0.761657
H 0.526396 -0.685384 -0.519087
H 1.125988 -2.246612 0.067586
O 1.451037 2.162788 -1.970354
H 2.05121 2.172759 -2.718544

conf_11

1 1
C -4.632635 -0.511745 1.66381
C -4.418576 -0.790418 0.194148
H -5.349443 0.304129 1.779961
H -3.705029 -0.216746 2.159881
H -5.045706 -1.383688 2.177568
C -2.758722 1.162263 -0.075483
H -1.928279 0.477412 0.131923
H -3.059745 1.59689 0.884968
C 0.042814 2.467023 0.029181
C 1.146258 3.418035 0.477769
H 0.38846 1.874827 -0.828201
H -0.156825 1.749535 0.837161
C 2.385454 2.714961 1.022082

H 0.747322 4.089555 1.247943
H 1.430623 4.059745 -0.366251
C 3.060214 1.782699 0.025072
H 2.112285 2.143543 1.920543
H 3.107177 3.46991 1.354996
C 4.32312 1.132295 0.571854
H 3.301521 2.337942 -0.892059
H 2.352685 1.000657 -0.275237
H 4.083734 0.613063 1.508921
H 5.041738 1.913962 0.844465
C 3.533008 -1.834006 0.237628
C 2.756795 -3.056177 -0.244991
H 2.894753 -1.216788 0.884113
H 4.355797 -2.174213 0.878005
C 1.502103 -2.749024 -1.064734
H 2.489109 -3.678454 0.618325
H 3.417911 -3.679557 -0.857707
C 0.466702 -1.872453 -0.359296
H 1.030099 -3.697132 -1.350433
H 1.782549 -2.260271 -2.002794
C 0.029209 -2.448503 0.968014
H -0.409787 -1.767211 -1.012517
H 0.853112 -0.860464 -0.198057
H 0.830516 -2.372151 1.707083
H -0.23832 -3.507953 0.867223
N -3.417612 -1.896587 -0.001566
H -3.153835 -1.873657 -0.99574
H -3.822327 -2.80545 0.215522
H -5.341075 -1.156749 -0.264752
H -2.547764 -1.779169 0.582823
C -3.941487 0.396619 -0.646185
H -4.800309 1.074725 -0.744366
O -3.649989 -0.196107 -1.907107
H -3.34749 0.474336 -2.524673
C 4.104769 -0.979075 -0.88705
H 4.69082 -1.620881 -1.556402
H 3.291606 -0.574575 -1.502067
C 4.994321 0.164287 -0.400595
H 5.35575 0.723432 -1.271924
H 5.885275 -0.257795 0.079621
C -1.239585 3.191009 -0.359839
H -1.667018 3.685145 0.522157
H -0.997543 3.992465 -1.066845
C -2.29321 2.288319 -0.994407
H -3.159052 2.896039 -1.287807
H -1.871197 1.868348 -1.918396
O -1.119393 -1.71688 1.453246
H -1.127064 -1.73939 2.413002

conf_12

1 1
C -3.319963 0.137198 2.497472

C -4.088361 -0.108074 1.219376
H -3.467644 1.170071 2.820458
H -2.24758 -0.024524 2.365513
H -3.674833 -0.510404 3.303289
C -2.292628 0.991563 -0.255267
H -1.851196 0.012375 -0.480403
H -1.796701 1.358603 0.647858
C 0.363029 2.514722 -0.604683
C 1.845026 2.515754 -0.954388
H 0.211753 1.869834 0.268151
H 0.055391 3.522931 -0.295096
C 2.750192 2.966321 0.190407
H 2.015799 3.16429 -1.821526
H 2.132885 1.506899 -1.278357
C 2.566475 2.192281 1.497402
H 2.57328 4.029709 0.391216
H 3.792633 2.896475 -0.138407
C 2.695566 0.67543 1.380346
H 1.587623 2.434959 1.928364
H 3.303274 2.554851 2.224008
H 1.953372 0.293726 0.666595
H 2.439529 0.227166 2.349434
C 4.133668 -1.948797 -0.445486
C 2.807557 -1.740993 -1.169307
H 4.331997 -3.025548 -0.372768
H 4.933443 -1.539112 -1.073599
C 1.610501 -2.412916 -0.506662
H 2.901739 -2.133011 -2.189354
H 2.605977 -0.668178 -1.280683
C 0.340609 -2.261726 -1.33593
H 1.470609 -1.984421 0.49593
H 1.826774 -3.479745 -0.361239
C -0.86896 -2.958508 -0.753927
H 0.511131 -2.680923 -2.334802
H 0.110775 -1.199079 -1.48171
H -0.670882 -4.023791 -0.590481
H -1.726721 -2.877458 -1.427041
N -3.864298 -1.510834 0.724769
H -4.253359 -1.550926 -0.225741
H -4.342522 -2.193735 1.309912
H -5.164485 -0.038389 1.400663
H -2.846508 -1.778223 0.683271
C -3.771277 0.834206 0.054751
H -4.195223 1.811971 0.321688
O -4.502026 0.267748 -1.026916
H -4.40469 0.80996 -1.813316
C 4.252245 -1.326672 0.945542
H 3.532887 -1.789787 1.634037
H 5.238355 -1.595964 1.336613
C 4.090936 0.198573 0.988253
H 4.373794 0.620146 0.0153
H 4.800636 0.620119 1.709326
C -0.52094 2.040097 -1.749909
H -0.395605 2.708696 -2.608525

H -0.176488 1.052749 -2.086756
C -2.006281 1.956217 -1.402276
H -2.381444 2.953771 -1.139431
H -2.548551 1.64551 -2.305227
O -1.305825 -2.370917 0.486788
H -0.570688 -2.358471 1.106184

conf_13

1 1
C 1.189349 4.223351 -1.132108
C 1.30951 2.990788 -0.258438
H 0.579127 4.97776 -0.635131
H 0.722881 3.983505 -2.090442
H 2.170129 4.67406 -1.311281
C -1.11582 3.031119 0.522299
H -1.286455 3.910432 -0.105753
H -0.791181 3.391446 1.507093
C -3.668679 0.280601 1.692634
C -4.362953 -0.130175 0.392605
H -4.345152 0.928804 2.261707
H -3.505936 -0.603704 2.317972
C -3.502692 -0.938842 -0.574919
H -4.738777 0.760197 -0.124472
H -5.25208 -0.717287 0.649189
C -2.935434 -2.224155 0.013291
H -2.679267 -0.31242 -0.950335
H -4.102128 -1.182189 -1.460424
C -2.231182 -3.11629 -1.005673
H -3.753861 -2.793279 0.47035
H -2.248481 -1.986161 0.835511
H -2.960411 -3.447085 -1.754432
H -1.889528 -4.027515 -0.502284
C 0.632408 -2.90828 0.142485
C 1.858499 -2.384866 0.885167
H 0.900798 -3.825152 -0.398666
H -0.131908 -3.201384 0.86931
C 3.132343 -2.399724 0.043082
H 2.033483 -2.99465 1.779169
H 1.649916 -1.368044 1.245678
C 4.366204 -1.840127 0.754931
H 2.976745 -1.855115 -0.897709
H 3.328477 -3.437027 -0.247377
C 4.619472 -0.365867 0.528536
H 5.269661 -2.357535 0.413236
H 4.303919 -2.043314 1.832666
H 4.764462 -0.166528 -0.537726
H 5.519612 -0.033033 1.055352
N 2.263884 2.020168 -0.900748
H 2.678319 1.35689 -0.195565
H 3.027035 2.504568 -1.370586
H 1.72927 3.24492 0.717956
H 1.716368 1.496782 -1.596693

C 0.011703 2.203 -0.068809
H 0.231948 1.36164 0.606043
O -0.279162 1.693411 -1.364014
H -1.007639 1.067763 -1.312867
C 0.034827 -1.898651 -0.831327
H -0.363619 -1.055476 -0.247798
H 0.827184 -1.481286 -1.466505
C -1.05535 -2.467424 -1.736549
H -0.604885 -3.214524 -2.400693
H -1.424424 -1.672922 -2.396577
C -2.33197 0.993699 1.505579
H -1.920463 1.247151 2.490876
H -1.618808 0.287892 1.058095
C -2.425571 2.258088 0.660529
H -2.809239 2.019304 -0.338796
H -3.171756 2.929544 1.098725
O 3.515931 0.475772 0.914864
H 3.287331 0.300878 1.831742

conf_14

1 1
C 0.313626 -2.135213 1.270356
C 0.802205 -1.861722 -0.137034
H 0.904532 -1.560436 1.983485
H 0.409516 -3.19447 1.52078
H -0.72892 -1.830296 1.384343
C 3.310501 -1.964354 0.508066
H 4.178283 -2.586301 0.256175
H 3.04322 -2.22427 1.537844
C 3.347515 1.944291 1.015805
C 3.357346 2.450206 -0.4292
H 4.357043 2.045392 1.430692
H 2.70845 2.587246 1.630114
C 2.061058 2.216285 -1.200046
H 4.184652 1.985161 -0.977239
H 3.580288 3.523121 -0.420085
C 0.838083 2.899708 -0.604307
H 1.867645 1.135022 -1.277764
H 2.194026 2.559918 -2.232564
C -0.451861 2.532473 -1.325738
H 0.981156 3.987397 -0.642211
H 0.747872 2.647214 0.458741
H -0.651539 1.459665 -1.174226
H -0.308622 2.658468 -2.406083
C -2.19702 1.887696 1.15993
C -3.272666 1.081591 0.443525
H -2.456724 1.969704 2.222213
H -1.250743 1.328148 1.129199
C -3.599768 -0.227113 1.151383
H -2.950706 0.886523 -0.587205
H -4.187509 1.682395 0.35997
C -4.511313 -1.15302 0.352061

H -4.072171 -0.003819 2.113593
H -2.669799 -0.75703 1.395657
C -3.847503 -1.788956 -0.851541
H -5.38906 -0.600494 -0.004492
H -4.904568 -1.94995 0.997014
H -3.413875 -1.037643 -1.515342
H -4.56305 -2.376163 -1.435069
N -0.117156 -2.555681 -1.109411
H -0.006718 -2.1897 -2.05461
H -1.133877 -2.505503 -0.840995
H 0.753107 -0.795764 -0.367888
H 0.172811 -3.541765 -1.127886
C 2.204596 -2.401476 -0.441641
H 2.479201 -2.074338 -1.458392
O 2.022703 -3.814207 -0.43812
H 2.866643 -4.259057 -0.545952
C -1.991996 3.291241 0.596849
H -2.899335 3.879837 0.776404
H -1.199198 3.788567 1.166581
C -1.666919 3.349392 -0.89553
H -1.50109 4.39684 -1.173052
H -2.538685 3.025736 -1.476056
C 2.880066 0.499464 1.178402
H 2.908917 0.232462 2.241828
H 1.825572 0.439041 0.883336
C 3.726718 -0.497352 0.393949
H 3.755759 -0.217879 -0.666428
H 4.76025 -0.409705 0.745264
O -2.745078 -2.639095 -0.475573
H -3.069682 -3.325937 0.113993

conf_15

1 1
C -4.227888 -1.093526 1.639282
C -4.50954 -0.949719 0.161265
H -5.049273 -0.653897 2.209406
H -3.30471 -0.588633 1.931634
H -4.157515 -2.145183 1.928599
C -3.789465 1.56619 0.167765
H -3.958296 1.708848 1.24139
H -4.095398 2.510652 -0.300599
C 0.069793 2.086001 0.026439
C 1.056615 3.225519 0.246148
H 0.217384 1.683624 -0.987169
H 0.299818 1.270169 0.727519
C 2.502628 2.840075 -0.050704
H 0.972415 3.587463 1.279006
H 0.76972 4.065653 -0.397341
C 3.087104 1.806931 0.906129
H 3.127423 3.740263 -0.017362
H 2.562462 2.470991 -1.082477
C 4.536073 1.434484 0.601258

H 2.473715 0.896466 0.89821
H 3.027216 2.197206 1.929819
H 4.898197 0.754577 1.380526
H 5.159451 2.333191 0.678925
C 4.185164 -1.593302 -0.101041
C 3.465557 -2.889799 -0.466527
H 3.925867 -1.298553 0.92315
H 5.263397 -1.791943 -0.081718
C 1.939778 -2.811914 -0.47016
H 3.775247 -3.677856 0.230806
H 3.799089 -3.215066 -1.458862
C 1.35363 -2.418227 0.880674
H 1.537489 -3.791269 -0.757533
H 1.603766 -2.11134 -1.243288
C -0.155163 -2.490078 0.950366
H 1.671899 -1.404483 1.161307
H 1.749366 -3.076765 1.663724
H -0.506523 -2.257451 1.962502
H -0.509247 -3.489756 0.682425
N -3.423383 -1.583792 -0.670116
H -3.491326 -1.168223 -1.6089
H -3.575089 -2.587415 -0.75638
H -5.412741 -1.506859 -0.101712
H -2.441661 -1.457612 -0.311164
C -4.740311 0.486895 -0.336087
H -5.757361 0.744718 -0.013414
O -4.705519 0.351634 -1.75211
H -4.919628 1.185752 -2.176907
C 3.920238 -0.438812 -1.06016
H 4.125291 -0.775234 -2.084222
H 2.854803 -0.171022 -1.045306
C 4.760813 0.803875 -0.774492
H 4.571066 1.548113 -1.555925
H 5.821599 0.542423 -0.867233
C -1.39197 2.477718 0.199791
H -1.560683 2.831633 1.224405
H -1.631605 3.319521 -0.461028
C -2.315932 1.306701 -0.102046
H -2.178276 1.015214 -1.152022
H -1.987065 0.460736 0.511522
O -0.786949 -1.590565 0.018675
H -0.249596 -0.794744 -0.053418

4E-3-DeoxySO

conf_00

1 1
C 1.403461 -2.109319 1.998782
C 0.572978 -1.520785 0.870969
H 2.013965 -1.331413 2.47572
H 2.074277 -2.86703 1.58007
C -0.997425 -1.880214 -1.011448

C -2.330016 -1.967379 -0.991302
H -0.524578 -1.229453 -1.748999
C -3.244571 -1.210301 -1.895303
H -2.801373 -2.639652 -0.269979
C -4.338991 -0.450722 -1.134462
H -3.727677 -1.913193 -2.584638
H -2.656683 -0.525419 -2.514935
C -3.834031 0.435804 0.000669
H -5.058741 -1.168838 -0.725944
H -4.895121 0.159475 -1.85384
C -2.775121 1.456375 -0.396711
H -3.443936 -0.201319 0.812003
H -4.688148 0.958097 0.44618
C -2.407333 2.404992 0.742026
H -3.135604 2.044952 -1.249119
H -1.879625 0.937449 -0.766204
C -1.152116 3.235131 0.484305
H -2.277373 1.835011 1.676762
H -3.257054 3.068268 0.933601
H -1.11031 4.071554 1.189957
H -1.208385 3.682425 -0.515629
C 2.669699 2.344527 0.377946
C 2.83221 1.354007 -0.769992
H 2.672874 1.803493 1.336707
H 3.551697 2.993151 0.406562
C 4.016738 0.413712 -0.584127
H 2.956944 1.910394 -1.707683
H 1.909086 0.773203 -0.901878
C 4.2355 -0.564235 -1.734844
H 3.894797 -0.150186 0.353825
H 4.921942 1.016487 -0.447989
C 3.139805 -1.612384 -1.875954
H 5.194703 -1.072231 -1.591523
H 4.328333 -0.003243 -2.672241
H 3.053221 -2.207011 -0.957829
H 3.355167 -2.307341 -2.691262
H 2.164577 -1.1611 -2.088658
N -0.525618 -0.71206 1.503056
H -1.31864 -0.667761 0.841168
H -0.240311 0.247625 1.71651
H 1.17887 -0.831309 0.279248
H -0.815637 -1.194731 2.362902
C -0.083375 -2.559781 -0.04236
H -0.624067 -3.293451 0.564326
C 0.135442 2.429705 0.601156
H 0.218796 2.062642 1.642105
H 0.078737 1.556103 -0.063297
C 1.410414 3.202693 0.290607
H 1.488085 4.046316 0.9849
H 1.33168 3.638958 -0.712838
H 0.722262 -3.088722 -0.56053
O 0.473759 -2.661222 2.90988
H 0.922892 -3.088954 3.642288

conf_01

1 1
C -2.972611 -2.239759 -1.617886
C -2.638145 -0.771656 -1.41224
H -2.403406 -2.651593 -2.461804
H -4.040361 -2.325465 -1.847687
C -3.082397 1.335858 -0.139907
C -2.468295 1.693363 0.991551
H -3.220686 2.077182 -0.927272
C -1.899912 3.038437 1.297297
H -2.359807 0.943783 1.780162
C -0.370704 2.997154 1.424109
H -2.328066 3.390439 2.242448
H -2.193085 3.758056 0.525435
C 0.342516 2.820543 0.090597
H -0.084913 2.187616 2.109708
H -0.027005 3.923297 1.895324
C 1.833329 2.546751 0.233846
H 0.186981 3.715072 -0.526339
H -0.128504 2.002297 -0.47431
C 2.58801 2.45089 -1.090314
H 1.974255 1.625626 0.815405
H 2.284633 3.342742 0.836865
C 2.055922 1.400613 -2.066513
H 3.644063 2.252551 -0.879405
H 2.563042 3.426557 -1.589047
H 2.704379 1.379855 -2.949482
H 1.069575 1.712573 -2.434261
C 3.185593 -2.089208 -0.653585
C 2.112723 -2.4248 0.381546
H 4.15994 -2.416072 -0.273667
H 3.000994 -2.682586 -1.557793
C 2.235607 -1.648894 1.686997
H 2.155116 -3.498986 0.598148
H 1.119092 -2.261188 -0.059855
C 1.204642 -2.027504 2.745993
H 2.159085 -0.570684 1.491257
H 3.241038 -1.805759 2.094683
C -0.23551 -1.737222 2.340662
H 1.428832 -1.480584 3.6675
H 1.303438 -3.090995 2.992805
H -0.341856 -0.691467 2.021277
H -0.926609 -1.893098 3.17291
H -0.569076 -2.401756 1.533772
N -1.250051 -0.701876 -0.851416
H -1.122442 0.226332 -0.416947
H -0.503821 -0.845402 -1.534728
H -2.624738 -0.246232 -2.370557
H -1.155285 -1.416982 -0.117014
C -3.562351 -0.055214 -0.421112
H -3.61448 -0.642619 0.501339
C 1.957456 -0.010819 -1.49327

H 1.275921 -0.002117 -0.630875
H 1.51949 -0.675506 -2.257795
C 3.280235 -0.612447 -1.035932
H 3.664834 -0.03333 -0.189201
H 4.02026 -0.500093 -1.836388
H -4.565332 -0.046534 -0.858433
O -2.63094 -2.88199 -0.407561
H -2.86427 -3.812398 -0.436802

conf_02

1 1
C -0.743923 2.812943 1.455548
C -0.060299 1.856009 0.493482
H -1.440237 2.271595 2.109322
H -1.314253 3.545344 0.874345
C 1.465482 1.524729 -1.434512
C 2.795328 1.398382 -1.424962
H 0.874105 0.812592 -2.012304
C 3.552147 0.325731 -2.134733
H 3.38822 2.128014 -0.867451
C 4.521346 -0.430797 -1.216688
H 4.130904 0.777938 -2.949063
H 2.84575 -0.365507 -2.605448
C 3.898806 -0.971792 0.067751
H 5.357209 0.226926 -0.953358
H 4.955521 -1.258305 -1.787487
C 2.672755 -1.853219 -0.132097
H 3.640664 -0.127437 0.728975
H 4.661165 -1.531526 0.621154
C 2.153167 -2.464068 1.167764
H 2.913121 -2.660164 -0.834878
H 1.878696 -1.273801 -0.622463
C 0.750205 -3.057115 1.055194
H 2.160797 -1.705119 1.967516
H 2.856885 -3.232566 1.503308
H 0.552487 -3.712469 1.909871
H 0.693479 -3.693 0.163256
C -2.777666 -1.399522 0.587926
C -2.707712 -0.704901 -0.769161
H -2.645996 -0.658679 1.392767
H -3.783598 -1.805992 0.730038
C -3.532092 0.577431 -0.858105
H -3.032594 -1.407622 -1.544848
H -1.663212 -0.47375 -1.022717
C -5.027487 0.402858 -0.595847
H -3.395652 1.019173 -1.853292
H -3.138372 1.311767 -0.138448
C -5.709152 -0.56169 -1.556126
H -5.506382 1.385283 -0.669127
H -5.187082 0.070639 0.436721
H -5.551517 -0.261267 -2.597161
H -6.787135 -0.591602 -1.380644

H -5.334888 -1.583997 -1.446189
N 0.933531 1.05887 1.290855
H 1.680031 0.736859 0.651888
H 0.515785 0.233172 1.727698
H -0.785861 1.146916 0.089291
H 1.330435 1.678355 2.008581
C 0.700942 2.540301 -0.645912
H 1.36769 3.304825 -0.233955
C -0.338573 -1.994403 0.983096
H -0.340316 -1.436559 1.938865
H -0.095534 -1.285619 0.179281
C -1.751322 -2.517275 0.76035
H -2.03069 -3.155256 1.605507
H -1.761302 -3.162817 -0.126611
H -0.040037 3.051946 -1.268197
O 0.297011 3.413076 2.200805
H -0.05081 4.063398 2.815012

conf_03

1 1
C 1.472434 -2.500355 1.825654
C 0.654686 -1.747999 0.789405
H 2.018854 -1.800809 2.471782
H 2.201051 -3.130368 1.304606
C -0.794069 -1.81272 -1.216585
C -2.124367 -1.913436 -1.275646
H -0.30451 -1.035009 -1.804796
C -2.995423 -0.9986 -2.071537
H -2.622671 -2.704499 -0.709922
C -4.095375 -0.334551 -1.231057
H -3.472176 -1.565074 -2.880066
H -2.370303 -0.23971 -2.553204
C -3.614476 0.301068 0.070878
H -4.867506 -1.074654 -0.993848
H -4.584262 0.424876 -1.850131
C -2.461449 1.287258 -0.070258
H -3.328809 -0.497441 0.77677
H -4.460468 0.802529 0.554198
C -2.089927 1.960171 1.252832
H -2.718982 2.045689 -0.817306
H -1.579758 0.77562 -0.484446
C -0.646782 2.451496 1.319125
H -2.27313 1.260272 2.083007
H -2.771051 2.7974 1.440663
H 0.037689 1.586803 1.269275
H -0.459957 2.921414 2.292173
C 2.238442 2.774594 0.35536
C 2.161366 1.756712 -0.775277
H 2.158698 2.250204 1.317869
H 3.235944 3.228184 0.358291
C 3.205607 0.654885 -0.657801
H 2.273333 2.267254 -1.740135

H 1.160025 1.300321 -0.802522
C 3.108482 -0.403927 -1.748672
H 3.120492 0.175973 0.33142
H 4.209319 1.096598 -0.669722
C 4.055433 -1.575194 -1.536213
H 3.309101 0.062119 -2.719928
H 2.073933 -0.770696 -1.814169
H 5.096368 -1.240689 -1.508244
H 3.96886 -2.316598 -2.334621
H 3.856723 -2.083034 -0.584416
N -0.514385 -1.132755 1.508765
H -1.268833 -0.965034 0.822031
H -0.289523 -0.242496 1.956633
H 1.237678 -0.92697 0.366682
H -0.829869 -1.805012 2.219883
C 0.095627 -2.627176 -0.333532
H -0.439261 -3.47996 0.09707
C -0.254171 3.412438 0.204558
H -0.428818 2.936204 -0.767029
H -0.920452 4.282505 0.234492
C 1.195403 3.889746 0.286017
H 1.315028 4.534664 1.164353
H 1.402593 4.522939 -0.584043
H 0.95329 -3.021987 -0.887639
O 0.539051 -3.260467 2.567211
H 0.981001 -3.804966 3.222445

conf_04

1 1
C -3.105258 -2.869301 0.492259
C -2.105868 -1.808574 0.059027
H -2.865741 -3.242765 1.496358
H -3.052668 -3.707521 -0.210619
C -1.300932 -0.277474 -1.743379
C -1.750791 0.95228 -2.009651
H -0.226087 -0.457497 -1.729601
C -0.901482 2.14318 -2.301752
H -2.831655 1.115437 -2.04773
C -1.216933 3.333915 -1.388847
H -1.08364 2.448333 -3.339479
H 0.156257 1.869352 -2.240525
C -0.970495 3.082507 0.095251
H -2.262498 3.628125 -1.538119
H -0.613088 4.18838 -1.712344
C 0.489082 2.834561 0.454713
H -1.593181 2.237471 0.428606
H -1.335166 3.944317 0.666086
C 0.739928 2.629106 1.946453
H 1.076743 3.695652 0.116134
H 0.877224 1.976147 -0.106968
C 0.126305 1.360419 2.540798
H 0.35451 3.493724 2.498917

H 1.819431 2.61997 2.127053
H -0.970764 1.445542 2.514625
H 0.384655 1.312157 3.604557
C 2.502132 -1.540049 1.368968
C 2.10995 -1.815088 -0.082783
H 2.08111 -2.329021 2.005316
H 3.588822 -1.629783 1.470379
C 2.576429 -0.764267 -1.089427
H 1.01778 -1.91079 -0.159319
H 2.502503 -2.796229 -0.369979
C 4.085107 -0.526618 -1.118316
H 2.073769 0.190805 -0.885323
H 2.249527 -1.070534 -2.09194
C 4.895828 -1.767064 -1.466762
H 4.417057 -0.130039 -0.151317
H 4.295455 0.261225 -1.849697
H 4.795392 -2.549426 -0.708561
H 5.959346 -1.53003 -1.548142
H 4.577163 -2.190798 -2.424894
N -2.409354 -0.56577 0.847691
H -2.058723 0.246853 0.311963
H -1.961656 -0.556813 1.764949
H -1.088818 -2.108858 0.320655
H -3.430148 -0.504387 0.954237
C -2.187723 -1.441675 -1.425737
H -3.228865 -1.222939 -1.68503
C 0.57198 0.062059 1.867376
H 0.083038 -0.793751 2.364522
H 0.246277 0.052464 0.817662
C 2.076377 -0.177428 1.90868
H 2.422249 -0.078908 2.944217
H 2.582839 0.61441 1.346375
H -1.890199 -2.328603 -1.993864
O -4.36699 -2.231925 0.471777
H -5.068669 -2.84881 0.691556

conf_05

1 1
C -0.669371 -3.166166 -1.610159
C -1.209741 -1.764236 -1.382055
H 0.383073 -3.127598 -1.921057
H -1.249957 -3.63994 -2.409184
C -3.169874 -0.331961 -0.791926
C -3.397197 0.060424 0.465077
H -3.255396 0.394103 -1.599951
C -3.684975 1.456119 0.908478
H -3.335804 -0.687742 1.260563
C -2.415087 2.120483 1.466518
H -4.460734 1.436945 1.679597
H -4.072765 2.049604 0.073986
C -1.45532 2.578569 0.375181
H -1.909374 1.423346 2.150895

H -2.696736 2.981442 2.080686
C -0.074272 2.956172 0.896729
H -1.898368 3.437748 -0.144477
H -1.36058 1.799091 -0.39497
C 0.789672 3.703581 -0.116349
H 0.444905 2.050908 1.245507
H -0.18702 3.585143 1.787295
C 1.008632 2.986204 -1.447918
H 1.757484 3.93178 0.34286
H 0.324069 4.67407 -0.3236
H 1.650713 3.61246 -2.077697
H 0.05612 2.905034 -1.985007
C 3.776037 0.288029 -0.791375
C 3.057374 -0.979218 -0.335794
H 4.714402 0.373569 -0.231703
H 4.061439 0.169788 -1.843262
C 2.548329 -0.944271 1.101
H 3.738903 -1.830394 -0.447838
H 2.227776 -1.199711 -1.031438
C 2.080186 -2.304264 1.611178
H 1.740744 -0.201508 1.206706
H 3.343402 -0.571038 1.757228
C 1.303727 -2.224451 2.917279
H 2.954458 -2.950997 1.741638
H 1.475745 -2.811925 0.84405
H 1.907681 -1.766 3.705243
H 0.996352 -3.212374 3.268422
H 0.399624 -1.608723 2.818719
N -0.552097 -1.234361 -0.143158
H -1.11277 -0.441828 0.207691
H 0.412691 -0.927712 -0.296721
H -0.91869 -1.104262 -2.203239
H -0.538144 -1.985531 0.558043
C -2.726259 -1.714088 -1.159383
H -2.990701 -2.431194 -0.375479
C 1.641962 1.601427 -1.340711
H 0.940761 0.937219 -0.811857
H 1.756391 1.184167 -2.34966
C 2.98931 1.586184 -0.628693
H 2.84741 1.805601 0.435931
H 3.59973 2.408377 -1.019734
H -3.202818 -2.052791 -2.083774
O -0.814517 -3.834253 -0.374156
H -0.521994 -4.745805 -0.44152

conf_06

1 1
C -3.666764 -1.530451 -0.942403
C -2.928478 -0.217186 -1.095213
H -4.716504 -1.375875 -1.214529
H -3.626442 -1.870853 0.102616
C -2.441457 2.082984 -0.270272

C -1.507787 2.451837 0.608209
H -2.588021 2.669866 -1.177426
C -0.583759 3.615753 0.480896
H -1.396837 1.862631 1.522503
C 0.88467 3.192036 0.58741
H -0.806713 4.32135 1.290365
H -0.765818 4.145678 -0.460325
C 1.394698 2.402803 -0.611903
H 1.015967 2.594403 1.499521
H 1.506759 4.082376 0.721766
C 2.717281 1.704157 -0.326941
H 1.494528 3.068345 -1.478575
H 0.641487 1.654887 -0.904184
C 3.356162 0.995909 -1.518708
H 2.565029 0.991344 0.494557
H 3.428936 2.442862 0.059676
C 2.475074 -0.034128 -2.226758
H 4.278171 0.512539 -1.178223
H 3.669091 1.743374 -2.256695
H 3.077968 -0.544136 -2.98636
H 1.682227 0.484162 -2.782378
C 2.159124 -3.048061 0.284832
C 0.872511 -2.714377 1.039422
H 2.886434 -3.469053 0.987713
H 1.938124 -3.846748 -0.433176
C 0.982646 -1.569442 2.037483
H 0.525777 -3.613428 1.561684
H 0.078517 -2.494059 0.307803
C -0.33897 -1.219087 2.708271
H 1.383167 -0.671876 1.548388
H 1.715166 -1.833564 2.809827
C -0.226213 -0.071895 3.700111
H -0.745443 -2.106633 3.206889
H -1.088804 -0.955131 1.939732
H 0.456999 -0.331164 4.513538
H -1.191805 0.175998 4.148613
H 0.168529 0.830733 3.221663
N -1.464356 -0.514551 -0.959045
H -0.915056 0.345486 -1.070952
H -1.181254 -1.221072 -1.643792
H -3.047713 0.142643 -2.121801
H -1.24019 -0.887366 -0.029281
C -3.307775 0.871076 -0.098636
H -3.219203 0.482952 0.923307
C 1.837256 -1.079939 -1.315516
H 1.129308 -0.575981 -0.642637
H 1.254022 -1.779114 -1.93388
C 2.808635 -1.875639 -0.453115
H 3.28639 -1.202628 0.267397
H 3.619269 -2.261452 -1.081485
H -4.363686 1.113535 -0.258046
O -3.012814 -2.435708 -1.805834
H -3.476498 -3.275598 -1.836839

conf_07

1 1
C -3.433005 0.773705 -1.573135
C -2.051497 1.382101 -1.450326
H -4.096317 1.503042 -2.05043
H -3.834117 0.540055 -0.576259
C -0.514957 3.011554 -0.333182
C 0.230827 2.847656 0.761284
H -0.097878 3.53525 -1.193687
C 1.630899 3.321364 0.965435
H -0.219586 2.344043 1.621155
C 2.575333 2.183554 1.367893
H 1.614597 4.070151 1.766958
H 1.997921 3.82951 0.067148
C 2.977691 1.27606 0.209795
H 2.092956 1.585373 2.152842
H 3.475255 2.604632 1.826171
C 3.488356 -0.078126 0.687965
H 3.740898 1.775485 -0.399726
H 2.117335 1.136174 -0.461342
C 4.072969 -0.984073 -0.39374
H 2.66897 -0.59606 1.205972
H 4.255191 0.0851 1.454243
C 3.164178 -1.233769 -1.598805
H 4.347072 -1.939174 0.067532
H 5.012178 -0.552444 -0.757685
H 3.614375 -2.007172 -2.231033
H 3.127296 -0.329247 -2.217861
C 0.114698 -3.142181 -0.026016
C -0.330828 -2.210885 1.099965
H -0.03427 -4.17948 0.290402
H -0.541455 -3.007366 -0.899979
C -1.843561 -2.117434 1.268135
H 0.084994 -1.203287 0.940932
H 0.119237 -2.537952 2.044152
C -2.285026 -1.02239 2.232642
H -2.234319 -3.081739 1.612449
H -2.324853 -1.965454 0.286857
C -3.7943 -0.93095 2.393462
H -1.888169 -0.046046 1.902144
H -1.816615 -1.192573 3.208438
H -4.291719 -0.749257 1.433721
H -4.080432 -0.12735 3.076182
H -4.200166 -1.86431 2.793051
N -1.11921 0.281963 -1.041948
H -0.161802 0.639715 -0.955975
H -1.156338 -0.484709 -1.719298
H -1.706108 1.693112 -2.440982
H -1.376621 -0.110311 -0.129596
C -1.929736 2.527105 -0.453869
H -2.304289 2.202849 0.524752
C 1.734904 -1.632167 -1.248324

H 1.299454 -0.813 -0.660729
H 1.15013 -1.70959 -2.176726
C 1.563371 -2.925743 -0.460757
H 2.211256 -2.918488 0.423567
H 1.896438 -3.767471 -1.076941
H -2.589037 3.332807 -0.794343
O -3.267712 -0.394166 -2.348993
H -4.117192 -0.787526 -2.561031

conf_08

1 1
C 1.099893 3.710822 -0.124179
C 1.582975 2.312012 -0.472168
H 0.003404 3.758754 -0.151195
H 1.496787 4.413611 -0.864528
C 3.504085 0.787811 -0.937597
C 4.183764 0.058264 -0.048479
H 3.211294 0.33068 -1.883263
C 4.600307 -1.361909 -0.231901
H 4.496247 0.532717 0.886021
C 4.064782 -2.29111 0.863275
H 5.696087 -1.402365 -0.212038
H 4.294457 -1.713779 -1.22178
C 2.544179 -2.395548 0.926305
H 4.448452 -1.960196 1.835686
H 4.481734 -3.290199 0.697477
C 1.899551 -2.912303 -0.354986
H 2.118659 -1.415795 1.19603
H 2.273977 -3.058449 1.75687
C 0.423745 -3.277584 -0.207472
H 2.444298 -3.806239 -0.681391
H 2.02663 -2.181816 -1.163574
C -0.497365 -2.13597 0.215829
H 0.333672 -4.087585 0.525434
H 0.063434 -3.688866 -1.157663
H -0.198392 -1.761517 1.205463
H -1.506685 -2.537741 0.34981
C -2.980525 -0.192942 -0.522454
C -3.896796 0.943427 -0.086826
H -3.212382 -1.097752 0.047343
H -3.169152 -0.433552 -1.576466
C -5.380208 0.671938 -0.31728
H -3.612981 1.855992 -0.628037
H -3.731241 1.153616 0.979428
C -5.944183 -0.487388 0.496418
H -5.553921 0.48566 -1.385531
H -5.945102 1.580062 -0.075209
C -7.445692 -0.652808 0.313638
H -5.717424 -0.324093 1.558352
H -5.442987 -1.421618 0.21677
H -7.982701 0.248848 0.62434
H -7.831637 -1.488825 0.902386

H -7.696876 -0.841355 -0.734917
N 1.255589 1.431245 0.698416
H 1.865028 0.598628 0.661915
H 0.276238 1.125722 0.695246
H 1.028902 1.919426 -1.32842
H 1.445473 1.968788 1.553423
C 3.089412 2.209786 -0.718685
H 3.623277 2.648052 0.130958
C -0.546169 -0.975497 -0.771787
H 0.47008 -0.58106 -0.918719
H -0.845974 -1.345705 -1.759829
C -1.502186 0.143323 -0.357162
H -1.29728 1.054983 -0.938935
H -1.353538 0.392075 0.711668
H 3.318404 2.823413 -1.595589
O 1.591531 3.968373 1.175727
H 1.362959 4.855843 1.460796

conf_09

1 1
C 1.472449 2.500352 -1.8255
C 0.654617 1.747991 -0.789351
H 2.018753 1.800841 -2.471758
H 2.201173 3.130148 -1.304329
C -0.794273 1.812664 1.216521
C -2.124569 1.913301 1.275611
H -0.304643 1.034964 1.804687
C -2.995588 0.998398 2.07147
H -2.622924 2.704343 0.709907
C -4.095493 0.334389 1.230915
H -3.47235 1.564808 2.880037
H -2.370466 0.239487 2.553098
C -3.614446 -0.301163 -0.070996
H -4.867606 1.074497 0.993684
H -4.584431 -0.425079 1.849897
C -2.461422 -1.287357 0.070226
H -3.328716 0.497381 -0.776817
H -4.460369 -0.802623 -0.554435
C -2.089769 -1.960182 -1.252861
H -2.719013 -2.045834 0.817209
H -1.57977 -0.775749 0.484529
C -0.646609 -2.451454 -1.319107
H -2.272958 -1.260242 -2.083007
H -2.770869 -2.79741 -1.440782
H 0.037835 -1.58674 -1.2692
H -0.459733 -2.921336 -2.29216
C 2.238613 -2.774568 -0.355306
C 2.161456 -1.756614 0.775251
H 2.15895 -2.25025 -1.317861
H 3.236102 -3.228186 -0.358132
C 3.205718 -0.654804 0.657848
H 2.273305 -2.267116 1.740143

H 1.160129 -1.300184 0.802368
C 3.108354 0.404083 1.748627
H 3.120744 -0.175971 -0.33142
H 4.209441 -1.096477 0.669976
C 4.055281 1.575388 1.536299
H 3.308778 -0.061886 2.719958
H 2.073773 0.770803 1.81387
H 5.096237 1.240938 1.508521
H 3.968528 2.316802 2.334675
H 3.856696 2.083196 0.584461
N -0.514339 1.132695 -1.508802
H -1.268901 0.965065 -0.82219
H -0.289454 0.242391 -1.956563
H 1.237601 0.927031 -0.366496
H -0.829619 1.804955 -2.220017
C 0.095356 2.627204 0.333476
H -0.439634 3.479872 -0.097225
C -0.254021 -3.412427 -0.204559
H -0.428722 -2.936229 0.767031
H -0.920267 -4.282521 -0.234548
C 1.195566 -3.889703 -0.285942
H 1.315217 -4.534659 -1.164241
H 1.402736 -4.522839 0.584161
H 0.952911 3.02222 0.887597
O 0.539191 3.260845 -2.566833
H 0.981069 3.802977 -3.224078

5E,14Z-1-Deoxyspinghadiene

conf_00

1 1
C -0.237386 -2.241416 1.238882
C -0.647186 -2.368213 -0.210671
H -0.376023 -3.199605 1.744514
H -0.83515 -1.491748 1.762419
H 0.815864 -1.970018 1.326515
C -3.154406 -2.202786 0.402022
H -2.962053 -2.419413 1.457656
H -4.100982 -2.707928 0.167282
C -3.539768 1.640057 1.050415
C -2.354303 2.574039 1.312253
H -4.327979 1.855217 1.782084
H -3.966829 1.849544 0.063074
C -1.368946 2.612878 0.155256
H -1.841408 2.275132 2.235665
H -2.732501 3.58598 1.490081
C -0.14067 3.476291 0.407638
H -1.888527 2.966728 -0.74555
H -1.045319 1.587022 -0.073407
C 0.703102 3.717905 -0.843977
H 0.465981 3.024011 1.202374
H -0.461896 4.447079 0.801305

H 1.597215 4.289937 -0.570483
H 0.135972 4.354794 -1.532416
C 2.307967 0.239041 -1.526466
C 2.813604 -0.888137 -1.004724
H 2.144015 0.266889 -2.604716
C 3.24941 -1.080231 0.41244
H 3.024574 -1.71082 -1.68784
C 4.764738 -0.887307 0.5647
H 3.000045 -2.09438 0.749791
H 2.73716 -0.377601 1.080381
C 5.233867 -1.10956 1.993667
H 5.026222 0.123202 0.232568
H 5.283061 -1.575614 -0.112105
H 4.750536 -0.41105 2.684103
H 6.313331 -0.965535 2.079647
H 5.007759 -2.125347 2.332839
N -0.513948 -1.049904 -0.91224
H -0.938172 -1.149397 -1.841359
H 0.482862 -0.766837 -1.015548
H 0.031694 -3.038938 -0.744824
H -1.046104 -0.32778 -0.411057
C -2.072169 -2.876113 -0.442651
H -2.058635 -3.945909 -0.197614
O -2.275233 -2.693159 -1.836225
H -3.095586 -3.1054 -2.117361
C 2.007597 1.512757 -0.786812
H 1.550163 1.292303 0.186935
H 2.949205 2.024149 -0.546525
C 1.121689 2.457214 -1.593132
H 0.226464 1.920371 -1.937756
H 1.655711 2.743814 -2.505888
C -3.210464 0.187432 1.166105
H -2.899042 -0.146264 2.158976
C -3.334232 -0.726606 0.199488
H -3.676791 -0.405447 -0.784867

conf_01

1 1
C 1.052223 3.979408 -0.812027
C 1.376372 2.523442 -0.559009
H 0.871337 4.484296 0.138105
H 0.155764 4.08197 -1.427944
H 1.884678 4.496267 -1.297938
C -0.415369 2.197655 1.269158
H -0.905999 3.155322 1.07061
H 0.35641 2.359008 2.030439
C -3.685437 0.132062 1.799097
C -4.396027 -0.420207 0.556724
H -4.446894 0.519288 2.486256
H -3.165296 -0.670932 2.33109
C -3.477404 -0.978756 -0.527084
H -5.021021 0.368267 0.121276

H -5.085141 -1.208123 0.880387
C -2.570089 -2.116765 -0.071959
H -2.877261 -0.166616 -0.961301
H -4.102184 -1.337822 -1.353401
C -1.807813 -2.785676 -1.216527
H -3.191203 -2.872303 0.423626
H -1.868566 -1.762338 0.693787
H -2.512068 -2.981747 -2.033001
H -1.450596 -3.769141 -0.888417
C 1.847561 -1.477431 -1.474854
C 2.889519 -0.904017 -0.852046
H 1.931418 -1.630403 -2.552202
C 3.058778 -0.708885 0.622632
H 3.76256 -0.658561 -1.460798
C 4.038994 -1.724209 1.222286
H 2.099194 -0.795082 1.14368
H 3.445665 0.299269 0.829171
C 4.248082 -1.51505 2.713525
H 4.997318 -1.653311 0.695335
H 3.658401 -2.733228 1.030522
H 4.657102 -0.521446 2.9228
H 4.94562 -2.250901 3.119891
H 3.307222 -1.612368 3.264475
N 1.728269 1.850389 -1.861969
H 2.020142 0.861405 -1.692331
H 2.45753 2.342821 -2.376228
H 2.249262 2.416495 0.089587
H 0.868409 1.811714 -2.421971
C 0.228729 1.681531 -0.012492
H 0.629841 0.677322 0.187394
O -0.703866 1.610734 -1.074146
H -1.508419 1.196435 -0.733884
C 0.60821 -2.041543 -0.850917
H 0.820127 -3.09331 -0.608244
H 0.393014 -1.562518 0.110194
C -0.614282 -2.005351 -1.7683
H -0.329205 -2.44045 -2.733467
H -0.894652 -0.965493 -1.976607
C -2.719504 1.221511 1.47209
H -3.132386 2.111342 0.99073
C -1.409988 1.171821 1.718577
H -1.002402 0.289031 2.212084

conf_02

1 1
C 0.246867 -2.598379 1.758034
C -0.196156 -1.480024 0.838194
H 1.271023 -2.885561 1.52055
H -0.391898 -3.478439 1.654183
H 0.243434 -2.271328 2.802148
C 0.892702 -2.474894 -1.239366
H 0.639306 -2.805869 -2.254847

H 1.187458 -3.368545 -0.682124
C 4.216124 -0.514658 -0.595915
C 4.323319 0.257203 0.72788
H 4.054346 0.176999 -1.428999
H 5.171554 -1.015499 -0.788214
C 3.094024 1.105945 1.039063
H 5.213145 0.895561 0.694635
H 4.492412 -0.457135 1.542206
C 2.948655 2.350419 0.164014
H 3.116884 1.41399 2.090417
H 2.203981 0.473299 0.928203
C 1.526668 2.907749 0.134356
H 3.256958 2.129786 -0.865369
H 3.642246 3.116954 0.524385
H 1.54064 3.949348 -0.205558
H 1.126142 2.929271 1.156584
C -1.602981 2.229551 0.444819
C -2.866239 1.78354 0.535867
H -1.078916 2.433563 1.379544
C -3.816227 1.514267 -0.591185
H -3.277332 1.66491 1.539646
C -4.216051 0.041175 -0.741205
H -3.401701 1.872879 -1.535856
H -4.724425 2.104153 -0.413444
C -4.944529 -0.539454 0.463888
H -3.3237 -0.556506 -0.976143
H -4.861734 -0.050421 -1.620084
H -4.355119 -0.485142 1.38721
H -5.212051 -1.58667 0.300676
H -5.871193 0.007608 0.659309
N -1.545668 -0.985585 1.281879
H -1.764104 -0.033897 0.911583
H -1.614142 -0.945299 2.298263
H 0.487557 -0.631981 0.903602
H -2.251985 -1.641786 0.925155
C -0.3708 -1.878715 -0.628025
H -0.639037 -0.970835 -1.192763
O -1.473155 -2.773573 -0.618336
H -1.63208 -3.118986 -1.500128
C -0.849965 2.561987 -0.804448
H -1.357303 2.141943 -1.677071
H -0.877521 3.6528 -0.936713
C 0.612479 2.111973 -0.792383
H 1.006133 2.194082 -1.812077
H 0.665315 1.044185 -0.543677
C 3.124781 -1.53431 -0.573432
H 3.269308 -2.37739 0.105694
C 2.007272 -1.475472 -1.295947
H 1.871648 -0.639785 -1.982647

conf_03

1 1

C 0.848185 0.903244 2.211763
C -0.012069 1.662852 1.226502
H 1.222895 1.591074 2.972517
H 1.703825 0.42731 1.73101
H 0.25933 0.13794 2.723697
C 1.667905 2.666354 -0.501696
H 2.066149 3.657369 -0.759934
H 1.184839 2.299743 -1.416589
C 4.406597 -0.111076 -0.602451
C 4.139697 -1.610703 -0.770431
H 4.749828 0.090154 0.418942
H 5.236753 0.164355 -1.265809
C 2.984543 -2.151434 0.076561
H 5.063329 -2.136995 -0.513858
H 3.953275 -1.835333 -1.828156
C 1.640068 -2.155483 -0.642398
H 2.908884 -1.570203 1.00624
H 3.204291 -3.177807 0.388931
C 0.470123 -2.547822 0.247898
H 1.69355 -2.838843 -1.498957
H 1.457158 -1.165104 -1.089935
H 0.392906 -1.832272 1.078613
H 0.683678 -3.511416 0.725796
C -2.301184 -1.670923 1.329318
C -3.169987 -0.662547 1.159853
H -1.710393 -1.668049 2.245362
C -4.126838 -0.467717 0.025887
H -3.217549 0.094064 1.945325
C -3.707927 0.639426 -0.948265
H -5.104585 -0.204295 0.446245
H -4.269343 -1.397478 -0.531809
C -4.787068 0.958422 -1.971565
H -2.798891 0.317553 -1.477835
H -3.455891 1.550024 -0.386706
H -5.048924 0.071654 -2.555897
H -4.460841 1.734107 -2.668459
H -5.69701 1.313023 -1.479629
N -0.489162 0.753127 0.135592
H -0.973127 1.31624 -0.571029
H -1.166633 0.045573 0.49099
H -0.923746 2.008868 1.721545
H 0.297878 0.262674 -0.29983
C 0.587725 2.900378 0.562401
H 0.98554 3.526037 1.371791
O -0.544895 3.513955 -0.03676
H -0.281832 4.311969 -0.503056
C -2.079122 -2.828371 0.401885
H -1.949719 -3.737933 0.998971
H -2.960656 -2.989558 -0.223323
C -0.853528 -2.64722 -0.502119
H -1.005042 -1.754497 -1.128588
H -0.802337 -3.484172 -1.206459
C 3.244672 0.773646 -0.913868
H 2.764683 0.617543 -1.882892

C 2.794684 1.754675 -0.130369
H 3.302178 1.948941 0.814282

conf_04

1 1
C 0.246867 -2.598379 1.758034
C -0.196156 -1.480024 0.838194
H 1.271023 -2.885561 1.52055
H -0.391898 -3.478439 1.654183
H 0.243434 -2.271328 2.802148
C 0.892702 -2.474894 -1.239366
H 0.639306 -2.805869 -2.254847
H 1.187458 -3.368545 -0.682124
C 4.216124 -0.514658 -0.595915
C 4.323319 0.257203 0.72788
H 4.054346 0.176999 -1.428999
H 5.171554 -1.015499 -0.788214
C 3.094024 1.105945 1.039063
H 5.213145 0.895561 0.694635
H 4.492412 -0.457135 1.542206
C 2.948655 2.350419 0.164014
H 3.116884 1.41399 2.090417
H 2.203981 0.473299 0.928203
C 1.526668 2.907749 0.134356
H 3.256958 2.129786 -0.865369
H 3.642246 3.116954 0.524385
H 1.54064 3.949348 -0.205558
H 1.126142 2.929271 1.156584
C -1.602981 2.229551 0.444819
C -2.866239 1.78354 0.535867
H -1.078916 2.433563 1.379544
C -3.816227 1.514267 -0.591185
H -3.277332 1.66491 1.539646
C -4.216051 0.041175 -0.741205
H -3.401701 1.872879 -1.535856
H -4.724425 2.104153 -0.413444
C -4.944529 -0.539454 0.463888
H -3.3237 -0.556506 -0.976143
H -4.861734 -0.050421 -1.620084
H -4.355119 -0.485142 1.38721
H -5.212051 -1.58667 0.300676
H -5.871193 0.007608 0.659309
N -1.545668 -0.985585 1.281879
H -1.764104 -0.033897 0.911583
H -1.614142 -0.945299 2.298263
H 0.487557 -0.631981 0.903602
H -2.251985 -1.641786 0.925155
C -0.3708 -1.878715 -0.628025
H -0.639037 -0.970835 -1.192763
O -1.473155 -2.773573 -0.618336
H -1.63208 -3.118986 -1.500128
C -0.849965 2.561987 -0.804448

H -1.357303 2.141943 -1.677071
H -0.877521 3.6528 -0.936713
C 0.612479 2.111973 -0.792383
H 1.006133 2.194082 -1.812077
H 0.665315 1.044185 -0.543677
C 3.124781 -1.53431 -0.573432
H 3.269308 -2.37739 0.105694
C 2.007272 -1.475472 -1.295947
H 1.871648 -0.639785 -1.982647

conf_05

1 1
C 0.293137 -2.298311 -1.344718
C 0.586979 -2.40743 0.133975
H 0.461577 -3.265097 -1.82427
H 0.937667 -1.562024 -1.83066
H -0.747099 -2.018771 -1.518498
C 3.136178 -2.284526 -0.282269
H 3.015904 -2.498628 -1.348954
H 4.052098 -2.809796 0.02004
C 3.662845 1.548799 -0.88637
C 2.520527 2.52484 -1.184196
H 4.488682 1.74899 -1.579695
H 4.05205 1.732788 0.121464
C 1.480059 2.566271 -0.076729
H 2.046371 2.265845 -2.139744
H 2.939289 3.527894 -1.315833
C 0.313581 3.50688 -0.344754
H 1.970345 2.849875 0.864577
H 1.09137 1.550751 0.086009
C -0.588709 3.718813 0.871112
H -0.267323 3.135253 -1.198274
H 0.705574 4.480575 -0.659061
H -1.432143 4.358338 0.586393
H -0.029423 4.27676 1.630801
C -2.41364 0.299771 1.209966
C -2.879023 -0.779059 0.562633
H -2.363879 0.253664 2.298928
C -3.151955 -0.862412 -0.908084
H -3.172137 -1.638071 1.164471
C -4.592683 -0.454202 -1.25687
H -2.993724 -1.89021 -1.257475
H -2.463385 -0.215887 -1.464521
C -5.648462 -1.345403 -0.620515
H -4.696759 -0.476389 -2.346684
H -4.748478 0.586088 -0.950726
H -5.505342 -2.393624 -0.90315
H -6.650156 -1.05216 -0.942714
H -5.626278 -1.285007 0.471412
N 0.421151 -1.076196 0.80359
H 0.759107 -1.169683 1.767907
H -0.573692 -0.768275 0.813942

H -0.141607 -3.059821 0.62341
H 1.01282 -0.373243 0.343081
C 1.981335 -2.932484 0.482756
H 1.970488 -4.00469 0.248167
O 2.077506 -2.736358 1.885704
H 2.865848 -3.158871 2.234961
C -2.011728 1.607933 0.59227
H -1.520808 1.437917 -0.374849
H -2.913458 2.187147 0.352668
C -1.120781 2.439846 1.509689
H -0.278554 1.828603 1.863797
H -1.688662 2.698106 2.410356
C 3.298332 0.107115 -1.034038
H 3.042681 -0.213788 -2.046809
C 3.335895 -0.812848 -0.065691
H 3.621616 -0.501865 0.939767

conf_06

1 1
C -1.072153 -1.362295 2.334851
C -0.43378 -2.063143 1.156985
H -1.371609 -2.098254 3.084102
H -1.959469 -0.798437 2.037415
H -0.366564 -0.675646 2.808741
C -2.716479 -2.717616 0.125708
H -3.270861 -2.513792 1.047374
H -3.203461 -3.591281 -0.327774
C -3.586877 0.745049 -1.482266
C -3.181616 2.079633 -0.854038
H -4.634373 0.812487 -1.801456
H -2.998689 0.556957 -2.388315
C -1.740951 2.116111 -0.360532
H -3.853851 2.310326 -0.01831
H -3.331764 2.874964 -1.591823
C -1.32801 3.494201 0.147163
H -1.080005 1.792399 -1.176317
H -1.622175 1.393199 0.462278
C -0.01307 3.572777 0.921971
H -2.127014 3.859088 0.803923
H -1.295449 4.193626 -0.697446
H -0.031766 2.837469 1.738636
H 0.014294 4.548237 1.417817
C 2.061082 1.173883 0.935699
C 2.75573 0.027033 0.921273
H 1.756241 1.561722 1.906817
C 3.325586 -0.645692 -0.287719
H 2.986031 -0.440143 1.879061
C 4.856702 -0.574736 -0.315966
H 2.928109 -0.201483 -1.2077
H 3.035946 -1.707334 -0.294391
C 5.448664 -1.293382 -1.517802
H 5.251478 -1.005274 0.611304

H 5.158943 0.478167 -0.316999
H 5.182386 -2.355098 -1.517076
H 6.538941 -1.226207 -1.517894
H 5.090624 -0.859504 -2.45672
N -0.043325 -1.064827 0.110245
H 0.27454 -1.582631 -0.715147
H 0.721357 -0.444118 0.451537
H 0.504991 -2.536922 1.45785
H -0.862297 -0.508532 -0.165254
C -1.289026 -3.138531 0.483928
H -1.338005 -3.979489 1.187431
O -0.530748 -3.49769 -0.662477
H -0.916878 -4.261765 -1.097413
C 1.712483 2.017641 -0.260861
H 2.58218 2.075832 -0.924229
H 0.929552 1.535334 -0.862095
C 1.276518 3.436799 0.109179
H 1.181374 4.02599 -0.809828
H 2.096016 3.896299 0.673243
C -3.451445 -0.409802 -0.544925
H -3.944754 -0.299847 0.423796
C -2.810638 -1.550493 -0.810175
H -2.351068 -1.675649 -1.791873

conf_07

1 1
C 0.999478 -2.330718 -2.195023
C 0.758664 -2.21914 -0.706896
H 1.361025 -3.333271 -2.433522
H 1.743253 -1.612151 -2.547039
H 0.074302 -2.174161 -2.755195
C 3.292501 -1.861235 -0.211609
H 3.673425 -2.379066 -1.098101
H 4.005798 -2.08927 0.588755
C 3.483576 2.039287 0.163845
C 2.357914 2.825494 0.847838
H 3.424864 2.193695 -0.920072
H 4.450603 2.442076 0.486814
C 0.991221 2.249537 0.512587
H 2.416884 3.869978 0.524831
H 2.502543 2.828916 1.934359
C -0.216561 3.086866 0.904298
H 0.904817 1.279348 1.01991
H 0.958271 2.054748 -0.571192
C -1.526001 2.442539 0.450012
H -0.13099 4.091463 0.472515
H -0.223851 3.22024 1.991504
H -2.366513 2.872961 1.004861
H -1.514332 1.37574 0.721084
C -2.504723 0.289146 -1.820781
C -2.802932 -0.821433 -1.131793
H -1.910186 0.169646 -2.729211

C -3.611945 -0.934656 0.119876
H -2.451067 -1.773672 -1.538502
C -2.816953 -1.515281 1.292192
H -4.467874 -1.59264 -0.078133
H -4.026458 0.035091 0.407985
C -3.677643 -1.766876 2.520398
H -2.008981 -0.818662 1.557298
H -2.33809 -2.453885 0.981049
H -4.148996 -0.84214 2.865768
H -3.086692 -2.169247 3.346612
H -4.474228 -2.482938 2.299357
N 0.273022 -0.852955 -0.339455
H 0.196793 -0.822838 0.682794
H -0.651218 -0.618477 -0.750135
H -0.047647 -2.892526 -0.402751
H 0.964842 -0.137393 -0.588754
C 1.965943 -2.522987 0.185702
H 2.108828 -3.61071 0.154308
O 1.530768 -2.127368 1.477931
H 2.170102 -2.390675 2.14377
C -2.940588 1.694553 -1.542399
H -3.33518 2.103175 -2.479078
H -3.766589 1.716391 -0.825955
C -1.804893 2.597184 -1.042866
H -2.061178 3.642396 -1.243584
H -0.900722 2.395904 -1.632544
C 3.452253 0.572674 0.452024
H 3.602033 0.277802 1.492248
C 3.272008 -0.378857 -0.467669
H 3.167205 -0.062793 -1.507733

conf_08

1 1
C -1.008777 -1.34325 2.258832
C -0.524929 -2.001058 0.986803
H -1.277528 -2.107846 2.990851
H -1.886644 -0.717038 2.083149
H -0.223618 -0.724217 2.699691
C -2.924846 -2.494787 0.140961
H -3.367478 -2.285059 1.119963
H -3.502235 -3.331048 -0.27453
C -3.627791 1.086859 -1.272589
C -2.455006 2.06099 -1.102144
H -4.565258 1.611873 -1.057523
H -3.677248 0.761972 -2.31641
C -2.298618 2.593512 0.318809
H -2.594707 2.904819 -1.786712
H -1.538003 1.561711 -1.440401
C -1.163261 3.59607 0.538767
H -2.170375 1.761509 1.027043
H -3.246979 3.067022 0.599721
C 0.218778 2.993397 0.794745

H -1.420202 4.206315 1.410151
H -1.114025 4.293 -0.3075
H 0.118063 2.205649 1.554761
H 0.853064 3.761583 1.253735
C 2.432081 0.851313 0.876955
C 2.794933 -0.422149 0.661479
H 2.189173 1.12816 1.902203
C 3.233605 -1.045894 -0.627757
H 2.842533 -1.079232 1.532002
C 4.739019 -1.349126 -0.640021
H 2.988187 -0.40482 -1.481593
H 2.693795 -1.992562 -0.772247
C 5.59924 -0.095569 -0.616667
H 4.963403 -1.939163 -1.534345
H 4.982219 -1.987112 0.217833
H 5.404659 0.533269 -1.491562
H 6.661322 -0.350781 -0.625448
H 5.410464 0.505602 0.277516
N -0.179585 -0.960548 -0.035461
H 0.013968 -1.443374 -0.919331
H 0.662413 -0.422134 0.253957
H 0.412312 -2.535135 1.167361
H -0.982894 -0.340051 -0.192826
C -1.491988 -2.995855 0.341333
H -1.518034 -3.870762 1.00356
O -0.858171 -3.324926 -0.886572
H -1.321888 -4.042096 -1.325645
C 2.376574 1.961177 -0.127732
H 2.96305 2.799703 0.268321
H 2.862869 1.661858 -1.059128
C 0.957292 2.458897 -0.428064
H 0.39164 1.649952 -0.906831
H 1.018584 3.251239 -1.182363
C -3.516977 -0.108871 -0.37861
H -3.830462 0.019834 0.65819
C -3.055584 -1.302688 -0.758766
H -2.774146 -1.447198 -1.80281

conf_09

1 1
C -0.86621 -1.490299 1.851067
C -0.949485 -2.080807 0.462164
H -1.112305 -2.255094 2.590878
H -1.559491 -0.656199 1.979627
H 0.144948 -1.13542 2.064563
C -3.532849 -1.852318 0.343896
H -3.634339 -1.68486 1.420932
H -4.405759 -2.448782 0.048507
C -3.408505 1.957578 -0.529015
C -1.980445 2.520548 -0.49124
H -4.092191 2.684323 -0.076241
H -3.722359 1.828109 -1.569439

C -1.458988 2.784763 0.915742
H -1.946819 3.449894 -1.070683
H -1.31432 1.825276 -1.022965
C -0.027284 3.306177 0.996699
H -1.522201 1.868951 1.519794
H -2.129491 3.504373 1.400675
C 1.060959 2.303003 0.590483
H 0.151393 3.617688 2.030218
H 0.06958 4.216383 0.392009
H 0.710975 1.279975 0.797297
H 1.930776 2.440972 1.242937
C 2.32022 0.040687 -1.362364
C 2.530768 -0.990507 -0.53088
H 1.863749 -0.180321 -2.329892
C 3.196278 -0.960679 0.808757
H 2.266802 -1.988039 -0.892657
C 4.584945 -1.614079 0.780431
H 2.572153 -1.504624 1.530223
H 3.279312 0.065612 1.178653
C 5.591306 -0.840449 -0.0571
H 4.49322 -2.639505 0.402367
H 4.945595 -1.69925 1.810518
H 5.265439 -0.751269 -1.097657
H 6.564619 -1.33644 -0.056678
H 5.737157 0.17065 0.336622
N -0.635819 -1.039834 -0.570915
H -0.839179 -1.448036 -1.489959
H 0.358954 -0.737019 -0.544694
H -0.183649 -2.850025 0.32855
H -1.261284 -0.233056 -0.450436
C -2.292822 -2.715892 0.088991
H -2.375991 -3.632186 0.687269
O -2.135984 -3.036421 -1.285913
H -2.87794 -3.559326 -1.599354
C 2.715719 1.471228 -1.164151
H 3.470881 1.555642 -0.377459
H 3.199466 1.802654 -2.08963
C 1.552425 2.418999 -0.849167
H 0.735533 2.259011 -1.564102
H 1.891549 3.44727 -1.013997
C -3.509477 0.65326 0.196309
H -3.527568 0.696189 1.285702
C -3.53506 -0.545533 -0.390262
H -3.557595 -0.597389 -1.479927

conf_10

1 1
C -0.439887 -1.135312 2.324296
C 0.326274 -1.740631 1.170244
H -0.489207 -1.850203 3.148462
H -1.461205 -0.873029 2.03889
H 0.058225 -0.236955 2.696113

C -1.702788 -3.15307 0.382027
H -2.245197 -3.014717 1.322816
H -1.922086 -4.179381 0.059025
C -3.562807 -0.224533 -1.42402
C -2.875036 1.135777 -1.251056
H -4.646975 -0.095666 -1.3291
H -3.373334 -0.6013 -2.433897
C -3.104516 1.771494 0.115962
H -3.232054 1.818355 -2.030153
H -1.80315 1.005615 -1.447672
C -2.413425 3.113571 0.357616
H -2.797512 1.079668 0.912925
H -4.185689 1.90245 0.244526
C -0.926616 3.043876 0.715836
H -2.930391 3.606176 1.187041
H -2.554888 3.76876 -0.511344
H -0.781047 2.234113 1.444959
H -0.655166 3.964516 1.24649
C 1.982764 1.900435 0.865104
C 3.009825 1.064017 0.652263
H 1.484828 1.847116 1.83366
C 3.913378 1.037531 -0.545474
H 3.279379 0.380953 1.460506
C 4.601699 -0.304912 -0.778459
H 4.683277 1.807252 -0.401803
H 3.366496 1.33269 -1.448507
C 3.639288 -1.440875 -1.089218
H 5.202852 -0.562522 0.10147
H 5.309338 -0.192912 -1.605167
H 3.005692 -1.680743 -0.228589
H 4.171378 -2.359748 -1.345544
H 2.995022 -1.191534 -1.941593
N 0.371409 -0.786788 0.013443
H 0.778339 -1.286473 -0.785406
H 0.963716 0.041136 0.227416
H 1.373556 -1.890607 1.447057
H -0.581077 -0.502268 -0.24389
C -0.199341 -3.085476 0.660007
H 0.043714 -3.820866 1.437987
O 0.574491 -3.326915 -0.505454
H 0.409134 -4.210334 -0.843512
C 1.517399 2.986852 -0.054939
H 1.679701 3.947082 0.453126
H 2.137325 3.013026 -0.954288
C 0.041454 2.894178 -0.454248
H -0.123911 1.952139 -0.991479
H -0.168253 3.682697 -1.185508
C -3.092229 -1.223801 -0.415325
H -3.507704 -1.140476 0.589548
C -2.19487 -2.183812 -0.64968
H -1.802307 -2.303039 -1.660664

6E-3-Keto-1-deoxySO

conf_00

1 1
C 0.165107 -2.157601 1.935729
C 0.696723 -1.236885 0.849169
H 0.991555 -2.644823 2.454267
H -0.477016 -2.937156 1.516263
H -0.401481 -1.591488 2.678956
C 2.821049 -2.571172 0.133534
C 3.943179 -1.702709 -0.4804
H 2.866468 -3.580872 -0.283856
H 2.942927 -2.633787 1.218222
C 3.976514 -0.323784 0.101302
H 3.797183 -1.658589 -1.563056
H 4.891593 -2.217073 -0.29859
C 3.592411 0.776722 -0.545371
H 4.340467 -0.235085 1.12677
C 3.582909 2.151801 0.050234
H 3.256215 0.676265 -1.578134
C 2.212218 2.550823 0.60736
H 3.892465 2.880846 -0.70688
H 4.319211 2.205373 0.857832
C 1.086222 2.544977 -0.417914
H 2.283421 3.546457 1.058706
H 1.961552 1.867126 1.431963
C -0.283443 2.797058 0.201973
H 1.085693 1.587593 -0.963744
H 1.280947 3.300332 -1.188282
C -1.438747 2.740232 -0.793574
H -0.283954 3.776148 0.694835
H -0.447851 2.09021 1.034639
C -2.819536 2.827496 -0.144305
H -1.368464 1.830511 -1.409968
H -1.321048 3.563791 -1.505395
C -3.236567 1.587868 0.646562
H -3.570349 3.017481 -0.919204
H -2.840891 3.701382 0.516383
C -3.566982 0.377465 -0.2212
H -4.115302 1.828889 1.254081
H -2.456615 1.32679 1.378839
C -3.861819 -0.883237 0.585652
H -2.763513 0.194388 -0.948349
H -4.443105 0.609375 -0.838076
C -4.175494 -2.119916 -0.251539
H -4.701487 -0.674847 1.257821
H -3.025318 -1.112441 1.274976
C -2.994614 -2.651401 -1.053287
H -5.001884 -1.885503 -0.931928
H -4.543954 -2.911667 0.408371
H -2.616816 -1.919849 -1.775676
H -3.27131 -3.539612 -1.625408
H -2.16896 -2.952235 -0.394545
N -0.419837 -0.575304 0.108919

H -0.328564 0.443513 0.078114
H -1.346841 -0.80163 0.47948
H 1.34125 -0.459078 1.268369
H -0.34201 -0.955676 -0.861418
C 1.498409 -1.979184 -0.229163
O 1.042427 -2.009905 -1.350924

conf_01

1 1
C -0.022965 -2.528931 1.471628
C 0.668574 -1.422425 0.690462
H 0.716837 -3.218022 1.881389
H -0.69898 -3.100933 0.829579
H -0.589914 -2.11694 2.309827
C 2.775395 -2.671976 -0.194111
C 3.938194 -1.740442 -0.606917
H 2.811748 -3.592056 -0.783617
H 2.853165 -2.929162 0.86613
C 3.952392 -0.466258 0.179784
H 3.849513 -1.52744 -1.675592
H 4.870909 -2.29282 -0.458743
C 3.613612 0.725411 -0.312851
H 4.261926 -0.538927 1.224194
C 3.589412 1.997562 0.476992
H 3.334116 0.78604 -1.365439
C 2.181119 2.395283 0.931532
H 4.015113 2.808424 -0.125167
H 4.228334 1.892229 1.359031
C 1.185084 2.613174 -0.200328
H 2.243347 3.306331 1.536595
H 1.80329 1.61526 1.610629
C -0.229962 2.89114 0.295813
H 1.18315 1.740211 -0.873145
H 1.518614 3.443555 -0.83343
C -1.270813 2.970851 -0.815845
H -0.23713 3.824173 0.871235
H -0.517912 2.12737 1.039132
C -2.712254 3.07614 -0.317998
H -1.166595 2.105458 -1.48669
H -1.045034 3.839348 -1.443492
C -3.229575 1.84087 0.418429
H -3.368962 3.282654 -1.170488
H -2.794728 3.945895 0.343451
C -3.391989 0.607152 -0.464014
H -4.19648 2.075491 0.875849
H -2.568018 1.606625 1.265308
C -3.670875 -0.670082 0.320264
H -2.503852 0.46917 -1.098658
H -4.207548 0.772939 -1.176867
C -3.678295 -1.936553 -0.526555
H -4.630767 -0.572887 0.840955
H -2.945919 -0.780673 1.151465

C -3.97678 -3.193002 0.277184
H -2.714127 -2.046592 -1.047135
H -4.419626 -1.823308 -1.325138
H -3.249004 -3.33968 1.0834
H -3.960761 -4.085228 -0.352967
H -4.964846 -3.134603 0.742121
N -0.332188 -0.510132 0.060958
H -0.092936 0.47976 0.168889
H -1.287811 -0.647931 0.403441
H 1.327663 -0.83021 1.329582
H -0.286586 -0.746165 -0.954987
C 1.486191 -1.976406 -0.483792
O 1.064544 -1.786936 -1.603177

conf_02

1 1
C 0.88874 -2.584369 1.500985
C 1.27228 -1.339525 0.716877
H 1.752944 -2.954227 2.05562
H 0.549374 -3.381034 0.833353
H 0.101051 -2.363434 2.225312
C 3.797653 -1.681675 0.203078
C 4.494153 -0.381154 -0.263364
H 4.303559 -2.548588 -0.229126
H 3.826222 -1.761377 1.294029
C 3.790188 0.846051 0.231324
H 4.525536 -0.381895 -1.356106
H 5.526797 -0.407709 0.096165
C 3.029055 1.628224 -0.534566
H 3.907249 1.090847 1.28864
C 2.247097 2.809777 -0.057645
H 2.937629 1.384655 -1.595122
C 0.754725 2.484414 0.033417
H 2.386462 3.649345 -0.748031
H 2.609528 3.138465 0.921699
C -0.148129 3.667546 0.357399
H 0.623621 1.733794 0.832473
H 0.436946 2.03474 -0.920179
C -1.590983 3.255468 0.634215
H -0.112582 4.387485 -0.46898
H 0.255153 4.185527 1.234723
C -2.343125 2.719264 -0.581315
H -2.141945 4.115359 1.030106
H -1.591772 2.509361 1.441217
C -3.704804 2.118732 -0.233691
H -1.740415 1.969448 -1.11189
H -2.47736 3.537036 -1.297979
C -3.633974 0.754153 0.451209
H -4.307774 2.02479 -1.144057
H -4.243425 2.820223 0.413163
C -3.259208 -0.387859 -0.486988
H -4.604991 0.524356 0.903156

H -2.924873 0.793061 1.290391
C -3.090777 -1.72535 0.223557
H -2.346768 -0.135506 -1.050064
H -4.033978 -0.493109 -1.255453
C -2.729603 -2.888811 -0.692687
H -4.019177 -1.965593 0.755233
H -2.350134 -1.638221 1.043491
C -2.59332 -4.212961 0.043685
H -1.80208 -2.670835 -1.244592
H -3.499753 -2.975349 -1.466772
H -1.829322 -4.164603 0.827747
H -2.322737 -5.024122 -0.636132
H -3.534001 -4.487983 0.528703
N 0.130053 -0.855464 -0.113306
H 0.138134 0.168329 -0.189552
H -0.788726 -1.153772 0.228964
H 1.584919 -0.528058 1.376968
H 0.304733 -1.246977 -1.060393
C 2.388022 -1.645492 -0.290335
O 2.072956 -1.801847 -1.448064

conf_03

1 1
C 0.000751 -2.523711 1.460035
C 0.81856 -1.571477 0.602089
H 0.643598 -2.995315 2.20543
H -0.449201 -3.315214 0.854096
H -0.787571 -1.988089 1.993878
C 3.205395 -2.599989 0.641116
C 4.27837 -1.585997 0.177376
H 3.532625 -3.616902 0.411411
H 3.038108 -2.514242 1.719003
C 3.861739 -0.165956 0.414106
H 4.469106 -1.751267 -0.886329
H 5.203519 -1.810208 0.716087
C 3.455417 0.665937 -0.545767
H 3.894822 0.191918 1.444759
C 2.996633 2.072044 -0.332621
H 3.449167 0.305492 -1.576819
C 1.486996 2.227245 -0.52597
H 3.51184 2.733366 -1.03928
H 3.269559 2.410438 0.67233
C 0.998776 3.671688 -0.509417
H 0.978242 1.669537 0.277556
H 1.194234 1.775261 -1.488075
C -0.523816 3.774162 -0.500432
H 1.40872 4.188188 -1.383778
H 1.408031 4.184245 0.370096
C -1.150394 3.455232 0.863441
H -0.923258 3.102055 -1.27221
H -0.827312 4.778897 -0.808456
C -2.497642 2.743275 0.779999

H -1.257008 4.383352 1.432715
H -0.466783 2.829398 1.454801
C -2.354532 1.290429 0.34634
H -3.157166 3.27196 0.080851
H -2.995896 2.773853 1.755142
C -3.659775 0.51924 0.220627
H -1.705028 0.783588 1.080515
H -1.832125 1.260453 -0.622646
C -3.447519 -0.930345 -0.193601
H -4.307849 1.01433 -0.511684
H -4.197147 0.54916 1.175715
C -4.723772 -1.751343 -0.326005
H -2.802219 -1.428352 0.552026
H -2.921229 -0.958235 -1.163425
C -4.459494 -3.195097 -0.723818
H -5.375996 -1.273784 -1.065742
H -5.266083 -1.719345 0.625761
H -3.946382 -3.254743 -1.68957
H -5.389563 -3.760654 -0.813245
H -3.837871 -3.705294 0.020337
N -0.010276 -0.985461 -0.493705
H 0.258182 -0.013455 -0.678745
H -1.017645 -1.010877 -0.307136
H 1.232796 -0.752673 1.193436
H 0.223588 -1.554703 -1.33306
C 1.953816 -2.30865 -0.118839
O 1.797759 -2.575504 -1.288972

conf_04

1 1
C 1.53183 2.977361 -0.519522
C 1.669851 1.499069 -0.188787
H 2.428598 3.323412 -1.036658
H 1.416667 3.5753 0.388919
H 0.678815 3.156884 -1.176809
C 4.233468 1.171224 0.05446
C 4.643525 -0.319404 0.01684
H 4.954549 1.739765 0.646814
H 4.201228 1.584457 -0.958302
C 3.639567 -1.170059 -0.701038
H 4.761835 -0.668416 1.046073
H 5.622337 -0.384507 -0.467117
C 2.797217 -2.000158 -0.085222
H 3.607933 -1.089917 -1.789113
C 1.750852 -2.836603 -0.745643
H 2.859699 -2.085283 1.001965
C 0.339329 -2.34671 -0.413981
H 1.84897 -3.872875 -0.400442
H 1.895919 -2.850106 -1.830633
C -0.769033 -3.317918 -0.804187
H 0.167494 -1.393986 -0.94646
H 0.28242 -2.147748 0.666159

```
C -2.173219 -2.753907 -0.608629
H -0.652216 -4.237465 -0.217688
H -0.63482 -3.604048 -1.853548
C -2.504225 -2.407181 0.838505
H -2.905566 -3.480938 -0.97724
H -2.289495 -1.867216 -1.246505
C -3.892943 -1.808423 1.046725
H -1.758578 -1.707138 1.238575
H -2.410425 -3.311036 1.452211
C -4.169251 -0.535362 0.246891
H -4.02137 -1.595073 2.114551
H -4.65407 -2.555216 0.794479
C -3.083853 0.527355 0.367983
H -5.123599 -0.111119 0.578364
H -4.304396 -0.777061 -0.814181
C -3.456354 1.858224 -0.269511
H -2.171497 0.144693 -0.116862
H -2.837991 0.684251 1.429202
C -2.284092 2.825828 -0.354115
H -4.270902 2.321151 0.299302
H -3.849011 1.685784 -1.279034
C -2.646997 4.199663 -0.897571
H -1.517194 2.378649 -1.009044
H -1.84432 2.94679 0.650871
H -3.076196 4.119639 -1.900077
H -1.776937 4.85936 -0.956978
H -3.387921 4.68542 -0.257252
N 0.50463 1.014579 0.611669
H 0.320202 0.021216 0.429522
H -0.356405 1.543003 0.437178
H 1.742604 0.88808 -1.090491
H 0.803208 1.099908 1.602588
C 2.891682 1.258961 0.705358
O 2.701736 1.118447 1.89183
```

conf_05

```
1 1
C 0.798166 -2.357637 1.845081
C 1.198703 -1.241133 0.89353
H 1.663061 -2.672447 2.431598
H 0.425164 -3.228575 1.299692
H 0.03079 -2.017673 2.544798
C 3.701291 -1.740318 0.392838
C 4.447318 -0.537608 -0.232239
H 4.162313 -2.674617 0.062587
H 3.744723 -1.690875 1.485097
C 3.80749 0.76802 0.129646
H 4.456441 -0.664881 -1.317978
H 5.484686 -0.572068 0.112858
C 3.069866 1.495696 -0.709138
H 3.945972 1.118128 1.154373
C 2.326992 2.741234 -0.34398
```

H 2.954621 1.14578 -1.736912
C 0.838395 2.442367 -0.154832
H 2.445991 3.494769 -1.130243
H 2.731619 3.172195 0.577473
C -0.050724 3.649582 0.107965
H 0.746524 1.766116 0.712472
H 0.476612 1.911342 -1.049284
C -1.464365 3.256014 0.526485
H -0.082497 4.283151 -0.786318
H 0.404224 4.255546 0.899376
C -2.291053 2.577879 -0.563337
H -2.003951 4.149038 0.859565
H -1.399788 2.605446 1.410693
C -3.616393 2.021702 -0.043185
H -1.722062 1.768002 -1.042356
H -2.480612 3.30399 -1.361405
C -3.489166 0.686902 0.691931
H -4.318742 1.895834 -0.87467
H -4.069522 2.764111 0.622956
C -3.352137 -0.512466 -0.240899
H -4.371795 0.530846 1.321576
H -2.639515 0.724682 1.391461
C -3.24726 -1.846607 0.492551
H -2.506471 -0.366774 -0.929616
H -4.231856 -0.551465 -0.894581
C -3.242574 -3.076919 -0.411033
H -4.091265 -1.918736 1.187555
H -2.36218 -1.869664 1.157938
C -2.011912 -3.219043 -1.29691
H -4.138782 -3.046401 -1.040852
H -3.341503 -3.974201 0.208266
H -1.87275 -2.355056 -1.956268
H -2.090547 -4.095426 -1.943865
H -1.10196 -3.359996 -0.699376
N 0.052095 -0.830297 0.030526
H 0.080399 0.177357 -0.162715
H -0.865694 -1.069924 0.416231
H 1.550139 -0.361479 1.436688
H 0.192788 -1.336119 -0.866613
C 2.285463 -1.704703 -0.085303
O 1.947771 -1.980708 -1.213614

conf_06

1 1
C 0.801538 -2.488898 1.556809
C 1.209177 -1.334803 0.654844
H 1.628336 -2.748486 2.220106
H 0.544239 -3.376253 0.972605
H -0.049924 -2.213533 2.183103
C 3.779779 -1.645342 0.440852
C 4.472858 -0.342472 -0.024833
H 4.360305 -2.51154 0.114484

H 3.696542 -1.662749 1.531615
C 3.690824 0.881107 0.344586
H 4.606596 -0.392196 -1.108757
H 5.468057 -0.315716 0.428376
C 2.98906 1.609616 -0.523954
H 3.700463 1.17412 1.396024
C 2.159584 2.804487 -0.184363
H 3.006879 1.319876 -1.576996
C 0.663175 2.533444 -0.352117
H 2.439258 3.637131 -0.841002
H 2.362562 3.12974 0.84127
C -0.215004 3.773831 -0.235813
H 0.355806 1.803857 0.41568
H 0.486709 2.075944 -1.338777
C -1.702943 3.449781 -0.328165
H 0.065738 4.470895 -1.032729
H -0.002277 4.286753 0.710444
C -2.284391 2.814309 0.940338
H -1.86002 2.78692 -1.190238
H -2.264184 4.360287 -0.558237
C -3.412454 1.824697 0.660436
H -2.637326 3.60635 1.607485
H -1.495304 2.292434 1.500844
C -2.887743 0.499063 0.123237
H -4.115839 2.260664 -0.059733
H -3.986654 1.635744 1.573813
C -3.960375 -0.476283 -0.342672
H -2.278873 0.0419 0.919478
H -2.215797 0.701338 -0.726174
C -3.396915 -1.794775 -0.866362
H -4.547284 0.004349 -1.13253
H -4.660471 -0.675583 0.477919
C -2.85138 -2.723149 0.215512
H -2.61606 -1.583195 -1.615645
H -4.173377 -2.336206 -1.416821
C -2.165387 -3.96235 -0.341119
H -3.677103 -3.023886 0.869693
H -2.169803 -2.181422 0.893417
H -2.857352 -4.542681 -0.957133
H -1.804747 -4.620322 0.45389
H -1.31332 -3.709967 -0.985504
N 0.136242 -1.013041 -0.334632
H 0.061124 -0.002887 -0.489977
H -0.787011 -1.366484 -0.068325
H 1.425762 -0.432628 1.230315
H 0.456319 -1.457331 -1.220573
C 2.431221 -1.697189 -0.197788
O 2.246325 -1.9618 -1.364444

conf_07

1 1
C 2.195711 0.374728 2.341283

C 1.424764 -0.492929 1.366135
H 2.977447 0.915428 1.807584
H 2.665514 -0.219815 3.131012
H 1.5396 1.121097 2.796356
C 3.509569 -1.353378 0.031718
C 3.308931 -0.560524 -1.283365
H 3.973023 -2.318027 -0.180866
H 4.177653 -0.792437 0.694629
C 2.994594 0.888312 -1.093549
H 2.539194 -1.045593 -1.891688
H 4.248722 -0.65788 -1.837348
C 1.882271 1.4882 -1.518291
H 3.758823 1.4877 -0.594766
C 1.558692 2.935748 -1.320811
H 1.143222 0.884825 -2.048127
C 0.536235 3.169907 -0.201627
H 1.165347 3.34797 -2.257433
H 2.474764 3.489199 -1.092784
C -0.779829 2.431142 -0.396704
H 0.343695 4.244945 -0.114866
H 0.985749 2.865985 0.754353
C -1.779264 2.648662 0.73463
H -0.577202 1.35709 -0.509193
H -1.237516 2.736938 -1.346011
C -2.971952 1.69711 0.670553
H -2.128587 3.685916 0.708865
H -1.272538 2.531103 1.704514
C -2.61189 0.276429 1.095155
H -3.368658 1.686822 -0.350934
H -3.782904 2.059931 1.310286
C -3.660202 -0.793078 0.8023
H -2.395797 0.289956 2.175581
H -1.687976 0.001262 0.558612
C -3.929536 -1.01369 -0.689071
H -4.597281 -0.525656 1.302541
H -3.343836 -1.738922 1.259305
C -2.677329 -1.185862 -1.546109
H -4.511658 -0.17543 -1.085976
H -4.571042 -1.895322 -0.796329
C -1.765119 -2.334151 -1.130772
H -2.10017 -0.25052 -1.550648
H -2.980788 -1.338499 -2.5878
C -0.467105 -2.368406 -1.923662
H -2.29781 -3.284968 -1.245907
H -1.53784 -2.26381 -0.057236
H -0.664025 -2.484974 -2.993111
H 0.182646 -3.190067 -1.611636
H 0.088858 -1.429843 -1.802305
N 0.254051 -1.157616 2.022315
H 0.305214 -2.14733 1.684815
H -0.642835 -0.728346 1.762171
H 1.045292 0.124973 0.544432
H 0.335635 -1.159663 3.03895
C 2.22688 -1.644818 0.737162

O 1.749648 -2.757642 0.814362

conf_08

1 1
C 2.133469 -1.409683 -2.285495
C 1.669689 -0.193774 -1.505186
H 2.576944 -2.133319 -1.601151
H 2.88034 -1.14876 -3.041398
H 1.28701 -1.902452 -2.770752
C 3.699356 -0.04373 0.141953
C 2.958105 -0.441323 1.441024
H 4.49599 0.668011 0.363476
H 4.154889 -0.936087 -0.301826
C 2.109312 -1.666058 1.323781
H 2.366315 0.404951 1.805903
H 3.739163 -0.615148 2.189302
C 0.807251 -1.720274 1.605854
H 2.622302 -2.581948 1.023779
C -0.04251 -2.945986 1.515759
H 0.314983 -0.805575 1.939188
C -1.108547 -2.863427 0.417409
H -0.542345 -3.102562 2.480053
H 0.592926 -3.820866 1.346008
C -2.084932 -1.707425 0.581928
H -1.663327 -3.808069 0.394145
H -0.609457 -2.784203 -0.559701
C -3.163254 -1.672411 -0.497659
H -1.533806 -0.760349 0.592403
H -2.567074 -1.773933 1.565827
C -4.038152 -0.413463 -0.478241
H -3.792112 -2.561295 -0.381351
H -2.696611 -1.775793 -1.488726
C -3.564829 0.699242 -1.415569
H -4.118972 -0.039511 0.549454
H -5.058717 -0.673049 -0.774346
C -2.13968 1.193893 -1.175834
H -4.248984 1.552356 -1.343197
H -3.641121 0.335735 -2.447463
C -1.919482 1.929573 0.139929
H -1.860585 1.861926 -2.006696
H -1.476249 0.310411 -1.209096
C -0.457456 2.276983 0.389103
H -2.299683 1.320415 0.965292
H -2.518271 2.848081 0.13874
C -0.190412 3.050491 1.677476
H -0.077626 2.868792 -0.456499
H 0.129512 1.344021 0.419191
C -0.504969 2.266126 2.943756
H -0.774699 3.977811 1.661934
H 0.861127 3.357955 1.691573
H -1.563731 2.002646 3.013317
H -0.256983 2.846658 3.835298

H 0.073061 1.335908 2.989738
N 0.961618 0.793119 -2.381332
H 1.497053 1.682587 -2.22879
H -0.017774 0.931176 -2.104583
H 0.982434 -0.506536 -0.711851
H 0.99664 0.538564 -3.367533
C 2.8047 0.614238 -0.853805
O 2.90127 1.783958 -1.16218

conf_09

1 1
C -0.961376 2.18309 2.366917
C -1.424726 1.443505 1.128472
H -1.684016 2.967316 2.591916
H 0.011607 2.656679 2.210096
H -0.898686 1.513687 3.228982
C -1.308057 2.047341 -1.449654
C 0.020834 2.74751 -1.81663
H -2.09609 2.39243 -2.122282
H -1.208692 0.965512 -1.59226
C 1.106128 2.591132 -0.791297
H -0.188347 3.813244 -1.947577
H 0.352856 2.360864 -2.783445
C 2.156556 1.767204 -0.889779
H 1.048668 3.252197 0.076912
C 3.257325 1.661051 0.11787
H 2.23969 1.145587 -1.782718
C 3.365115 0.274769 0.763524
H 3.115467 2.423721 0.890758
H 4.207538 1.894265 -0.379005
C 3.801573 -0.831798 -0.190493
H 2.397843 0.01909 1.219117
H 4.069436 0.327924 1.600555
C 3.633847 -2.241498 0.374168
H 4.853641 -0.673949 -0.451271
H 3.253069 -0.757057 -1.136339
C 2.195833 -2.624926 0.726314
H 4.260554 -2.357228 1.265714
H 4.019452 -2.956795 -0.360797
C 1.205044 -2.427984 -0.413006
H 1.85946 -2.057343 1.606645
H 2.178001 -3.673909 1.041583
C -0.221031 -2.839603 -0.072271
H 1.542168 -2.987503 -1.294408
H 1.21203 -1.373903 -0.732199
C -1.218096 -2.512062 -1.180973
H -0.525477 -2.374707 0.878906
H -0.256111 -3.915585 0.131238
C -2.683115 -2.702361 -0.802639
H -0.976187 -3.125707 -2.055048
H -1.070489 -1.473441 -1.532894
C -3.219515 -1.631091 0.140362

H -2.817384 -3.69044 -0.346154
H -3.295538 -2.702565 -1.710717
C -4.68315 -1.826377 0.507397
H -3.101285 -0.648963 -0.346788
H -2.622576 -1.613689 1.06486
H -5.31221 -1.826719 -0.387004
H -5.046116 -1.03618 1.169733
H -4.831363 -2.782246 1.017253
N -0.408458 0.409261 0.738021
H -0.794597 -0.30114 0.108175
H -0.056609 -0.083407 1.560304
H -2.349498 0.895583 1.336511
H 0.403488 0.861641 0.260693
C -1.74218 2.394639 -0.04539
O -2.340068 3.402833 0.212181

1-DeoxymethylSA

conf_00

1 1
C -1.093126 2.485848 -0.862096
C -2.734876 1.067082 0.39174
H -3.422821 1.919821 0.381903
H -2.115802 1.163913 1.291387
C -1.549826 -1.628947 1.294946
C -0.904584 -3.011937 1.328151
H -1.913964 -1.386112 2.301131
H -0.780478 -0.877192 1.071415
C -0.398791 -3.531275 -0.01732
H -1.632529 -3.730654 1.722225
H -0.08064 -2.996872 2.050084
C 0.620347 -2.644045 -0.729936
H -1.247319 -3.710789 -0.687758
H 0.056733 -4.514438 0.148458
C 1.85526 -2.325319 0.108052
H 0.139611 -1.710591 -1.05919
H 0.931478 -3.152409 -1.651007
H 2.150031 -3.226273 0.659172
H 1.602864 -1.583551 0.8773
C 2.24099 0.579329 -0.870799
C 3.044916 1.098288 0.316278
H 1.220472 0.338411 -0.540365
H 2.180415 1.403877 -1.60664
C 2.322173 2.178576 1.115763
H 4.006442 1.476819 -0.046871
H 3.280918 0.265245 0.98712
C 1.181305 1.646249 1.977805
H 1.946603 2.958734 0.43252
H 3.037996 2.692324 1.766414
C 0.43721 2.736442 2.7346
H 1.591448 0.922761 2.691774
H 0.474747 1.069611 1.364599

H 1.112212 3.280875 3.400821
H -0.368576 2.324791 3.34796
H -0.004732 3.478601 2.058353
N -0.301286 2.505393 -2.134133
H 0.538756 1.918864 -2.038424
H -0.008939 3.442743 -2.409257
H -0.413005 2.631913 -0.024133
H -0.90948 2.101134 -2.861431
C -1.811335 1.153914 -0.807119
H -1.063061 0.349238 -0.745679
O -2.469579 1.083648 -2.06602
H -2.968278 0.265142 -2.13506
C 2.827799 -0.633142 -1.589745
H 2.161454 -0.908661 -2.416275
H 3.779781 -0.349035 -2.05234
C 3.061964 -1.854229 -0.700395
H 3.89394 -1.6559 -0.016137
H 3.399264 -2.675728 -1.34279
C -2.703791 -1.511979 0.305431
H -2.316067 -1.605111 -0.719229
H -3.374373 -2.369235 0.433148
C -3.532857 -0.236177 0.448231
H -4.318209 -0.219723 -0.317734
H -4.066046 -0.256954 1.404099
H -1.811891 3.305404 -0.914

conf_01

1 1
C 0.083764 -1.948107 1.297589
C -1.559801 -2.07008 -0.662574
H -1.680148 -2.578563 -1.627552
H -1.365906 -1.019806 -0.913771
C -3.92391 -0.056549 -0.775912
C -3.901369 0.76446 0.515954
H -4.757663 0.284856 -1.397381
H -3.026841 0.15361 -1.371481
C -3.160561 2.091695 0.383971
H -3.436429 0.191815 1.330111
H -4.930523 0.944126 0.841709
C -1.643535 1.929569 0.37426
H -3.430381 2.753486 1.214042
H -3.475653 2.60554 -0.532385
C -0.878649 3.237728 0.227112
H -1.361974 1.243954 -0.441136
H -1.35796 1.461792 1.334336
H -1.210679 3.920163 1.017261
H -1.159312 3.712295 -0.720972
C 2.813573 2.435143 -0.86035
C 3.416851 1.434681 0.126625
H 3.143481 3.447376 -0.602815
H 3.220537 2.231979 -1.8572
C 3.5738 0.025615 -0.435072

H 2.821152 1.416649 1.053127
H 4.40583 1.781133 0.443988
C 4.048241 -0.995013 0.592553
H 4.297036 0.057734 -1.258813
H 2.64603 -0.319975 -0.924772
C 4.284665 -2.38167 0.01362
H 3.330206 -1.058675 1.427186
H 4.970724 -0.621537 1.051502
H 3.367701 -2.795389 -0.415852
H 4.644155 -3.076135 0.77709
H 5.03839 -2.34622 -0.778174
N 0.598144 -0.587711 0.951506
H 1.243168 -0.683539 0.152281
H 1.118236 -0.159911 1.717546
H 0.909259 -2.483301 1.768241
H -0.149773 0.063413 0.683162
C -0.327933 -2.652945 0.013762
H -0.507649 -3.703945 0.275625
O 0.825822 -2.529656 -0.805281
H 0.664725 -2.922321 -1.667173
C 1.285853 2.437449 -0.920807
H 0.964965 2.980701 -1.816822
H 0.913891 1.411508 -1.066815
C 0.639413 3.100816 0.294071
H 1.076184 4.100354 0.397142
H 0.915219 2.574926 1.2211
C -4.053651 -1.563347 -0.561968
H -4.951189 -1.770331 0.031164
H -4.209706 -2.054711 -1.529255
C -2.853025 -2.207754 0.132611
H -2.735395 -1.766127 1.129829
H -3.06177 -3.27036 0.299896
H -0.737889 -1.845484 2.005077

conf_02

1 1
C -1.858972 1.664669 -0.735481
C -4.087188 0.5306 0.016241
H -4.531442 0.57084 -0.98581
H -4.912328 0.750284 0.703673
C -2.221564 -2.860879 -0.643438
C -0.830243 -2.508041 -0.138088
H -2.128334 -3.469567 -1.548733
H -2.74169 -3.483123 0.094392
C 0.099226 -3.6815 0.146793
H -0.908636 -1.922394 0.792514
H -0.350323 -1.873312 -0.901285
C 1.40681 -3.24518 0.804174
H 0.3051 -4.216456 -0.788169
H -0.41705 -4.391867 0.801916
C 2.246639 -2.296903 -0.043039
H 2.002207 -4.130557 1.051167

H 1.176447 -2.762436 1.764695
H 1.621983 -1.464473 -0.392367
H 2.582602 -2.813457 -0.952042
C 3.546123 0.381446 -0.796709
C 2.802974 1.282061 0.182801
H 2.842382 -0.003651 -1.54728
H 4.262304 0.992854 -1.357566
C 2.133936 2.473261 -0.493611
H 3.502697 1.654113 0.94114
H 2.061694 0.694474 0.748243
C 1.429096 3.433466 0.457817
H 1.434728 2.127139 -1.280266
H 2.890101 3.030021 -1.059774
C 0.814877 4.63443 -0.246354
H 2.153509 3.778854 1.20341
H 0.658634 2.907782 1.042659
H 1.584738 5.229688 -0.744919
H 0.292739 5.289242 0.455162
H 0.098739 4.33321 -1.02043
N -0.803396 0.828414 -0.086588
H -0.886753 0.996226 0.926547
H 0.137097 1.098574 -0.390623
H -1.440949 2.668889 -0.809728
H -0.923599 -0.177701 -0.251145
C -3.107903 1.693529 0.148094
H -3.640106 2.614497 -0.120181
O -2.568797 1.810991 1.45927
H -3.268459 1.916279 2.108572
C 4.300315 -0.779131 -0.149061
H 4.791491 -1.355481 -0.941517
H 5.10656 -0.376672 0.475134
C 3.444925 -1.720479 0.697244
H 3.091961 -1.199159 1.596994
H 4.076086 -2.538677 1.061803
C -3.053277 -1.617374 -0.949759
H -2.465433 -0.95623 -1.60733
H -3.930255 -1.888088 -1.546622
C -3.536349 -0.869913 0.29607
H -4.320545 -1.473842 0.761528
H -2.752178 -0.808489 1.061325
H -2.058117 1.28147 -1.735305

conf_03

1 1
C -1.571838 -2.587945 -0.430945
C 0.901182 -2.128964 -0.577665
H 0.643222 -1.276446 -1.222912
H 1.026815 -2.990088 -1.245694
C 4.654678 -1.177628 -0.125187
C 4.601285 0.227409 0.478935
H 5.468128 -1.205312 -0.859365
H 4.933793 -1.892027 0.659473

C 4.323633 1.349785 -0.529318
H 5.569254 0.408642 0.956432
H 3.871429 0.265603 1.297142
C 2.856771 1.75136 -0.6955
H 4.730185 1.0565 -1.505034
H 4.882747 2.245908 -0.236709
C 2.290766 2.523967 0.492492
H 2.246894 0.861592 -0.892375
H 2.762594 2.381131 -1.588964
H 2.913025 3.41129 0.654811
H 2.374917 1.929547 1.412354
C -1.60242 2.233096 0.077245
C -2.567206 1.055561 0.104043
H -1.649537 2.717465 -0.905459
H -1.921842 2.986617 0.80661
C -4.010964 1.391843 -0.235108
H -2.529128 0.593621 1.102607
H -2.203831 0.302109 -0.613166
C -4.92119 0.168958 -0.246201
H -4.052978 1.879399 -1.216317
H -4.398348 2.121124 0.485867
C -6.369062 0.485111 -0.590913
H -4.886817 -0.311933 0.744577
H -4.533094 -0.552983 -0.98762
H -6.794621 1.184584 0.133676
H -6.992111 -0.412595 -0.593174
H -6.44193 0.945882 -1.579762
N -2.696373 -2.380845 0.53587
H -2.967335 -3.236401 1.020463
H -3.52588 -1.958191 0.109842
H -1.665564 -3.566503 -0.901579
H -2.300417 -1.711495 1.229156
C -0.261519 -2.415389 0.352761
H -0.058832 -3.327074 0.927302
O -0.484696 -1.399564 1.320502
H -0.140592 -0.553283 1.007972
C -0.164071 1.820503 0.363655
H -0.109642 1.371704 1.36903
H 0.130514 1.049087 -0.364539
C 0.840856 2.964943 0.307053
H 0.578175 3.69759 1.078392
H 0.740089 3.483962 -0.654721
C 3.378562 -1.664231 -0.806932
H 3.100095 -0.968926 -1.608471
H 3.579917 -2.622423 -1.300671
C 2.207279 -1.839706 0.151627
H 2.426838 -2.650661 0.856823
H 2.092762 -0.935497 0.760012
H -1.667163 -1.811127 -1.191363

conf_04

1 1

C 2.6116 -0.488929 -2.118772
C 1.893923 -1.22136 0.236022
H 1.742085 -2.13913 0.817428
H 0.890582 -0.794112 0.112396
C 0.98599 0.975804 2.329713
C 1.26071 2.417767 1.876986
H 0.507081 0.992166 3.313225
H 0.243281 0.513573 1.667034
C 0.18462 2.970241 0.946106
H 2.230133 2.479628 1.364449
H 1.355939 3.061323 2.755953
C 0.292885 2.385381 -0.460272
H 0.26078 4.060402 0.876363
H -0.805373 2.756161 1.365963
C -0.902016 2.671471 -1.370669
H 0.424133 1.296947 -0.333713
H 1.217007 2.767694 -0.915589
H -0.621519 2.529197 -2.42565
H -1.153822 3.733821 -1.284846
C -3.197893 -0.516791 -1.218621
C -3.702666 -0.558496 0.223786
H -2.977183 -1.535161 -1.558472
H -4.004097 -0.153385 -1.865954
C -2.656424 -0.944375 1.265872
H -4.535378 -1.269338 0.272744
H -4.129186 0.414021 0.49341
C -1.981908 -2.290375 1.027734
H -3.137516 -0.960968 2.25078
H -1.887084 -0.162404 1.334535
C -1.064684 -2.69322 2.17359
H -1.411302 -2.262143 0.088873
H -2.750149 -3.059873 0.883998
H -0.295822 -1.934171 2.353472
H -0.562595 -3.645752 1.979318
H -1.628439 -2.805976 3.104098
N 1.250027 -0.054609 -2.56541
H 0.687967 -0.912165 -2.673175
H 1.269804 0.452507 -3.449277
H 3.137186 -0.843267 -3.00587
H 0.785594 0.541518 -1.868556
C 2.437953 -1.630121 -1.125068
H 3.424613 -2.095626 -1.005264
O 1.550888 -2.508216 -1.806972
H 1.324366 -3.257323 -1.24954
C -1.959816 0.349734 -1.435166
H -1.135586 -0.081769 -0.847308
H -1.674513 0.281156 -2.497066
C -2.136078 1.821012 -1.075906
H -2.404685 1.914165 -0.018223
H -2.987719 2.219287 -1.638119
C 2.226917 0.088066 2.395401
H 3.003187 0.597736 2.976324
H 1.995841 -0.83319 2.942861
C 2.79852 -0.280932 1.02592

H 2.980941 0.640461 0.457567
H 3.778214 -0.75443 1.157095
H 3.136142 0.367145 -1.697548

conf_05

1 1
C -1.306932 -2.214502 1.2503
C 0.99374 -2.313774 0.213156
H 0.971013 -3.409307 0.252442
H 1.414903 -1.962298 1.162064
C 3.996503 -1.51036 0.466497
C 3.67191 -0.042063 0.731144
H 5.082108 -1.63264 0.382496
H 3.706392 -2.098549 1.345964
C 4.036931 0.91453 -0.39797
H 4.190587 0.269754 1.645986
H 2.60157 0.058696 0.957277
C 3.812977 2.388571 -0.048962
H 3.470941 0.659253 -1.302293
H 5.090807 0.773006 -0.665382
C 2.436071 2.709173 0.531245
H 3.987377 2.996255 -0.944566
H 4.570965 2.6995 0.67937
H 2.387793 3.778195 0.766877
H 2.32334 2.19268 1.492883
C -1.251207 1.914784 -0.491896
C -2.521582 1.654078 0.305345
H -0.996471 1.016893 -1.074405
H -1.445697 2.69523 -1.236737
C -3.669665 1.096147 -0.525653
H -2.84523 2.575522 0.803489
H -2.278235 0.972759 1.144701
C -4.936741 0.814186 0.274267
H -3.341486 0.186247 -1.054944
H -3.907316 1.804934 -1.327002
C -6.033591 0.155226 -0.548094
H -5.304416 1.75398 0.700331
H -4.706689 0.18844 1.153999
H -6.318995 0.787753 -1.393132
H -6.931496 -0.024307 0.047635
H -5.708016 -0.806367 -0.961725
N -2.714619 -1.841402 0.896818
H -2.861783 -0.825295 0.933473
H -3.410674 -2.289007 1.49239
H -1.038855 -1.736117 2.192083
H -2.852085 -2.127385 -0.08373
C -0.419175 -1.77495 0.10028
H -0.377393 -0.675327 0.084206
O -1.11884 -2.236902 -1.048973
H -0.608445 -2.059886 -1.843483
C -0.065281 2.311266 0.376947
H -0.257341 3.286869 0.840091

H 0.02352 1.601444 1.214298
C 1.261488 2.34008 -0.36875
H 1.204734 3.036267 -1.214593
H 1.429863 1.349737 -0.811277
C 3.356011 -2.107744 -0.788278
H 3.859387 -1.716544 -1.678382
H 3.532086 -3.189514 -0.79729
C 1.862121 -1.83248 -0.946238
H 1.709636 -0.753313 -1.072954
H 1.52951 -2.305574 -1.879503
H -1.284312 -3.299979 1.363355

conf_06

1 1
C 0.460916 -1.397797 -0.883447
C 2.583578 -2.764272 -0.718216
H 2.515614 -3.260728 -1.693542
H 3.066907 -3.494834 -0.056893
C 4.388958 0.59734 0.209241
C 3.364892 1.663748 -0.181928
H 5.155754 0.513595 -0.569588
H 4.912153 0.927397 1.113505
C 2.531437 2.163489 0.996768
H 2.711667 1.273136 -0.975326
H 3.87922 2.519998 -0.631583
C 1.341177 3.033 0.598006
H 3.188967 2.730219 1.664736
H 2.166803 1.314947 1.592516
C 0.173761 2.234746 0.029554
H 1.660535 3.782108 -0.137276
H 0.986643 3.593899 1.469764
H -0.160797 1.535614 0.8125
H 0.528518 1.632898 -0.820928
C -2.997309 1.534431 0.020535
C -4.049433 0.618472 -0.597511
H -3.495142 2.278126 0.65371
H -2.361427 0.974514 0.724419
C -4.828806 -0.219543 0.414519
H -3.58062 -0.035228 -1.349027
H -4.749966 1.236599 -1.168739
C -4.036528 -1.370587 1.034163
H -5.722336 -0.631672 -0.067591
H -5.19224 0.43785 1.212061
C -3.766676 -2.518041 0.065285
H -4.587995 -1.768161 1.891267
H -3.099043 -0.986569 1.462035
H -4.697551 -3.022393 -0.207379
H -3.112441 -3.282161 0.500254
H -3.340439 -2.179404 -0.891102
N -0.805161 -1.109994 -0.148243
H -1.562156 -1.767881 -0.360116
H -1.15176 -0.162009 -0.322581

H 0.23569 -1.584188 -1.933453
H -0.583739 -1.20538 0.85307
C 1.153934 -2.574358 -0.214592
H 0.588066 -3.49476 -0.423992
O 1.04292 -2.27514 1.173599
H 1.525261 -2.919205 1.697622
C -2.149497 2.259489 -1.021914
H -1.746308 1.536019 -1.751776
H -2.810721 2.90531 -1.609138
C -0.997499 3.082297 -0.450633
H -1.364059 3.707955 0.372221
H -0.632841 3.773164 -1.218251
C 3.765849 -0.777659 0.450834
H 4.443435 -1.397564 1.049303
H 2.859276 -0.662808 1.053307
C 3.453348 -1.515137 -0.850763
H 3.000129 -0.826256 -1.575023
H 4.396781 -1.819375 -1.316509
H 1.070305 -0.499408 -0.792966

conf_07

1 1
C 0.753811 1.621495 -0.40461
C -1.082897 3.236937 0.36882
H -1.192191 4.257545 0.750177
H -1.357636 2.592622 1.213624
C -2.860309 0.705968 -0.091865
C -4.045717 -0.240884 0.059081
H -2.340136 0.718992 0.882318
H -2.161257 0.294054 -0.835431
C -3.618984 -1.660091 0.424253
H -4.618201 -0.255157 -0.876499
H -4.719914 0.156766 0.825697
C -2.95654 -2.424636 -0.731065
H -4.487749 -2.222508 0.778217
H -2.939245 -1.6073 1.285878
C -1.788609 -3.312731 -0.311463
H -2.593999 -1.719387 -1.491392
H -3.712277 -3.031625 -1.238179
H -1.515451 -3.972966 -1.142162
H -2.093096 -3.968427 0.513739
C 1.877255 -2.551795 0.872482
C 2.541047 -1.798218 -0.27953
H 2.625683 -3.210273 1.325164
H 1.599696 -1.858304 1.686345
C 3.656404 -0.855569 0.155732
H 1.797516 -1.241323 -0.875062
H 2.943928 -2.530292 -0.988766
C 4.337566 -0.138328 -1.002586
H 4.40397 -1.420686 0.725024
H 3.273857 -0.106117 0.869633
C 5.383316 0.867959 -0.547552

H 3.580777 0.36521 -1.621395
H 4.799675 -0.884041 -1.659415
H 4.940187 1.649583 0.079314
H 5.864359 1.356751 -1.397951
H 6.167098 0.381568 0.04041
N 0.549 0.685918 0.741516
H 0.903534 1.15866 1.583055
H 1.046962 -0.199834 0.609676
H 1.811277 1.572801 -0.665693
H -0.444625 0.470143 0.868424
C 0.394469 3.025472 0.056401
H 0.711178 3.711395 -0.739739
O 1.201413 3.189153 1.214739
H 1.052599 4.051997 1.610027
C 0.649867 -3.365974 0.470886
H 0.908006 -4.019195 -0.371448
H 0.372107 -4.03012 1.29631
C -0.557504 -2.513875 0.09959
H -0.302743 -1.840772 -0.733367
H -0.815451 -1.880018 0.962892
C -3.234138 2.128523 -0.490835
H -3.885629 2.086205 -1.370068
H -3.830684 2.587229 0.306847
C -2.034682 3.016087 -0.818028
H -1.495662 2.570706 -1.66445
H -2.389545 3.982564 -1.184423
H 0.15264 1.276866 -1.244118

conf_08

1 1
C -2.316549 -1.630676 -0.334292
C -4.199376 0.148625 -0.076173
H -4.522065 -0.22249 0.904331
H -5.122842 0.432305 -0.594024
C -1.400614 2.469314 1.42196
C -0.232277 2.203554 0.482374
H -1.018529 2.557426 2.444337
H -1.85786 3.436536 1.181479
C 0.876693 3.250267 0.520418
H -0.588494 2.13886 -0.558043
H 0.205496 1.226295 0.746713
C 2.031432 2.91502 -0.420455
H 1.247023 3.349112 1.548353
H 0.448823 4.222432 0.252017
C 2.873827 1.727251 0.04017
H 2.673303 3.792749 -0.545376
H 1.620542 2.70442 -1.418357
H 2.222608 0.979248 0.508718
H 3.562579 2.047289 0.831666
C 3.576664 -1.244938 0.040974
C 2.318609 -1.659322 -0.714881
H 3.303469 -0.872477 1.034126

H 4.192207 -2.135515 0.214861
C 1.512504 -2.757542 -0.022147
H 2.594634 -1.99509 -1.721626
H 1.694376 -0.762112 -0.8619
C 0.99253 -2.401501 1.368659
H 2.129402 -3.66059 0.054614
H 0.669681 -3.072308 -0.664628
C 0.106434 -3.483991 1.967028
H 0.452242 -1.440221 1.343681
H 1.840294 -2.22095 2.035665
H -0.760921 -3.700292 1.332664
H -0.261915 -3.203041 2.956796
H 0.659708 -4.420725 2.076598
N -1.156867 -0.829691 -0.830395
H -1.362037 -0.587609 -1.809439
H -0.278306 -1.353829 -0.774674
H -2.184838 -2.634164 -0.740635
H -1.030652 0.046546 -0.311806
C -3.618095 -1.025256 -0.858928
H -4.35415 -1.838597 -0.844822
O -3.295979 -0.685469 -2.201899
H -4.075242 -0.382866 -2.674487
C 4.418118 -0.190731 -0.679082
H 5.252513 0.088454 -0.025955
H 4.869814 -0.635235 -1.573598
C 3.650719 1.065264 -1.090276
H 2.950647 0.818217 -1.900258
H 4.352547 1.785577 -1.525358
C -2.463835 1.375136 1.374498
H -1.973568 0.400946 1.536898
H -3.145602 1.480109 2.224969
C -3.302138 1.376904 0.093456
H -3.929273 2.272961 0.113711
H -2.679924 1.503311 -0.801969
H -2.27768 -1.677358 0.752904

conf_09

1 1
C 2.068431 1.763663 1.150845
C 4.263815 0.768964 0.160188
H 4.724701 0.600399 1.141176
H 5.082752 1.102747 -0.487396
C 2.375514 -2.681522 0.18264
C 0.981479 -2.283805 -0.284341
H 2.280974 -3.432946 0.973495
H 2.918529 -3.166438 -0.637279
C 0.098344 -3.439408 -0.744506
H 1.049727 -1.567158 -1.118422
H 0.473313 -1.776113 0.552291
C -1.286394 -2.97826 -1.193957
H 0.00579 -4.170132 0.068444
H 0.600642 -3.957363 -1.568892

C -2.172859 -2.477696 -0.055321
H -1.792983 -3.793307 -1.720531
H -1.163813 -2.176882 -1.93682
H -1.561589 -1.921588 0.665375
H -2.574162 -3.331038 0.504754
C -3.271805 -0.124877 1.593387
C -2.290426 0.855346 0.953272
H -2.711123 -0.814463 2.234174
H -3.94056 0.42603 2.263889
C -2.919753 1.987177 0.153978
H -1.627713 0.281971 0.284791
H -1.670954 1.291569 1.756761
C -1.88978 2.886203 -0.518261
H -3.554111 2.588286 0.816286
H -3.584566 1.572409 -0.612034
C -2.504923 4.055347 -1.272545
H -1.292723 2.28312 -1.220145
H -1.195606 3.276243 0.243224
H -3.182961 3.701157 -2.054209
H -1.7417 4.675799 -1.748657
H -3.08194 4.694124 -0.598051
N 1.011762 1.069827 0.356949
H 1.032824 1.482672 -0.585748
H 0.070262 1.186951 0.746979
H 1.65489 2.735539 1.42291
H 1.185291 0.063326 0.261735
C 3.311317 1.95557 0.281198
H 3.865639 2.789287 0.729723
O 2.767605 2.352047 -0.971581
H 3.465882 2.592687 -1.585274
C -4.112443 -0.925736 0.593993
H -4.663985 -1.691448 1.150799
H -4.874668 -0.277494 0.148302
C -3.312806 -1.584163 -0.528828
H -2.899636 -0.80694 -1.187303
H -3.995207 -2.162924 -1.161108
C 3.186854 -1.500247 0.70875
H 2.587458 -0.974155 1.46995
H 4.06189 -1.86413 1.257317
C 3.677691 -0.539513 -0.377768
H 4.446805 -1.061145 -0.954689
H 2.893678 -0.31807 -1.113462
H 2.272243 1.191996 2.055307

13*Z*-1-DeoxymethylSO

conf_00

1 1
C 0.44935 -1.355957 -0.354469
C 2.327816 -2.607707 0.760204
H 3.180494 -3.259485 0.532413
H 1.626842 -3.208956 1.351791

C 3.584869 0.481011 -0.008936
C 2.679825 1.5897 0.514551
H 3.120966 0.026904 -0.895618
H 4.511324 0.933625 -0.380235
C 2.323786 2.612719 -0.557561
H 3.170187 2.094989 1.356138
H 1.760869 1.161674 0.936402
C 1.31825 3.675164 -0.116143
H 1.944222 2.08837 -1.446236
H 3.240022 3.111892 -0.891936
C -0.027452 3.133453 0.364578
H 1.150163 4.367313 -0.949457
H 1.759296 4.274281 0.688472
H -0.67036 3.9759 0.643269
H 0.110271 2.552747 1.285482
C -2.745424 0.787138 -1.104692
C -3.242085 -0.442213 -0.892836
H -2.887783 1.218459 -2.096712
C -3.261257 -1.21374 0.391481
H -3.771482 -0.913181 -1.722634
C -4.663675 -1.244941 1.018578
H -2.559264 -0.791672 1.11929
H -2.949215 -2.249124 0.198751
C -5.14195 0.1236 1.47689
H -4.642962 -1.936976 1.866506
H -5.370188 -1.667212 0.294807
H -4.464503 0.549255 2.22453
H -6.133335 0.057995 1.930692
H -5.207023 0.827788 0.642388
N -0.326719 -1.286539 -1.627123
H 0.183472 -0.771583 -2.345889
H -1.256671 -0.822376 -1.49541
H 0.749872 -0.347397 -0.077211
H -0.449401 -2.249355 -1.962865
C 1.63355 -2.281677 -0.553158
H 2.341654 -1.819189 -1.254872
O 1.056037 -3.431704 -1.161217
H 1.713821 -4.123896 -1.264853
C -2.052788 1.673572 -0.119712
H -2.737119 2.491641 0.142373
H -1.855516 1.143153 0.817508
C -0.757049 2.276402 -0.661301
H -0.970767 2.870614 -1.558581
H -0.091498 1.468349 -0.995793
C 3.95677 -0.586401 1.021195
H 4.707657 -1.259361 0.589253
H 4.460465 -0.081334 1.85301
C 2.818569 -1.428199 1.605324
H 3.168904 -1.85952 2.54779
H 1.974919 -0.787704 1.890373
H -0.22622 -1.758771 0.402699

conf_01

1 1
C -0.219138 1.655322 -1.266989
C -2.02923 2.524466 0.355965
H -2.272708 3.451361 0.887112
H -1.768867 1.805524 1.143589
C -3.762164 -0.149177 0.780369
C -3.952003 -1.662638 0.664218
H -4.687484 0.291919 1.167778
H -3.000003 0.071424 1.538787
C -2.784002 -2.408718 0.022396
H -4.854163 -1.862759 0.075327
H -4.146058 -2.071172 1.662364
C -1.453642 -2.259644 0.752183
H -2.664271 -2.075762 -1.016603
H -3.032697 -3.473078 -0.04388
C -0.260485 -2.66402 -0.102616
H -1.466588 -2.842187 1.681016
H -1.329465 -1.216666 1.093238
H -0.259625 -2.052825 -1.017997
H -0.397672 -3.692533 -0.456431
C 2.372549 -1.626332 -1.358475
C 3.14515 -0.529407 -1.324407
H 1.757461 -1.772684 -2.246488
C 4.114546 -0.136189 -0.253494
H 3.096497 0.14308 -2.182871
C 3.593364 0.964331 0.67783
H 5.0295 0.225487 -0.736674
H 4.404064 -1.002255 0.348365
C 4.661576 1.479779 1.629832
H 2.756553 0.560103 1.266577
H 3.200987 1.800488 0.082611
H 5.060553 0.670616 2.248253
H 4.264186 2.2472 2.298354
H 5.496918 1.918467 1.077049
N 0.357219 0.682176 -0.296977
H 0.811154 1.233635 0.443374
H 1.085163 0.063346 -0.713956
H 0.605289 2.001401 -1.891811
H -0.357341 0.086526 0.129366
C -0.795179 2.827658 -0.48835
H -1.042005 3.60451 -1.223546
O 0.29969 3.235643 0.321784
H 0.044513 3.97474 0.879816
C 2.296442 -2.699123 -0.312616
H 2.249441 -3.673481 -0.811691
H 3.205106 -2.703484 0.294204
C 1.081023 -2.555121 0.61263
H 1.153826 -1.593963 1.144789
H 1.13425 -3.318679 1.395598
C -3.410277 0.532215 -0.537931
H -2.498229 0.082193 -0.955211
H -4.193323 0.307832 -1.271283
C -3.254615 2.049166 -0.433567

H -3.232142 2.49067 -1.437854
H -4.155392 2.452055 0.039097
H -0.958972 1.153168 -1.887662

conf_02

1 1
C 0.757535 -1.615632 0.322211
C 3.210878 -2.230185 0.269451
H 4.017409 -2.444999 -0.443602
H 2.953122 -3.188339 0.735337
C 4.092377 0.849912 -0.179927
C 2.849863 1.593514 0.293213
H 3.879077 0.34099 -1.129902
H 4.871979 1.580323 -0.42373
C 2.239015 2.495391 -0.77375
H 3.096445 2.190873 1.17992
H 2.09849 0.871341 0.634213
C 0.887593 3.092872 -0.388707
H 2.127362 1.930539 -1.711451
H 2.942202 3.303436 -1.001275
C -0.245762 2.073513 -0.356857
H 0.623464 3.883554 -1.0993
H 0.963935 3.578009 0.592372
H 0.005876 1.277021 0.357853
H -0.324591 1.617665 -1.360351
C -2.620072 0.521136 0.95911
C -3.091956 -0.729779 0.836746
H -2.149637 0.786484 1.907299
C -3.862604 -1.299568 -0.31669
H -2.991824 -1.386844 1.702112
C -5.358609 -1.440499 0.004884
H -3.474186 -2.297538 -0.562096
H -3.751342 -0.677623 -1.212219
C -6.052767 -0.105012 0.219396
H -5.475492 -2.071191 0.893658
H -5.834422 -1.980779 -0.8197
H -5.621916 0.440849 1.063942
H -7.115476 -0.247488 0.427842
H -5.973219 0.528865 -0.669835
N -0.37636 -1.256637 -0.57522
H -0.170967 -0.382747 -1.0678
H -1.259712 -1.081471 -0.046991
H 0.856345 -0.830379 1.069882
H -0.489415 -1.994751 -1.274759
C 2.000043 -1.773605 -0.530373
H 2.211349 -0.810127 -1.015559
O 1.598119 -2.719078 -1.513006
H 2.342987 -2.95625 -2.071105
C -2.744567 1.641641 -0.029686
H -2.860764 1.256379 -1.049853
H -3.684404 2.166362 0.187042
C -1.600841 2.653408 0.023311

H -1.538488 3.079442 1.032064
H -1.835888 3.487795 -0.645292
C 4.663714 -0.142754 0.830775
H 5.556016 -0.609162 0.396985
H 5.015547 0.412234 1.708127
C 3.733342 -1.251913 1.329362
H 4.294322 -1.839484 2.061981
H 2.900366 -0.821131 1.898092
H 0.501921 -2.557413 0.811867

conf_03

1 1
C -0.835043 -2.031035 0.372009
C -3.345876 -1.584131 -0.035248
H -4.235366 -2.144806 -0.343349
H -3.164652 -0.87019 -0.849126
C -3.406674 1.585474 0.5129
C -2.679677 2.91933 0.690193
H -4.476853 1.74208 0.688621
H -3.332906 1.25927 -0.532579
C -1.160013 2.847045 0.554586
H -2.919736 3.325055 1.67942
H -3.077921 3.637422 -0.035485
C -0.662288 2.362209 -0.802554
H -0.749693 2.20183 1.342285
H -0.737412 3.838609 0.748219
C 0.806086 1.960927 -0.785954
H -0.830053 3.136863 -1.560247
H -1.279068 1.515184 -1.151697
H 0.953602 1.155545 -0.050258
H 1.402359 2.795858 -0.399344
C 2.619686 -0.585839 -1.616031
C 2.834137 -1.101635 -0.395684
H 2.368941 -1.285671 -2.415865
C 3.271487 -0.374084 0.834371
H 2.765524 -2.187105 -0.293509
C 4.679154 -0.790525 1.272533
H 2.575159 -0.593041 1.656833
H 3.239708 0.708827 0.682779
C 5.116046 -0.094986 2.552085
H 5.380581 -0.565472 0.461676
H 4.708505 -1.877995 1.408925
H 5.123898 0.99256 2.430053
H 6.123276 -0.402007 2.842739
H 4.444242 -0.332452 3.383044
N -0.375808 -1.173303 -0.757136
H -0.647418 -1.656349 -1.622935
H 0.658151 -1.025431 -0.765925
H -0.114989 -2.845647 0.460205
H -0.820519 -0.251319 -0.749709
C -2.203517 -2.594188 0.022255
H -2.433243 -3.358404 0.775952

O -1.979119 -3.194196 -1.247064
H -2.789141 -3.58846 -1.580521
C 2.726358 0.848328 -2.040656
H 3.369616 1.411951 -1.3584
H 3.216234 0.871479 -3.018924
C 1.361874 1.545038 -2.14293
H 0.652661 0.88454 -2.665478
H 1.450773 2.430528 -2.779935
C -2.913196 0.486809 1.448419
H -1.829415 0.355553 1.323708
H -3.035518 0.824759 2.483814
C -3.634095 -0.850448 1.27936
H -3.395489 -1.51076 2.122597
H -4.710671 -0.668313 1.350312
H -0.836261 -1.447535 1.290643

conf_04

1 1
C 0.219006 -1.655432 -1.266685
C 2.029387 -2.524684 0.355845
H 2.27298 -3.451563 0.88695
H 1.769233 -1.805752 1.143538
C 3.762119 0.148784 0.780303
C 3.952205 1.662228 0.664277
H 4.687282 -0.292477 1.167893
H 2.999778 -0.071729 1.538558
C 2.784266 2.408464 0.022516
H 4.854376 1.862285 0.075386
H 4.146353 2.070673 1.662437
C 1.453912 2.25962 0.752356
H 2.664408 2.07551 -1.016462
H 3.033093 3.472787 -0.043828
C 0.260869 2.66435 -0.102413
H 1.467021 2.842087 1.681232
H 1.329535 1.216643 1.093352
H 0.259845 2.053165 -1.01779
H 0.398406 3.69282 -0.456206
C -2.372172 1.626801 -1.358271
C -3.144854 0.529944 -1.323844
H -1.757019 1.772753 -2.246302
C -4.114395 0.137124 -0.252959
H -3.096201 -0.142868 -2.182057
C -3.594364 -0.964965 0.677134
H -5.029982 -0.222897 -0.736208
H -4.402591 1.003003 0.349783
C -4.663154 -1.480512 1.628439
H -2.75725 -0.562233 1.266473
H -3.202638 -1.80077 0.081013
H -5.061472 -0.671681 2.247714
H -4.266535 -2.249031 2.296155
H -5.49882 -1.917828 1.075067
N -0.357258 -0.682289 -0.296551

H -0.811199 -1.233755 0.443781
H -1.085135 -0.063344 -0.713473
H -0.605491 -2.001433 -1.891443
H 0.357372 -0.086708 0.129765
C 0.795125 -2.827813 -0.488169
H 1.041704 -3.604716 -1.223381
O -0.299597 -3.235636 0.322265
H -0.044707 -3.975376 0.879558
C -2.295943 2.699934 -0.312776
H -2.24856 3.674116 -0.812158
H -3.204709 2.704796 0.293878
C -1.080725 2.555867 0.612716
H -1.153803 1.594825 1.14504
H -1.133944 3.319597 1.395511
C 3.41045 -0.532466 -0.538109
H 2.498575 -0.082315 -0.955602
H 4.193709 -0.308142 -1.271256
C 3.254595 -2.049396 -0.433983
H 3.231757 -2.490674 -1.438362
H 4.155467 -2.452517 0.0383
H 0.958782 -1.153233 -1.887385

conf_05

1 1
C 0.144635 -1.195971 -0.362082
C 1.821759 -2.942816 0.312211
H 2.460042 -3.731304 -0.104419
H 1.16016 -3.423256 1.042146
C 4.251841 0.104523 0.756671
C 3.27117 1.266362 0.921353
H 5.110184 0.446434 0.16759
H 4.651237 -0.177206 1.737774
C 2.91294 1.967988 -0.387128
H 3.706728 2.007976 1.599941
H 2.362468 0.914864 1.427605
C 1.799721 3.005129 -0.252882
H 2.620821 1.232819 -1.151818
H 3.815003 2.45043 -0.779072
C 0.416022 2.393624 -0.072916
H 1.787545 3.646106 -1.141758
H 2.016489 3.666541 0.595151
H 0.408095 1.732752 0.804937
H 0.204222 1.760803 -0.949986
C -2.571912 1.938838 -0.806393
C -3.364228 0.857304 -0.739146
H -2.332 2.321635 -1.800556
C -3.917207 0.207297 0.490249
H -3.728317 0.445282 -1.683478
C -3.822129 -1.318577 0.476348
H -4.97832 0.482839 0.564556
H -3.443787 0.604123 1.3936
C -4.527174 -1.962592 1.660135

H -2.76611 -1.625527 0.483367
H -4.250099 -1.699725 -0.459762
H -4.106261 -1.615572 2.60859
H -4.439768 -3.051178 1.633333
H -5.592196 -1.714 1.660289
N -0.833489 -0.846637 -1.43186
H -0.356423 -0.509228 -2.269025
H -1.518682 -0.113033 -1.137469
H 0.767393 -0.325023 -0.170827
H -1.333707 -1.705649 -1.68793
C 0.951499 -2.397514 -0.811545
H 1.582968 -2.100275 -1.662458
O -0.031495 -3.321485 -1.258481
H 0.376416 -4.157771 -1.496708
C -2.067956 2.769493 0.333359
H -2.804608 3.565303 0.507281
H -2.03993 2.185157 1.259637
C -0.705019 3.410031 0.079166
H -0.470874 4.082865 0.910609
H -0.755779 4.040169 -0.817553
C 3.647085 -1.133329 0.094361
H 3.148107 -0.827523 -0.833957
H 4.446187 -1.81498 -0.217065
C 2.681485 -1.887317 1.007646
H 3.253492 -2.378898 1.80084
H 2.026758 -1.178664 1.529449
H -0.436254 -1.43377 0.531578

conf_06

1 1
C 0.434852 -1.298295 -0.421487
C 2.276404 -2.772444 0.448318
H 3.053209 -3.470136 0.112848
H 1.622875 -3.329639 1.129193
C 4.198607 0.601105 1.005023
C 3.049346 1.609669 1.037181
H 5.049968 1.051295 0.482334
H 4.540394 0.406402 2.028121
C 2.707318 2.209696 -0.325237
H 3.313082 2.428655 1.715394
H 2.160767 1.144176 1.483722
C 1.457269 3.087742 -0.32186
H 2.579885 1.41552 -1.076068
H 3.564877 2.79822 -0.669269
C 0.159034 2.297714 -0.215523
H 1.429713 3.690368 -1.236803
H 1.515252 3.801126 0.5096
H 0.179693 1.662672 0.680876
H 0.094467 1.621838 -1.083287
C -2.724125 1.411569 -1.050893
C -3.164195 0.146488 -0.954408
H -2.69161 1.856015 -2.047213

C -3.38199 -0.640077 0.303139
H -3.506129 -0.331847 -1.874837
C -4.857805 -0.667477 0.717447
H -2.787024 -0.234466 1.128805
H -3.052326 -1.679128 0.156025
C -5.095401 -1.508685 1.961223
H -5.457495 -1.050895 -0.115875
H -5.191355 0.362192 0.88578
H -4.795788 -2.549284 1.801083
H -6.151633 -1.508782 2.239744
H -4.528403 -1.124886 2.815233
N -0.448775 -1.082272 -1.603325
H 0.067787 -0.659107 -2.375504
H -1.266666 -0.464661 -1.389332
H 0.901324 -0.349268 -0.167309
H -0.774093 -2.003032 -1.91926
C 1.449661 -2.369557 -0.764437
H 2.111925 -1.987169 -1.555828
O 0.660636 -3.429698 -1.287648
H 1.208888 -4.194957 -1.478452
C -2.369361 2.339286 0.066784
H -3.212433 3.034278 0.178845
H -2.297644 1.800893 1.017352
C -1.097147 3.153753 -0.167858
H -1.000251 3.889297 0.637489
H -1.190127 3.72919 -1.097164
C 3.845069 -0.728303 0.338627
H 3.399644 -0.521041 -0.642436
H 4.760463 -1.291927 0.12761
C 2.91484 -1.595369 1.186444
H 3.475461 -1.988635 2.040222
H 2.123779 -0.977649 1.628804
H -0.2084 -1.623027 0.399129

conf_07

1 1
C 0.521163 -0.945251 -0.429528
C 2.39246 -2.429627 0.43542
H 2.491383 -3.502057 0.638965
H 2.117278 -1.973395 1.392693
C 3.680432 0.461746 0.872124
C 3.677375 1.956626 0.552162
H 4.490099 0.247278 1.578707
H 2.758115 0.198274 1.405466
C 2.433958 2.442202 -0.191901
H 4.569219 2.194646 -0.038723
H 3.773157 2.523564 1.485158
C 1.162996 2.40483 0.649391
H 2.291099 1.847114 -1.106229
H 2.599483 3.465868 -0.544718
C -0.112114 2.623527 -0.152357
H 1.23035 3.160587 1.440943

H 1.087552 1.444775 1.176303
H -0.119713 1.93854 -1.015014
H -0.110852 3.629401 -0.58988
C -2.853366 1.579712 -1.202717
C -3.487915 0.401703 -1.101648
H -2.465245 1.856537 -2.183096
C -4.135744 -0.176745 0.115676
H -3.588318 -0.19651 -2.010696
C -3.474896 -1.473345 0.593248
H -5.185865 -0.39103 -0.119734
H -4.141372 0.54574 0.936369
C -4.217742 -2.121401 1.751262
H -2.44279 -1.258698 0.906557
H -3.41733 -2.184568 -0.24338
H -4.276059 -1.443919 2.608029
H -3.721152 -3.03676 2.081527
H -5.240145 -2.380619 1.462644
N -0.682393 -0.975496 -1.313017
H -1.145371 -1.879666 -1.153404
H -0.424368 -0.926731 -2.299198
H 1.137823 -0.091079 -0.699186
H -1.372644 -0.21732 -1.124787
C 1.245386 -2.27364 -0.55887
H 1.643147 -2.374977 -1.582505
O 0.198002 -3.217344 -0.362308
H 0.54697 -4.112016 -0.365374
C -2.678629 2.589039 -0.106104
H -2.709429 3.59112 -0.547065
H -3.51981 2.533504 0.589734
C -1.372823 2.429239 0.678387
H -1.358629 1.433134 1.145075
H -1.369272 3.145556 1.507198
C 3.86486 -0.430875 -0.350888
H 3.166391 -0.148106 -1.149692
H 4.857726 -0.241305 -0.773704
C 3.749723 -1.925447 -0.060485
H 4.019531 -2.487619 -0.96267
H 4.496675 -2.195978 0.693617
H 0.154353 -0.819816 0.590624

conf_08

1 1
C 0.271351 -1.305236 -0.338031
C 1.98141 -2.8626 0.653909
H 2.711567 -3.630883 0.370393
H 1.223644 -3.365639 1.266747
C 3.65938 0.021816 -0.109618
C 2.963801 1.240062 0.485936
H 3.08775 -0.328244 -0.980424
H 4.62535 0.338104 -0.519393
C 2.721656 2.337979 -0.543102
H 3.566526 1.637399 1.312289

H 2.010587 0.946195 0.945114
C 1.916049 3.529686 -0.028066
H 2.223978 1.905779 -1.423057
H 3.688062 2.699095 -0.912081
C 0.524332 3.189904 0.504038
H 1.822006 4.264893 -0.835754
H 2.480822 4.027781 0.768063
H 0.033242 4.114016 0.828988
H 0.608986 2.569985 1.405597
C -2.584489 1.335308 -0.890101
C -3.250498 0.18904 -0.674997
H -2.696565 1.801728 -1.87003
C -3.322976 -0.587127 0.605613
H -3.868606 -0.183724 -1.491505
C -4.643566 -0.362005 1.358715
H -2.493148 -0.322449 1.269116
H -3.235386 -1.661003 0.387255
C -5.877114 -0.796197 0.581457
H -4.719801 0.699087 1.620237
H -4.590789 -0.912836 2.303533
H -6.020003 -0.201962 -0.325504
H -6.777267 -0.679882 1.189071
H -5.812077 -1.849512 0.288803
N -0.542228 -1.078192 -1.568138
H 0.008911 -0.628739 -2.300511
H -1.380243 -0.478292 -1.379707
H 0.737753 -0.364127 -0.053049
H -0.830986 -1.999572 -1.918001
C 1.286685 -2.396111 -0.616176
H 2.025205 -2.02809 -1.34209
O 0.510154 -3.425097 -1.219211
H 1.047502 -4.206339 -1.372454
C -1.729506 2.088954 0.077663
H -2.265151 3.002925 0.367007
H -1.588253 1.522823 1.004209
C -0.37236 2.483988 -0.50427
H -0.518156 3.122687 -1.384478
H 0.139986 1.585154 -0.874815
C 3.911807 -1.121348 0.8745
H 4.527879 -1.887502 0.387936
H 4.527377 -0.72523 1.689889
C 2.687153 -1.796958 1.498268
H 3.011685 -2.304348 2.411644
H 1.966468 -1.044211 1.840677
H -0.423189 -1.621867 0.442524

conf_09

1 1
C -0.605261 -2.407667 -0.641804
C -2.894161 -2.234456 0.399578
H -3.401524 -1.871237 1.300586
H -2.996216 -3.325636 0.404658

C -3.971612 0.683799 0.148053
C -3.85956 2.19427 -0.059673
H -3.469065 0.405907 1.083091
H -5.026825 0.4237 0.294095
C -2.43396 2.710977 -0.24386
H -4.322007 2.703692 0.79374
H -4.453556 2.474516 -0.937397
C -1.536314 2.537897 0.980308
H -2.472784 3.77557 -0.499189
H -1.981708 2.223079 -1.11567
C -0.048267 2.674213 0.666849
H -1.704638 1.555948 1.443383
H -1.825723 3.272519 1.739118
H 0.515426 2.848096 1.590445
H 0.117491 3.555963 0.035322
C 2.452496 0.385822 -1.223672
C 3.308198 -0.57349 -0.839907
H 2.069549 0.337787 -2.24448
C 4.015852 -0.692067 0.474315
H 3.590389 -1.317568 -1.588366
C 5.509301 -0.352243 0.359365
H 3.927112 -1.723902 0.841244
H 3.553519 -0.047654 1.229158
C 5.76449 1.109474 0.02718
H 5.966319 -0.995198 -0.402161
H 5.99284 -0.608869 1.307277
H 5.308692 1.392088 -0.926287
H 6.835481 1.311467 -0.046179
H 5.358094 1.767307 0.80228
N 0.84362 -2.390777 -0.265704
H 0.881152 -2.584375 0.74659
H 1.328601 -1.487366 -0.455868
H -0.746067 -1.806525 -1.539289
H 1.373416 -3.1082 -0.758657
C -1.420604 -1.892488 0.536364
H -1.292585 -0.80584 0.616424
O -0.816186 -2.522204 1.662421
H -1.296754 -2.310557 2.466514
C 1.985794 1.558594 -0.421199
H 2.599741 1.695552 0.472722
H 2.119238 2.461933 -1.028569
C 0.514514 1.440253 -0.027786
H 0.401526 0.581565 0.650986
H -0.07967 1.214523 -0.923711
C -3.418252 -0.138264 -1.010469
H -3.92741 0.159995 -1.93412
H -2.360991 0.111678 -1.17002
C -3.571551 -1.648745 -0.841333
H -4.635432 -1.902059 -0.78903
H -3.197051 -2.154483 -1.740143
H -0.871049 -3.445624 -0.851803

Synthesis Data

The 1-deoxysphingolipids 4*E*-1-deoxySO and 4*E*-3-deoxySO were purchased from Avanti Polar Lipids (Alabaster, USA). 3-Keto-1-deoxySA, 8*E*-1-deoxySO, 12*E*-1-deoxySO, 13*E*-1-deoxySO, 14*E*-1-deoxySO, 14*Z*-1-deoxySO, 13*Z*-1-deoxymethylSO, 1-deoxyPS, ω-OH-1-deoxySA and 5*E*,14*Z*-1-deoxysphingadiene were synthesized as reported previously.[9] The synthesis of 1-deoxySA, 5*E*-1-deoxySO, 1-deoxymethylSA and 6*E*-3-keto-1-deoxySO was carried out according to largely analogous synthesis routes.[10]

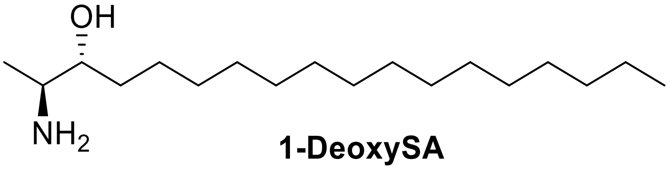

1-DeoxySA

R_f: 0.42 (EtOAc/ iso-propanol 9:1, visualized with 1.3% ninhydrine).

^{1}H NMR (MeOD, 500 MHz, ppm) δ 3.72 – 3.68 (m, 1H), 3.27 (qd, *J* = 6.8, 3.0 Hz, 1H), 1.53 (dt, *J* = 11.0, 8.2 Hz, 1H), 1.45 (dt, *J* = 13.6, 4.6 Hz, 2H), 1.38 – 1.27 (m, 25H), 1.22 (d, *J* = 6.8 Hz, 3H), 0.90 (t, *J* = 7.0 Hz, 3H).

^{13}C NMR (MeOD, 126 MHz, ppm) δ 71.65, 52.62, 34.00, 33.07, 30.78, 30.76, 30.72, 30.67, 30.63, 30.47, 26.97, 23.73, 14.43, 12.05.

ESI-MS: *m/z* calculated for $C_{18}H_{40}NO$ $[M+H]^+$ 286.32; observed 286.3.

^{1}H-NMR spectrum for 1-DeoxySA

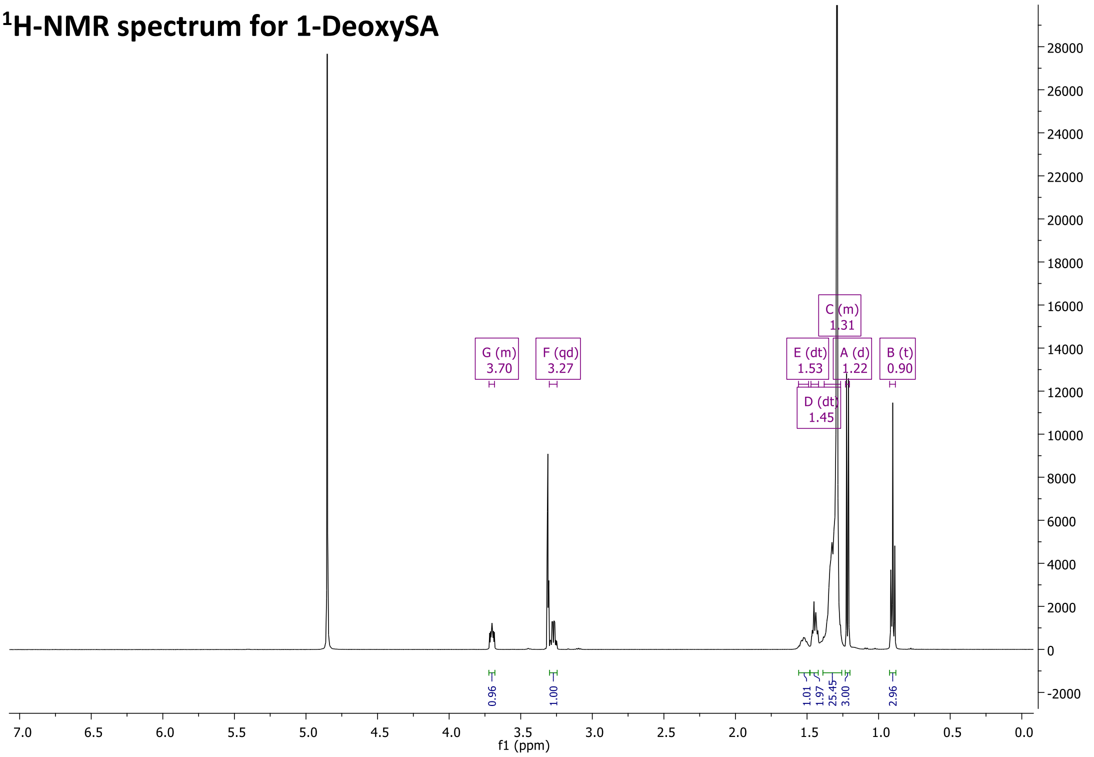

^{13}C-NMR spectrum for 1-DeoxySA

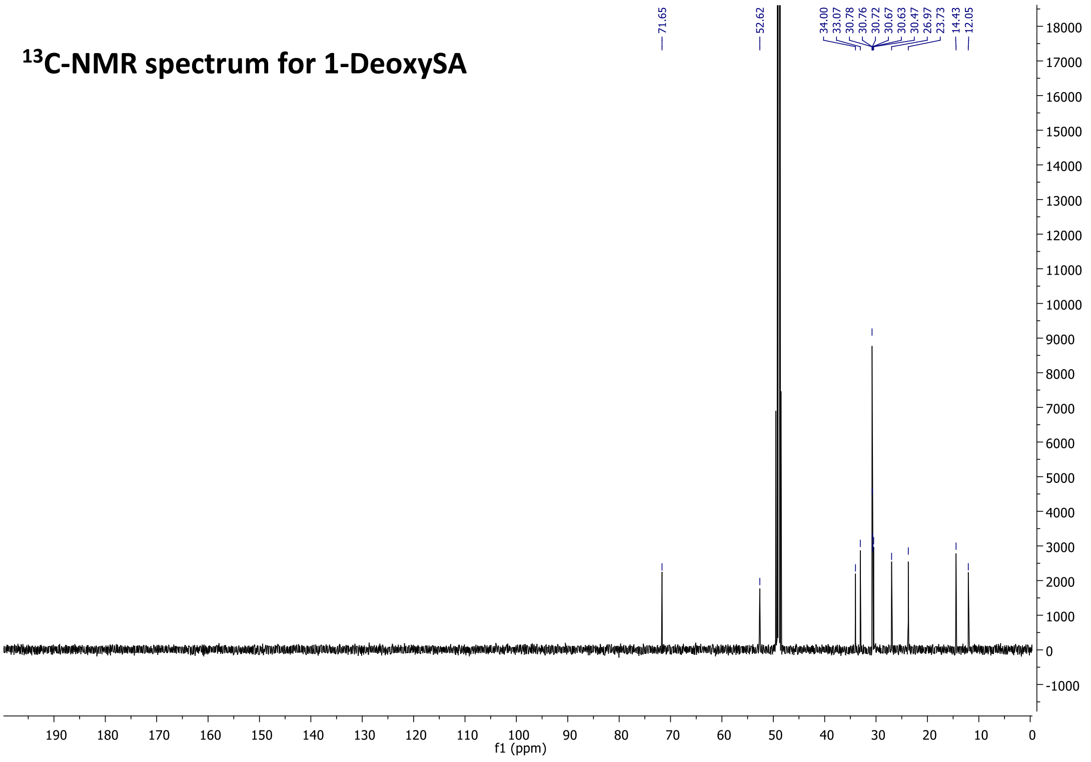

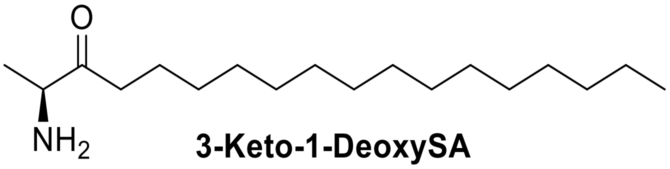

3-Keto-1-DeoxySA

R_f: 0.34 (EtOAc/ iso-propanol 9:1, visualized with $KMnO_4$ solution).

^{1}H NMR (MeOD, 500 MHz, ppm) δ 4.17 (q, *J* = 7.3 Hz, 1H), 2.68 (dt, *J* = 17.7, 7.3 Hz, 1H), 2.59 (dt, *J* = 17.7, 7.2 Hz, 1H), 1.68 – 1.60 (m, 2H), 1.54 (d, *J* = 7.4 Hz, 3H), 1.38 – 1.26 (m, 24H), 0.92 (t, *J* = 7.0 Hz, 3H).

^{13}C NMR (MeOD, 126 MHz, ppm) δ 207.44, 55.85, 39.21, 33.10, 30.80, 30.74, 30.63, 30.56, 30.50, 30.16, 24.31, 23.76, 15.75, 14.47, 2.31.

ESI-MS: *m/z* calculated for $C_{18}H_{37}NONa$ $[M+Na]^+$ 306.27; observed 306.3.

^{1}H-NMR spectrum for 3-Keto-1-DeoxySA

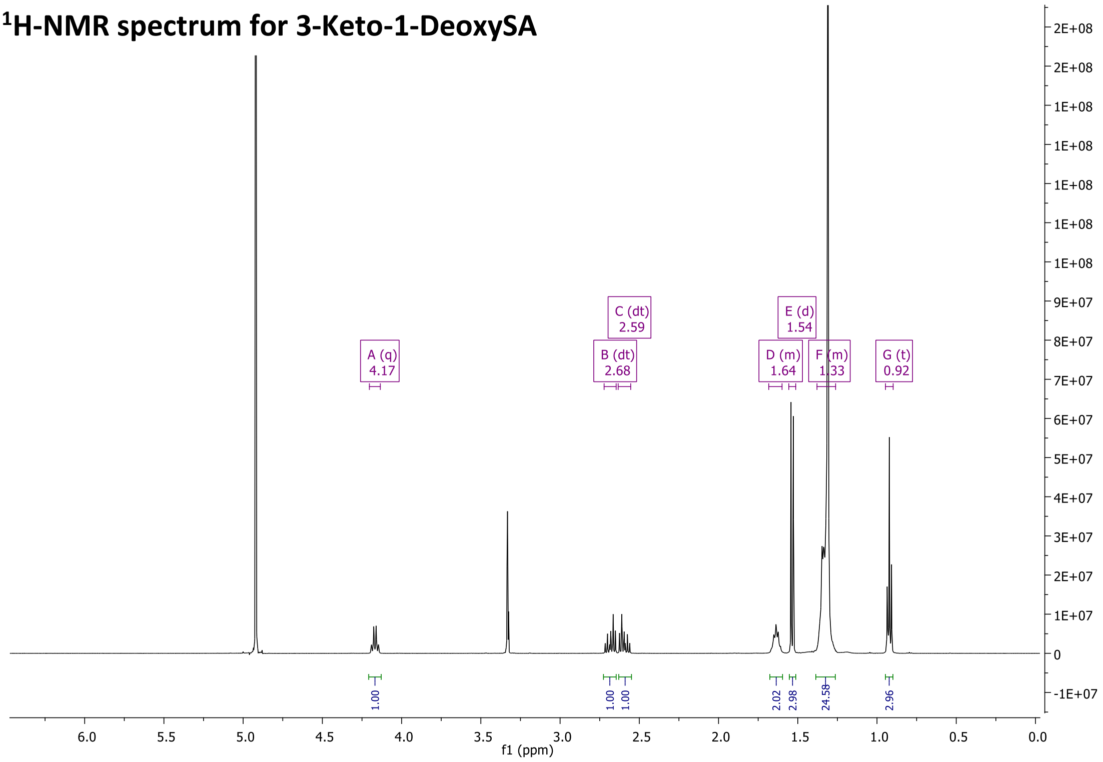

^{13}C-NMR spectrum for 3-Keto-1-DeoxySA

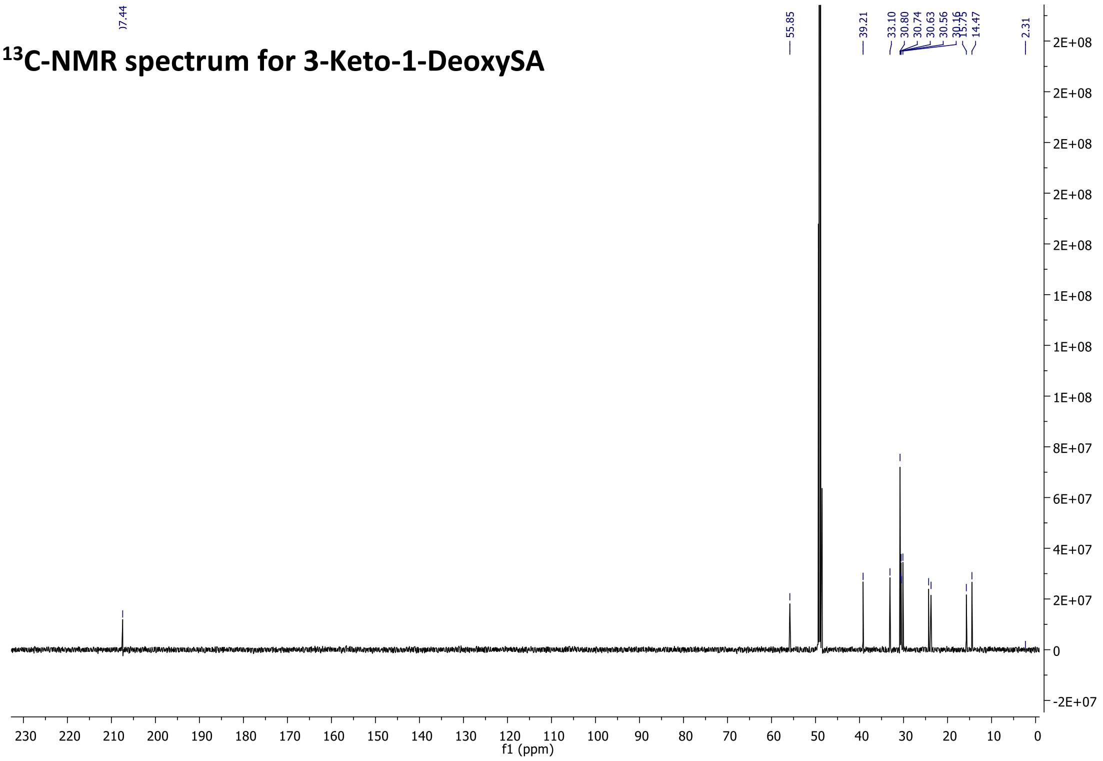

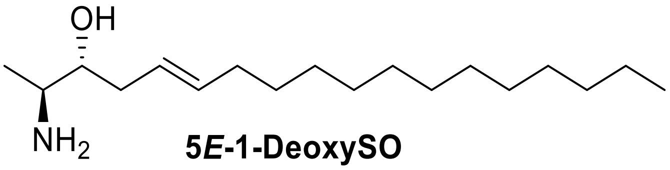

5*E*-1-DeoxySO

R_f: 0.45 (EtOAc/ iso-propanol 4:1, visualized with 1.3% ninhydrine).

^{1}H NMR (MeOD, 500 MHz, ppm) δ 5.58 (dd, *J* = 14.3, 7.5 Hz, 1H), 5.44 (dd, *J* = 14.6, 7.2 Hz, 1H), 3.78 – 3.72 (m, 1H), 3.26 (dd, *J* = 6.7, 2.8 Hz, 1H), 2.29 – 2.13 (m, 2H), 2.03 (dd, *J* = 13.3, 6.6 Hz, 2H), 1.42 – 1.27 (m, 20H), 1.23 (d, *J* = 6.8 Hz, 3H), 0.90 (t, *J* = 6.7 Hz, 3H).

^{13}C NMR (MeOD, 126 MHz, ppm) δ 135.37, 126.18, 71.69, 51.92, 37.68, 33.67, 33.04, 30.78, 30.70, 30.59, 30.43, 30.37, 23.77, 14.48, 11.62.

ESI-MS: *m/z* calculated for $C_{18}H_{38}NO$ $[M+H]^+$ 284.29; observed 284.3.

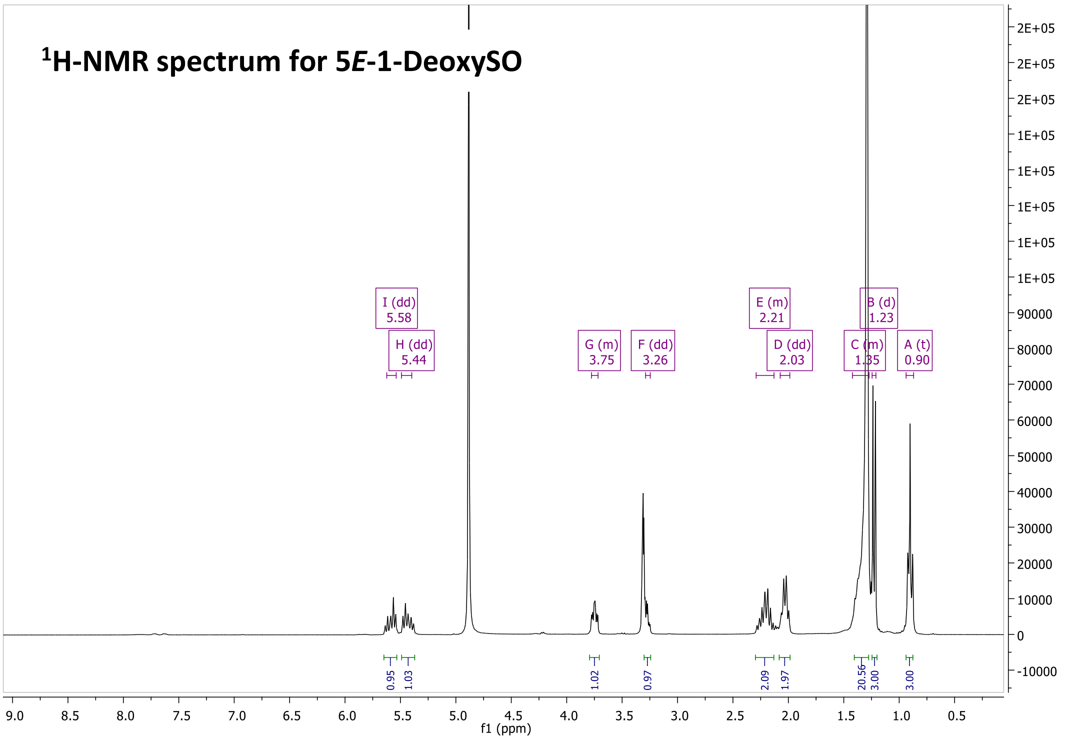

1H-NMR spectrum for 5E-1-DeoxySO
I (dd) 5.58
H (dd) 5.44
G (m) 3.75
F (dd) 3.26
E (m) 2.21
D (dd) 2.03
C (m) 1.35
B (d) 1.23
A (t) 0.90
0.95
1.03
1.02
0.97
2.09
1.97
20.56
3.00
3.00
f1 (ppm)

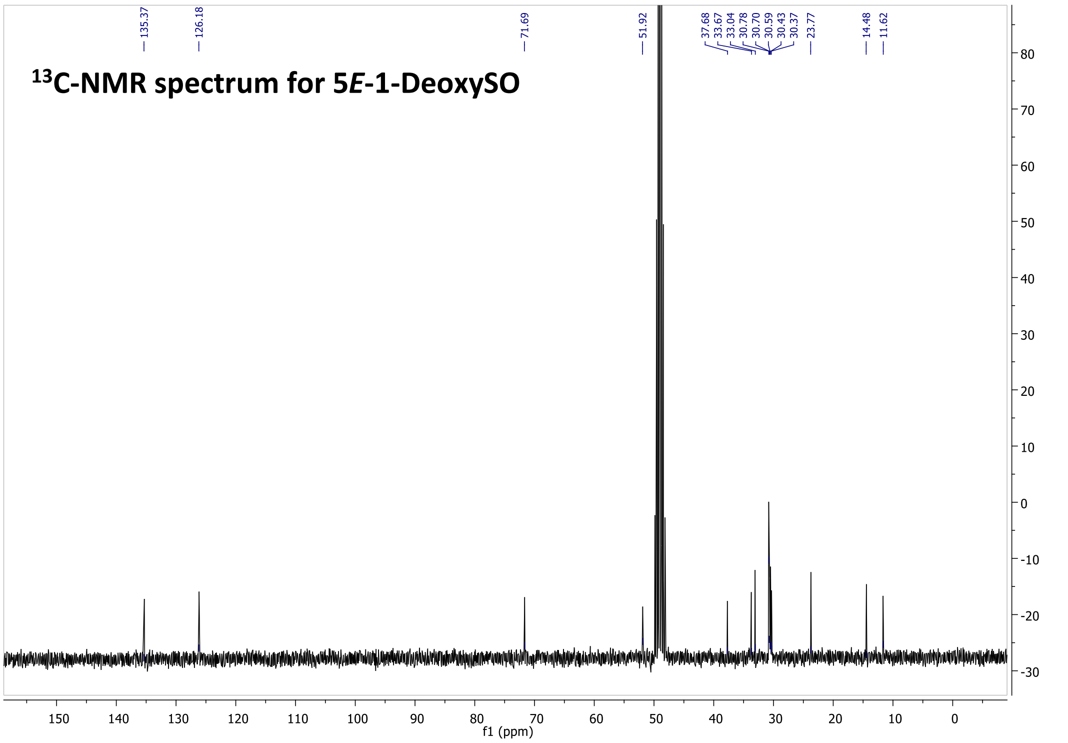

13C-NMR spectrum for 5E-1-DeoxySO
135.37
126.18
71.69
51.92
37.68
33.67
33.04
30.78
30.70
30.59
30.43
30.37
23.77
14.48
11.62
f1 (ppm)

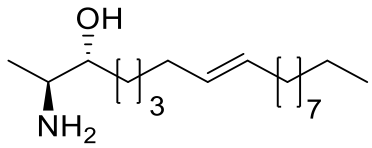

8*E*-1-DeoxySO

R$_f$: 0.45 (EtOAc/iso-propanol 4:1, visualized with $KMnO_4$ solution).

^{1}H NMR ($CDCl_3$, 500 MHz, ppm): δ 5.44 – 5.32 (m, 2H), 3.82 (m, 1H), 3.29 (m, 1H), 2.04 – 1.90 (m, 4H), 1.31 (m, 23H), 0.87 (t, J = 6.9 Hz, 3H).

^{13}C NMR ($CDCl_3$, 126 MHz, ppm): δ 130.52, 130.43, 70.85, 51.68, 32.73, 32.43, 31.98, 31.56, 29.82, 29.62, 29.49, 29.32, 25.87, 22.69, 14.21, 11.19.

ESI-MS: *m/z* calculated for $C_{18}H_{38}NO$ $[M+H]^+$ 284.29; observed 284.3.

^{1}H-NMR spectrum for 8*E*-1-DeoxySO

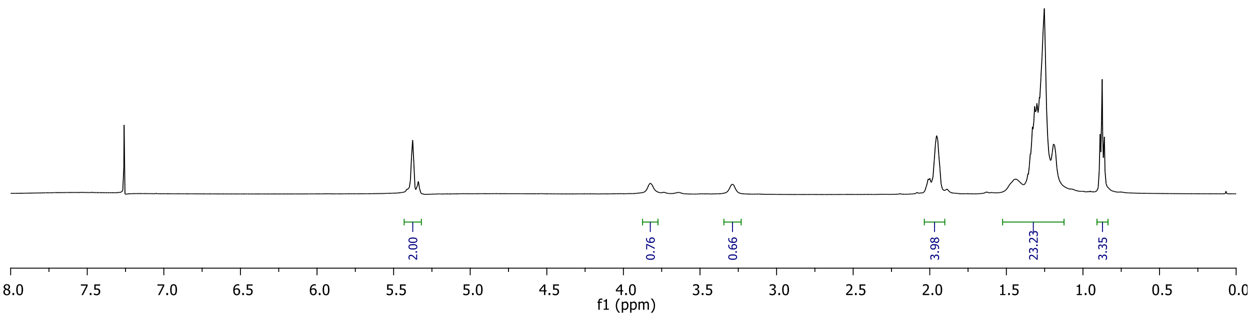

^{13}C-NMR spectrum for 8*E*-1-DeoxySO

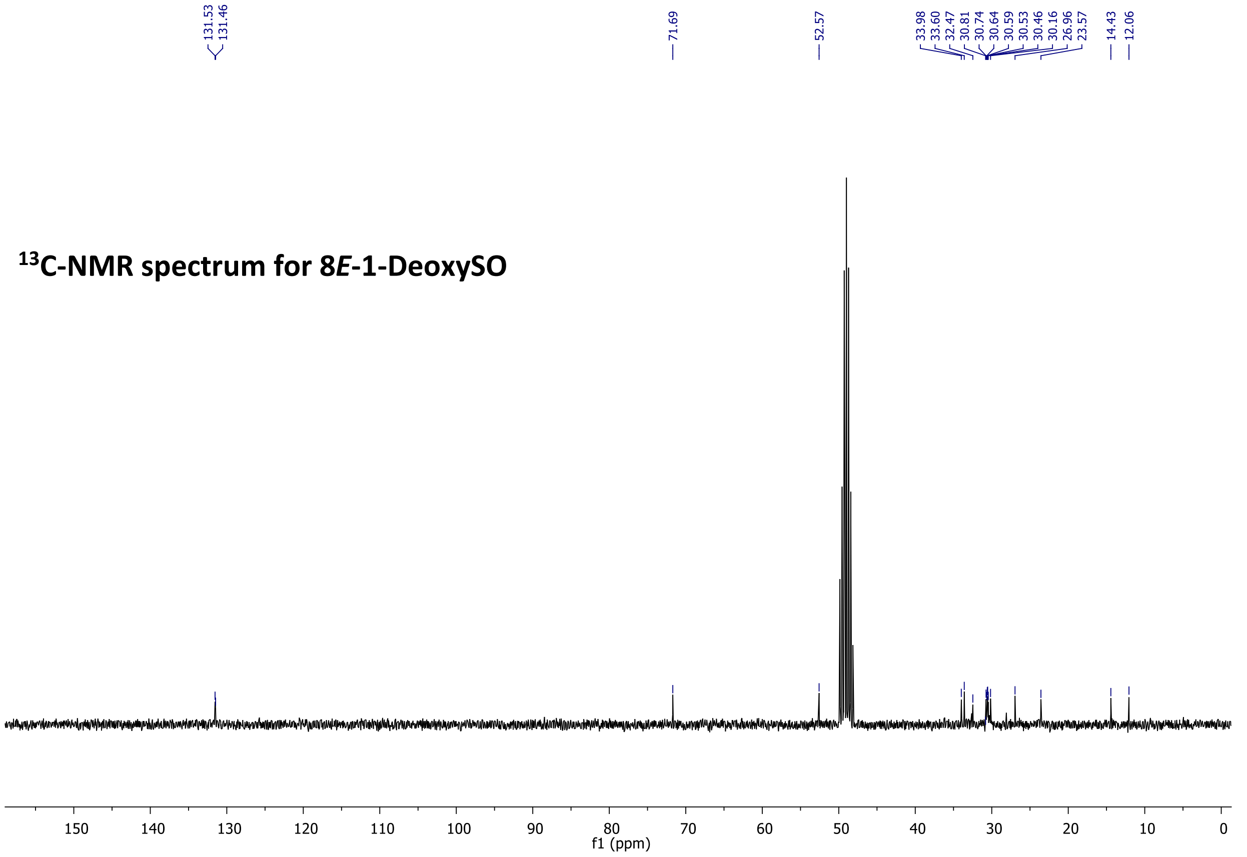

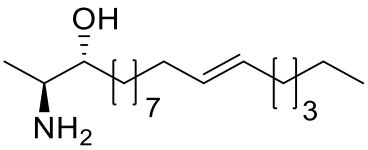

12*E*-1-DeoxySO

R_f: 0.45 (EtOAc/iso-propanol 4:1, visualized with $KMnO_4$ solution).

^{1}H NMR (MeOD, 500 MHz, ppm): δ 5.46 – 5.28 (m, 2H), 3.66 (m, 1H), 3.22 (m, 1H), 2.07 – 1.88 (m, 4H), 1.53 – 1.20 (m, 20H), 1.17 (d, *J* = 6.7 Hz, 3H), 0.85 (t, *J* = 6.4 Hz, 3H).

^{13}C NMR (MeOD, 126 MHz, ppm): δ 131.71, 131.13, 71.56, 52.49, 33.81, 33.61, 33.47, 33.01, 30.72, 30.68, 30.56, 30.42, 30.21, 26.39, 23.69, 14.46, 12.01.

ESI-MS: *m/z* calculated for $C_{18}H_{38}NO$ $[M+H]^+$ 284.29; observed 284.3.

^{1}H-NMR spectrum for 12*E*-1-DeoxySO

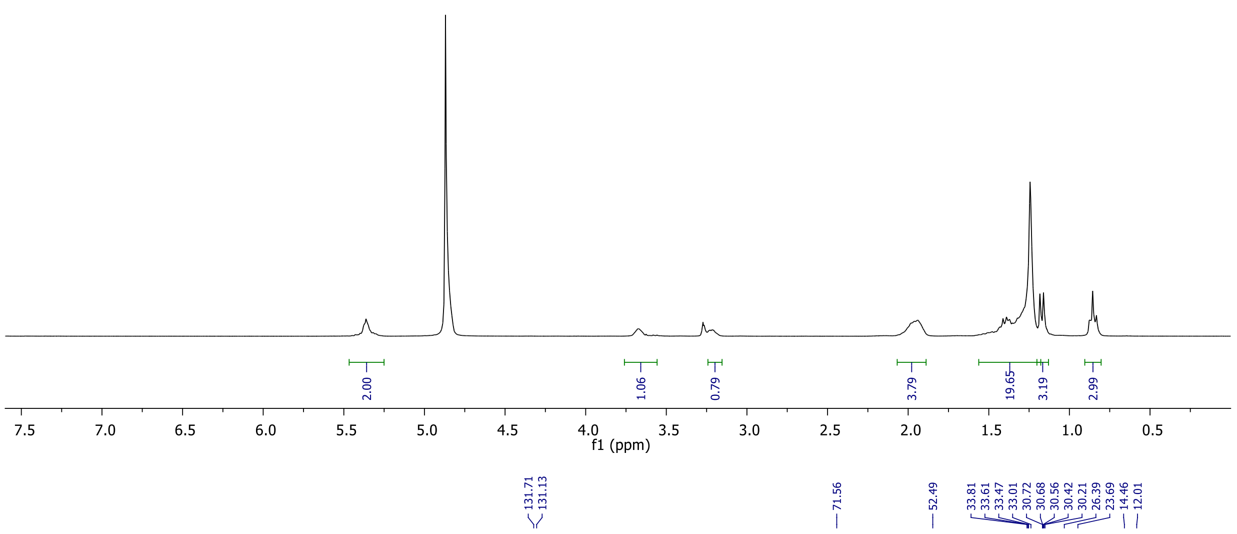

^{13}C-NMR spectrum for 12*E*-1-DeoxySO

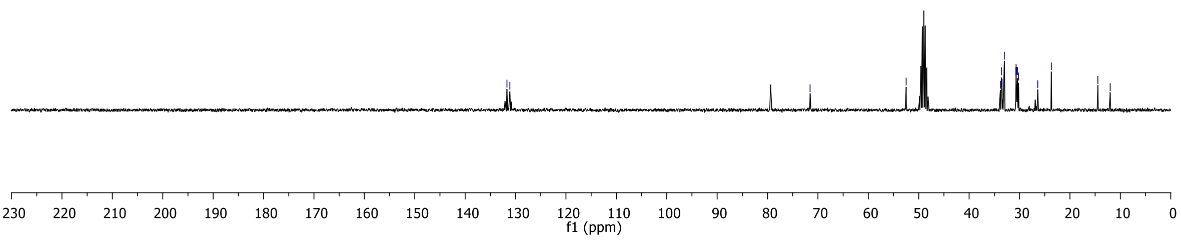

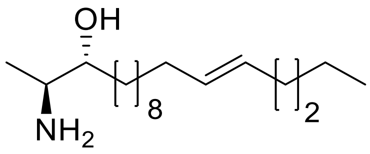

13*E*-1-DeoxySO

R_f: 0.45 (EtOAc/iso-propanol 4:1, visualized with $KMnO_4$ solution).

^{1}H NMR ($CDCl_3$, 500 MHz, ppm): δ 5.45 – 5.32 (m, 2H), 3.86 (m, 1H), 3.36 (m, 1H), 2.05-1.92 (m, 4H), 1.52 – 1.19 (m, 23H), 0.88 (t, J = 7.0 Hz, 3H).

^{13}C NMR ($CDCl_3$, 126 MHz, ppm): δ 130.46, 130.00, 70.93, 51.84, 32.90, 32.76, 32.43, 31.97, 29.82, 29.70, 29.64, 29.56, 29.32, 25.91, 22.33, 14.09, 11.40.

ESI-MS: *m/z* calculated for $C_{18}H_{37}NONa$ $[M+Na]^+$ 306.27; observed 306.3.

^{1}H-NMR spectrum for 13*E*-1-DeoxySO

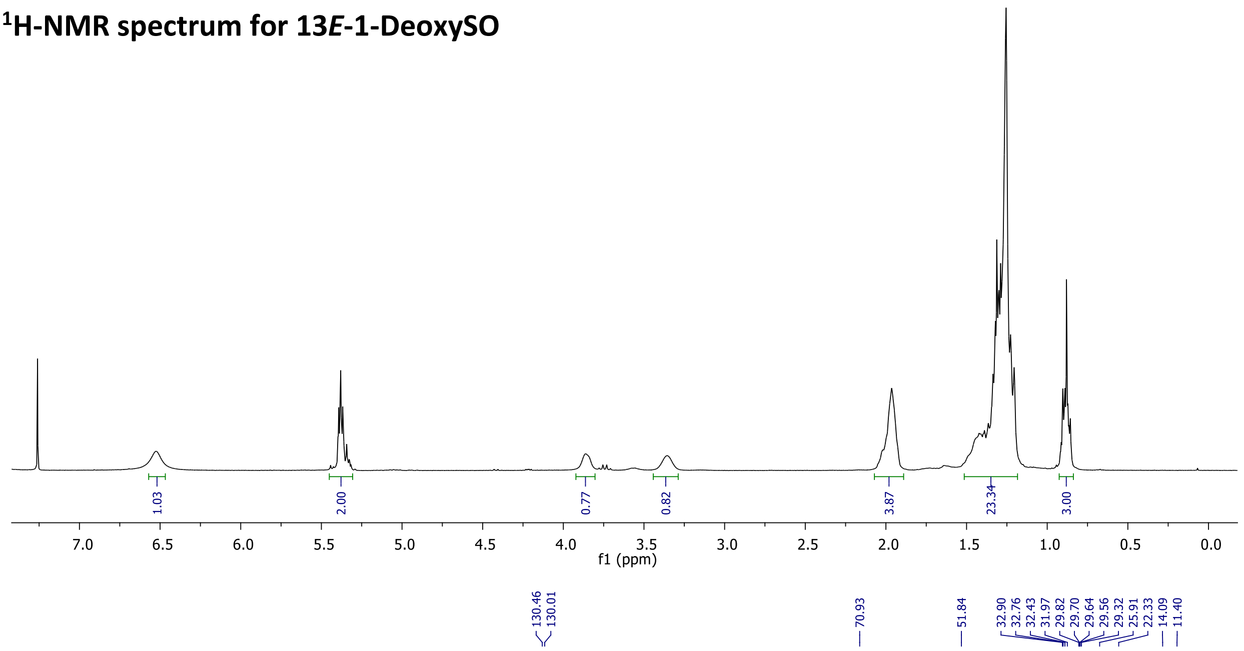

^{13}C-NMR spectrum for 13*E*-1-DeoxySO

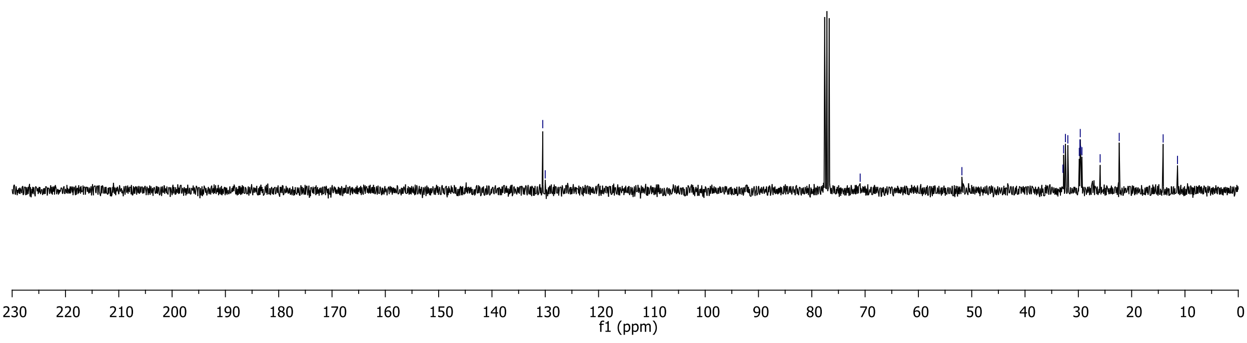

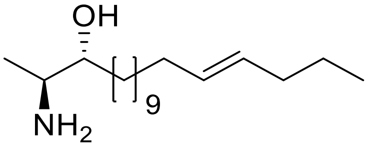

14*E*-1-DeoxySO

R_f: 0.45 (EtOAc/iso-propanol 4:1, visualized with $KMnO_4$ solution).

^{1}H NMR ($CDCl_3$, 500 MHz, ppm): δ 5.46 – 5.31 (m, 2H), 4.28 (br.s, 1H), 3.85 (m, 1H), 3.32 (m, 1H), 2.05-1.91 (m, 4H), 1.53 – 1.20 (m, 23H), 0.88 (t, J = 7.3 Hz, 3H).

^{13}C NMR ($CDCl_3$, 126 MHz, ppm): δ 130.71, 130.24, 70.70, 51.62, 34.86, 32.86, 32.78, 29.83, 29.78, 29.68, 29.62, 29.35, 22.89, 13.79, 11.44.

ESI-MS: *m/z* calculated for $C_{18}H_{38}NO$ $[M+H]^+$ 284.29; observed 284.3.

^{1}H-NMR spectrum for 14*E*-1-DeoxySO

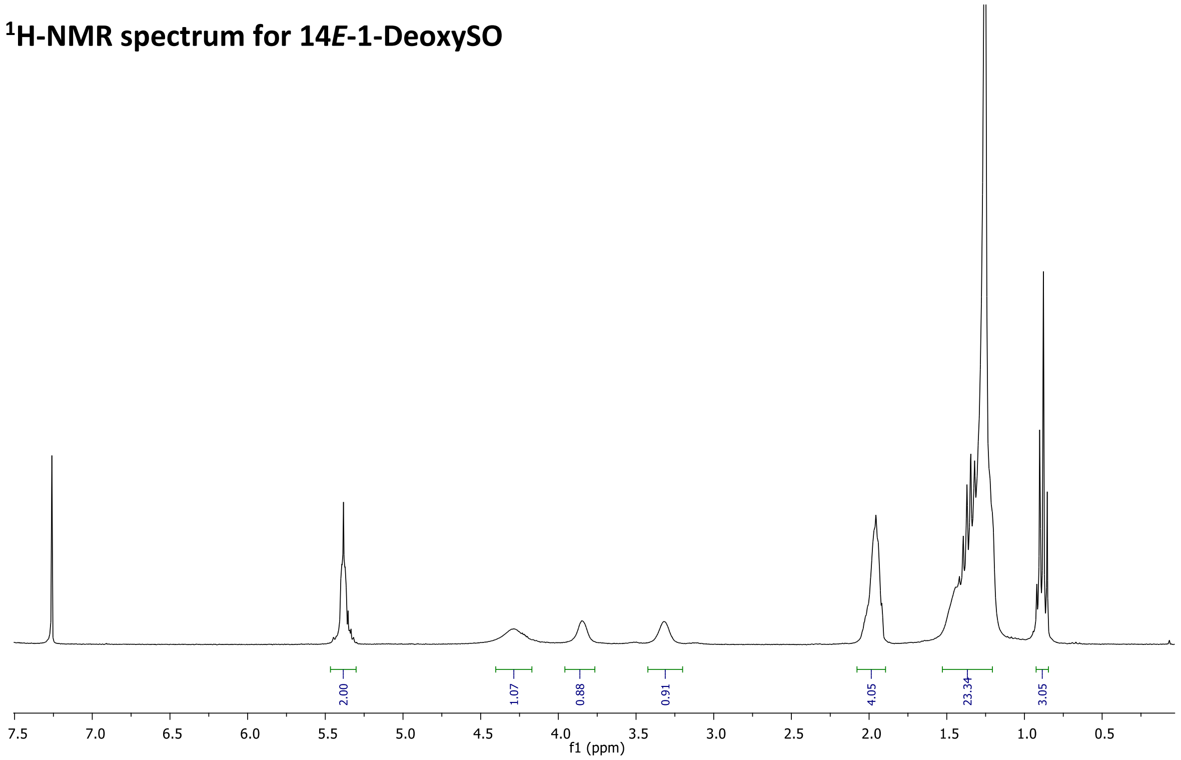

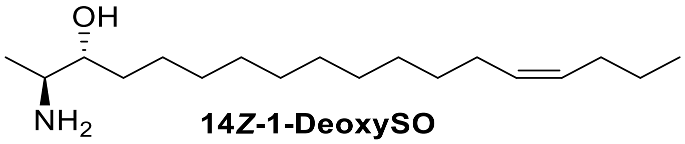

14*Z*-1-DeoxySO

R_f: 0.45 (EtOAc/iso-propanol 4:1, visualized with 1.3% ninhydrine).

^{1}H NMR (500 MHz, MeOD) δ 5.35 (ddd, *J* = 5.9, 3.6, 2.8 Hz, 2H), 3.70 (ddd, *J* = 8.1, 5.3, 3.0 Hz, 1H), 3.26 (qd, *J* = 6.7, 3.1 Hz, 1H), 2.07 – 1.98 (m, 4H), 1.47 – 1.28 (m, 20H), 1.21 (d, *J* = 6.8 Hz, 3H), 0.91 (t, *J* = 7.4 Hz, 3H).

^{13}C NMR (126 MHz, MeOD) δ 131.05, 130.65, 71.71, 52.61, 34.02, 30.86, 30.75, 30.73, 30.69, 30.65, 30.35, 30.31, 28.15, 27.00, 23.98, 14.16, 12.08.

ESI-MS: *m/z* calculated for $C_{18}H_{38}NO$ $[M+H]^+$ 284.29; found 284.3.

^{1}H-NMR spectrum for 14*Z*-1-DeoxySO

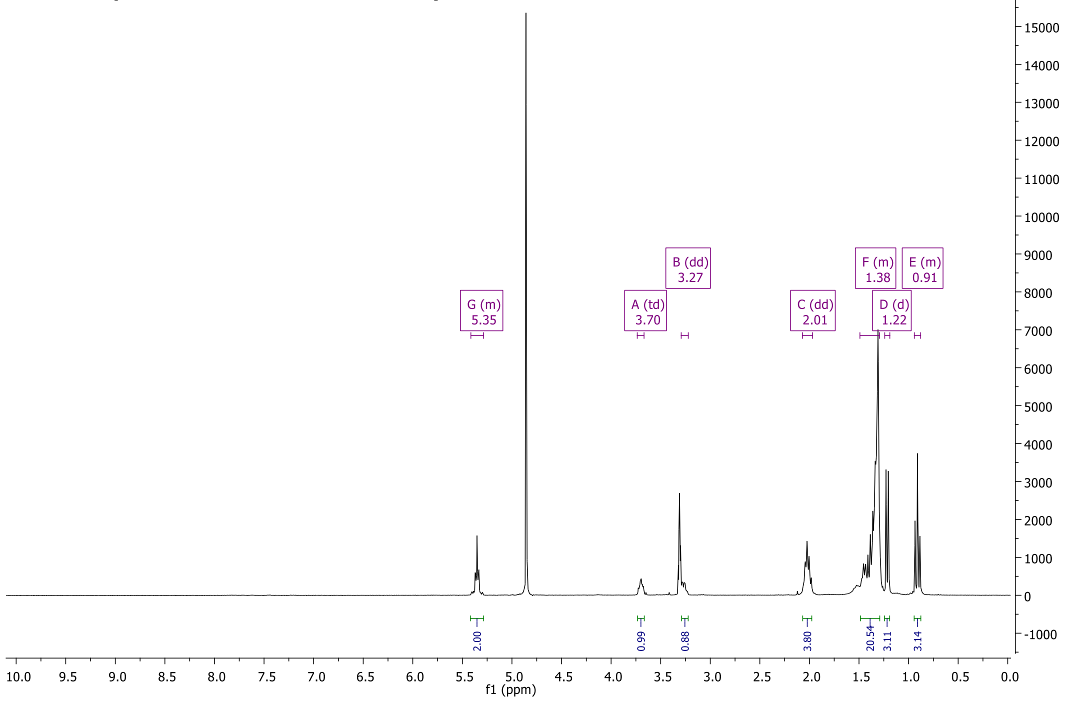

^{13}C-NMR spectrum for 14*Z*-1-DeoxySO

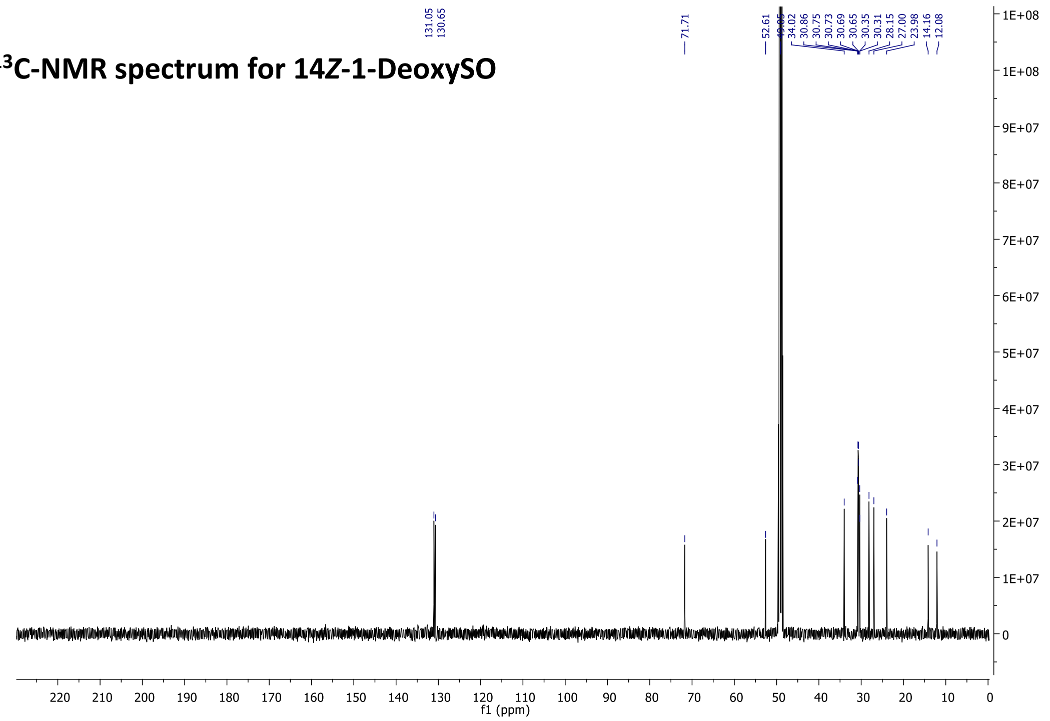

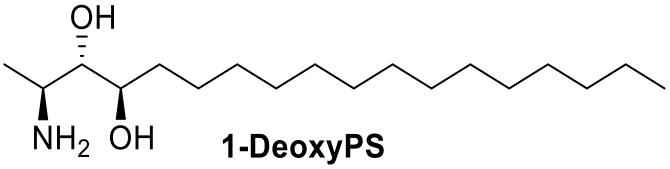

1-DeoxyPS

R_f: 0.27 (EtOAc/ iso-propanol 5:1, visualized with $KMnO_4$ solution).

^{1}H NMR (MeOD, 500 MHz, ppm) δ 3.58 (dt, *J* = 9.5, 4.7 Hz, 1H), 3.40 (ddd, *J* = 16.9, 11.8, 5.9 Hz, 2H), 1.82 (t, *J* = 9.5 Hz, 1H), 1.60 –1.52 (m, 1H), 1.40–1.26 (m, 24H), 1.25 (d, *J* = 6.7 Hz, 3H), 0.89 (t, *J* = 6.8 Hz, 3H).

^{13}C NMR (MeOD, 126 MHz, ppm) δ 74.7, 72.8, 50.4, 35.3, 33.1, 30.8, 30.8, 30.7, 30.5, 26.3, 23.7, 14.4, 11.9.

ESI-MS: *m/z* calculated for $C_{18}H_{40}NO_2$ $[M+H]^+$ 302.31; observed 302.3.

^{1}H-NMR spectrum for 1-DeoxyPS

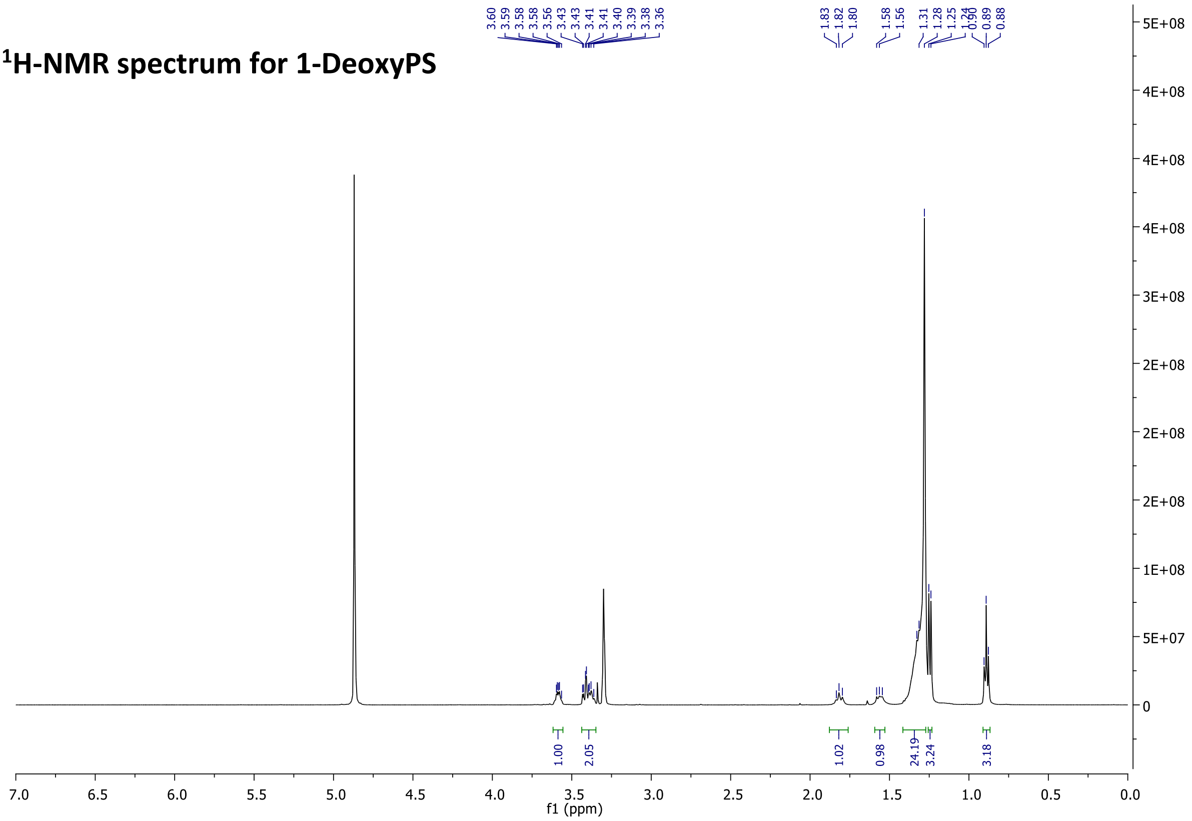

^{13}C-NMR spectrum for 1-DeoxyPS

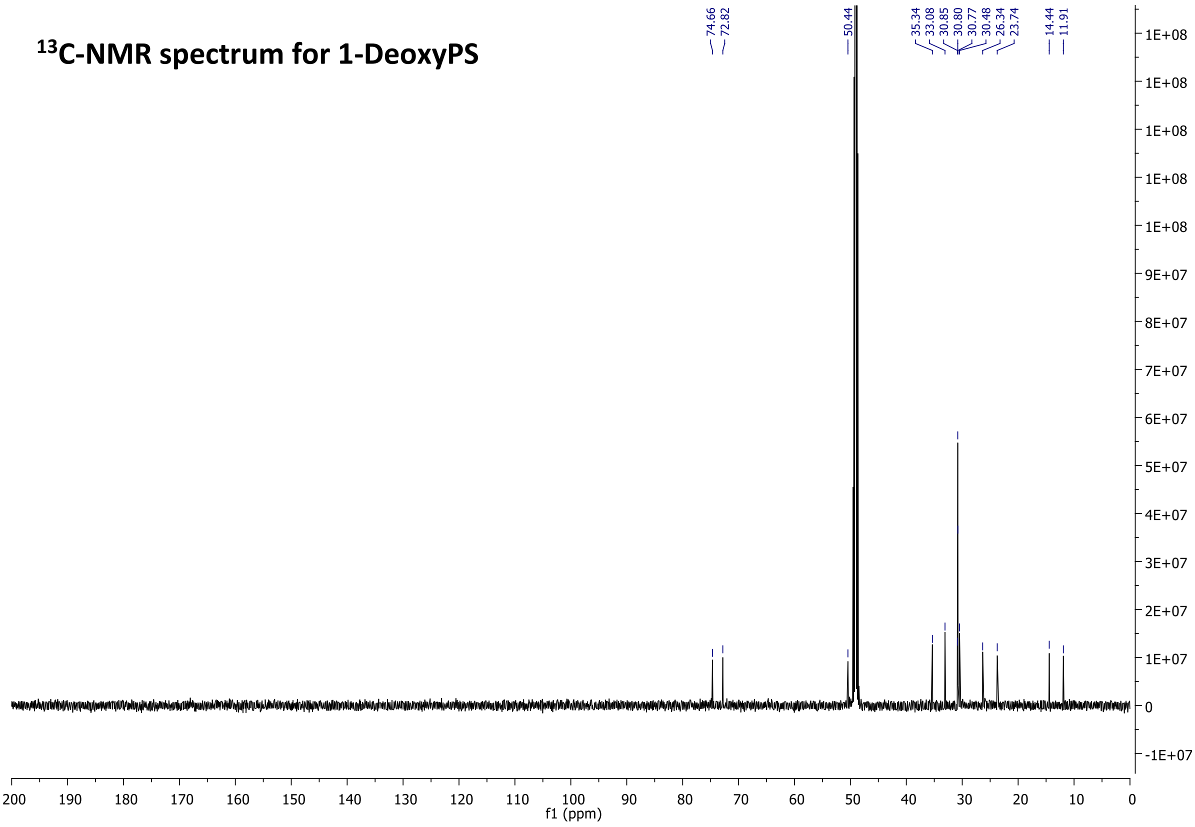

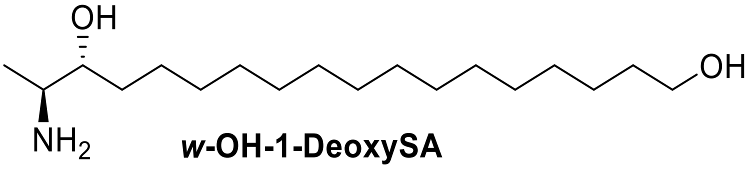

w-OH-1-DeoxySA

R$_f$: 0.48 (EtOAc/iso-propanol 4:1, visualized with $KMnO_4$ solution).

^{1}H NMR (MeOD, 500 MHz, ppm): δ 3.69 (ddd, *J* = 10.4, 5.3, 3.0 Hz, 1H), 3.54 (t, *J* = 6.7 Hz, 2H), 3.26 (qd, *J* = 6.7, 3.0 Hz, 1H), 1.52 (dt, *J* = 13.7, 6.8 Hz, 2H), 1.47 – 1.41 (m, 2H), 1.39-1.27 (m, 24H), 1.21 (d, *J* = 6.8 Hz, 3H).

^{13}C NMR (MeOD, 126 MHz, ppm): δ 71.65, 63.01, 52.58, 34.00, 33.67, 30.76, 30.67, 30.61, 26.96, 12.02.

ESI-MS: *m/z* calculated for $C_{18}H_{39}NO_2Na$ $[M+Na]^+$ 324.28; observed 324.3.

^{1}H-NMR spectrum for *w*-OH-1-DeoxySA

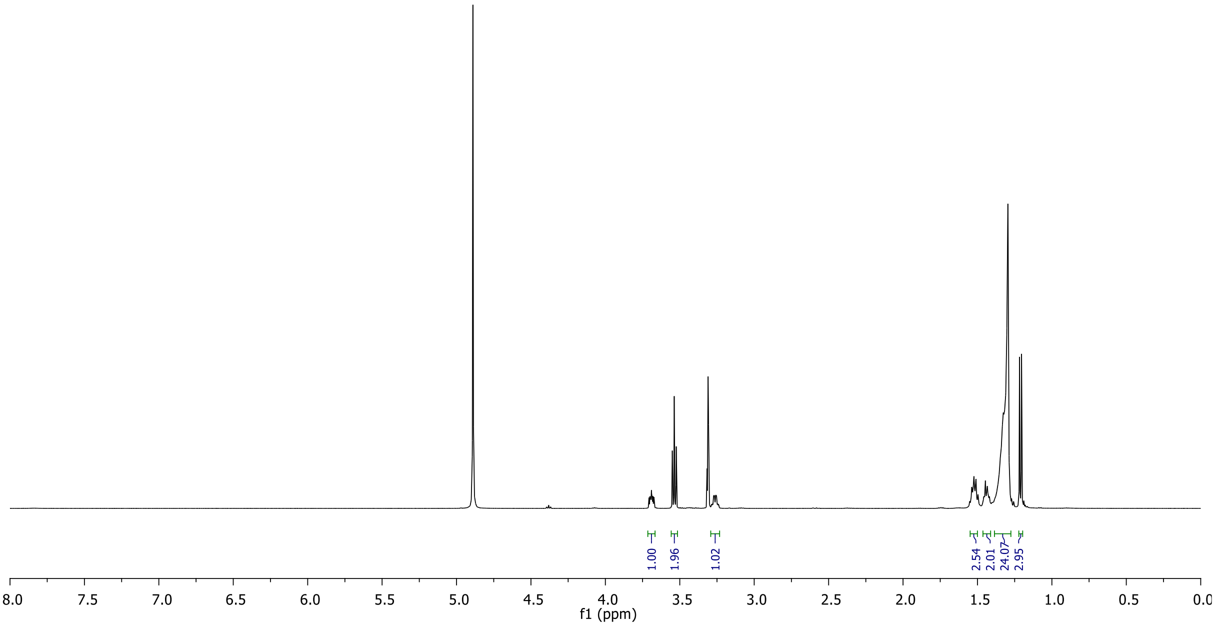

^{13}C-NMR spectrum for *w*-OH-1-DeoxySA

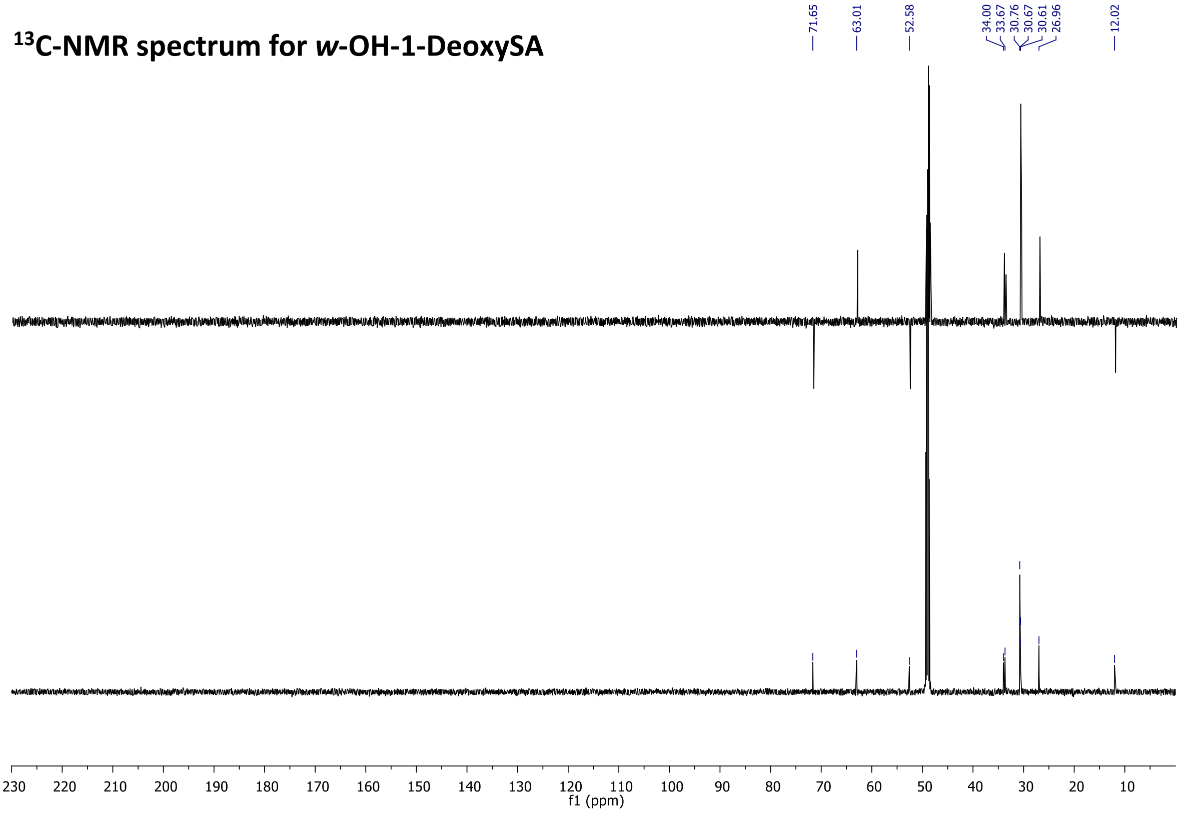

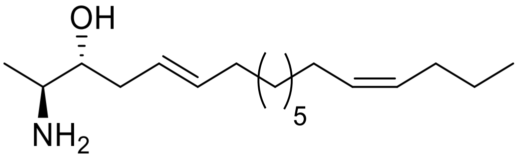

5*E*,14*Z*-1-Deoxysphingadiene

R_f: 0.43 (EtOAc/iso-propanol 4:1, visualized with $KMnO_4$ solution).

^{1}H NMR (MeOD, 500 MHz, ppm) δ 5.58 (dt, *J* = 15.0, 6.8 Hz, 1H), 5.47 – 5.39 (m, 1H), 5.39 – 5.32 (m, 2H), 3.76 – 3.72 (m, 1H), 3.28 (tt, *J* = 6.7, 3.2 Hz, 1H), 2.28 – 2.13 (m, 2H), 2.10 – 1.99 (m, 6H), 1.41 – 1.29 (m, 12H), 1.22 (d, *J* = 6.8 Hz, 3H), 0.91 (t, *J* = 7.4 Hz, 3H).

^{13}C NMR (MeOD, 126 MHz, ppm) δ 135.30, 131.00, 130.62, 126.14, 71.67, 51.87, 37.71, 33.69, 30.84, 30.53, 30.49, 30.29, 30.26, 28.12, 23.95, 14.11, 11.64.

ESI-MS: *m/z* calculated for $C_{18}H_{35}NONa$ $[M+Na]^+$ 304.26; observed 304.3.

^{1}H-NMR spectrum for 5*E*,14*Z*-1-Deoxysphingadiene

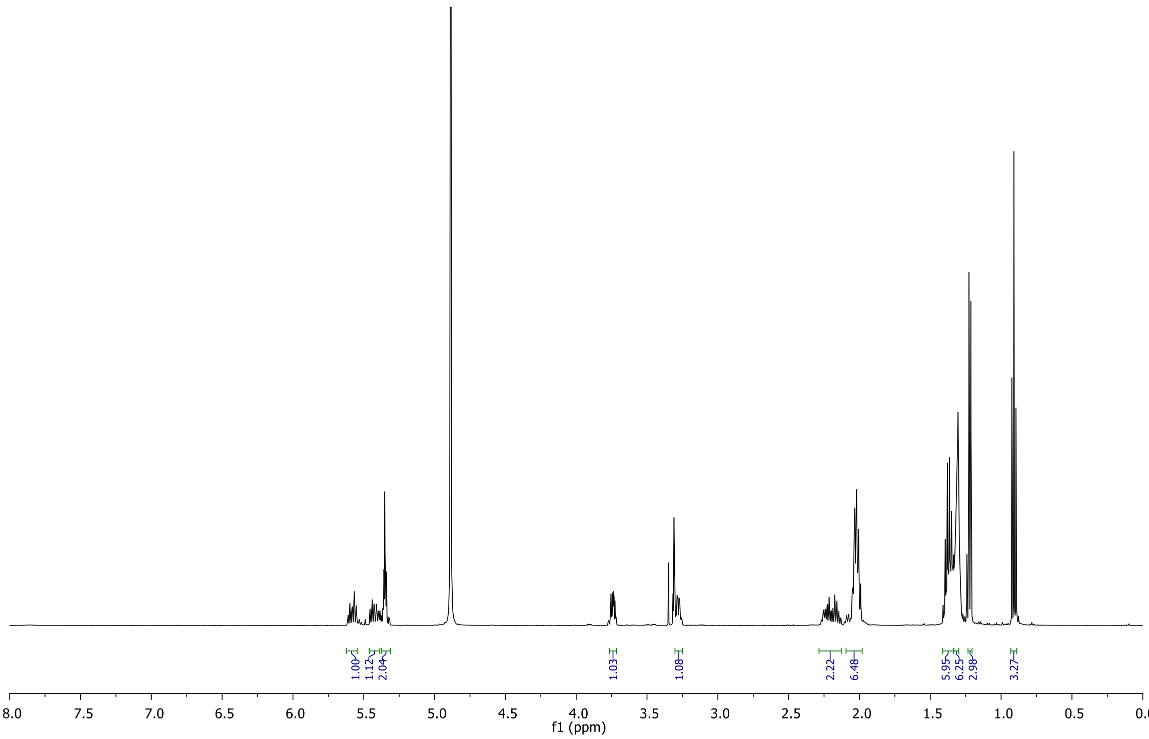

^{13}C-NMR spectrum for 5*E*,14*Z*-1-Deoxysphingadiene

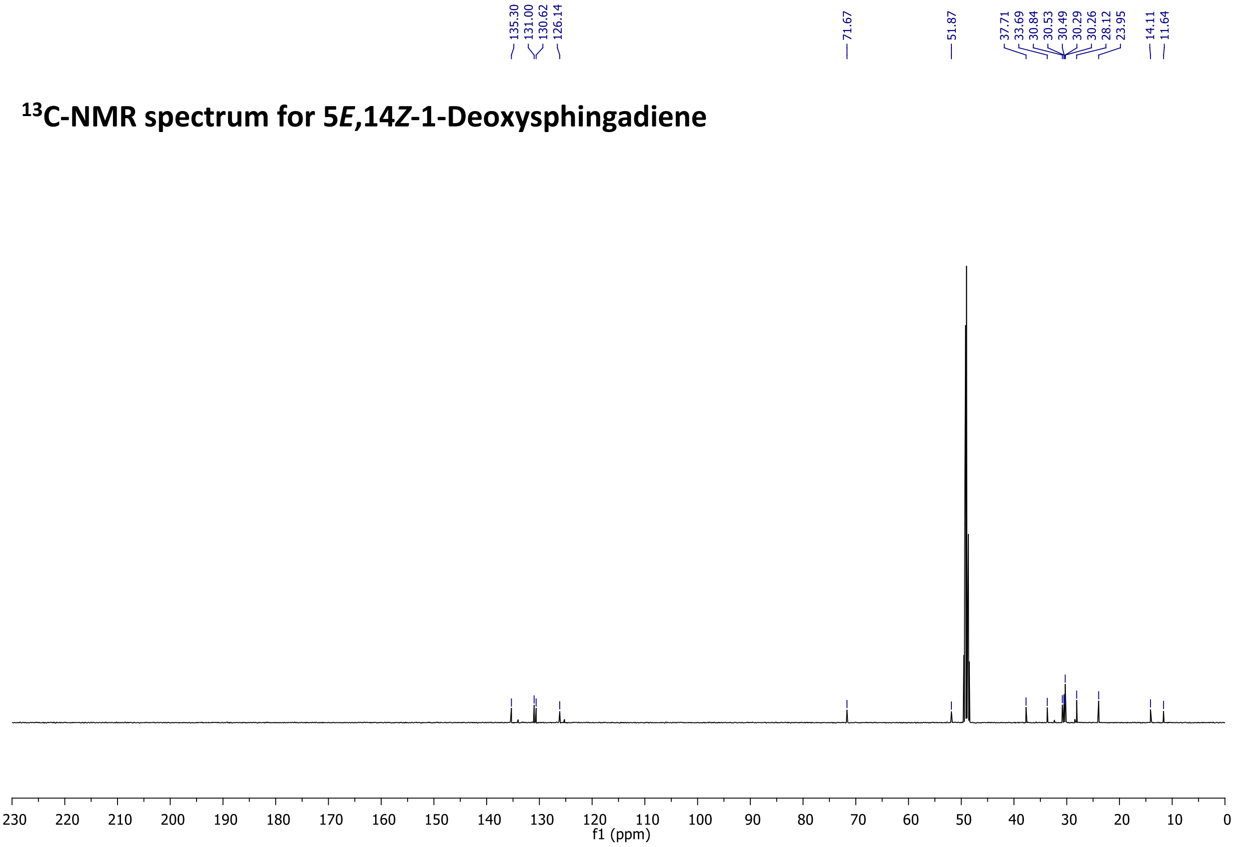

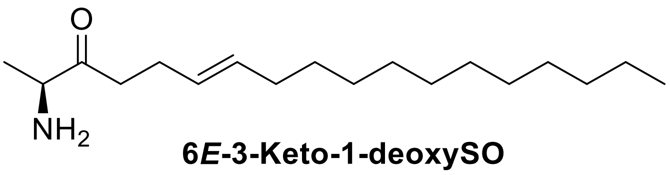

6*E*-3-Keto-1-deoxySO

R_f: 0.49 (EtOAc/iso-propanol 4:1, visualized with $KMnO_4$ solution).

^{1}H NMR (MeOD, 500 MHz, ppm) δ 5.55 – 5.47 (m, 1H), 5.45 – 5.39 (m, 1H), 4.14 (q, *J* = 7.3 Hz, 1H), 2.76 – 2.69 (m, 1H), 2.66 – 2.58 (m, 1H), 2.35 – 2.27 (m, 2H), 1.98 (q, *J* = 6.6 Hz, 2H), 1.53 – 1.49 (m, 4H), 1.37 – 1.27 (m, 17H), 0.90 (t, *J* = 7.0 Hz, 3H).

^{13}C NMR (MeOD, 126 MHz, ppm) δ 206.76, 133.10, 129.15, 55.86, 39.32, 33.57, 33.07, 30.78, 30.74, 30.60, 30.46, 30.27, 27.21, 23.73, 15.66, 14.43.

ESI-MS: *m/z* calculated for $C_{11}H_{36}NO$ $[M+H]^+$ 282.28; observed 282.3.

^{1}H-NMR spectrum for 6*E*-3-Keto-1-deoxySO

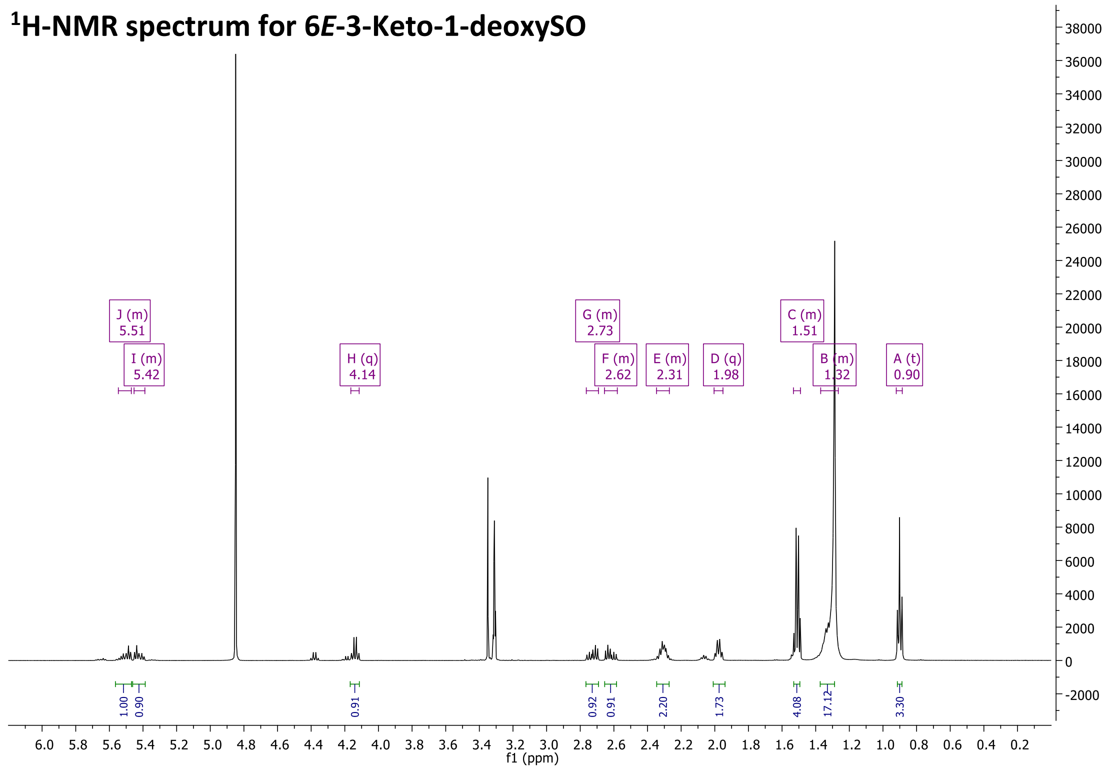

^{13}C-NMR spectrum for6*E*-3-Keto-1-deoxySO

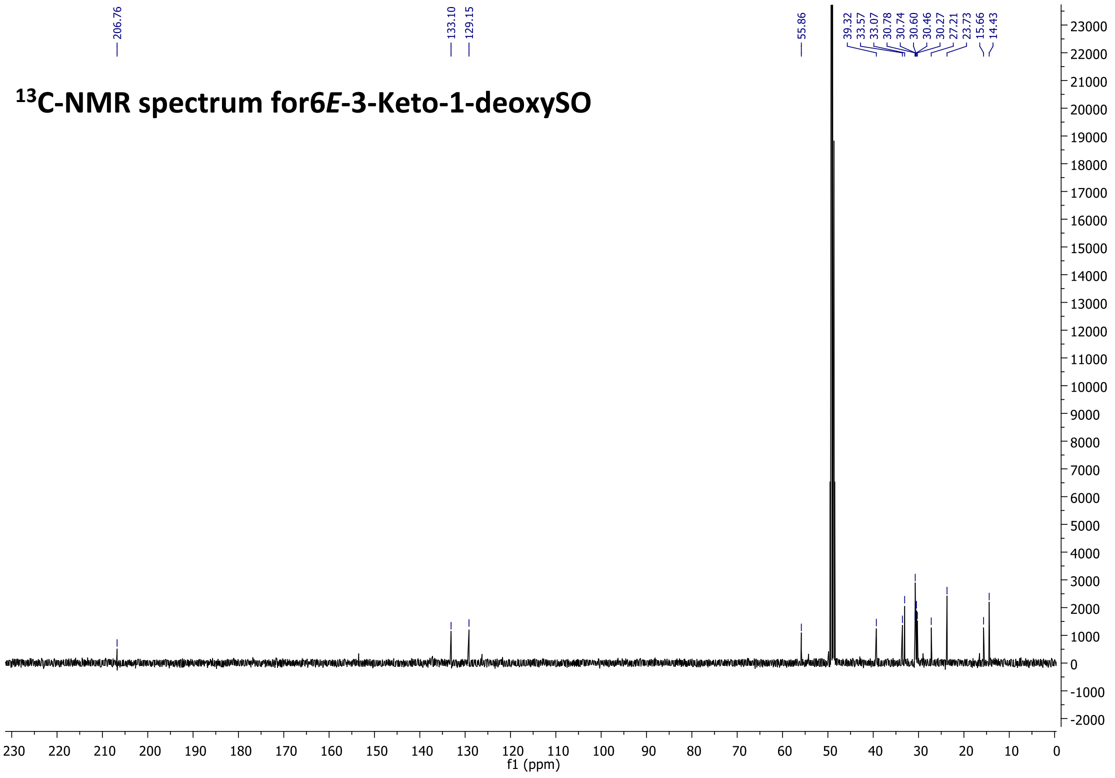

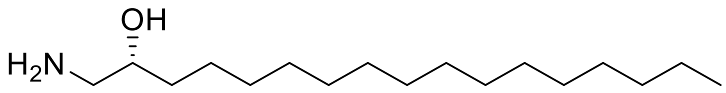

1-DeoxymethylSA

R_f: 0.35 (EtOAc/iso-propanol 5:1, visualized with 1.3% ninhydrine).

^{1}H NMR (MeOD, 500 MHz, ppm) δ 3.77 – 3.70 (m, 1H), 3.01 (dd, *J* = 12.7, 3.0 Hz, 1H), 2.75 (dd, *J* = 12.7, 9.5 Hz, 1H), 1.48 (t, *J* = 7.8 Hz, 2H), 1.43 – 1.23 (m, 26H), 0.90 (t, *J* = 6.9 Hz, 3H).

^{13}C NMR (MeOD, 126 MHz, ppm) δ 68.76, 46.09, 36.03, 33.07, 30.78, 30.72, 30.67, 30.64, 30.46, 26.40, 23.73.

ESI-MS *m/z* calculated for $C_{17}H_{38}NO$ $[M+H]^+$: 272.29; observed 272.3.

^{1}H-NMR spectrum for 1-DeoxymethylSA

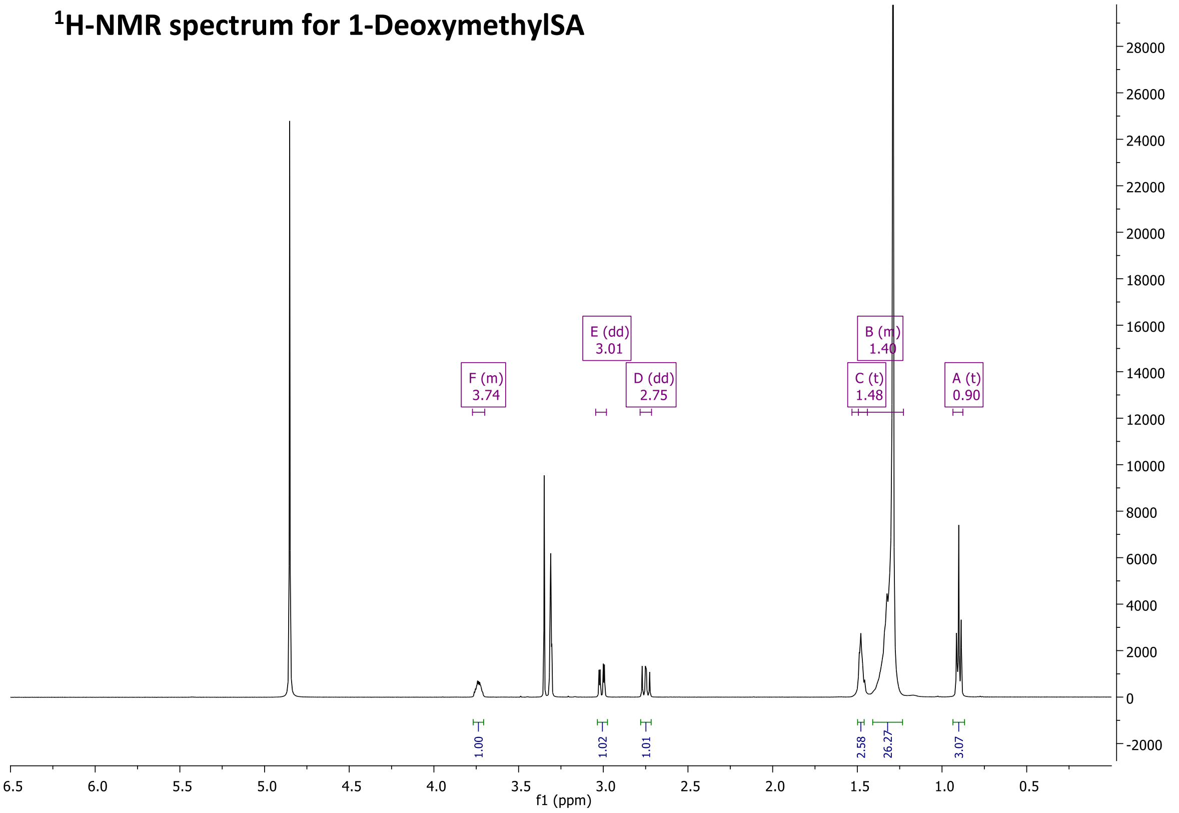

^{13}C-NMR spectrum for 1-DeoxymethylSA

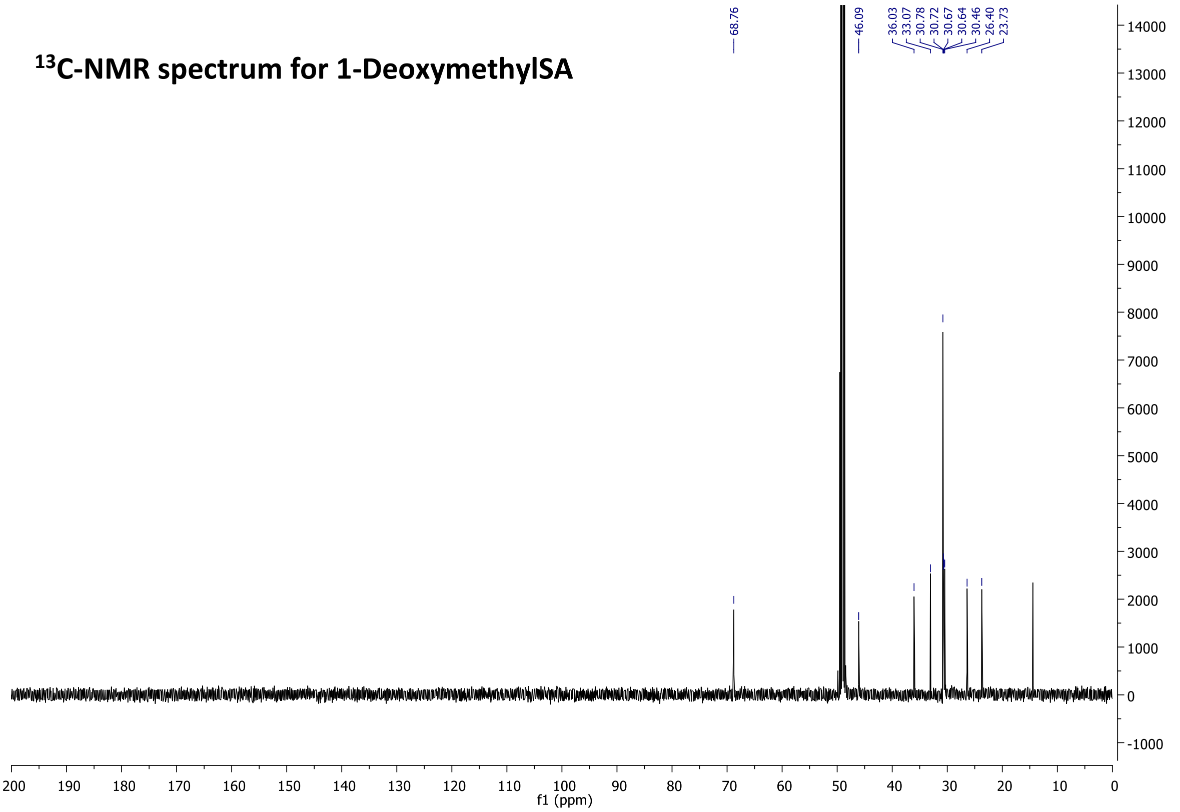

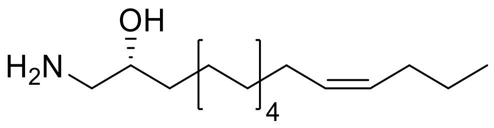

13*Z*-1-DeoxymethylSO

R_f: 0.36 (EtOAc/iso-propanol 5:1, visualized with 1.3% ninhydrine).

^{1}H NMR (MeOD, 500 MHz, ppm) δ 5.34 (ddd, J = 5.9, 3.3 Hz, 2H), 3.57 (dd, *J* = 11.5, 8.9 Hz, 1H), 2.82 – 2.54 (m, 2H), 2.07 – 1.93 (m, 4H), 1.52 – 1.28 (m, 20H), 0.91 (t, *J* = 7.4 Hz, 3H).

^{13}C NMR (MeOD, 126 MHz, ppm) δ 131.03, 130.60, 72.01, 47.65, 36.01, 33.65, 30.86, 30.78, 30.74, 30.65, 30.35, 30.30, 28.14, 26.67, 23.96, 14.14.

ESI-MS: *m/z* calculated for $C_{17}H_{36}NO$ $[M+H]^+$: 270.28; observed 270.3.

^{1}H-NMR spectrum for 13*Z*-1-DeoxymethylSO

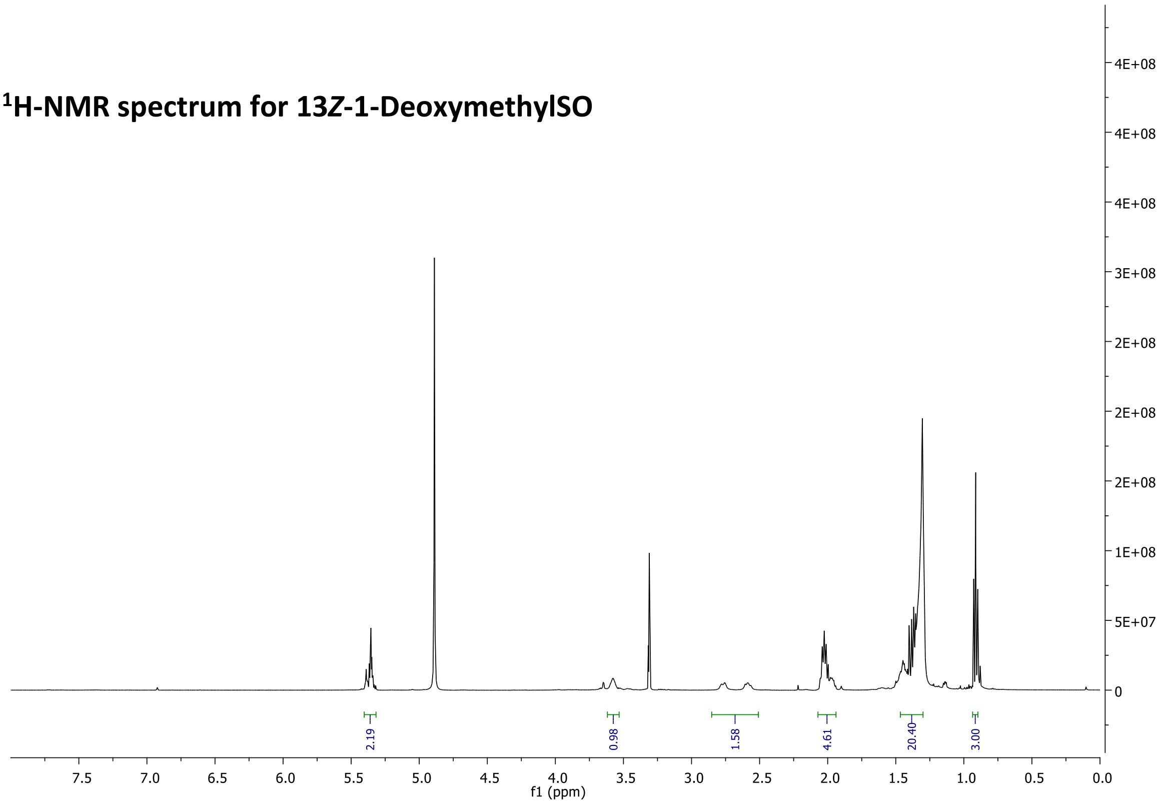

^{13}C-NMR spectrum for 13*Z*-1-DeoxymethylSO

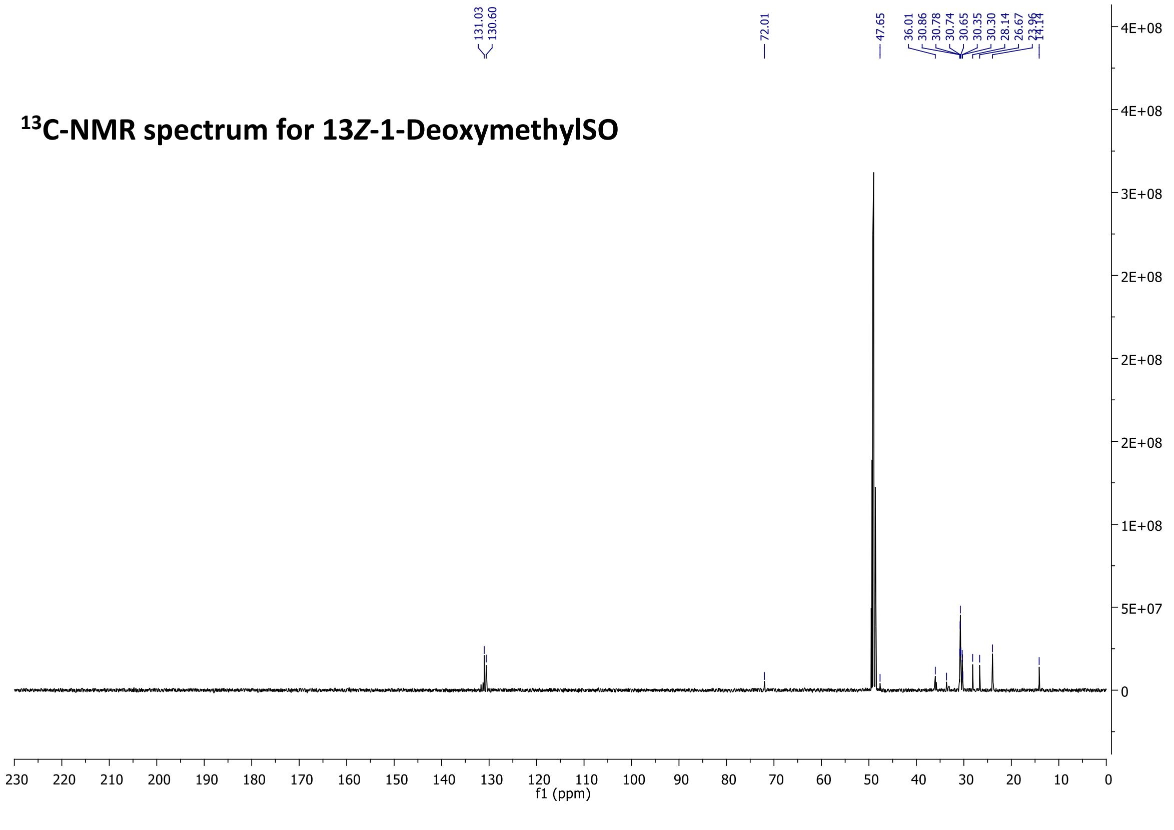

References

[1] S. J. Allen, K. Giles, T. Gilbert, M. F. Bush, *Analyst* **2016**, *141*, 884-891.

[2] H. E. Revercomb, E. A. Mason, *Anal. Chem.* **2002**, *47*, 970-983.

[3] E. Mucha, et al., *Angew. Chem. Int. Ed.* **2017**, *56*, 11248-11251.

[4] W. Schöllkopf, S. Gewinner, H. Junkes, A. Paarmann, G. von Helden, H. Bluem, A. M. M. Todd, *Proc. SPIE* **2015**, *9512*, 95121L.

[5] A. Supady, V. Blum, C. Baldauf, *J. Chem. Inf. Model.* **2015**, *55*, 2338-2348.

[6] a) J. P. Perdew, K. Burke, M. Ernzerhof, *Phys. Rev. Lett.* **1996**, *77*, 3865-3868; b) V. Blum, R. Gehrke, F. Hanke, P. Havu, V. Havu, X. Ren, K. Reuter, M. Scheffler, *Comput. Phys. Commun.* **2009**, *180*, 2175-2196; c) A. Tkatchenko, M. Scheffler, *Phys. Rev. Lett.* **2009**, *102*, 073005.

[7] a) C. Adamo, V. Barone, *J. Chem. Phys.* **1999**, *110*, 6158-6170; b) M. J. Frisch, et al., Gaussian, Inc.,Wallingford, CT, **2016**.

[8] B. L. J. Poad, et al., *Anal. Chem.* **2018**, *90*, 5343-5351.

[9] a) R. Steiner, E. M. Saied, A. Othman, C. Arenz, A. T. Maccarone, B. L. Poad, S. J. Blanksby, A. von Eckardstein, T. Hornemann, *J. Lipid Res.* **2016**, *57*, 1194-1203; b) E. M. Saied, T. L. Le, T. Hornemann, C. Arenz, *Bioorg. Med. Chem.* **2018**, *26*, 4047-4057; c) J. Ren, E. M. Saied, A. Zhong, J. Snider, C. Ruiz, C. Arenz, L. M. Obeid, G. D. Girnun, Y. A. Hannun, *J. Lipid Res.* **2018**, *59*, 2126-2139.

[10] E. M. Saied, C. Arenz, **2020**, ChemRxiv preprint DOI 10.26434/chemrxiv.12094041.v12094041.
